# *In Silico* Structure and Sequence Analysis of Bacterial Porins and Specific Diffusion Channels for Hydrophilic Molecules: Conservation, Multimericity and Multifunctionality

**DOI:** 10.3390/ijms17040599

**Published:** 2016-04-21

**Authors:** Hilde S. Vollan, Tone Tannæs, Gert Vriend, Geir Bukholm

**Affiliations:** 1 Department of Clinical Molecular Biology (EpiGen), Division of Medicine, Akershus University Hospital and University of Oslo, PO box 28, N-1478 Lørenskog, Norway; h.s.vollan@studmed.uio.no (H.S.V.); t.m.tannas@medisin.uio.no (T.T.); 2 Norwegian Institute of Public Health, Box 4404 Nydalen, N-0403 Oslo, Norway; 3 Centre for Molecular and Biomolecular Informatics, Radboud University Medical Center, 6525 GA Nijmegen, The Netherlands; vriend@cmbi.ru.nl; 4 Department of Chemistry, Biotechnology and Food Science, Norwegian University of Life Sciences, Pb 5003, N-1430 Ås, Norway

**Keywords:** β-barrel membrane proteins, bacteria, porins, specific diffusion channels, structure analysis, multiple sequence analysis, entropy-variability analysis

## Abstract

Diffusion channels are involved in the selective uptake of nutrients and form the largest outer membrane protein (OMP) family in Gram-negative bacteria. Differences in pore size and amino acid composition contribute to the specificity. Structure-based multiple sequence alignments shed light on the structure-function relations for all eight subclasses. Entropy-variability analysis results are correlated to known structural and functional aspects, such as structural integrity, multimericity, specificity and biological niche adaptation. The high mutation rate in their surface-exposed loops is likely an important mechanism for host immune system evasion. Multiple sequence alignments for each subclass revealed conserved residue positions that are involved in substrate recognition and specificity. An analysis of monomeric protein channels revealed particular sequence patterns of amino acids that were observed in other classes at multimeric interfaces. This adds to the emerging evidence that all members of the family exist in a multimeric state. Our findings are important for understanding the role of members of this family in a wide range of bacterial processes, including bacterial food uptake, survival and adaptation mechanisms.

## 1. Introduction

**Figure 1:**
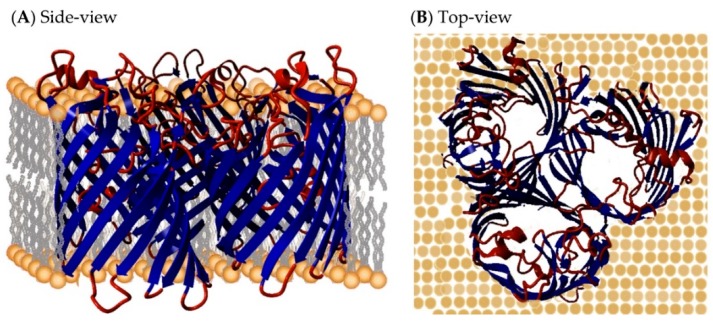
Outer membrane protein (OMP) structure. OMP structures are composed of a transmembrane motif that consists of β-strands that form a transmembrane β-barrel. These strands are connected by short loops (also called turns) in the periplasm and longer extracellular loops. The number of β-strands, the length of the β-strands and the loop lengths vary widely among OMPs. Here, the trimeric general diffusion porin (GDP) from *E. coli* (PDB (Protein Data Bank) ID: 2J1N, [4]) is shown as an example from (**A**) the side-view with the ß-barrel core embedded in the membrane and (**B**) the top-view, where the pores are visible viewed from the extracellular side. The β-barrel with loops and turns removed (blue) is referred to as the core.

Bacteria thrive in an extremely wide range of environments. They can evolve rapidly to cope with continuously-changing environments. The cell envelope provides their main line of defense against their surroundings. The cell envelope must carefully discriminate between external molecules that are useful, e.g., nutrients, and those that are harmful, e.g., antibiotics. Gram-positive bacteria have a cell wall that consists of a single membrane covered with a thick peptidoglycan layer, while Gram-negative bacteria have two membranes that are separated by the periplasm [1,2]. Their outer membrane contains several outer membrane proteins (OMPs), which typically consist of a transmembrane barrel comprising of β-strands that are connected by short periplasmic loops (also called turns) and longer extracellular loops (see Figure 1) [3]. These OMPs regulate the passage of molecules through the outer membrane.

**Figure 2:**
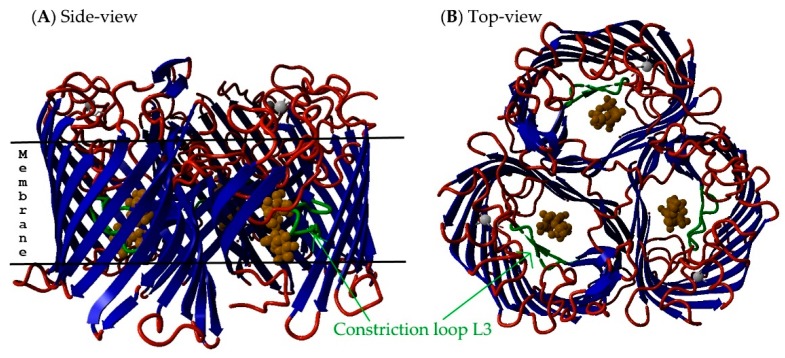
Porin structure. The constriction loop L3 of sugar-specific *Salmonella* Typhimurium ScrY porin is highlighted in green (PDB ID: 1OH2, [12]). The barrel core is colored blue; periplasmic turns and extracellular loops are red; the substrate is orange; and calcium ions are gray. (**A**) Side-view of the porin where strands 14, 15 and 16 of molecule P were removed to better visualize the constriction loop L3; (**B**) top-view of the porin.

Non-specific and specific diffusion channels form the largest OMP family, and they are involved in the selective uptake of molecules. The number of β-strands in a protein barrel ranges from eight to 26. The pore size and amino acid composition in the channel are the main determinants of porin specificity and function [3]. These proteins are often multifunctional, and they may also function as a bacteriocin receptor [5,6,7] or in host-cell interactions [8,9]. Non-specific channels, or porins, and specific diffusion channels are upregulated in the presence of nutrients and downregulated in the presence of toxins or other harmful molecules (e.g., antibiotics, heavy metals, detergents or bile salts) [10,11]. The gating mechanism of the larger channels is achieved through a constriction loop. This is usually the largest extracellular loop (loop L3) that is located inside the barrel (see Figure 2). Unlike other extracellular loops, this loop never faces the environment. The amino acid composition of this loop determines the substrate specificity of the pore [5].

Outer membrane protein channels involved in the diffusion of hydrophilic molecules have been divided into two groups in two different ways: specific diffusion channels *versus* non-specific porins [11,13,14,15,16,17] and monomeric *versus* trimeric channels [3,8,18]. Although these divisions are not universally accepted [3,8,11,17,19], there appears to be little doubt that some OM channels are monomeric and others are trimeric [5,11,20]. The subdivisions of the four main porin and specific diffusion channel groups, based on their multimeric state and specificity, are [3,8,11,13,15,21,22,23,24]:

1. Non-specific monomeric porins (NMPs): Outer membrane protein A (OmpA) and OmpG are NMPs. OmpA is an eight-stranded multifunctional protein where current research reveals porin activity [22,25,26,27], while OmpG is a 14-stranded, pH-dependent porin [5].
2. Non-specific trimeric porins (NTPs): NTPs, also known as general diffusion porins (GDPs), have 16-stranded β-barrels that allow the diffusion of hydrophilic substances smaller than 600 kDa [21].
3. Specific monomeric diffusion channels (SMDCs): The oligogalacturonate-specific KdgM channel, outer membrane porin B (OprB) and outer membrane carboxylate channel (Occ) proteins belong to the SMDC group. KdgM is an acidic, sugar-specific channel with a 12-stranded β-barrel [24]. OprB is a carbohydrate-specific channel with a 16-stranded β-barrel [23]. Finally, Occ porins are water-soluble, specific channel for small substrates with a carboxyl group with 18-stranded β-barrels [28,29].
4. Specific trimeric diffusion channels (STDCs): STDCs include OprP and maltoporins. OprP is a phosphate-specific porin that has a 16-stranded β-barrel [30]. Sugar-specific channels (maltoporins/ScrY) are slightly larger, as they consist of 18-stranded β-barrels [12,31].

The aim of this study was to examine the sequence- and structure-derived interaction patterns for non-specific porins and specific diffusion channels. To shed light on the structure-function relations of amino acids in these subclasses, MSAs (multiple sequence alignments) were constructed based on 3D structures of non-specific porins and specific diffusion channels. EVAs (Entropy-variability analyses) [32] were used to study the evolutionary footprints in the alignments.

## 2. Results

### 2.1. Data Collection

Eighty-nine unique OMP structures were extracted from the PDB, of which 34 belong to the porin and specific diffusion channel family. The 34 protein structures are listed in Table 1, together with biological and structural information.

The PDB contains structures of proteins, isolated from a wide variety of species, which engage in homotrimeric interactions (see Table 1). The NTP/GDP group contains structures isolated from γ-Proteobacteria (*Escherichia coli*, *Klebsiella pneumoniae* and *Salmonella enterica* serovar Typhimurium (*Salmonella* Typhimurium)), α-Proteobacteria (*Rhodobacter capsulatus* and *Rhodopseudomonas blastica*) and β-Proteobacteria (*Delftia acidovorans*). Despite the species variability among this group, they are all non-specific porins, and they have the same barrel size. The STDCs isolated from γ-Proteobacteria were subdivided into two groups based on their β-barrel size and specificity: the 18-stranded β-barrel, sugar-specific diffusion channels and the 16-stranded β-barrel, phosphate-specific channels.

### 2.2. Non-Specific Porin and Specific Diffusion Channel Secondary Structure Composition

The secondary structure composition of the four protein groups is summarized in Table 2. In total, 56.3% of the amino acids were found in β-strands; 30.8% were found in extracellular loops; and 12.5% were found in periplasmic turns. Their sizes varied from 171 (NMP; PDB ID 1QJP) to 422 (SMDC; PDB ID 3SY9) amino acids. The proportions of secondary structures remained roughly the same for all of the protein structures, with an average ratio of 6:1:3 for amino acids in the β-strand:periplasmic loop:extracellular loop.

### 2.3. Non-Specific Porin and Specific Diffusion Channel Phylogenetic Tree

**Figure 3:**
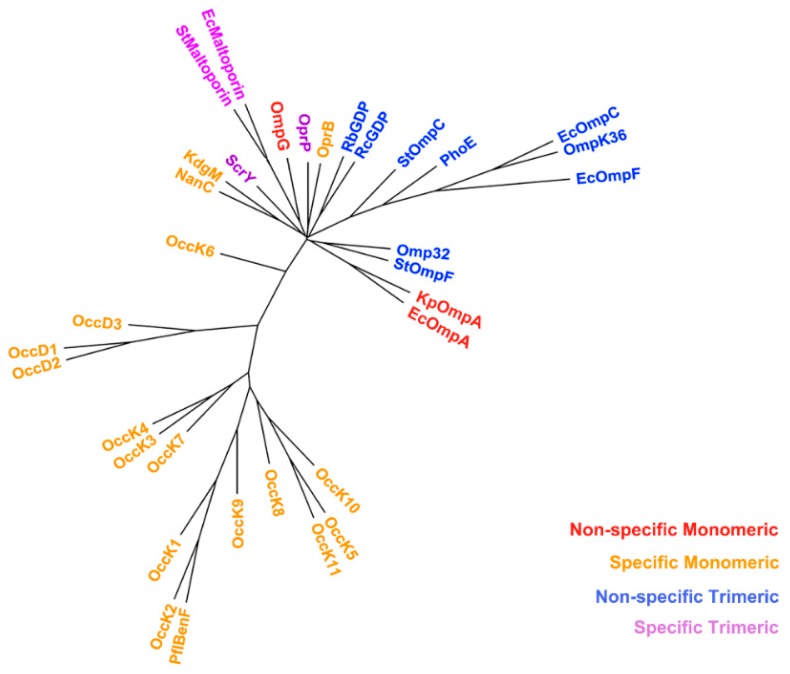
Phylogenetic tree of bacterial porins. Colors represent the four groups and the multimeric state of the porin based on the old classification system.

A phylogenetic tree, which was constructed from the structure-based alignment of the 34 non-specific porin and specific diffusion channel sequences, is depicted in Figure 3. The sequences were colored according to the four groups discussed in the Introduction.

### 2.4. Non-Specific Porin and Specific Diffusion Channel Classification System

Several different OMP classification systems are based on protein structures. Many databases group non-specific porin and specific diffusion channel with transporters, surface proteins or mitochondrial proteins. For example, the “Class, Architecture, Topology, Homology” (CATH) database [33], “The Transporter Classification Database” (TCDB) [34] and the “Outer Membrane Protein Database” (OMPdb) [35] do not distinguish non-specific porin and specific diffusion channel from other OMPs. However, the OMPdb has 12 non-specific porin and specific diffusion channel classes that are relevant to our analysis. The remaining 79 OMP classes were excluded because they lacked a solved structure or because they were not non-specific porin nor the specific diffusion channel (see the Discussion and Table S1 for how the relevant non-specific porin and specific diffusion channel families correspond to the eight subclasses used in our analysis). Finally, only three porins (Omp32, OmpF and PhoE) were included in the “Structural Classification of Proteins-extended” (SCOPe) superfamily [36].

The non-specific porin and specific diffusion channel family are often divided into four groups, depending on their specificity and multimericity (see the Introduction). These four groups were used in Table 1 and Table 2 and Figure 3. However, for the remaining analyses, we divided non-specific porins and specific diffusion channels into six classes with eight subclasses, based on their specificity and size. Table 3 lists class number and names, corresponding protein names (of structures solved to date) and PDB IDs.

### 2.5. Non-Specific Porin and Specific Diffusion Channel Structure Analysis

A superposition of non-specific porin and specific diffusion channel core structures resulted in an average root-mean-square deviation (RMSD) of 1.2 Å over 95.2% of the aligned residues, with a 51.7% sequence identity per class (ranging from RMSDs of 0.9 to 1.6 Å over 90.7 to 98.7% of the aligned residues, with sequence identities between 25.6% and 96.1%). These results are listed in Table 4, while an analysis of the entire non-specific porin and specific diffusion channel structure is found in Table S2 and Table S2raw (these tables compare the core structure analysis with whole structures and the sequence analysis). The structure alignment of specific, small channels (Class 3B; KdgM and NanC) resulted in the lowest sequence identity score, while OmpA structures (Class 1A) resulted in the highest average sequence identity. Three of the subclasses could not be analyzed because they currently hold only one structure (Classes 4A, 5B and 5C).

**Figure 4:**
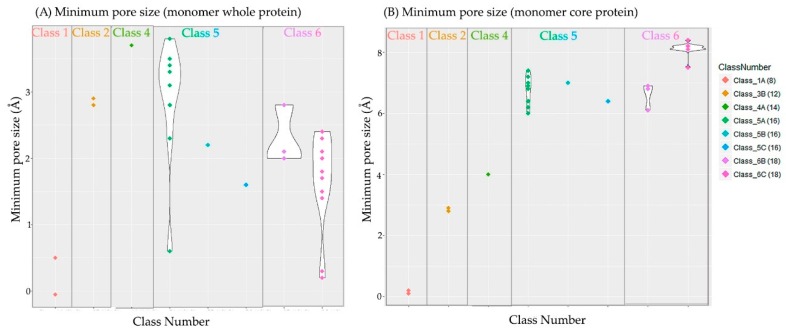
Estimated minimum Angstrom pore size radius for each subclass using the HOLE software [37]. Each colored diamonds represent one monomer structure. (**A**) Whole structure; (**B**) core structure where loops and turns were removed. The barrel size number of strands in each β-barrel) is listed in brackets next to the class name labels.

Minimum pore size estimates are listed in Table 4 and visualized in Figure 4 (see the associated website for more information).

### 2.6. Multiple Sequence Alignments

A profile-based sequence alignment of the core proteins, the barrel structure, was used to generate an MSA; see Table 3 for the templates that were used to generate the initial profile. Based on the alignment results shown in Table 4 and Text S2, the core structure was defined to be the transmembrane β-barrel domain. Those classes with multiple structures were used to update the profile before including the sequences for the eight subclasses of the non-specific porin and specific diffusion channel protein family (see the Methods Section and Table S2). The number of sequences used in the MSAs of the eight different subclasses (listed in Table 5) ranged from 50 to 1384, with an average of approximately 490 sequences.

### 2.7. Entropy-Variability Analysis

**Figure 5:**
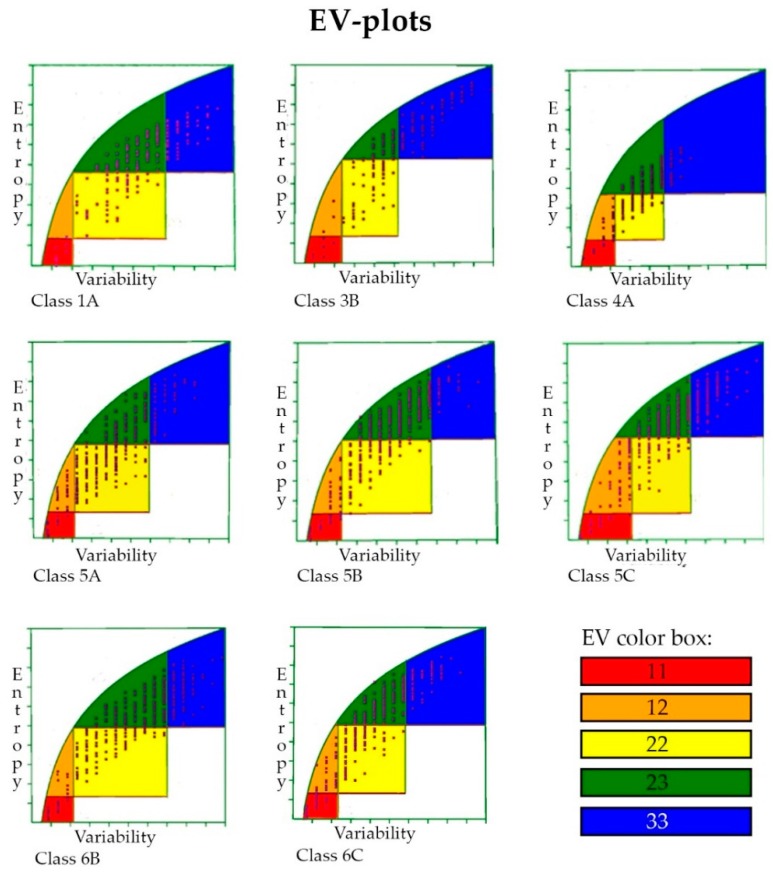
Entropy-variability (EV) plots of the eight subclasses. Red is for those residues likely to be in the main active site; orange is the main active site; green is the regulatory site; yellow communicates; blue is those with unknown function (see the Methods Section for more details).

Entropy-variability analysis (EVA) was then used to obtain a better understanding of these alignments (see the Discussion for more information regarding EVA). Figure 5 shows the entropy-variability (EV) plots generated for the eight different subclasses. These EV plots show that the distribution of points over the plots varied from each subclass, but were scattered throughout the plot, thereby ensuring that there were not too many or too few sequence variations in the alignments. The plots also show that the number of conserved residues varied from each subclass.

**Figure 6:**
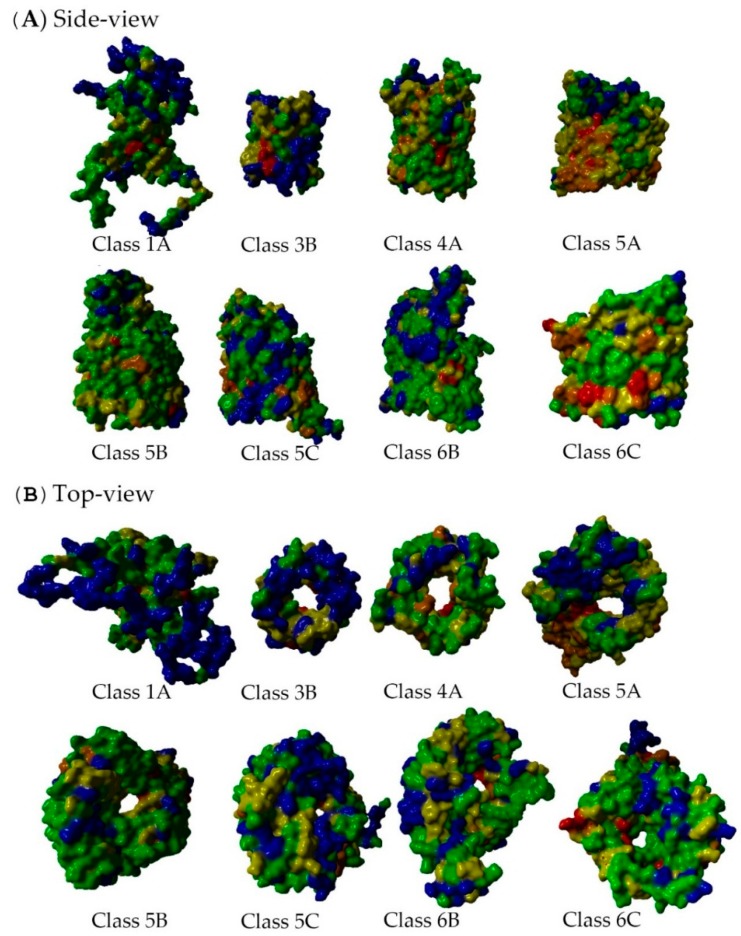
Entropy-variability (EV) results of the eight subclass from (**A**) side-view and (**B**) top-view. The structures are visualized as molecular surfaces and are colored according to residue conservation as described in the Methods Section. This figure illustrates differences in barrel size, pore size and loop variability. All structures are visualized in the monomeric form. These results can be downloaded and viewed in 3D from the associated websites (YASARA (Yet Another Scientific Artificial Reality Application) scene files; see the Supplementary Materials).

**Figure 7:**
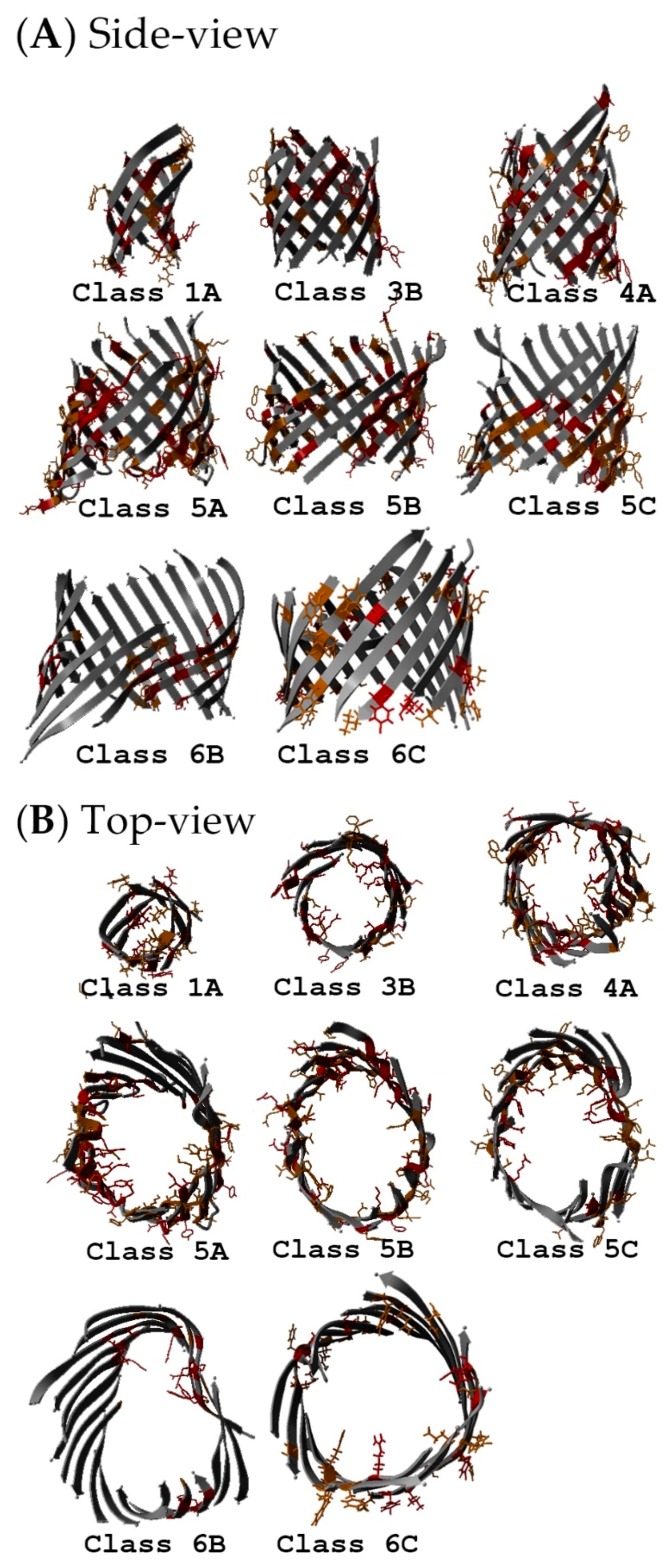
Conserved non-specific porin and specific diffusion channel residues. (**A**) Side view and (**B**) top view of conserved residues (Boxes 11, 12, and 22). Only the monomeric state is shown for all subclasses. Loops and turns are deleted to visualize the protein core. These results can be downloaded and viewed in 3D from the associated websites (YASARA scene files; see the Supplementary Materials).

**Figure 8:**
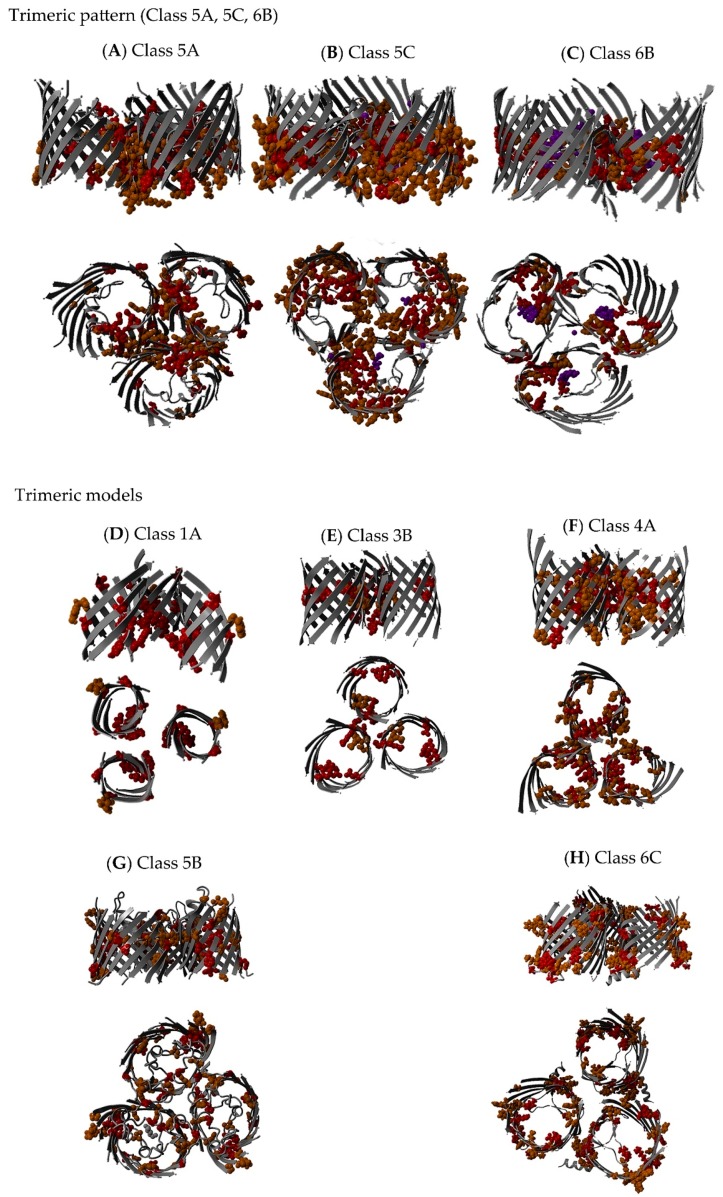
Non-specific porin and specific diffusion channel conservation. This figure illustrates differences in barrel size, pore size and loop variability. All structures are visualized in the trimeric form. Only the structures of Classes 5A (**A**); 5C (**B**); and 6B (**C**) proteins have been solved in an oligomeric conformation (all of them being homotrimeric). Class 1A (**D**); Class 3B (**E**); Class 4A (**F**); Class 5B (**G**) and Class 6C (**H**) are monomeric structures modeled in a trimeric conformation by hand. Same remark as the previous figures.

**Figure 9:**
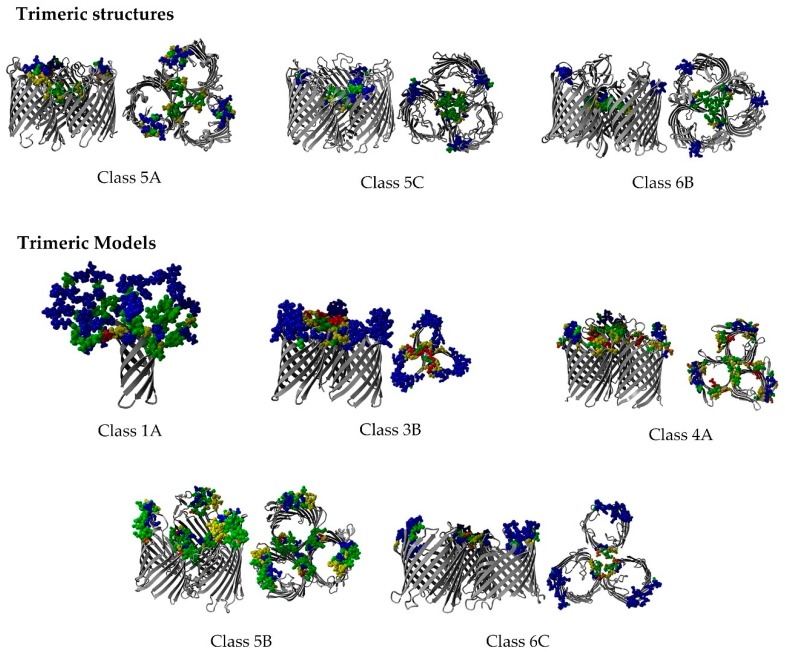
Porin variability. Side and top views displaying the loop variability of all protein subclasses analyzed (including the modeled trimeric interactions shown in Figure 8). All structures are visualized in the predicted trimeric form for Class 1A (2, 3, 5) and eight porins. Class 1A porins are represented in the monomeric state, and only conserved residues are highlighted (see the Discussion for more details). Red, orange and yellow represent conserved residues; green shows partially conserved residues involved in regulation; blue represents highly variable sites with unknown functions. Same as previous figures.

The EVA results were mapped onto structures representing the eight analyses (see Table 3, Figure 6, Figure 7, Figure 8 and Figure 9, and the Methods Section): 5.6% of the residues were contained in Box 11 (highly conserved residues colored red); 7.0% of the residues were contained in Box 12 (very conserved residues colored orange); 26% of the residues were contained in Box 22 (quite conserved residues colored yellow); 40.9% of the residues were contained in Box 23 (moderately conserved residues colored green); and 19.8% of the residues were contained in Box 33 (highly variable residues with unknown function colored blue). The distributions of extracellular loops found in these were 0.5% (Box 11), 3.0% (Box 12), 21.2% (Box 22), 38.9% (Box 23) and 36.4% (Box 33). Of the highly variable (Box 33) extracellular loop residues, 62.9% were located in large loops (longer than 15% of the extracellular loop length for each subclass, except for Class 1A, in which three of the four loops were termed long loops). A constriction loop is essential for diffusion, and it contains a high number of conserved residues (and only 8% of such residues were found in Box 33; see Table S6). These residues are situated inside the barrel and, unlike the other loops, are not in contact with the environment. In summary, the constriction loops contained more conserved residues than the longer extracellular loops (more statistics are found in Tables S3–S6), and a trimeric pattern was observed in the extracellular loops (see next section; 2.8. Non-Specific Porin and Specific Diffusion Channel Multimericity).

### 2.8. Non-Specific Porin and Specific Diffusion Channel Multimericity

Trimeric structures exist for Class 5A (GDP), Class 5C (OprP) and Class 6B (ScrY and maltoporins) (see Figure 8A–C). The trimeric models (Classes 1A, 3B, 4A, 5B and 6C; see Figure 8D–H) were handmade models using the YASARA-WHAT IF twinset that were used for illustrations [38,39]. Trimeric non-specific porins and specific diffusion channels were used as templates when possible (Classes 5C and 6B were used as templates to superpose three monomeric Class 5B and 6C structures). However, the barrels were rotated so that the conserved residues were facing each other, thereby illustrating the most likely orientation with regard to a trimeric interaction. The patterns of protein-protein interaction and multimericity in the different subclasses are illustrated in Figure 8D–H. No trimeric template is currently available for the trimeric motif alignment in Classes 1A, 3B and 4A; thus, the models of possible trimeric interactions among the monomeric non-specific porins and specific diffusion channels were manually generated (Figure 8D–F). The models were generated by manually aligning the most conserved residues (from the EVA) from each monomer to a forced, handmade, homotrimeric model.

The majority of the variable residues were found in the long extracellular loops that are located away from the oligomeric interaction interface. More variable residues were observed in the central loops in the larger barrels than in those in the smaller oligomers (Classes 1A to 4A and 5A). Presumably, the larger barrels do not need these central loops for oligomerization as much as the smaller barrels. In some classes (*i.e.*, Classes 1A to 4A, and 5B), we even observed highly conserved (Boxes 11 and 12) residues in the central loops, indicating the substantial functional importance of these central loops. The central loops were usually shorter and less variable, while the distal loops were longer and more variable. This effect was more pronounced in non-specific porin and specific diffusion channel with smaller barrels (Classes 1A to 4A). We suggest that the short central loop L2 could be important for trimer stabilization, as has been shown for OmpF [40,41].

## 3. Discussion

### 3.1. Data Collection and Secondary Structure Composition

There has been an increase in the number of solved OMP structures; yet, the structures of many OMPs remain unsolved, and very few structures of OMP complexes have been determined [42]. Currently, only 0.1% of the protein structures deposited in the PDB belong to the OMP superfamily. Nearly 40% of the solved OMP structures are protein channels (non-specific porin or specific diffusion channel), indicating their importance in the bacterial protein field.

Despite the variations observed in protein size (the average length varied from 171 to 459 residues in KpOmpA and OccD3, respectively), the secondary structure composition reflected their transmembrane β-barrel motif (see the Introduction). The average 6:1:3 β-strand:periplasmic loop:extracellular loop residue ratio, shown in Table 2, confirmed that non-specific porins and specific diffusion channels are composed of a large number of β-strands that are connected by shorter periplasmic loops and longer extracellular loops.

### 3.2. Non-Specific Porin and Specific Diffusion Channel Phylogenetic Tree

The grouping of porin clades shown in Figure 3 reflects non-specific porin and specific diffusion channel function and size variations, rather than the current ideas about a particular porin′s multimeric state. Non-specific monomeric trimeric porins (NTPs) formed three separate clades; α-Proteobacteria (*Rhodopseudomonas blastica* general diffusion porin (RbGDP) and *Rhodobacter capsulatus* general diffusion porin (RcGDP)); γ-Proteobacteria (*Salmonella* Typhimurium outer membrane protein C (StOmpC), PhoE, *Escherichia coli* outer membrane protein C (EcOmpC), OmpK36 and EcOmpF); and a clade with *Salmonella* Typhimurium outer membrane protein F (StOmpF) (γ-Proteobacteria) and Omp32 (β-Proteobacteria) that clustered with the non-specific smallest monomeric porins (OmpA). Two of the three specific trimeric channels (maltoporins) clustered together, while ScrY clustered with specific monomeric channels. OmpG (a non-specific monomeric rescue porin) clustered with maltoporins, which is not surprising, as it can mimic maltoporin uptake if needed [43]. Specific monomeric and specific trimeric *Pseudomonas* channels (OprB and OprP) clustered in one clade. Although function is the most likely explanation for these clades, size could also have an impact, as the largest porins (Occ channels) form a separate clade from the other eight- to 16-stranded β-barrels. Furthermore, the two OmpA sequences cluster together (eight-stranded β-barrel) as does the KdgM subclass (12-stranded β-barrel).

### 3.3. Non-Specific Porin and Specific Diffusion Channel Classification System

A naming scheme for protein families should be simple, robust and reproducible. It should also give space for future sequences to be added. We believe this has been accomplished for those proteins involved in the diffusion of hydrophilic molecules, which is based on available structure, sequence and literature information. This enabled us to analyze the six classes (with same β-barrel sizes) with eight different subclasses, looking at structure-function relationship. This classification system yields one ontology for those working with porins and specific diffusion channels. New information (new structures, new function studies) might require an updated schema with a more detailed nomenclature than presented in this article. However, this classification system represents the groups of an OMP family that were required for the analyses performed.

### 3.4. Non-Specific Porin and Specific Diffusion Channel Structure Analysis

Non-specific porin and specific diffusion channel structures were used as a template to guide the MSA of all of the collected sequences (see the Methods Section for more details). The most unambiguous alignments tended to be obtained when only the barrels (with all loop residues removed) were aligned. Structure comparisons were possible for those subclasses containing more than one structure. Three of the eight subclasses have only one structure. Some of these alignments (e.g., Class 4A) resulted in fewer sequences than the optimum number required to harvest the full potential of the EV method used to analyze each protein subclasses. However, these alignments did yield relevant information, which will be discussed in light of other studies and published experiments. Table 1 lists the details of the structures found in each subclass (PDB ID, protein names, *etc.*).

Pore size may vary with environmental factors, e.g., pH-sensitivity of OmpF and OmpG channels [44,45]. Some of the structures analyzed have been proven to be in a closed state (e.g., OmpA, OccD1 and D2). Our pore size estimates complement literature findings (a complete list of literature estimates is found on the associated website). However, pore sizes analyzed using the whole protein revealed more variability within each subclass than the core structures (for those subclasses with more than one protein structure). Figure 3 shows that the pore size estimates correlate best with our classification system (based on barrel size, specificity and function).

The OmpF pore illustrates how estimations of the minimum core value can be useful. Experiments indicate that *E. coli* OmpF has a pore size of 10 to 12 Å. This would allow raffinose to pass through the pore [46]. Although this is about twice the structure-based pore size estimation using the entire protein, the minimum OmpF pore core radius is 6.2 Å. Removing the loops actually results in being closer to the estimates derived from laboratory experiments. This supports the theory of flexible loops affecting pore sizes [29,47,48,49,50,51]. The pore size calculations of the core protein are not occluded by any flexible loops. The constriction loop (usually loop L3) and extracellular loops govern the pore and limit pore size. These loops will shrink or increase the pore size in response to the continuously-changing environment. Having a minimum and maximum radius of a pore channel yields a better understanding of what might get through the pore.

### 3.5. Multiple Sequence Alignments

Each multiple sequence alignment (MSA) generated was based on available structure and sequence information (see Table 1, Table 2, Table 3 and Table 4 for more details). The number of solved structures in each subclass varied from one to 15 (see Table 1 for a complete list of PDB IDs), and the number of sequences also varied greatly for each subclass. The generated MSAs were used to construct the EV plot and figures discussed below. Some of the subclasses held a less than optimal number of sequences for the EVA, although they contained enough variability to create sensible plots and figures.

### 3.6. Entropy-Variability Analysis

Each residue position in an aligned EV plot correlates with the residue′s function and structural characteristics. This sequence analysis technique is based on a combination of two commonly-used sequence variability measures. The first is variability, defined as the number of different amino acid types observed at each position. The second is Shannon entropy. Each residue position in the alignment is plotted on the EV plot. Boxes in this plot appear to represent groups of residues that share a common structural or functional characteristic. Conserved amino acids within a subclasses indicate that these residues are functionally important. The analysis is based on the collection of a large number of sequences that was used to filter the variability patterns. A profile alignment is used to identify conserved features in the structures. Finally, a plot is created based on where the residues are placed according to their structure-function characteristics. This plot is divided into five boxes (Boxes 11, 12, 22, 23 and 33); each box contains the residues involved in the same functional category (see the Methods Section for more details) [32].

The most conserved residues that are important for protein functions are found in Box 11. Previous EV studies showed that highly-conserved residues (Box 11) are located in the active sites of proteins (e.g., G-protein-coupled receptors, globins, Ras-like proteins and proteases). Those residues supporting the active site were also quite conserved (Box 12). Box 22 contains the signal transducing residues between the modulator and main functions, while the residues found in Box 23 modulate the main function. The remaining residues that do not have any specific function are found in Box 33 [32,52,53].

According to the literature, the exterior loops of the non-specific porins and specific diffusion channels are continuously changing to avoid detection by the host immune system, phage invasion and as a response to ecological pressure. The exterior loops are the most variable regions in non-specific porins and specific diffusion channels, which reflects the adaptive traits accomplished through mutation or DNA rearrangement [10,54,55]. Both non-specific porins and specific diffusion channels had variable residues that were located mainly in extracellular loops. These proteins have long, highly variable, protruding loops, as well as more conserved, shorter loops that face the extracellular environment, as shown in Figure 8. This could be a mechanism in which the long, variable loops help to evade the host immune systems, while the shorter, conserved loops bind substrates [5,56]. For example, Class 1A is a multifunctional protein targeted by the immune system and is a bacteriophage receptor. These features are mainly conferred by the exterior loops [22]. Class 1A OmpA has probably adapted loop mutations to evade the immune system; e.g., loop L2 mutation may be the difference of an invasive and less invasive *E. coli* strain [57]. The fraction of variable, long loop residues is slightly lower in the specific diffusion channels (30%) than in the non-specific porins (42%) (see Table S3).

The conserved constriction loop residues observed in the EVA (for the larger barrels of Classes 5A–6C) support their importance in determining the substrate specificity. The presence of charged residues in the constriction loop (loop L3) of non-specific porins and specific diffusion channels creates an electrostatic field, which largely determines the permeability and ion selectivity of the pores [47,58,59].

This variability is mainly determined by differences in the number of sequences in the underlying MSA and by the average sequence identity between the sequences in the MSA. Nevertheless, a series of trends was clearly observable, but all classes have both conserved residues facing the core and the lipid membrane. These are important residues for protein function, as they are involved in either substrate or protein–protein interactions. It was not possible to directly discriminate between the monomeric and trimeric structures from these EV analyses, but mapping the residues in Boxes 11, 12 and 22 onto the protein structures revealed that all non-specific porins and specific diffusion channels have important residues pointing away from the core of the barrel. The only function imaginable for these residues is in protein–protein interactions. This observation strongly suggests that neither monomeric non-specific porins, nor specific diffusion channels exist. Figure 6 and Figure 7 summarize the EV results for the barrel- and lipid-facing residues. These figures also show the observed differences in core size between the eight classes analyzed.

### 3.7. Non-Specific Porin and Specific Diffusion Channel Multimericity

The conserved residues facing the barrel core are likely to be important for pore activity. The conserved, lipid-facing residues are likely to be involved in protein-protein interactions, e.g., trimerization. Recent publications with laboratory data have verified these results (see Table 6). Loops involved in trimerization have a high proportion of residues in Boxes 22 and 23 and fewer residues in Box 33. Their interaction pattern is shown with the conserved residues in Figure 6A.

Table 6 describes the eight subclasses used in our analyses (including MSAs and EVAs), and it provides information regarding the presumed multimeric state (monomeric, dimeric or oligomeric protein channels). Classical porins (belonging to Class 5A) have long been described as trimeric porins. Trimeric structures of Class 5C (OprP) and Class 6B (ScrY and maltoporins) porins have also been solved. Several publications have shown that the classic monomeric non-specific porins and specific diffusion channels actually oligomerized in the right conditions (e.g., lower temperatures or less detergents) [44,70,74,88,94,95,96,97]. Thus, unconventional methods might be necessary to detect protein oligomerization of non-specific porins and/or specific diffusion channels. These proteins can form less stable trimers compared to those in Classes 5A, 6 and 7. This is likely due to the environmental changes, such as high temperature and detergents, which has been shown to break protein subunit interactions. The weaker non-specific porin or specific diffusion channel oligomerization does not need to imply a non-essential function. The OM efflux pump TolC can be used as an example of this. One TolC subunit form 1/3 of a β-barrel; hence, a TolC trimer forms one β-barrel structure (a trimer is essential for a functional protein) when not boiled using SDS–PAGE [98].

Only one NMR structure is available from the PDB for Class 1A porins. The structures of the loops in this porin were not determined experimentally, which makes it nearly impossible to construct a trimeric model (and consequently, it is missing from Figure 7). However, we know that the structure contains four long loops ranging from 18 to 25 residues in length. The shortest loop (18 residues) contains many conserved residues, including one in Box 11 (see Table S3). This loop sequence variability analysis corroborates the idea that Class 1A porins are trimeric. Figure 8D–H highlight the possibility that monomeric porins can form homotrimers. This model only shows three monomeric barrels, with the most conserved sites turned toward each other. The most conserved outward-facing residues are aligned to interact with each other to simulate the trimeric pattern observed in the EVA of the trimeric structures. The interaction would be better in a trimeric crystal (like those of the homotrimeric Class 5A structures), where the binding sites are optimized compared to those of the monomeric structures. Monomeric, dimeric and trimeric barrels may show different conformations in monomeric and oligomeric states, e.g., the outer membrane phospholipase A (OMPLA) and OprP structures [5,99].

There is much debate about Class 5B OprB multimericity. Based on the results from two-dimensional gel electrophoresis and liquid chromatography-tandem mass spectrometry analyses, Shrivastava *et al.* [88] concluded that *Pseudomonas* OprB-specific diffusion channels form homotrimers. This conclusion has been debated in the literature, and arguments for a monomeric protein have been made. The results of Shrivastava *et al.* did not take large micelles into account when calculating protein size, which according to van den Berg [23], would result in OprB forming monomers, rather than homotrimers. In addition, the OprB channel lacks the amino-terminal region found in the *Pseudomonas* OprP trimeric structure [23]. Stabilization of trimers by amino-terminal strand exchange has thus far only been identified in OprP trimerization [5,30]. This does not necessarily have to be a common trait only for *Pseudomonas* channels; it could be a common trait for all phosphate-specific channels. However, the motif aligner suggested that the amino-terminal OprB helices take part in protein–protein interactions. These preliminary results would also explain the conserved residues found in this region According to our analyses, Class 5B channels are also likely to be trimeric (see Figure 6G). OprB is similar to trimeric structures (e.g., those of Class 5A), and it has an asymmetrical extracellular structure. Class 5B channels exhibit a conservation pattern similar to that observed in other trimeric structures. This supports the findings by Shrivastava *et al.* [88]. The conserved residues found on the opposite side of the proposed trimeric interaction site reveal protein interactions that are important to this subclass. It is also possible that this is the site of trimerization, and the amino-terminal loops are involved in protein binding in the periplasmic space. Further studies are needed to better understand the protein interactions of non-specific porins and specific diffusion channels.

The periplasmic amino-terminal residues found in the Class 5C OprP channel structure have a stabilizing effect on the trimer [30]. This is likely to be a *Pseudomonas* sp.-specific feature that is not found throughout the Class 5C MSA (see Figure 8B). This indicates that trimerization in Class 5C channels is likely to be caused by other residues in the trimer interface. The conserved trimeric pattern of Class 6B channels is shown in Figure 8C. The conserved residues are involved in binding and loop specificity.

Regarding the possibility of Class 6C oligomerization, our results support the findings of Biswas *et al.* [93], in which polyacrylamide gel electrophoresis of OccD1 (OprD) revealed the presence of oligomers. They concluded that OccD1 might exist as a trimer (although the trimer was less stable than those of other homotrimeric channels). Figure 8H illustrates a homotrimeric channel based on the conformation of another protein structure, although it requires experimental validation (a manually-generated model would probably rotate the monomers into a potentially more stable conformation). Class 6C channels displayed a trimeric pattern in the EVA (see Text S1 for more information). Conserved residues were concentrated on the largest side of the asymmetric barrel facing out toward the lipid membrane. In addition, Class 6C channels had conserved residues facing the pore, thereby providing substrate specificity. High loop variability was observed on the amino-terminal end (facing the periplasm). The loops on the side of the barrel pointing away from the trimer interface may be involved in interactions with host proteins.

We generally found that the patterns observed for channels were highly conserved, indicating a specific protein-protein interaction. Protein-lipid interactions are seldom highly conserved, as there are many amino acids with similar properties [100]. The assembly machinery would require conserved residues for all classes; however, the pattern we observed was not identical between the eight subclasses analyzed. The number of conserved residues and side-chain features varied, indicating that each subclass has its own interaction pattern, which supports the homotrimeric hypothesis.

Oligomeric proteins generate stable non-specific porins and specific diffusion channels that can withstand harsh environments. The oligomerization of OmpF occurs in a step-wise manner. Naveed *et al.* [101] demonstrated that these trimeric porins may also have stable monomeric and dimeric states. An oligomeric analysis of Class 1A OmpA porins showed that dimers and monomers were found in the same population, but the physiological role of the dimers remains unknown [27]. These results suggest that some porins may oligomerize only under certain physiological conditions. Further studies are required to fully understand when non-specific porin and specific diffusion channel oligomerize, but our analyses indicate that they all trimerize at some point.

## 4. Materials and Methods

### 4.1. Non-Specific Porin and Specific Diffusion Channel Multimericity

This is an *in silico* study based on the extraction of bacterial β-barrel OMP structures from the Protein Data Bank (PDB). The extraction was performed on February 2014. From this collection, non-specific porins and specific diffusion channels were manually selected and divided into four groups (see the numbered list in the Introduction). Two NMP structures are available from the PDB: KpOmpA (2K0L holds 20 NMR structures of this *K. pneumoniae* protein) and EcOmpA (1QJP holds an X-ray structure of this *E. coli* protein). The latter structure lacks 34 extracellular loop residues. Although both structures were used in the analyses, only KpOmpA (Model 2) was used in the structure superposition that guided the MSAs.

Structure and literature databases were searched for relevant information. We compared the current classification system with structural and functional information before a new schema was constructed. Search criteria included porins (specific and non-specific channels) and OMPs. All non-bacterial hits were filtered out. Literature searches included searching for each porin′s name, structure and/or function to retrieve more information for each class. Relevant information discussing the function of porin structures was included.

### 4.2. Structure Analysis

All structure analyses were performed using the YASARA-WHAT IF twinset [38,39]. Water molecules, co-factors, substrates and lipids were removed, and YASARA′s “clean” function was used on all structures. For trimeric molecules, only the first structure (labeled “A”) in the PDB file was used for analyses that were not explicitly related to multimeric interactions. This molecule was renumbered before starting the analyses. Structure alignments were performed on the monomeric structures, using the MUSTANG (A multiple structural alignment algorithm) pairwise motif aligner [102] as implemented in YASARA. These structure analyses were compared to the pairwise sequence identity gathered using EBI Clustal Ω [103,104] (see Tables S1–S5 for an overview of the core and whole protein structure analyses and Table S2raw for the complete list of pairwise identities used to calculate the average values found in Table 2).

Pore sizes were estimated using the HOLE software (Version 2.2.004, SmartSci, Cambridge, UK) [37]. The minimum pore size was calculated for both the whole protein and core protein (where all loops and turns were removed) using default settings. Our associated web page also contains pore size estimation of poly-alanine mutated β-barrel structures (where loops and turns were removed).

### 4.3. Multiple Sequence Analysis

**Figure 10:**
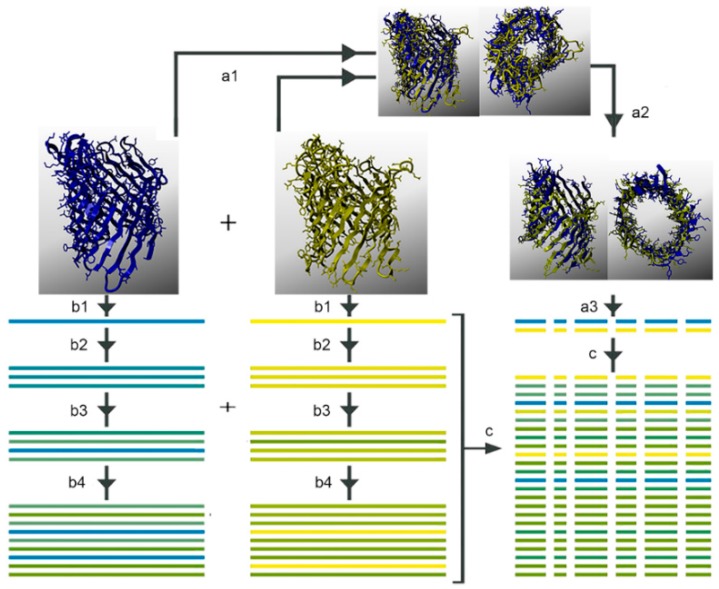
The sequence analysis workflow [102] (copied with permission from Bio-Prodict) shows the process of structure superposition guided by a profile alignment of the sequences. Step a1 shows the superposition of two structures (Omp32, colored blue, and PhoE in yellow; PDB IDs: 2FGQ and 1PHO, respectively); Step a2 shows the core alignment of the barrel structures in which loops have been removed; Step a3 is core alignment of similar structure; Step b is a sequence alignment against the nearest structure. All sequences and structures from one subclass are aligned together in step c. Both structures used in this MSA (multiple sequence alignment) belong to the general diffusion protein subclass. The WHAT IF module of the YASARA/WHAT IF twinset was used for these analyses [39].

The workflow of the MSA is described by Kuipers *et al.* [105] and depicted in Figure 10. The first step in this workflow is data collection. This was accomplished through DELTA-BLAST (Domain Enhanced Lookup Time Accelerated BLAST algorithm) searches to collect the top 1000 sequences with an *e*-value <0.1 [103,104]. OMPdb [35] sequences were also included in the analyses. The second step is an iterative profile alignment step using the WHAT IF module in the YASARA/WHAT IF twinset [32,38,39,106] (see Appendix A for more information). The collected sequences were aligned to the profile generated for each subclass.

### 4.4. Sequence Filtering

Sequence identities >90% were filtered out in WHAT IF using the DMATCH (asks for sequence identity cutoff value) option. This is an iterative algorithm that uses pairwise sequence comparisons (after sequence alignments) to identify and remove sequences with >90% sequence identities. Sequences with low identities (<20% to 30%) were also filtered out. This step removes little information, but throws out a lot of noise. It ensures an unbiased alignment and aids the entropy-variability calculations.

### 4.5. Entropy-Variability Analysis

EVAs were used to develop an evolutionary model [32]. The entropy and variability terms in the EVAs provide two different descriptions of the variability patterns for individual residue positions in the MSAs. The Shannon entropy (*E*_i_) at each position is defined as Σ*P* log(*P*), where *P* represents the frequency of occurrence of a given amino acid in the MSA at position *i*. The variability *V*_i_ is the number of amino acid types observed (for more than 0.5%) at position *i*.

By plotting the entropy, *E*_i_, *versus* the variability, *V*_i_, for all residue positions *i* in the MSA, the EV plot can be divided into five sectors, each of which tends to contain residues mainly involved in one broad functional category [32]. The low entropy and low variability box (Box 11), colored red, contains residues in the main active site; the intermediate entropy and low variability box (Box 12), colored orange, contains residues that support the structure of the main active site (often situated next to the red residues); the intermediate entropy and intermediate variability box (Box 22), colored yellow, contains residues involved in communication between the main active site and regulatory sites (further away from the core than the red and orange residues); the high entropy and intermediate variability box (Box 23), colored green, contains residues involved in the regulation of protein activity (modulators may be located on the surface or in the core of proteins); and the high entropy and high variability box (Box 33), colored blue, contains residues for which no function is known. EVA is based on well-established experimental methods from multiple, large protein families: globin chains, G-protein-coupled receptors, Ras-like proteins and serine-proteases [33,53]. Signal transduction residues have also been identified in other protein families, including the nuclear receptor family [52]. Figure 6 and Figure 7 can be downloaded as YASARA scene files from the associated website. This allows users to see in 3D what we study. See the Supplementary Materials for more information.

### 4.6. Phylogenetic Analysis

An MSA of non-specific porins and specific diffusion channels with solved structures was generated (see Step b in the workflow found in Figure 10). The OccK3 channel sequence was used to guide the MSA. A maximum-likelihood phylogenetic tree was built with PhyML Version 3.0 (French National Institute of Bioinformatics, Montpellier, France) [107] using 1000 bootstrap replicates for branch support tests [108]. The Whelan and Goldman (WAG) model of amino acid substitution, with nine γ-distributed rate categories (WAG + G), and nearest-neighbor interchange methods of a tree topology search were used [107,109]. Molecular Evolutionary Genetics Analysis software Version 6.0 (MEGA 6.0, Biodesign Institute, Tempe, AZ, USA) [110] selected WAG + G to be the best-fit model [109]. The results were combined by “majority rule” consensus using Consense [111]. The final phylogenetic tree was visualized as unrooted radial trees in FigTree v 1.4.2 (Prof. Rambaut, Molecular Evolution, Phylogenetics and Epidemiology, University of Edinburgh, Edinburgh, Scotland).

## 5. Conclusions

EVA results were correlated to known structural and functional aspects of non-specific porins and specific diffusion channels, e.g., structural integrity, multimericity, specificity or biological niche adaptations. All non-specific porins have highly variable loops facing the environment, which strengthens the hypothesis that they have been subject to positive selection. Loop variability among many of the porins (especially Class 5A and Class 1A porins) supports the high mutation rate needed for events, such as host evasion. Loop variability is a pathogenic mechanism. However, conserved protein binding sites can also reveal cooperative interactions. Cooperative porin binding to bacterial pili and the human CR3 receptor has been observed during host invasion [112]. Our results indicate that all non-specific porins and specific diffusion channels are multimers, and the residues involved in protein-protein interactions can be determined in all eight classes analyzed. Trimeric interaction patterns were identified in non-specific porins and specific diffusion channels that are currently classed as monomeric, with conserved residues found at the interface of the trimeric interactions. EVA showed that conserved residues are concentrated in the trimeric interface facing inward and outward from the barrel. The loops of sugar- and phosphate-specific channels were more highly conserved than those of OmpA and other non-specific porins. This indicates that loop specificity might be needed in some porins (or specific diffusion channels) to “capture” the right molecules in the extracellular milieu. Not enough data are available for an extensive study of non-porin OMPs. Our preliminary work on OMPLA indicates that this protein is dimeric, as commonly stated in the literature [99,113,114].

Most classification systems that exist today (including CATH [33], SCOPe [36], TCDB [34] and OMPdb [35] classifications) rely on a division between monomeric and trimeric non-specific porins and specific diffusion channels. We suggest that it is time to discuss a new classification scheme that discriminates between structure and function, rather than relying upon a classification system based on multimeric states. The three trimeric non-specific porin and specific diffusion channel families that exist today are the sugar-specific channels, the phosphate-specific channels and the GDPs (porins). Evidence that many of these proteins exist in multimeric states is emerging in the literature (see Table 6 for references). Our data support these findings, which suggest that most non-specific porins and specific diffusion channels are multimeric and are likely to form homotrimeric interactions.

## Figures and Tables

**Table 1:** Protein structure information of bacterial porins and specific diffusion channels. This table lists protein names and biological and structural information of the structures used in our analyses. The size column lists the number of β-strands.

Groups	Name	Bacteriae	Size	PDB ID	Resolution (Å)
Monomeric proteins					
NMP	EcOmpA	*Escherichia coli*	8	1QJP	1.7
NMP	KpOmpA	*Klebsiella pneumonia*	8	2K0L	–
NMP	OmpG	*Escherichia coli*	14	2IWV	2.3
SMDC	NanC	*Escherichia coli*	12	2WJR	1.8
SMDC	KdgM	*Dickeya dadantii*	12	4FQE	1.9
SMDC	OprB	*Pseudomonas putida*	16	4GEY	2.7
SMDC	OccD	*Pseudomonas putida/Pseudomonas fluorescens*	18	3SYS, 3SZD, 3SZV, 3T0S, 3T20, 3T24, 4FRT, 4FRX, 4FT6, 4FSO, 4FSP, 3JTY, 3SY7, 3SY9, 3SYB	Average: 2.4 (1.5–3.2)
Trimeric proteins					
NTP	EcOmpC	*Escherichia coli*	16	2J1N	2.0
NTP	StOmpC	*Salmonella* Typhimurium	16	3UPG	3.2
NTP	OmpK36	*Klebsiella pneumonia*	16	1OSM	3.2
NTP	StOmpF	*Salmonella* Typhimurium	16	3NSG	2.8
NTP	EcOmpF	*Escherichia coli*	16	4GCS	1.9
NTP	PhoE	*Escherichia coli*	16	1PHO	3.0
NTP	RcGDP	*Rhodobacter capsulatus*	16	2POR	1.8
NTP	RbGDP	*Rhodopseudomonas blastica*	16	1PRN	2.0
NTP	Omp32	*Delftia acidovorans*	16	2FGQ	1.5
STDC	EcMaltoporin	*Escherichia coli*	18	1AF6	2.4
STDC	StMaltoporin	*Salmonella* Typhimurium	18	2MPR	2.4
STDC	ScrY	*Salmonella* Typhimurium	18	1OH2	2.4
STDC	OprP	*Pseudomonas aeruginosa*	16	2O4V	1.9

EcMaltoporin (*Escherichia coli* maltoporin); EcOmpA (*E. coli* outer membrane protein A); EcOmpC (*E. coli* outer membrane protein C); EcOmpF (*E. coli* outer membrane protein F); KdgM (oligogalacturonate-specific channel); KpOmpA (*K. pneumonia* outer membrane protein A); NanC (*N*-acetylneuraminic acid-inducible outer-membrane channel); NMP (non-specific monomeric porins); NTP (non-specific trimeric porin); OccD (outer membrane carboxylate channel); Omp32 (outer membrane protein 32); OmpG (outer membrane protein G); OmpK36 (outer membrane porins of *K. pneumoniae*); OprB (outer membrane porin B); OprP (outer membrane porin P); PDB ID (Protein Data Bank Identifier); PhoE (phosphoporin); RbGDP (*R. blastica* general diffusion porin); RcGDP (*R. capsulatus* general diffusion porin); ScrY (Sugar specific porin); SMDC (specific monomeric diffusion channel); STDC (specific trimeric diffusion channel); StMaltoporin (*S.* Typhium maltoporin); StOmpC (*S.* Typhimurium outer membrane protein C); StOmpF (*S.* Typhimurium outer membrane protein F); Å (Angstrom).

**Table 2:** Secondary structure composition. Secondary structure composition of the average non-specific porin and specific diffusion channel composition, which lists the average length (number of amino acids), percentages of amino acids located in β-strands, periplasmic loops and extracellular loops.

Porin Group	Average Length	Barrel (%)	Periplasmic Loop (%)	Extracellular Loop (%)
NMP	219	57	12	30
SMDC	385	56	14	28
NTP	325	57	11	30
STDC	417	54	11	34

NMP (non-specific monomeric porins); NTP (non-specific trimeric porin); SMDC (specific monomeric diffusion channel); STDC (specific trimeric diffusion channel).

**Table 3:** The non-specific porin and specific diffusion channel family distributed among six classes and further divided into eight subclasses. Corresponding OMPdb [35] classifications are found in Table S1. The empty classes (and subclasses) are reserved for future structures. “Size” refers to the number of strands.

Class		Subclass		Protein Structures	
Number	Size	Number	Name	Protein Name	PDB ID Template (Other)
1	8	1A	Non-specific, petite porin	OmpA	2K0L (1QJP)
2	10	2A	Non-specific, mini porin	–	–
3	12	3A	Non-specific, small porin	–	–
		3B	Oligogalacturonate-specific, small channel	KdgM and NanC	4FQE (2WJR)
4	14	4A	Non-specific, intermediate porin	OmpG	2IWV
5	16	5A	Non-specific, medium porin	OmpC, OmpK36, OmpF, PhoE, Omp32, RcGDP and RbGDP	2J1N (1PRN, 3UPG, 1OSM, 3NSG, 4GCS, 2POR, 2FGQ, and 1PHO)
		5B	Sugar-specific, medium channel	OprB	4GEY
		5C	Phosphate-specific, medium channel	OprP	2O4V
6	18	6A	Non-specific, large porin	–	–
		6B	Sugar-specific, large channel	Maltoporin and ScrY	2MPR (1AF6 and 1OH2)
		6C	Carboxyl-specific, large channel	Occ Channels	3SZV (3SYS, 3SZD, 3T0S, 3T20, 3T24, 4FRT, 4FRX, 4FT6, 4FSO, 4FSP. 3JTY, 3SY7, 3SY9, and 3SYB)

EcMaltoporin (*Escherichia coli* maltoporin); EcOmpA (*E. coli* outer membrane protein A); EcOmpC (*E. coli* outer membrane protein C); EcOmpF (*E. coli* outer membrane protein F); KdgM (oligogalacturonate-specific channel); KpOmpA (*K. pneumonia* outer membrane protein A); NanC (N-acetylneuraminic acid-inducible outer-membrane channel); OccD (outer membrane carboxylate channel); Omp32 (outer membrane protein 32); OmpG (outer membrane protein G); OmpK36 (outer membrane porins of *K. pneumoniae*); OprB (outer membrane porin B); OprP (outer membrane porin P); PDB ID (Protein Data Bank Identifier); PhoE (phosphoporin); RbGDP (*R. blastica* general diffusion porin); RcGDP (*R. capsulatus* general diffusion porin); ScrY (Sugar specific porin); StMaltoporin (*S.* Typhium maltoporin); StOmpC (*S.* Typhimurium outer membrane protein C); StOmpF (*S.* Typhimurium outer membrane protein F).

**Table 4:** Structure alignment statistics of non-specific porins and specific diffusion channels. Core structures are analyzed after the loops were removed (so only the barrel was used). The percentages of aligned residues are averages for each pair-wise alignment possible in the subclass. Empty cells represent classes with only one structure. Minimum pore radii are determined using HOLE [37] for the whole monomeric protein and its β-barrel core, respectively.

Subclasses		Resolution	% Residues Superposed	% Sequence Identity		Pore Size	
Number	Name	RMSD (Å)	Mustang	Mustang	Clustal Ω	Core	Whole
1A	Non-specific, petite porin	1.6	98.7	96.1	93.6	~ 0	0.1
3B	Oligogalacturonate-specific, small channel	1.4	95.0	25.6	28.1	2.9	2.9
4A	Non-specific, intermediate porin	–	–	–	–	3.7	4.0
5A	Non-specific, medium porin	1.4	90.7	40.2	41.3	2.8	6.9
5B	Sugar-specific, medium channel	–	–	–	–	2.2	7.0
5C	Phosphate-specific, medium channel	–	–	–	–	1.6	6.4
6B	Sugar-specific, large channel	0.9	95.6	50.3	49.5	2.1	6.9
6C	Carboxyl-specific, large channel	0.9	96.2	46.4	45.7	1.8	7.8
Average		1.2	95.2	52.0	51.6	2.4	7.5

**Table 5:** Number of sequences used in MSA (multiple sequence alignment analyses). The number of sequences used in the final MSA that was generated for each class is listed together with the class number and name. The lacking classes (and subclasses) are reserved for future structures and highlighted with a question mark.

Subclass Number	Subclass Name	Number of Sequences Used in the MSA
1A	Non-specific, petite porin	389
3B	Oligogalacturonate-specific, small channel	246
4A	Non-specific, intermediate porin	50
5A	Non-specific, medium porin	725
5B	Sugar-specific, medium channel	319
5C	Phosphate-specific, medium channel	180
6B	Sugar-specific, large channel	663
6C	Carboxyl-specific, large channel	1394

**Table 6:** Non-specific porin and specific diffusion channel oligomerization state. This table lists articles discussing the multimeric state of non-specific porins and specific diffusion channels, the protein name and information regarding the presumed multimeric state (monomeric, dimeric or oligomerization).

Class Number	Class Name	Function	Monomer	Dimer	Oligomer
1A	Non-specific, petite porin	Abundant multifunctional porin; host evasion [22]	EcOmpA [3,41,59,60,61,62,63,64,65], KpOmpA [56]	EcOmpA [26,27,66,67]	FopA [68], Pgm6/7 [69], EcOmpA [70]
3B	Oligogalacturonate-specific, small channel	Oligogalacturonate-specific channel [71]	KdgM [71,72,73], NanC [24]	NanC [74]	–
4A	Non-specific, intermediate porin	pH-dependent rare rescue porin [75]	EcOmpG [44,75,76,77,78]	EcOmpG [44]	–
5A	Non-specific, medium porin	Classical porins or general diffusion porin (GDP) [11,79]	–	–	GDP [3,4,16,80,81,82,83,84,85,86]
5B	Sugar-specific, medium channel	Oligosaccharide specific channel [23]	OprB [23,87].	–	OprB [88]
5C	Phosphate-specific, medium channel	Phosphate specific channel [89]	–	–	OprP [30,90], OprO/P heterotrimer [91]
6B	Sugar-specific, large channel	Sugar specific channel [31]	–	–	EcMaltoporin [31], StMaltoporin [92], ScrY [12]
6C	Carboxyl-specific, large channel	Small water-soluble specific channel (Occ channels) [29]	Occ [28,29], OccD1 (OprD) [93].	–	OccK1 (OpdK) [94], AbOprD [95]

AbOprB (*Acinetobacter baumannii* outer membrane porin B); EcMaltoporin (*Escherichia coli* maltoporin); EcOmpA (*E. coli* outer membrane protein A); EcOmpC (*E. coli* outer membrane protein C); EcOmpF (*E. coli* outer membrane protein F); KdgM (oligogalacturonate-specific channel); KpOmpA (*K. pneumonia* outer membrane protein A); NanC (*N*-acetylneuraminic acid-inducible outer-membrane channel); OccD (outer membrane carboxylate channel); Omp32 (outer membrane protein 32); OmpG (outer membrane protein G); OmpK36 (outer membrane porins of *K. pneumoniae*); OprB (outer membrane porin B); OprP (outer membrane porin P); PDB ID (Protein Data Bank Identifier); PhoE (phosphoporin); RbGDP (*R. blastica* general diffusion porin); RcGDP (*R. capsulatus* general diffusion porin); ScrY (Sugar specific porin); StMaltoporin (*S.* Typhium maltoporin); StOmpC (*S.* Typhimurium outer membrane protein C); StOmpF (*S.* Typhimurium outer membrane protein F).

**Figure A1:**
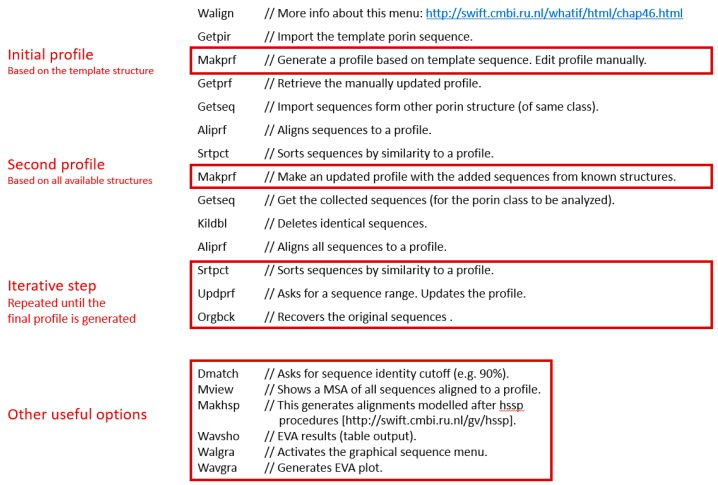
This shows the commands used to generate a profile in WHAT IF. This method was used to generate the multiple sequence alignment (MSA) and to run the entropy-variability analysis (EVA) discussed in the article.

## Supplementary Materials

Supplementary materials can be found at http://www.mdpi.com/1422-0067/17/4/599/s1. Our associated website (www.hildevollan.no/publications/porins) will be updated with relevant analyses, an abstract video, and 3D structures (that can be viewed by downloading YASARA View free from www.yasara.org).

## Abbreviations

AbOprD	*Acinetobacter baumannii* OprD
EcMaltoporin	*E. coli* maltoporin
EcOmp	*E. coli* outer membrane protein
EV	entropy-variability
EVA	entropy-variability analysis
GDP	general diffusion porin
KpOmpA	*K. pneumoniae* outer membrane protein A
MSA	multiple sequence alignment
NanC	*N*-acetylneuraminic acid-inducible outer-membrane channel
NMP	non-specific monomeric porin
NTP	non-specific trimeric porin
Occ	outer membrane carboxylate channel
OMP	outer membrane protein
OmpK36	outer membrane porin of *K. pneumoniae*
OMPLA	outer membrane phospholipase A
Opr	outer membrane porin
PhoE	Phosphoporin
PDB	Protein Data Bank
RbGDP	*R. blastica* general diffusion porin
RcGDP	*R. capsulatus* general diffusion porin
RMSD	root-mean-square deviation
SMDC	specific monomeric diffusion channel
*Salmonella* Typhimurium	*Salmonella enterica* serovar Typhimurium
StMaltoporin	*Salmonella* Typhimurium maltoporin
StOmp	*Salmonella* Typhimurium outer membrane protein
STDC	specific trimeric diffusion channel

## Appendix A

### Iterative Profile Alignment Method

The commands used to perform an iterative profile alignment in WHAT IF is shown in Figure A1.
