# Supplementary material for: In Silico Structure and Sequence Analysis of Bacterial Porins and Specific Diffusion Channels for Hydrophilic Molecules: Conservation, Multimericity and Multifunctionality
Source: Int J Mol Sci. 2016 Apr 21;17(4):599. doi: 10.3390/ijms17040599 (PMC4849052; doi:10.3390/ijms17040599)
Supplement: Supplementary file 1 [file ijms-17-00599-s001.zip › ijms-120323-Supplementary Materials/ijms-120323-Supplemental-Text S2.pdf]

MSA: Multiple Sequence Alignments

(mview format)

Class 1A

```
CONSENSUS       IAVALAASVAQAAPQDNTWYAGAKLGWSQFHDGGFQGNGKDNNNGPTDRNSLGAGAFGGYQFNPYLAFELGYDWLGRMKYKGDVNNGAFKAQGVQLSLKLSYPVTDDLDVYGRLGGAVWRADSKGNYAAGGARLKDHDTGVSPLAALGVEYAITPNLATRLEYQWVNNIGDANTVGYRPDNGSLSLGVSYRFGQGEAAPVVAPAPAPAPV
150954392.pir   VALAGFATVAQAAPKDNTWYAGGKLGWSQYHDTGFYGNGFQNNNGPTRNDQLGAGAFGGYQVNPYLGFEMGYDWLGRMAYKGSVDNGAFKAQGVQLTAKLGYPITDDLDIYTRLGGMVWRADSKGNYASTGVSRSEHDTGVSPVFAGGVEWAVTRDIATRLEYQWVNNIGDAGTVGTRPDNGMLSLGVSYRFGQEDAAPVVAPAPAPAPE
639191197.pir   VALAGFATVAQAAPKDDTWYTGAKLGWSQYHDTGFYGNGYQNgnGPTHESQLGAGAFLGYQANQYLGFEMGYDWLGRMPYKGSVNNGAFKAQGVQLAAKLSYPIMDDLDIYTRLGGMVWRADSSAHYGATDQRLSNHDTGVSPLAAVGVEYALTRDWATRLDYQWVNNIGDAGTVGARPDNGMLSVGVSYRFGQEVAAPVVAPAPAPAPV
320577122.pir   VALAGFATVAQAAPKDDTWYTGGKLGWSQYHDTGFYGNGFDnaNGPTRDDQLGAGAFLGYQANPYLGFELGYDWLGRMAYKGSVNNGAFKAQGISLAAKLSYPLTDDLDIYTRLGGMVWRADSKGAYDNGNarVSDHDTGVSPLAAVGLEYAWTKNWATRLDYQWVNNIGDAGTVGTRPDNSMLSVGVSYRFGQDEVAP.PAPAPAPAPV
377818202.pir   VALAGFATVAQAAPKDNTWYTGAKLGWSQYHDTGYYGNGYQNNDGPTHESQLGAGAFAGYQANPYLGFELGYDWLGRMPNKGNVTNGAFKAQGIQLAAKLSYPIADDLDVYTRLGGMVWRADSTQQNVATGNRISDHDTGVSPLAAVGVEYALTKNWATRLDYQWVNNIGDAQTVGARPDNGMLSLGVSYRFGQDdaAPAPAPAPAPV
445211870.pir   VALAGFATVAQAAPKDNTWYTGAKLGWSQYHDTGFYGNGYQNgnGPTHKDQLGAGAFLGYQANQYLGFELGYDWLGRMPYKGSVNNGAFKAQGVQLAAKLSYPIADDLDIYTRLGGMVWRADSKANYGRTGQRLSDHDTGVSPLAAVGVEYALTKNWATRLDYQFVSNIGDAGTVGARPDNTMLSLGVSYRFGQDDvaPAPAPAPAPVVE
515917846.pir   VALAGFATVAQAAPKDDTWYTGAKLGWSQYHDTGNYGNGYLNNNGPTHESQLGAGAFLGYQANPYLGFELGYDWLGRMPYKGTVdnGAFKAQGVQLTAKLSYPITDDLDVYTRLGGMVWRADGTQNNGTYG.RFKDHDSGVSPLAAVGVEYALTKNWATRLDYQWVNNIGDAQTVGARPDNATLSVGLSYRFGQDeaAPVVAPAPAPAPV
544019843.pir   VALAGFATVAQAAPKDDTWYTGAKLGWSQYHDTGYYGNGYENNNGPTHESQLGAGAFLGYQANPYLGFELGYDWLGRMPNKGSVTNGAFKAQGVQLAAKLSYPITDDLDVYTRLGGMVWRADSTQTNPTTG.RISDHDTGVSPLAAVGVEYALTKNWATRLDYQWVNNIGDAGTVGARPDNSMLSVGVSYRFGQDEAavVAAPTPAPAPV
668965564.pir   VALAGFATVAQAAPKDNTWYAGGKLGWSQFHDTGFYGNGYQNNNGSTHNSQLGAGAFGGYQVNPYVGLEMGYDWLGRMAYTGSVDNGAFKAQGVQLAAKLSYPITDELDIYTRLGGMVWRADSKGNYDNGTTRVSNHDTGVSPLAAVGLEYAITRDIATRLDYQWVSNIGDAQTVGTRPDNGMLSLGVSYRFGQYDAAPVVAPAPAPAPE
321167474.pir   VALAGFATVAQAAPKDNTWYTGGKLGWSQYHDTGFANAYNGIGNGPTHESQLGAGAFLGYQANQYLGFEMGYDWLGRMPYKGSVNNGAFKAQGVQLAAKLSYPLTDDLDLYTRLGGMVWRADAKGNFNNgnGRVSDHDTGVSPLAAVGVEYAVTKNWATRLDYQWVNNIGDAATVGARPDNAMLSVGVSYRFGQDEAapVVAPAPAPAPV
507923870.pir   VALAGFTTLAQAAPKDNTWYTGAKLGWSQYHDTGYYGNGYVNNDGPTHQNQLGAGAFGGYQANQYLGFELGYDWLGRMPYKGNNVNGAFKAQGVQLAAKLSYPIADDLDVYTRLGGMVWRADSTQNN..NGTRIGDHDTGVSPLAAIGAEYALTKNWATRLDYQWVNNIGDANTVGTRPDNGMLSLGVSYRFGQDdaAPVPAPAPAPAPV
658468934.pir   VALAGFATVAQAAPKDNTWYTGAKLGWSQYHDTGFYGNNYVNNDGPTHDNQLGAGAFVGYQANQYLGFEGGYDWLGRMPYKGNNVNGAFKAQGIQLAAKLSYPITDDLDVYTRLGGMVWRADSKENRA..GARLSNTDTGVSPLAAVGVEYALTKNWATRLDYQWVNNIGDAETVGARPDNSMLSLGVSYRFGQDdaAPAPAPAPAPAPV
386924607.pir   VALAGFATVAQAAPKDNTWYAGGKLGWSQYHDTGFYGNGYQNNNGATHESQLGAGAFGGYQVNPYLAFEGGYDWLGRMPYKGDQVNGAFKSQGVQLTAKLSYPIYDDLDIYTRLGGMVWRADAKNNVV.GGDRRSNHDTGVSPVFAGGVEYAWTPSIATRLEYQWINNIGDAGTVGTRPDNGMLSVGVSYRFGQDEAAPVVAPAPAPAPQ
253781671.pir   VAVAAFTTAAQAAPKDNTWYTGGKLGWSQYHDVNFYGNGYTngNGPTHKNQLGAGAYLGYQTNPYLGFEIGYDWLGRMPYKGSVNNGAFKTHGFQLATKLSYPITDDLDIYTRLGGMVWRADSKATYGATNTHRKDHDTGVSPLAAIGVEYALTKNWATRLDYQWVNNIGDAGTVGARPDNGLLSVGVSYRFGQDeaAPVVAPAPVVAPT
562747541.pir   VALAGFATVAQAAPKDDTWYTGGKLGWSQYHDTGYYGNGYQNNDGPTHESQLGAGAFLGYQANPYLGFELGYDWLGRMPNKGNETNGAFKAQGVQLAAKLSYPIADDLDVYTRLGGMVWRADATQTNPTYG.RISNHDTGVSPLAAVGVEYALTKNWATRLDYQWVNNIGDAGTVGTRPDNSMLSVGVSYRFGQDDVAPVVAPAPAPAPV
KpOmpA.pir      ...MKAIFVLNAAPKDNTWYAGGKLGWSQYHDTGFYGNGFQNNNGPTRNDQLGAGAFGGYQVNPYLGFEMGYDWLGRMAYKGSVDNGAFKAQGVQLTAKLGYPITDDLDIYTRLGGMVWRADSKGNYASTGVSRSEHDTGVSPVFAGGVEWAVTRDIATRLEYQWVNNIGDAGTVGTRPDNGMLSLGVSYRFGQEDAAPVVAPAPAPAPE
653403653.pir   VALAGFATVAQAAPMDNTWYAGGKLGWSQYHDTGFYGNGYSANNGPTHESQLGAGAYGGYQVNPYVGFEMGYDWLGRMPYKGSVTNGAFKAQGVQLTAKLSYPVMDDLDVYTRLGGMVWRADSKEN.....TGLKDHDTGVSPVFAGGVEYAFTPEIATRLEYQWVNNIGDAGTVGARPDNGMLSVGVSYRFGQSEPAPVVAPAPAPAPA
535653471.pir   VALAGFATVAQAAPKDNTWYTGAKLGWSQYHDTG.....FINNNGPTHENQLGAGAFGGYQVNPYVGFEMGYDWLGRMPYKGDNINGAYKAQGVQLTAKLGYPITDDLDVYTRLGGMVWRADTKSNVP.GGVSTKDHDTGVSPVFAGGVEYAITPEIATRLEYQWTNNIGDAHTIGTRPDNGMLSLGVSYRFGQGEAAPVVAPAPAPAPE
498911745.pir   IAVAAFATAAQAAPKDNTWYTGGKLGWSQYHDVNFYGNGYDtgNGSTHKSQLGAGAYVGYQANPYLGFELGYDWLGRMAYKGSINNGAFRAQGVQLTTKLSYPVMDNLDVYTRLGGMVWRTDSSATYNANalRKKDHDTGVSPLAAIGVEYALTKNLATRIDYQWVNNIGDATTVGARPDNGMLSVGVSYRFGQDEAAPvvAPVVAPAPV
515941328.pir   VALAGFATVAQAAPKDNTWYAGAKLGWSQYHDTGFYGNGYdiGNGSTHESQLGAGAFGGYQVNPYVGFELGYDWLGRMEYTGNTENGAFKAQGIQATAKLSYPILDDLDIYTRLGGMVWRADSSAKNA.LGDRLKDHDTGVSPLYAAGLEWAVTKDVATRLEYQWVNNIGDAHTVGTRPDNGMLSLGVSYRFGQDAPAPVVAPVPAPAPE
639212611.pir   VALAGFATVAQAAPKDNTWYTGAKLGWSQYHDTGFDGNGYDNgsGPTHENQLGAGAFAGYQVNPYVGFEIGYDWLGRMEYKGQPENGALKAQGIQLTTKLSYPVTDDLDVYTRLGGMVWRADATAVNA.EGVRLKDHDTGVSPLAAGGVEWAVTRDIATRLEYQWTNNIGDRNTVGTRPDNGMLSVGVSYRFGQEEaaPVVAPAPAPAPE
666611786.pir   VAVAAFATVAQAAPKDNTWYTGAKLGWSQYHDVNFYGNGYDngNGPTRKNQLGAGAYVGYQANPYLGFELGYDWLGRMAYKGSVNNGAFRAQGVQLAAKLSYPVMDNLDVYTRLGGMVWRADSSATYNANarRLKNHDTGVSPLAAIGVEYALTKDLATRLDYQWVNNIGDAASVGARPDNGMLSVGVSYRFGQDDVAPVVVPAAPVIPA
238728287.pir   VALAGFATVAQAAPKDNTWYTGGKLGWSQYQDTGTGgyGDVGNDGPTHKNQLGAGAFLGYQANQYLGFEMGYDWLGRMPYKGDVNNGAFKAQGVQLAAKLSYPVAQDLDLYTRLGGMVWRADSSAYDAPTDTRSSNHDTGVSPLVALGAEYAWTKNWATRMEYQWVSNIGDRATVGARPDNGMLSLGVSYRFGQDDaaPVVAPAPAPAPV
575847724.pir   VAVATFATAAQAAPKDNTWYTGAKLGWSQYHDVNFYGNGYTdgNGPTRKSHLGAGAYVGYQANPYLGFELGYDWLGRIAYKGSVNNGSFRAQGVQLTTKLSYPVLDNLDVYTRLGGMVWRADSSATYNSNarRLKDNDTGISPLAAIGVEYALTKNIATRLDYQWTNNIGDANTVGARPDNGLLSVGVSYRFGQDEAAPVIPpaPAPAPV
640472995.pir   VALAGFATLAQAAPKDNTWYTGAKLGWSKYYDTGFDGNGFGSnnGPTHRTQLGTGAFVGYQANQYLGFEMGYDWLGRIPYKGDVNNGAFKAQGVQLATRLSYPIADDLDIYTRLGGMVWRADSKANYNNgdGRRSEHDTGVSPLAAIGLEYAVTPNWATRLDYQWTNNIGDKGTVGARPDNAMLSVGVSYRFGQDeaAPVIAPAPAPAPV
383296451.pir   VALAGFATVAQAAPQDNTWYTGGKLGWSQYHDHGMSNAYKGVGNGPTRDSQLGAGAFVGYQVNPYVGFELGYDWLGRMAYKGSVNNGAFKTQGIQLTTKLSYPLTDDLDVYTRLGAMVWRADSKGNFG.NGTRISDNDTGVSPVAAAGFEYAITKQWATRLEYQWVNNIGDAATVGARPDNANLSVGVSYRFGQEDAapVVAPAPAPAPV
391321428.pir   VALAGFATVAQAAPKDNTWYTGAKLGWSQFHDIG...NNQINNNGPTHESQLGAGAFGGYQVNPYVGFEMGYDWLGRMPYKGNTENGAFKAQGVQLTAKLGYPITDDLDVYTRLGGMVWRADAKNN.....DGFKDHDTGVSPVFAGGVEYAITPEIATRLEYQWTNNIGDANTVGGRPDNGLLSVGVSYRFGqeEAAPVVAPAPAPAPE
238707374.pir   VALAGFATVAQAAPKDNTWYTGGKLGWSQFHDVGT..GSDISNDGPTHKSQLGAGAFVGYQANQYLGFEMGYDWLGRMPYKGDTVNGAFKAQGVQLAAKLSYPIAQDLDLYTRLGGMVWRADASVNEPSTNSHASAHDTGVSPLAAVGLEYAVTKNWATRLDYQWVNNIGDRGTVGARPDNGMLSVGVSYRFGQDDavPVVAPAPAPAPV
595642407.pir   VALAGFATVAQAAPKDNTWYTGGKLGWSQYQNTGTGgyGDQGNDGPTHKDQLGAGAFLGYQANQYLGFELGYDWLGRMPYKGDVRNGDFKAQGVQLAAKLSYPIAQDLDLYTRLGGLVWRADSSAFNAASGERLNNHDTGVSPLVALGAEYAWTKNWATRMEYQWVSQIGDKATVGARPDNGMLSVGVSYRFGQDDaaPVVAPAPVPAPV
661561039.pir   MAVAAFATAAQAAPKDNTWYTGAKLGWSQYHDVNFYGNGYNdgNGSAHKNQLGAGAYVGYQANPYLGFELGYDWLGRIAYKGSVNNGAFRAQGVQLTTKLSYPVLDNLDVYTRLGGMVWRADSSATYNANarRLKNNDTGVSPLAAIGVEYALTKNLATRLDYQWVNNIGDATTVGARPDNGLLSVGVSYRFGQDEAAPVVAPVAPPVAP
320029504.pir   VALAGFATVAQAAPKDNTWYTGAKLGWSQYHDVGFYGNGYQDgnGPTHKNELGAGAFLGYQANQYLGFELGYDWLGRMPYKGSVNNGAFKAQGVQLAAKLSYPIVDNLDIYTRLGGMVWRADSKASYD.NVRRLSANDTGVSPLAAVGVEYALTKNLATRLDYQFVSNIGDAGTVGARPDNTMLSLGVSYRFGQNDVVAPVVSAPAPVVE
488393628.pir   VALAGFATVAQAAPKDNTWYTGAKLGWSQYHDTG.....FIPNNGPTHESQLGAGAFGGYQVNPYVGFEMGYDWLGRMPYKGDNVNGAFKSQGVQLTAKLGYPITDELDIYTRLGGMVWRADSKYNIP.GGASFKDHDTGVSPVFAGGLEWAVTRDIATRLEYQWTNNIGDANTVGTRPDNGLLSVGVSYRFGQQEAapVVAPAPAPAPE
555088851.pir   VALAGFATVAQAAPKDNTWYAGGKLGWSQFHDTGWY.NSSLNNDGPTHESQLGAGAFGGYQVNPYVGFEMGYDWLGRMPYKGDTINGAFKAQGVQLTAKLGYPITDDLDVYTRLGGMVWRADSSNNI.....SGDNHDTGVSPVFAGGVEWAMTRDIATRLEYQWVNNIGDGNTVGVRPDNGMLSVGVSYRFGqeEAAPVVAPAPAPAPE
EcOmpA.pir      VALAGFATVAQAAPKDNTWYTGAKLGWSQYHDTG.....FINNNGPTHENQLGAGAFGGYQVNPYVGFEMGYDWLGRMPYKGSVENGAYKAQGVQLTAKLGYPITDDLDIYTRLGGMVWRADTKSN.....VYGKNHDTGVSPVFAGGVEYAITPEIATRLEYQWTNNIGDAHTIGTRPDNGMLSLGVSYRFGQGEAAPVVAPAPAPAPE
647629804.pir   VALAGFATVAQAAPKDNTWYTGAKLGWSQYHDTGFYGNNYNVSNDP.HENQLGAGAYAGYQANQYLGFEGGYDWLGRMPYNG.TNHGAFKAQGVQLAAKLSYPLTDDLDVYTRLGGMVWRADSKDF....RNGNSDHDTGVSPLAAVGVEYALTKNWATRLDYQWVNNIGDAGTVGARPDNATLSVGVSYRFGQDdaAPAPAPAPAPAPV
308749336.pir   VALAGFATVAQAAPKDNTWYAGGKLGWSQFHDTGWY.NDDLNNNGPTHNSQLGAGAFGGYQVNPYVGFEMGYDWLGRMPYKGDKVNGAFKAQGVQLTAKLGYPITDDLDIYTRLGGMIWRADSSNSIA.....GDNHDTGVSPLVAGGVEWAITRDIATRLEYQWVNNIGDAGTVGVRPDNGMLSLGVSYRFGQGeaAPVVAPAPAPAPA
493948210.pir   VALAGFATVAQAAPKDNTWYAGAKLGWSQYHDTG.....FINNDGPTHENQLGAGAFGGYQVNPYVGFEMGYDWLGRMPYKGDNINGAYKAQGVQLTAKLGYPITDDLDVYTRLGGMVWRADTKSNVPggASNKDHDTGVSPVFAGGIEYAITPETAPRLESQWTNNIGEATTIGPRPDNGLLSVGVSYRFGQQEAAPVVAPAPAPAPE
639142206.pir   VALAGFATVAQAAPKDDTWYTGAKLGWSQFHDTGFYGNGYTVDN.DARKDQIGAGAFVGYQANQYLGFEMGYDWLGRMKYTGA.NTGSFKAQGVQLAAKLSYPIMNDLDIYTRLGGMVWRADSDGA....GSNGGNHDTGVSPLAAVGVEYALNRDWATRLDYQWVNNIGDAGTVGARPDNGMLSVGVSYRFGQEVAAPVVAPAPAPAPV
655274779.pir   VALAGFATVAQAAPKDNTWYTGAKLGWSQYHDTGFYGNNYGVEGSGT.KNQLGAGAFVGYQANPYLGFEFGYDWLGRMPYKNGVDHGAFKAQGLQLAAKLDYPITDDLDIYTRLGGMLWRAESKDH....GQGFKDHDTGISPLAAVGVEYALTPSWATRLDYQWTNNIGDAGTVGTRPDNSMLSVGVSYRFGQDTVAPAPAPAPAPAPV
661565430.pir   VAVAAFATAAQAAPKDNTWYTGAKLGWSQYHDVNFYGNGYNdgNGPTRKNQPGVGAYVGYQANPYLGFELGYDWLGRMNYNGSINNGSFRAQGVQLTTKLSYPVLSDLDVYTRLGGMIWRADSSATYNanQSSLKNHDTGVSPLAAVGVEYALTKNLATRLDYQWVNNIGDANSVGARPDNGLLSVGVSYRFGQDEVAPIAAPVAPPVAP
282948231.pir   VALAGFATVAQAAPKDNTWYTGAKLGWSQYHDTG.....FIDNNGPTHENQLGAGAFGGYQVNPYVGFEMGYDWLGRMPYKGSVENGAYKAQGVQLTAKLGYPITDDLDIYTRLGGMVWRADTKSHNNLTGASTKDHDTGVSPVFAGGIEWAMTRDIATRLEYQWTNNIGDANTIGTRPDNGLLSVGVSYRFGQQEEAAPIAPAPAPAPE
238713147.pir   VALAGFATVAQAAPKDNTWYTGGKLGWSQFQDTG....SIIENDGPTHKDQVGAGAFLGYQANQYLGFEMGYDWLGRMPYKGDTNNGAFKAQGVQLAAKLSYPVAQDLDVYTRLGGMVWRADATGSI..NGDRTSGHDTGVSPLVALGAEYAWTKNWATRMEYQWVNNIGDRATVGARPDNGMLSVGVSYRFGQDDAVVPVAPAPAPV
668995539.pir   VALAGFASVAQAAPQDNTWYAGGKLGWSQYHDTGWY.DSALNNNGSTHNSQLGAGAFGGYQVNPYVGFEMGYDWLGRMKYTGSQTNGALKAQGVQLTAKLGYPVTDDLDIYTRLGGMVWRADSSNN.....QIGDNHDTGVSPVFAGGVEWAVTRDIATRLEYQWVNNIGDANSVGVRPDNGMLSVGVSYRFGqdAAAPVIAPAPAPAPE
586611009.pir   VALAGFATVAQAAPKDNTWYTGGKLGWSQYHDKG....AVWQNDGPTHESQLGAGAFGGYQVNPYVGFELGYDWLGRMPYKGDTVNGAFKAQGVQLTAKLGYPVTDDLDLYTRLGGMVWRADAQNN.....QGFKDHDTGVSPVFAGGVEWAVTRDIATRLEYQWVNNIGDANTVGARPDNGMLSVGVSYRFGQQeaAPVvaPAPAPAPE
652342851.pir   VALAGLASVAQAAPNDNTWYAGAKLGWSQFHDTGLYGNGYQTTNSP.HENQLGAGAFGGYQANQYLGFELGYDWLGRMKYTGE.NRGNFKAQGVQLAAKLSYPIVDDLDVYTRLGGFFWRADSHDDV....TGRGGNDTGISPLAAVGLEYAVTKNWATRLDYQWVNNIGDAGTVGGRPDNGMLSVGVSYRFGQETPAPVVAPTPAPAPV
455420285.pir   VAVAAFASVAQAAPKDNTWYTGGKLGWSQYQNTGTGFDGESLGngPVHKDQLGAGIYAGYQHNQYMGFELGYDWLGRMPYKGDGNNGAFKAQGVQLTTKLSYPVMDDLDVYTRLGAMVWRADATASY..NGERIKDNDTGVSPLFALGTEYAITRDIAARVEYQWINNIGDKETTMARPDNGMLSVGVAYRFGQDeaAPVAEPAPVVAPV
51589075.pir    VALVGFATVAQAAPKDNTWYTGGKLGWSQYQDTG....SIINNDGPTHKDQLGAGAFFGYQANQYLGFEMGYDWLGRMPYKGDINNGAFKAQGVQLAAKLSYPVAQDLDVYTRLGGLVWRADAKGSFDGGLDRASGHDTGVSPLVALGAEYAWTKNWATRMEYQWVNNIGDRETVGARPDNGLLSVGVSYRFGQEDAapIVAPTPAPAPI
668658916.pir   VALAGFATVAQAAPKDNTWYTGAKLGWSQYHDTGFYGNGYRVDN.DARKDQLGAGAFLGYQANQYLGFEMGYDWLGRMKYTG.RNTGSFKAQGVQLAAKLSYPIVDDLDIYTRLGGMVWRADSDGV....GSNGGNHDTGVSPLAAVGVEYALTKNWATRLDYQFVSNIGDAGTVGARPDNTMLSLGVSYRFGQDDvaPAPAPAPAPVVE
242132173.pir   IAVAGFATVAQAAPNDNTWYAGGKLGWSQFHDTGLNGSGYQTTN.DAQENQLGAGVFGGYQANPYLGFEMGYDWLGRMKYTG.VNRGNFKAQGVQLAAKLSYPIVDDLDVYTRLGGFVWRADSHDDV....SGNSGHDTGVSPLAAVGVEYAITKNWATRLDYQWVNNIGDASTVGGRPDNGLLSVGVSYRFGQEAPAPIVAPAPAPAPT
194682162.pir   VAVAAFATAAQAAPKDNTWYTGGKLGWSQYQSTGNNWDGVNIGNGSTHKDQIGAGAFAGYQYNQYLGFELGYDWLGRMAYKGSYNNGAFKAQGIQLTTKLSYPVMDDLDVYTRLGGMVWRADSTATINatQKRFSENDTGVSPVFALGTEYAITPNIATRLEYQWINNIGDKGTLNARPDNGMLSVGVAYRFNQETPAPVVEPAPVVAPA
498975325.pir   VALAGFATVAQAAPKDNTWYAGGKLGWSQYHDTG.....FINNNGPTHENQLGAGAYGGYQVNPYVGFELGYDWLGRMEYKGngQNGAYKAQGVQLTAKLGYPITDDLDIYTRLGGMVYRADTTSNSATG..RHKDHDTGVAPVFAGGVEYAITPQIATRLEYQWTNNIGDRSEIGTRPDNGMLSVGVSYRFGQDEaaPIPAPAPAPEVQ
497991444.pir   VALASFATIAQAAPKDNTWYTGGKLGVSQFHDTGFYGNGYTGVNNNPIKSKLGAGAFVGYQANPYLGFELGYDWLGRMKYAGSTSNVafKAQGIQLAAKLSYPVLPDLDVYTRLGGMVWRADSHAAI..GGGDINNDDTGVSPLAAIGVEYAIDKNWATRVDYQWVSNIGDAGTVGARPDNMLMSVGLSYRFGQDdVAPVVAPAPTPAPA
558649082.pir   VAVAAFATAAQAAPKDNTWYTGGKLGWSQYQGTSNHFGETSIGNGNTHRDQLGAGAFAGYQYNQYLGFELGYDWLGRMTYKGSYDNGAFRAQGIQLTTKLSYPVMEDLDVYTRLGGMVWRADSSATVAatQKNFSNNDTGVSPVFALGTEYAITPNIATRVEYQWINNIGDKGTLNARPDNGMLSVGVAYRFNQETPAPVVAPAPVVAPA
666391907.pir   VALAGFATVAQAAPKDNTWYTGAKLGWSQYHDTGF..VDGAIGNGPTRDSQLGAGAFFGYQANQYMGFEMGYDWLGRMAYKGNVNNGALKAQGVQLTTKLSYPIMNDLDIYTRLGGMVWRVDSTAYFGGnnERVSGDDTGVSPLAAIGFEYALNQNWATRLDYQWTSNIGDAQTVGARPDNGQLSVGVAYRFGQDTVAPVVAPAPTPAPT
549989173.pir   VALAGFATVAQAAPNDNTWYAGGKLGWSQYHDTGLTGNGYNVTNAAQ..NQLGAGAFGGYQANPYLGFEMGYDWLGRMKYNG.GNQGSFKAQGVQLAAKLSYPIVDDLDIYTRLGGFVWRADSHDN.....SGLNDHDTGVSPLAAVGVEYAITKNWATRLDYQWVNNIGDASTVGGRPDNGLLSVGVSYRFGQETAAPAPVIAAAPAPT
640732380.pir   VAVAAFASVAQAAPKDNTWYTGGKLGWSQYQNTG....STIGNTGPTHKDQVGAGIYAGYQHNQYMGFELGYDWLGRMPYKGDENNGAFKAQGVQLTTKLSYPVMDDLDVYTRLGAMVWRADSSYSTPANRTQTTNNDTGVSPLFALGTEYAITRDIAARVEYQWINNIGDKETTLARPDNGMLSVGVAYRFGQDeaAPVAEPAPVVAPV
385872404.pir   VALASFATIAQAAPKDNTWYTGGKLGVSQFHDTGFYGNGYTGINNNPIKSKLGAGAFVGYQANPYLGFELGYDWLGRMKYAGsaDSASFKAQGIQLATKLSYPVMPDLDVYTRLGGLVWRTDSHADRS..GTHLNNNDTGVSPLAAVGIEYAIDKNWATRVDYQWVSNIGDAGTVGARPDNLLMSVGLSYRFGQDdVAPVVAPAPTPAPA
542100660.pir   VALAGFATVAQAAPNDNTWYVGGKLGWSQYHDAGMNGNGYQTTN.DAQKDQLGTGIFAGYQANPYLGFEMGYDWLGRMKYVGA.NRGNFKAQGIQLAAKLSYPIMSDLDVYTRLGGFVWRADSSDD....ATGNSGHDTGVSPLAAIGVEYAITKNWATRLDYQWINNIGDASTVGGRPDNSMLSVGVSYRFGQdaAAPTPAPAVEPAPA
414096805.pir   VAVASFATVAQAAPKDNTWYTGAKLGWSHYEDTKFNPVDRNAGSVGtdRDQLGAGAYAGYQYNQYLGFELGYDWFGKMKYKGDGNHGDLKSMGVSLATKLSYPVMDDLDVYTRLGGMVWRTDVKANQA.GQAHYKENDTGVSPLYALGVEYAITPNWATRLDYQWVSKIGDNNSLGARPDNGMLSVGLAYRFNQ.EAAPVVEPAPVVEPA
353674958.pir   VALAGFATVAQAAPKDNTWYTGGKLGWSQYHDTSLYGNGYRVSNNP.DESQLGAGAFLGYQANQYLGFELGYDWLGRMKYTG.TYHGDYRAQGVQLSAKLSYPVATDLDVYTRLGGFIWRVDSSDD.....LGHSNNDTGVSPLAAAGLEYAIDKNWATRLEYQWVNNIGDAATVGGRPDNGLLSVGVSYRFGQDDNYVAPAPAPAPAPV
402279534.pir   ......................AKLGWSQYHDTGFYGNGFQGNNGPTRNDQLGAGAFGGYQVNPYLGFEMGYDWLGRMAYKGSVDNGAFKAQGVQLTAKLGYPITDDLDIYTRLGGMVWRADSKGNYASTGVSRSEHDTGVSPVFAGGLEWAVTRDIATRLEYQWVNNIGDAGTVGTRPDNGMLSLGVSYRFGQDDVAPVVAPAPAPAPE
414100436.pir   VAVAAFATVAQAAPKDNTWYTGAKLGWSHYEDTKFtfDNGTSVGNSTDRDTLGAGAYAGYQYNEYMGFELGYDWFGKMKYKGAGNSGDLKSMGVSLTTKLSYPIMDDLDVYTRLGGMVWRTDVKAHNDNLG.KIKENDTGVSPVYALGVEYAITPNWATRLDYQWVSNIGDKDNLGVRPDNGMLSVGLAYRFNQ.EVAPVVEPAPVVAPA
384481477.pir   VAVAAFATVAQAAPKDNTWYTGAKLGWSHYEDTSFTPVDRENTVgsTDRDTFGAGAYAGYQYNQYMGFELGYDWFGKMKYKGAGNNGDLKSMGVSLTTKLSYPVMDDLDVYTRLGGMVWRTDVKAH.QTGEERYKENDTGVSPVYALGVEYAITPNWATRLDYQWVSNIGDNDNLGVRPDNGMLSVGLAYRFNQ.EAAPVVEPAPVVEPA
320577062.pir   ..................WYTGSKLGWSQYHDTGFYGNGFDnaNGPTRDDQLGAGAFLGYQANPYLGFELGYDWLGRMAYKGSVNNGAFKAQGISLAAKLSYPLTDDLDIYTRLGGMVWRADSKGAYDNGNarVSDHDTGVSPLAAVGLEYAWTKNWATRLDYQWVNNIGDAGTVGTRPDNSMLSVGVSYRFGQDEVAP.PAPVVQTKHF
532965078.pir   VALAGFATVAQAAPKDNTWYTGAKLGWSQYHDTG.....FINNNGPTHENQLGAGAFGGYQVNPYVGFEMGYDWLGRMPYKGDNINGAYKAQGVQLTAKLGYPITDDLDIYTRLGGMVWRADTKANEP.GGASFKDHDTGVSPVFAGGVEYAITPEIATRLEYQWTNNIGDANTIGTRPDNGLLSLGVSYRFG.................
647476296.pir   VALAGFATVAQAAPKDDTWYVGGKLGWSHYYDNS....INHFGSTNVRPDQLGGGAFFGYQANPYLGFEMGYDWLGRMEYRGN.NNGAFKSQGVQLAAKLSYPIADDLDIYTRLGGMVWRADGSANSETRGRYIDSHDTGVSPLAAIGVEYALNKDWATRLDYQFVSNIGDANETAARPDNTLLSVGVTYRFGQDEAAPVVAPAPAPAPV
414096252.pir   VAVAAFATVAQAAPKDNTWYTGAKLGWSHYEDtgTDYAGWGTVGNSTDRDTIGAGAYAGYQYNQYLGFELGYDWFGKMKYKGAGNSGDLKSMGVSLTTKLSYPIIDDLDVYTRLGGMVYRTEAKAHQ..NGERFKETDTGVAPVYALGLEYAITPEWATRLDYQWVSNLGDKDNIGTRPDNGMLSVGVAYRFGQAD.APVVAPAPVVAPA
653021557.pir   VALAGFATVAQAAPLDNTWYTGAKVGWSYYHDTGFY..NKYASGADTHPHQLGAGAYVGYQANQYLGFEFGYDWLGRMTYRGD.DSAAYKAQGIQLSAKLSYPIMNDLDIYTRLGAMGWRGDVTAHPAAGGTY.KDHETGVSPLAAVGVEYAITPSLATRLDYQWVNNIGDADSVGTRPDNGMLSLGLAYRFGQTKPAPAVALPVVQTKR
284007554.pir   VAVAAFATAAQAAPKDNTWYTGAKLGWSHFEDTGFHPYGDNVGSGKTKRDQLGAGAYLGYQHNQYLGFELGYDWFGRMRYSGQPNNGSLKSMGVQLTARLSYPLTDELDVYTRLGGMVWRTDAKANVE.NQPSFKEHDTGVSPVYALGFEYAITPEWATRLDYQWVSHIGDKDSLSVRPDNGMLSVGVAYRFNQ.EPAPVIVPAPAPIEN
145688456.pir   VALAGFATVAQAAPKDNTWYVGGKLGWSHFISNSF....EDMGTTKPHPNQLGAGAFFGYQANPYLGFEMGYDWLGRMGYTGDV.NAKFKSQGVQLAAKLSYPLMDDLDVYTRLGGMVWRSDIHGAAD.GGDYTSSHDTGVSPLAAIGVEYALNKDWATRLDYQYVNKVGTRSETGARPDNTMLSLGVVYRFGQDeaAPAPIPAPAPAPV
385191048.pir   VAGLAAASVAQAAPQENTFYAGVKAGQASFHDGIKANTDASnfGTGYNRNSFTYGVFGGYQinLGLAVELGYDDFGRAKFKDAGKVakHTNHGAHLSLKGSYGllEGLDVYGRVGAALVRSDYKDYDATSGARngEHSLRVSPMFAAGVEYAllPELALRLEYQWLTRVGKLRtvDYNPWIGSINAGLSYRFGQG.AAPVVAPEVVSKTF
288225941.pir   ...................YTGAKLGWSQYHDKGYYGNSYQDNDGPSHESQLGAGAFLGYQANPYLGFELGYDWLGRMPNKGYQVNGAFKAQGVQLAAKLSYPVMDDLDVYTRLGGIVWRVDSTQQVV.GGDRISDHDTGVSPLAAVGVEYAVTKNWATRLDYQWVNNIGDAGTVGARSDNSMLSVGVSYRFGQDEAapVVAPAPVVETK
532965070.pir   VALAGFATVAQAAPKDNTWYAGGKLGWSQFHDTGWY.NSSLNNDGPTHESQLGAGAFGGYQVNPYVGFEMGYDWLGRTPYKGDNVNGAFKAQGVQLTAKLGYPVTDDLDVYTRLGGMVWRADSSNSIA.....GDDHDTGVSPVFAGGVEWAMTRDIATRLEYQWVNNIGDGNTVGVRPDNGMLSLGVSYRFG.................
517168214.pir   VAGLAATSVAQAAPQENTFYVGAKAGWASFHDGLKQFDSKHvnGFGVNRNSVTYGVFGGYQinLGLAVELGYDDFGRAKfkGEKVVSKHTNHGAHLSLKGSYEvLEGLDLYGRAGAALVRSDYKNAKGVEDsgKDAHSLKVSPVFAAGVEYAllPELALRVEYQWLASVGKDNKidYTPWIGSVTAGLSYRFGQG..APVVAPEVVSKTF
491435098.pir   VALAGFATVAQAAPKDDTWYVGGKLGWSHYIGNSFENMD....STKSHPNQLGAGAFFGYQANPYLGFEMGYDWLGRMGYTGDV.NGKFKAQGIQLAAKLSYPLMDNLDVYTRLGGMVWRADAHASDA..TDYVSGHDTGVSPLAAIGVEYALNKDWATRLDYQYVNKIGTSSQTGARPDNTLLSLGVVYRFGQDDvaPAPIPAPAPAPI
301156596.pir   VAGLAAASVAQAAPQENTFYVGAKAGLASFHDGIKANTDATnfGTGYNRNSVTYGVFGGYQinLGLAVELGYDDFGRVKFKDAGKTvkHTNHGTHLSFKGSYGLTEGLDFYGRAGAALVRSDYKVYDETTGAReGRHSLRVSPVFAAGFEYAllPELALRLEYQWLARVGKLRtvDYNPWIGSINAGLSYRFGQG.AAPVVAPEVVSKTF
656055076.pir   MAFAGFTTVAQAAPLDNTWYTGAKVGWSNYHDTGFYDNTLNNSNDQPH..QLGAGAYMGYQANQYLGFELGYDWLGRMTYRGA.DSGAFKAQGIQLAAKLSYPIMNDLDLYTRLGAMGWRADTT.YHPSTGGEHKNHDTGVSPLAAIGVEYAITPSIATRLDYQWVNNIGDGDSIGTRPDNGTLSLGVAYRFGQTQPAPVIAVPVVETKR
325160326.pir   VAGLAAASVAQAAPQANTFYAGAKAGQASFHDGIKANTDATnfGTGYNRNSVTYGVFGGYQinFGLAVELGYDDFGRAKFKHNGItfKHTNHGAHLSVKGSYDllEGLDFYGRAGAALVRSDYKVYNAKGahGAGEHSLRVSPVFAAGFEYAllPELALRLEYQWLARVGKLRtvDYNPWIGSINAGLSYRFGQG.AAPVVAPEVVSKTF
652755191.pir   LAVLSAAAVAQAAPQANTFYAGAKAGWASFHDGLTQFDAKDnnGYGINRNSVTYGVFGGYQinFGLATELGYDYFGRVRGNteKRAAKHTAHGAHLSLKPSYEIAPNLDVYGKVGVALVRNDYYqqNVAKDSRIKAHNLKPSLLGAGLEYAITPELAARVEYQYLNRVGNLdnSQYSPDIHSVSAGLTYRFGQG.AAPVEAPEVVTKNF
532965072.pir   VALAGFATVAQAAPKDNTWYTGGKLGWSQYQNSG....SIINNDGPTHKDQLGAGAFLGYQANQYLGFEMGYDWLGRMPYKGDTNNGAFKAQGVQLAAKLSYPVAQDLDVYTRLGGMVWRADADGSI..GGVRNSNHDTGVSPLVALGAEYAWTKNWATRMEYQWINNIGDRATVGARPDNGMLSVGVSYRFG.................
```

```
110086793.pir    IAGLTAASVAQAAPQANTFYAGAKAGWASFHDGLNQFED.QENAQGTLRNSVTYGVFGGYQITDNFAVELGYDDFGRAKlkGGETVIKHTNHGAHLSLKASYPVLEGLDIYARVGAALIRSDYKSTKRAESDYvhEHSLKVSPVFAGGLEYNllPELALRVEYQWVNKVgdGSRvDYTPSIGSVTAGLSYRFGQS..APVVEPKVVAKTF
575444313.pir    LAVLSAAAVAQAAPQANTFYAGAKAGWASFHHGTKQFDSARAgnYGINRNSVTYGVFGGYQinFGLATELGYDYFGRVRGngDERAVKHTAHGAHLSLKPSYEIAPNLDAFGRVGVALVRNDYKAYGAanPATAKAHNLKPSLVLGGGLEYAITPELAARVEYQWLSKAGNYAkvKYSPDIHSVSAGLSYRFGQAPVEAPEVVTKNF
575850166.pir    VAVATFATIAQAAPKENTWYAGAKLGWSQFNKTKFTDFGSNAagDAINRNQLGTGAYIGYQATPYLGFELGYDWLGRIKYKSSANNGDFRAQGVQLTAKLSVPVTNDLDVYTRLGGMVWHSKAAAHYNSKdVNVKTSSTGISPLIAIGAEYALTKNLATRLDYQWTPKVGRGGdiGARPNIGLLTVGISYRFGQDEAIPvvTPAPTPATE
648091927.pir    IAGLAAAVAQAAPQANTFYAGAKAGWASFHDGLNQFENSQ.NAYGTLRNSVTYGVFGGYQITDNFAVELGYDDFGRAKlRQDGEtgKHTNHGAHLSLKASYPVLEGLDVYARVGAALIRSDYKPTKRAAPNETHEHSLKASPVFAGGLEYNllPELALRVEYQWVNKVgnGSRvDYTPSIGSVTAGLSYRFGQS..APVVEPKVVAKTF
343393839.pir    VAGLAAASVAQAAPQENTFYAGAKAGWASFHDGIKYTADHFVYTqgFNRNSVTYGVFGGYQinLGLAVELGYDDFGRAKFRAEGQnvKHTNHGAHLTLKGSYDllQGLDVYGRAGVALVRSDYKFYNLEGEKWHKEHSLKASPVFAAGLEYAllPELALRLEYQWVNKVgnGDRvDYAPWIGSVTAGLSYRFGQG.AAPVVAPEVVNKTF
573500222.pir    VAVAAFATVAQAAPKDNTWYTGGKLGWSHYEHadAAGDETSVGTGKKTRDQLGAGLYAGYQYNQYMGFEMGYDWFGKMKYKGD.NAGSVSSMGVSLTTKLSYPIMDDLDVYTRLGGMVYRTEAKVN.....GVGKETDTGVSPVYALGLEYAITPELATRLDYQWVSRMGDKDNIGVSPDNGMLSVGLAYRFGQ.EAAPVVAPAVVAEPA
385200765.pir    IAALAAASVAQAAPQPNTFYVGAKAGWASFHDGLNQFNDITEPATGFKRNSVTYGVFGGYQITDNFAVELGYDDFGRAKakETVGAAKHTNHGAHLSLKASYPVLDGLDVYARVGAALVRSDYKVYdaDLSFLKRTHSTQVSPVFAGGLEYAFMPELALRVEYQWLNNVGKLeRVDYRPDIGSVTAGLSYRFGQSVYVPEVVSKTFTLNS
387134155.pir    IAALAAASVAQAAPQPNTFYVGAKAGWASFHDGLNQAKDFdaGSFGFKRNSVTYGVFGGYQITDNFAVELGYDDFGRAKLRNTVGeaKHTNHGAHLSLKASYPVLEGLDVYARVGAALVRSDYKLYDKENTPSLQdhSTQVSPVFAGGLEYAFIPELALRVEYQWLNNVGKLKnvDYRPDIGSVTAGLSYRFGQSVYVPEVVSKTFTLNS
247533723.pir    IAGLAAATVAQAAPQANTFYAGAKAGWASVHHGLGQFTD..GNDGKIRNSEAYGIFGGYQITDNLAVEAGYEYFGRAKVKYSgDQFRHTAHGTTLALKGSYPVLDNLDVYGRAGAALIRSDYKQVND..GVADKYHNLKVSPVFAAGVEYAILPELALRAEYQWVSRVGNLGkvRYSPDIGSVAVGLSYRFGQG.AAPAPAPEVVSKTF
347605235.pir    VALAGFATVAQAAPRDETWYAGAKVGWSGYHDTLMNHAYKGVGDGSTRKDQLGAGAFLGYQINQYLGLEMGYDWFGRMHYSGDVKNGAFKAHGVQLAAKLSYPLEDDLDVYTRLGGMLWRADSTGNLGDPNKKIRDYDIGVSPVAAVGLEYALTQNWVTRLDYQFVNNMGDEATIGTRPDNTNLSLGVAYRFGQDAPIVPIPPTPAEPER
404383034.pir    IAALAAASVAQAAPQPNTFYVGAKAGWASFHDGLNQAKYLEDADagFKRNSVTYGVFGGYQITDNFAVELGYDDFGRAKLRIAedVAKHTNHGAHLSLKASYPVLDGLDVYARVGAALVRSDYKVYdaKLPQLKRTHSTQVSPVFAGGLEYAFMPELALRVEYQWVNNVgdGNRvDYRPDIGSVTAGLSYRFGQSVYVPEVVSKTFTLNS
42528870.pir     ...............GSNYVMLTWRRPQYHDTG.....FIHNDGPTDENQLGAGAFGGYQVNPYVGFEMGYDWLGRMPYKGDNINGAYKAQGVQLTAKLGYPITDDLDVYTRLGGMVWRADTKSNVP.GGPSTKDHDTGVSPVFAGGIEYAITPEIATRLEYQWTNNIGDANTIGTRPDNGLLSVGVSYRFGQEAAPVAPAPAPE
341949621.pir    VAGLAASVAQAAPQENTFYAGVKAGQASFHDGIKANTDATnfGTGYNRNSFTYGVFGGYQinLGLAVELGYDDFGRVKFKDAGkaAKHTNHGTHLSLKGSYEVLNGLDVYGKAGVALVRSDYKFYDENGarAEGRHSLRTSGLFAVGAEYAVLPELAVRLEYQWLTRVGKLRtvDYNPWIGSINAGVSYRFGQGAAPVVAAPEVVSKTF
315476784.pir    IAGLAAATVAQAAPQADTFYAGAKAGWASVHHGLNQ..FKDEPGQTVSRNSEAYGVFGGYQITDNLAVEAGYEYFGRAKAKTNGehFRHTAHGTTLALKGSYPVLADLDVYGRAGAALIRSDYKFTD..GSDVEKYHNLKVSPVFAAGVEYAILPELALRAEYQWVSRVGNLGkvRYSPDIGSVALGLSYRFGQG..APVVAPEVVNKTF
302650272.pir    LAVLSAAAVAQAAPQQNTFYAGAKVGWATFHEGVRQFNDKYSNDagINRNSVTYGVFGGYQinFGLATELGYDYYGRVRGngEFAMKHSAHGLNLALKPSYEVLPNLDVYGKVGAVAVRNDYKRYGatNEPTEKFHKLKASTILGAGVEYAILPELAARIEYQYLNKAGNLNKALvrpDIHSVTAGLSYRFGQGAVAPVVEPEVVTKNF
575444312.pir    LAVLSTAAVVQAAPQANTFYAGAKAGWASFHDGLTQFDHKDGGKFGINRNSVTYGVFGGYQinVGLAVELGYDYFGRVRGngEDKAVKHVAHGTHLSLKPSYEVAPNLDVYGKVGVALVRNDYKGYNNgnYEDQRAHNLKTSLLLGAGLEYAITPELAARVEYQWLNRVGNLdnSQySPDIHSVSAGLTYRFGQG.AAPV.EPTVETKNF
329755864.pir    IAALAAASVAQAAPQPNTFYVGAKAGWASFHDGLNQFKDLNDEDagFKRNSVTYGVFGGYQITDTFAVELGYDDFGRAKLRVAedLAKHTNHGAHLSLKASYPVLGDLDVYARVGAALVRSDYKLYGESEdeFKRTHSTQVSPVFAGGLEYAFIPELALRVEYQWLNNVgnGERvDYRPDIGSVTAGLSYRFGQTVYVPEVVNKTFTLNS
306852754.pir    LAVLSAAAVAQAAPQQNTFYAGAKVGQSSFHGVNQLKSGHDdkYGINRNSVTYGVFGGYQinFGLAAELGYDYYGRVRGNVderTVKHSAHGLNLALKPSYEVLPDLDVYGKIGavVRNDYKKYGatNESTTeFHKLKASTILGAGVEYAILPELAARVEYQYLNKAGNLNKALvrpDIHSVTAGLSYRFGQGAVAPVVEPEVVTKNF
388301.pir       VAGLAASVAQAAPQENTFYAGVKAGQASFHDGLRALA..REKNVGYHRNSFTYGVFGGYQinLGLAVELGYDDFGRAKGREKGKTVahTNHGAHLSLKGSYEVLDGLDVYGKAGVALVRSDYKFYEDANGTRdgRHTARASGLFAVGAEYAVLPELAVRLEYQWLTRVGknTAINyNPWIGSINAGISYRFGQGAAPVVAAPEVVSKTF
404384025.pir    IAALAAASVAQAAPQPNTFYVGAKAGWASFHDGLNQAKDVSE.NLEVKRHHVTYGVFGGYQITDNFAVELGYDDFGRAKLngKLNIAKHTNHGAHLSLKASYPVLDGLDVYARVGAALIRSDYKVYdaKDLTHSLNRSLRvsPVFAGGLEYAFIPELALRVEYQWVNNVgnGERvDYRPDIGSVTAGLSYRFGQSVYVPEVVSKTFTLNS
299773255.pir    VSGLAVASVAQAAPQANTFYAGAKAGWATFHNDINQLNSkSANGYVINRNSVTYGVFGGYQIDNLAVELGYDYFGRVRgkQEFRAFKHSAHGTHLSLKPSYEVLNGLDVYGKVGAALVRNDYKRYSQTAgqTQKAHNLKTSLVLGAGVEYAILPELALRLAFRVEYQWLSNVGnrATYNYSPDSHSVTAGLSYRFGQGAAPVAAAPEVVTKNF
229811200.pir    VAGLAAASVAQAAPQENTFYAGVKAGQGSFHDGINNNGAIKnnSYGYRRNTFTYGVFGGYQinFGLAAELGYDNFGRVKLRLAGkqAKHTNHGAHLSLKGSYEVLDGLDVYGKAGVALVRSDYKFYEAANGTRdgRHTARASGLFAVGAEYAVLPELAVRLEYQWLTRVGknTAINyNPWIGSINAGISYRFGQGAAPVVAAPEVVSKTF
144986331.pir    VAGLAASVAQAAPQENTFYAGVKAGQGSFHDggAIKEALTSASYGYRRNTFTYGVFGGYQinFGLAAELGYDNFGRAKLREVGksAKHTNHGAHLSLKGSYEVLDGLDVYGKAGVALVRSDYKRYEEANGTRNHdhSLRTSGLFSVGAEYAVLPELAVRLEYQWLTRVGKLRtiNYNPWIGSINVGISYRFGQGEAPVVAAPEMVSKTF
408795016.pir    IVALAVASVAQAAPQPNTFYVGAKAGWASFHDGLNQIEYKSETSFGSKRNSVTYGVFGGYQITDNFAVELGYDDFGRAKVRANSdAAKHTNHGTHLSLKASYPLLDGLDVYARVGAALIRSDYKLYAPLISERLSPhiTQVSPVFAGGLEYAFIPELTLRVEYQWVNNVgdGQYADFRPDIGSVTAGLSYRFGQGSVYVPEVVSKTFTLNT
209968918.pir    VSGLAVASVANAAPQANSFYVGAKAGWATFHNDINQLNSKYENDagINRNSVTYGVFGGYQIIDNLAVELGYDYFGRVRGKeeFRAFKHSAHGTHLSLKPSYEVLNGLDVYGKVGAALVRNDYKFY....DTEDKAHNLKTSLLLGAGVEYAILPELAFRVEYQWLSRVGNANksTYSPDAHSVSAGISYRFGQGAaaPAAAPEVVTKNF
341952950.pir    VASLAAASVAQAAPQENTFYAGVKAGQASFHDGLKANTVEHATNagYKRNSFTYGVFGGYQinLGLAVELGYDDFGRAKfnAGKTSLKHTNHGAHLSFKGSYEVLNGLDVYGKVGAALVRSDYKFYDENGARERAdhSLRTSGLFAVGAEYAVLPELAVRLEYQWLTRVGKYRtvDYNPWIGSINAGVSYRFGQGAAPVVAAPEVVSKTF
575442127.pir    LAVLSTAAVVQAAPQANTFYAGAKAGWASFHDGLTQFDAKDNkgFGINRNSVTYGVFGGYQinVGLAVELGYDYFGRVRGngEDKAIKHVAHGTHLSLKPSYEVAPNLDVYGKVGAALVRNDYKGYN..GYKDQRAHNLKTSLLLGAGLEYAITPELAARVEYQWLYQAAgdWIDAQYSPKIHSVSAGLTYRFGQG.AAPVEAPEVVSKTF
517168215.pir    IAALSVVSIAQAAPQENTFYVGAKAGWASFHDGIKY..HADNTQHGYNRNSVTYGVFGGYQinFGLAVELGYDDFGRVKFENGGEGRVAKHTNHGTHLSLKASYEILQGLDVYARVGAALVRSDYKIYTPSGPFYDNSHSLKVSPVFAGGMEYAllPELAFRLEYQWINRVGKHDSEGLRLdiGSVVAGLSYRFGQG..APVVAPEVVSKTF
99032472.pir     ..........MAPKDNTWYTGLGFSQYHDTG....FINNNGPTHENQLGAGAFGGYQVNPYVGFEMGYDFLGRMPYKGSVENGAYKAQGVQLTAKLGYPITDDLDIYTRLGGMVFRADTKSN.....VYGKNHDTGVSPVFAGGVEYAITPEIATRLEYQFTNNIGDAHTIGTRPDNGMLSLGVSYRFGQGEAA...........
497813888.pir    LAVLAASAATAAPQENTFYAGAKAGWAAFHHGIKQIDDKHATDagINRNSVTYGVFGGYQinFGLATELGYDYYGRVRGNDRVNNskHSAHGLNLSLKPSYAVLPDLDVYGKVGAALVRNDYKFYDVTggNHSKYHNLKPSLTLGGGVEYAITPELAFRVEYQYLNKAGNYSktKYSPDIHSVSAGLTYRFGQGAaaPVAEPQIETKNF
247533937.pir    GLVVAVATVAQAAPQANTFYAGAKAGWASVRHGLNQYQYEDSDGAVkINSEAYGVFGGYQITDNFAVEAGYEFFGRGKVeaGGEKRITAHGSSLSLKASYPALDNLDLYARAGALIRSDYVKDT..TEGKTHkFHDLKVSPVFAAGVEYAILPELALRAEYQWVARVGNFGKvRYSPDIGSVSLGLSYRFGQG.AAPIVAPEVATKVF
306852258.pir    LTVLSAAAVAQAAPQQNTFYAGAKAGWASFHDGIEQLDSAKNTDRGtnRNSVTYGVFGGYQIllGLAAELGYDYFGRVRGSEKPNGKADkaHGATIALKPSYEVLPDLDVYGKVGIALVNNTYKTFNA.AQEKVkTRRFQSSLILGAGVEYAILPELAARVEYQWLNNAGKagATDYRSDISSVSAGLSYRFGQG.AAPVAAPAVETKNF
240295730.pir    LAVLAASAATAAPQENTFYAGAKAGWANFRDGFTQfnPKLNNGYGINKNTVTYGVFGGYQinFGLAAELGYDYFGRARGNTtkVAAKHTAHGAHLSLKPSYEVIPNLDVYGKVGALIRNDYFvsTPVKGKRTKVHTLKPSLTLGAGVEYAILPELAARIEYQYVNKVGNLdnYQFSPDLHSVSAGLSYRFGQG.AAPVAAPAEVVTKN
517157242.pir    ISGLAFVSVAQAAPQANTFYAGAKAGWASFHNDVNQFNDIYGQDagVHRNSVTYGVFGGYQITDNFAVEAGYDFYGQLkhAGDTERSRHTAHGLNLSLKASYPVadGLDVYGRLGAALIRSDYTTHVN.GEKVGSDHKLKVSPVFAAGVEYAILPELALRVEYQWIAKVGkaDDYSySPSIGSVSLGLSYRFGQS.AAPVVEPVVNKKF
386905158.pir    IAGLAAASIAQAAPQENTFYAGAKAGWASFHDGLNQLKDEENGDT.VNRNHVTYGVFGGYQITDNLAVELGYDDFGRLKTKSSLDngKHTTHGAHLSLKASYPVLDNLDIYGRVGAALIRSDYKKDMSADGTEHS.HSLKTSPVFAGGLEYAILPELALRVEYQWITKVgnGEKLDYHPDIGSVALGLSYRFGQG.ASPVV.........
386908900.pir    VAGLAASVAQAAPQENTFYAGVKAGQASFHDGI......RDQAPGYHRNSFTYGVFGGYQinLGLAVELGYDDFGRVRYRSDAqlGKHTAHGAHLTLKGSYDvlNGLDFYGRAGVALVRSDIKITKAAydGKRKQHNLHVSPVFAAGLEYAILPELAARLEYQWVNKVgdGNRvDYSPWIGSINAGVSYRFGQGAAPVVAAPEVVSKTF
348001059.pir    AIAGLAATVAQAAPQENTFYAGAKAGWASSHHGLNQFKQK...GVSINRNSEAYGVFGGYQITDNFAVEAGYEYFGRSKAKVNGarFRHTAHGTTLALKASYPVLDNLDVYGRVGAALIRSDYKVGQa..NKPDRYHNLKVSPVFAAGVEYAILPELALRAEYQWVSRVGnaRTIDYSPDIGSVAVGLSYRFGQG.VAPAPAPEVVRKTF
386902687.pir    IASLAATTVAQAAPQENTFYAGAKAGWASYHDGIKYTADEE..GLGFNRNSVTYGVFGGYQinLGLAVELGYDDFGRVKYKEEGNTVaqTAHGAHLSLKGSYEVLPGLDAYARVGAALVRTDVKTYNL...NSERAHDLKVSPVFAAGLEYAILPELALRIEYQWVNRVgdGDRIDYQPSIGAVTAGLSYRFGQG.AAPVV.........
359755603.pir    ALASVAAVVQAAPQANTFYTGAKAGWASVRHGLNQYQYEQDGtgKAKKNSEAYGVFAGYQITDNFAVEAGYEFFGRGKVKEATGEKRITAHGSSLSLKASYPVLHNLDLYARAGALIRVDYKDNTF..GETHKFHDLKVSPVFAAGVEYAILPELALRAEYQWVSRVGNFGkvRySPDIGSVALGLSYRFGQG.AAPVIAPEVVNKVF
322118233.pir    LAVLSAAAVAQAAPQANTFYAGAKAGWASFHDGIEQLDAAKGGKYGINKNSVTYGVFGGYQIllGLATEVGYDYFGRVRGTEKPNDKADkaHGATIALKPSYEVLPDLDVYGKAGIALVNNTYKTFNA.AQEKVkSRRFQSSLILGAGVEYAILPELAARIEYQWLNNAGKagATDYRPDISSVSAGLSYRFGQG.AAPVAAPAIETKNF
386907695.pir    LAVLAASAATAAPQENTFYAGAKAGWANFRDGFTQFDAKSVggFGINKNTVTYGVFGGYQinFGLAAELGYDYFGRSRGNETVDnvKHSAHGAHLSLKPSYEVLPNLDVYSKVGALIRNDYKRYGLtnVYTAKAHTLKPSLTLGAGVEYAILPELAARVEYQYINRVGNLdnTSFSPDLHSVSAGLSYRFGQG.ATPVAPAAPEVMTK
46397593.pir     AVAGFAVASANAAPEANSFYVGAKAGWATFHNDVNQFNSKYALdyGINRNSVTYGVFGGYQIIDNLAVELGYDYFGRVRGnqDSAVKHTAHGTHLSLKPSYEVVSGLDVYGKVGAALVRNDYKNYVD....QEKSHNLKTSLLLGAGVEYAITPALAARLEYQWLSNVGnFnnvTYRPDIHSVSAGLSYRFGQG.AAPVAAPEIVSKNF
260094727.pir    VASLAASVAQAAPQENTFYAGVKAGQGSFHDGV..RAMGKQFSAGHYRNTFTYGVFGGYQinFGLAAELGYDDFGRAKLKTTGktGKHTNHGTHLSLKGSYEVLDGLDVYGRLGVALIRSDYKYYDQGVRQRAkeHSSRVSPVFAAGLEYAVLPELAVRLEYQWVNQIGKLRsyDyNPSVGSINAGISYRFGQGEAPVVAAPEMVSKTF
497813887.pir    ALAVLASAATAAPQENTFYAGAKAGWASFHDGIKQLDAKDGGKYGINKNSVTYGVFGGYQinFGLATEVGYDYYGRVRGhgEKQTFKHSAHGTTISLKPSYEVLPNLDVYGKVGIALVNNRYKTNVVTKAPTkTHRFQSSLILGAGVEYAILPELSARVEYQWLNNAGKagATDYRPDISSVSAGLTYRFGQGAaaPVAAPQVETKNF
322118234.pir    VSGLAASVAQAAPQQNTFYAGAKVGQSSFHGVNQLKFGHDdtYGINRNSVTYGVFGGYQinFGLAVELGYDYYGRVRGkgEFRSFKHSAHGLNFALKPSYEVLPNLDVYGKVGAVVRNDYKFYSatNEPTEKFHKLKASTILGAGVEVILPELAARIEYQYLNKAGNLNKAgyAPDIHSVTVGLSYRFGQGAAPVEAAPEVVTKNF
385693846.pir    LAVLAASAATAAPQENTFYAGAKAGWANFRDgsSQPNAKFNGGYGVNKNTVTYGVFGGYQinFGLAAELGYDYFGRARGnqEKRAAKHAAHGAHLSLKPSYEVLPNLDVYGKVGALIRNDYYVQPTVadSRVKAHNLKPSLTLGAGVEYAILPELAARIEYQYVNKVGNLdnTQySPDLHSVSAGLSYRFGQG.ATPVAPAAPEVVSK
223017549.pir    ........ATAAPQENTFYAGAKAGWASFHDGIKQFDTSDKDGGAFGINRNSVTYGVFGGYQinFGLAAELGYDYFGRVRGngERRAAKHTAHGAHLSLKPSYEVIPNLDVYGKVGALVRNDYtQATVVKADREkAHNLKTSLTLGAGVEYAILPELAARVEYQYLNRVGNLdnTQySPDIHSVSAGLSYRFGQG.AAPVAAPQVETKNF
188474314.pir    VAFSGLTTLVQAAPKDDTWYLGSKLGWSRYASTGMKNDYIGIGDGPTHKNQIGAGAFLGYQANQYLGFELGSDWFGRMPYKGDTKNGAFKAYGVQIGAKLNYPIAEDLDIYTRLGGVVWKAKTKGYTSPEAKRLSENDTGVSPFAAVGLEYALTENWATRFDYQWTRKIGNFDSIGAAPNNGMLSIGVSYRFNQEKP.PEILPEPVTKID
260631448.pir    IAALAAATAAQAAPQANTFYVGTKAGWASFHDGLNQLDSKNGGPVGIKRNSVTYGVFGGYQITDNFAVELGYDDFGRVKgkAENFSAKMTNHGTHLSLKASYPVIADLDAYARVGAALIRSDYKAK...GTKDARAHSLKVSPVFAGGIEYAILPELAARLEYQWVNGVgnGDRLDFTPSVGSVSAGLTYRFGQGavVPEVISKKFTLNS
341950755.pir    VAGLAASVAQAAPQENTFYAGVKAGQASFHDGIRALA..RENSEGYHRNSFTYGVFGGYQinLGLAVELGYDDFGRQAGKtaKHTNHGAHLSLKGSYEVLDGLDVYGKVGAALVRSDYKKV...SDQFREHSLRTSGLFAVGAEYAILPELAVRLEYQWLTRVGKLRtiNYNPWIGSINAGVSYRFGQGSAPVVAAPEMVSKTF
386905832.pir    IAGLAVASVAQAAPQANTYYAGARVGSASFHEDVRQfaSKDFGTDAFKRKFVTYGVFGGYQITDYLAAELGYDDFGRAKynAGTTnFKHTAHGASVSLKASYPiaDGLDAYARVGAALVRSDYKGYHD...TGVREHSLHVSPLYAAGLEYSllPELALRVEYQWLNKVgdGVAYSySPSISSVTAGLSYRFGQGT.APV.APEVVNKTF
299773237.pir    AVKARFASVANAAPQANSFYVGAKAGWATFHNDINQLNSKYKNDagINRNSVTYGVFGGYQIIDNLAVELGYDYFGRVRgkQEFREFKHSAHGTHLSLKPSYEVLSGLDVYGKVGAALIRTDYKDYRYFQ.TREPVKSHNLKTSLIVGAGVEYAILPELAFRVEYQWLSNVGnrATYNYSPDSHSVTAGLSYRFGQGAAPVVAAAPEVVTKNF
52307327.pir     IAGLAAATVAQAAPQENTFYAGARAGWASFHDGVDAFHNSD..GLSAKKNSVTYGVFGGYQinFGLAVELGYDDYGRIRLIedAKRGKFTHTAHGAHLSLKASYPVLDGLDVYARVGAALVRSDYKDY...SIDAAGHSLKVSPVFAAGLEYAllPELALRVEYQWIENVggYNDADFTPDIGAVTFGLSYRFGQG.AAPVEAPEVVNKTF
320577074.pir    ...........................GFYGNGFDNRIANGPTRDDQLGAGAFLGYQANPYLGFEMGYDWLGRMAYKGSVNNGAFKAQGISLAAKLSYPLTDDLDIYTRLGGMVWRADSKGAYDNGNarVSDHDTGVSPLAAVGLEYAWTKNWATRLDYQWVNNIGDAGTVGTRPDNSMLSVGVSYRFGQDEVAP.PAPVVQTKHF
52307325.pir     IAGLAAATAAQAAPQENTFYAGAKAGWASFHDGYTQ...YAEDGVGSHTKSVTYGVFGGYQinLGLAVELGYDDFGRAALRTNgtSAKHTNHGAHLSLKPSYDllSGLDVYGKVGAALVRSDYKVNDGYSYGFNkdHSLKTSLLLGAGLEYAllPELAFRLEYQWLNKVGKLENadYTPEIHSVTAGVSYRFGQGVAAPVAEEVVSKTFT
46397578.pir     AVAGFAVASANAAPEANSFYVGAKAGWATFHDGVNQFNSKYANdyGINRNSVTYGVFGGYQIIDNLAVELGYDYFGRVRGngDRRAFKDTKHGAHISLKPSYEVVSGLDVYGKVGAALVRNDYKDYYA.NGTKDKSHNLKTSLLLGAGVEYAITPALAARLEYQWLSRAGNYGklRYSPDAHSVSAGLTYRFGQGAAPVVAAPEIVTKNF
541485360.pir    IAGLAAATVAQAAPQANTFYTGAKAGWTSSHNGFTQ...FRNAGYDVNRHSVNYGVFGGYQITDNFAAEIGYEYFGGSKLkqDKTETKHTAHGTTLALKGSYPVLADLDVYGRAGAALIRSDYKATLA.GGGKEKAHNFKVSPVLAAGIEYAILPELAARLEYQWTARVGNLgtTEFSPNMGTVALGLSYRFGQG.QAPMPAPEAKVVNK
150840440.pir    IAGLAAASVAQAAPQENTFYAGARVGWSAMHHGVDRIADQFVTDGGLNRNSVTYGVFGGYQinFGLAAELGYDFFGKTKADA...AGYHAAHGASLALKPSYEIYPDLDVYGKVGALVRNMYKatLSASGDAEKFNKTKASLILGAGVEYAILPELAARIEYQWLSKAGNLdnARySPDIHSVTAGLSYRFGQG.AAPVAAPEVVNKTF
317432871.pir    VAGLAAASVAQAAPQENTFYAGVKAGQASFHDGLNRVRAQARKH..DAGYHRNSFTYGVFGGYQinLGLAVELGYDDFGRAKGreGRTVAKHTNHGTHLSLKGSYEVLNGLDVYGKAGVALVRSDYKFYSV..DDHKDGHRVRASGLFAVGAEYAVLPELAVRLEYQWLTRVGknSAINYTPWIGSINAGISYRFGQGEAPVVAAPEMVSKTF
168825896.pir    IALSIAGFVaqAAPQANTFYAGAKAGWASFHDGLNQFENSTEKKG.TVRNSVAYGIFGGYQITDHVAVELGSEYFGQAKGRKDKKEAKHTAQGMQLGLKASYPVLEGLDIYGRVGAALIRSNYLNVKNFENSADGVKNtkVSPVFAAGVEYSllPELALRVEYQWVKGVgdGKRFDYTPSIGAVTLGLSYRFGQG...KPVMAPEVVNKVF
229812260.pir    VAGLATASVAQAAPQENTFYVGVKAGQASFHDV..RANAKENLYAYYRNSFTYGVFGGYQvnLGLALELGYDDFGRLKGRDRLGHgkYTNHGTHLSLKGSYEVLDGLDVYGKVGAALVRSDYKFYANGVKEr1GTHSLRPSVLFAVGAEYAVLLPELAVRLEYQGVTRVGKlsTRDyNPWIGSINAGISYRFGQGEAPVVAAPEMVSKTF
364571900.pir    VLNSCAVSTAFAAADAGTWYGGAKLGWSHYFDASGNGNFNDstDFNTDSDNVGGGVFGGYQINNWLAVEGGYDYLGNMQVNGnvNGGKMKSQGLQLSMKASYAINDAWDIYGRAGAMGYRAEsDVA.....GHNNFETGVRPLAAVGTEYAFTPALAGRLEYQWVSNVGNSNQIGISADSSTVTAGIVYRFGQNEETPVVAPAPTPAPV
499065673.pir    LAGMAFAASTQAAPQDGTWYTGAKLGWSNYFNTESGQDFFGSDgtNFTKNSLGGGVFGGYQVNPWFAVEMGYDYLNKLKSD....NGDLRTQGIQLSGKFSYPVMQDLDLYTRLGAMGYRADMKGN....ANEDYNRHETGIRPLAAVGAEYAFNDNWAGRLEYQYTNKLGNSNQIGLLTFGVSYRFGQteAAPAPVPAPAPEIE
308747049.pir    ILNACTMSTAFAAADAGTWYGGAKFGWSHYSDtdFNDNMRQATDFDTNSDNVGGGIFGGYQINNWLAVEGGYDYLGNMQFNGNNggGKMKSQGLQLSLKASYGLSDSWDLYGRAGAMGYRAETEAA.....GHSTFDTGVRPLAVGTEYAFTPSLAGRLEYQWVSNVGNENQIGMTADASSVTAGIVYRFGQNTPEPVAAPAPAPAPV
1872470.pir      LSVLSATSAFAAPDANTFYLGAKAGWASTHKDINQFDSKYQNDkgIHRNSVTYGVFGGYQIFngLALELGYDYYGQMKGSSQNKVakHTAHGANLSLKPSYEILPNLDVYGKVGMGLVRNDYKFILDlvKDETKLHTLKPSLLGTGVEYAITPELALRTEYQYLNKAGNLYklAYAPDIHSVSVGLSYRFGQgpTPVVVEPEVVSKNF
469488134.pir    AVAGFAVASANAAPEANSFYVGAKAGWATFHGVKQFNSKYvtDLGINRNSVTYGVFGGYQIIDNLAVELGYDYYGRVRGneDKKAAKHTAHGANLALKGNYEVISGLDTYAKVGAALIRNDYKDYLA...QEKAHNLKTSLLLGAGVEYAITPSVGFRVEYQWLSRVGNLDkyNySPDIHSVSAGLTYRFGQGAAPVVAEPEIVTKNF
668959406.pir    VLNACAVSNVFAAADANTWYGGAKMGWSHYFdadFNDNVSNATDFDfDKDNVSGGVFAGYQVLPWLAVEGGYDYLGNMGINGNNggAKMKSQGLQLSLKASYGLTDSWDIYGRAGAMGYRAETS.....GAGRSDFETGVRPLAAVGTEYAFTKNWAGRLEYQWVSNVGNSNQIGISSDVSSVSAGIVYRFGQHDdaPVVAPVAAAPEV
518275551.pir    VSGLLAAGAVQAAAQDNTWYVGGKAGWSNYYGVDHNQYAESigNTSENTDDLGLGLFAGYQLNPNLGFELGYDWLGKYDTTSGTGAneAKSQMIQATMKISFPATNVLDLYGRLGGAYAWTDsEISFANTSETGKKH..GAAFVGALGAEYAIDRDWAARLEYQYTTPLGdadRSGIEMDNGLISFGMLYRFGqeVAAPAPAPAAEPAPV
575563355.pir    IANALTATTAFAAADAGTWYGGKFGWSHYFDTstGANGNKYGDVGFDSDNVGGGVFTGYQITPWLAVEGGYDYLGNMQFNGKNggSQMKSQGLQLSLKASYGLTDDWDLYGRAGAMGYRAEsDIK.....GNNRFDTGVRPVVAVGTEYAFNKNWAGRVEYQWVSNVGNENQIGVSSDVSSVTAGLSYRFGQHdapAPVAAATPAPEPQ
49182348.pir     IVALAVASVAQAAPQPNTFYVGAKAGWASFHDGLNQAKYLEaaTFGFKRNSVTYGVFGGYEQITDNFAVELGYDDFGRAKLrKAKDAAKHTNHGAHLSLKASYPVLDGLDVYARVGAALIRSDYKVYdaKLPQFKRTHSTQVSPVFAGGLEYAFMPELALRVEYQWVNNVGKVKDvgERVDYRPDIGSVTAGFGQSVYVPEVVSKTFTLNS
142850002.pir    VSGLLATGAVQAAAQDNTWYAGGKAGWSKYFDVDQKQVVSDTlnTGTSKDALGLGAFAGYQLNPNLGFEFGYDWLGKYTSSGerVEGEAKSQMIQATMKIGFPVNDALDLYGRIGGGYAWTDsELSARTATDFIkedKHGAAFVGALGAEYAIDRDWAARLEYQYTTPLGdadRSGIEMDNGLLSVGMLYRFGqeVAAPAPAPAVEPAPA
404613379.pir    VSGLLAAGAVQAAAQDNTWYAGGKAGWSKYFDVDQKQVVSStgNTGTSQDALGLGAFAGYQLNPNLAFELGYDWFGKYTSSGekIEGEAKSQMIQATMKISFPVNDVFDLYGRIGGGYAWTNsELSARTATDFIEKskHGAAFVGALGAEYAIDRDWAARLEYQYTTPLGdadRSGIEMDNGLISFGMLYRFGqeVAAPAPAPAAEPAPV
1872471.pir      LAVLSATAVATAAPQADTFYVGAKAGWASFHRGINQFDNkkEGKLNIRRDSVTYGVFAGYQIFngLAAELGYDYYGQLSAknEEYVVRHTAHGVNFSLKPSYELLPDLDAYAKVGIGLARNDYSvnNKTEKVKNRVYDVKPSLLGAGFEYAIIPELALRVEYQYLNRVGNLGKAiySPDMHSVSAGLSYRFGQgePTPVAEPEVVSKNF
648450279.pir    LVSGLLAANAQAAGQDNTWYGGAKLGWSNFYGVDHNQSIKdnaIGEEDKNDVGAGAFLGYQVNQNLGFELGYDWLGKYKyTadIGRDEIKAQLAQLTMKIGMPVSDSLDLYTRLGGAYAWTDsKQF........DNDNGAAFVGALGAEYAFNRDWAARLEYQYTTPLGdlDKTGAELDNGLLAVAVYRFGQ..VAPVVapMPAPAPE
668982613.pir    .......................MGWSYYHDTGFY..NKYASGADTHPHQLGAGAYVGYQANQYLGFEFGYDWLGRMTYRGD.DSAAYKAQGIQLSAKLSYPIMNDLDIYTRLGGAMGWRGDVTAHPAAGGTY.KDHETGVSPLAAVGVEYAITPSLATRLDYQWVNNIGDADSVGTRPDNGMLSLGLAYRFGQTKPAPAVALPVVQTKR
404621651.pir    LVTGLLAANAQAAGQDNTWYGGAKLGWSNFYGID...ESNALTLGNETNDALGAGVFGGYQINQNLGLELGYDYLGNYKYDATTAAGAfKAQLAQLTLKLGFPVTDNLDLYGRVGGGYGWADTKSD........VYKDDSRFVMVGALGAEYAFNLDWAARLEYQYTTPYGSRDDTGLRMDNGLLSLAAVYRFGQVavAPAPAPEPAPAPV
615553950.pir    IGALMATGTVQAAAQDNTWYVGGKAGWSNFYGVDYDDQVKELLDagQDHSDLGLGAFAGYQVNRNVAVELGYDWLGKYQVdeAGLDGEAKAQMIQATMKIGFPATEALDIYGRLGGAYAWTDsISSAWANdaSGGGKYHGAAFVGALGAEYAIDKDWAARLEYQYTTPLGklDESGIELDNGLLSVGMLYRFGQIGDEPvvaPEPAPAPV
651333110.pir    VSGLLATGAVQAAAQDNTWYVGGKAGWSNYYGVDYDQQVKEVLDanEKGNELGLGAFAGYQINQNVAFEFGYDWLGKYKVDqaGLEGEAKAQMLQATMKIGFPATSALDIYGRLGGAYAWTESsLDasVSGSSKGNKYHGAAFVGALGIEYAIDKDWAARLEYQYTTPLGklDESGIELDNGLLSVGMLYRFGqpTPAPVVapAPVVAPK
291421697.pir    VLNACAVTPAFAAADTGTWYGGAKLGWSHYFDASADKDFSDNLDHATdkDNTTGGVFAGYQINSWLAVEGGYDYLGNMQINGNNGVAGAksQGLQLSMKASYGLTDNWDLYGRAGAMAYRAETE.....HAGHSAFETGVRPLAAVGTEYAFNDNWAGRVEYQWVSNVGNSNQIGVSADVSSLTAGVVYRFGQHaaAVVAPVAGPAEP
499067302.pir    LAGMAFAASTQAAPQSGTWYTGAKLGWSQYYSLS...DDSNMTVTDTHKNNVGGGIFGGYQFNPWLAAELGYDYYMGNMRVHTLHDggRFSSQGIQLTGKLSYPlnNSMDIYTRLGIMGYRAD.MRNLDNTHFDHNGDKYGVAPVAAAVGAEYAFNDHWSGRMEYQWVGKMGKESDTGMKADNGMLSLGVAYHFG..EAAPVPAPIPAPAPE
516391880.pir    VSGLLVSGAVQAAAQDNTWYVGGKAGWSKFFDVDQKQVVSDTlnTSTSKDTLGLGAFAGYQLNPNLGFEFGYDWFGKYTSSGerVEGEAKSQMIQTTMKIGFPVNDALDLYGRIGGGYAWTNsELSAQTATDFIKdnKHGAVFVGALGAEYAIDRDWAARLEYQYTTPLGdlDRSGIEMDNGLLAFAMLYRFGqeVAAPVPAPAAAPAPA
469488135.pir    AVAGFAVASANAAPEANSVYVGAKAGWASFHDGISQIDHKNGGKYGINKNSVTYGAFVGYQIIDNLAAEVGYEYFGRVRGRglSKKTFRHSAHGTTIALKPSYEVISGLDTYAKAGIALVNNSYKTVNVDTKVPTKTSRFQSSLILGAGVEYAILPELAARVEYQWLNNAGKASysDYRPDISSVSAGLTYRFGQG.AAPVAAPEIVTKNF
549073716.pir    VVLAGFATVVQATPRDNTWYNGVAMGWSNYHDINFYGNHYRPNQGPSTKCHPGLRGFLGYQANPHLGFELGYDWLGRMANKGVIVNGSKFAHGLQLTTKLSRRISPHADLYARLGFMLWTADAMQHHS.YMEFVNNRDNGIAPLAAIGVEYAITKNFATRLDYQWVNNIGDAYTIGMRPDNGLLSIGVSYRFFQNQPtpAVSPTIISSTP
651305662.pir    IGALMATGTVQAAAQDNTWYVGGKAGWSNYYGVDYNAQVEELFDanENSDDLGLGLFAGYQVNRNVAVELGYDWLGKYQVddFALDGEAKAQMVQATMKIILPATSALDIYGRLGGAYAWTESsLNVRASDGTSSLSEsgAAFVGALGVEYAIDKDWAARLEYQYTTPLGklDESGIEMDNGLLSVGMLYRFGqePVAPTPAPEPAPAPA
440047182.pir    IANAFTASTAFAAADAGTWYGGTKFGWSHYFDsgQVDNAHNSDQFDFDrDNVGGGVFTGYQITPWLAVEGGYDYLGNMQIKGNHggAKMKSQGLQMSLKASYGLTNKWDIYGRAGAMGYRAEsDIG......GHNRFETGIRPLAAVGTEYAFNKNWAGRLEYQWVSNVGNANQIGVSSDVSSVTAGISYRFGQHDDEPvvaPLPAPEPQ
```

```
363988451.pir  LVITGFATLAQAAPKNNTWYIGSKLGWSEDHDISLFSTGYLnnNNSTHKNKLGAGAFFGYQANKYLGFEIEYDWLGRTMYKGSLYNGAIKLQGVQLAAKLSYPIIDNLDIYTRFGGMVWHQHSKYNYE.NAPYLSSGDTGVIPLTAIGVEYALTKNLATRLDYQFIRNINNngIISTDLDNTMLSIGISYHFSQKNTINPIVQTPENITI
213950522.pir  ................DSFYVGAKAGWATFHNDINQIDSKYANDagISRNSVTYGVFGGYQIIDNLAVELGYDYFGRVRgkQEFRAFKHSAHGTHLSLKPSYEVLNGLDVYGKVGAALVRNDYKRYSQTAgqTQKAHNLKTSLVLGAGVEYAILPELAFRVEYQWLSNVGnrATYNYSPDSHSVTAGISYRFGQGAAPVAAAPEVVTKNF
651306045.pir  LVSGLLAANAQAAGQDNTWYGGAKLGWSNFYGVD...ESNAIAITDKDDDEVGAGAFAGYQINQNLGVELGYDYLGKLKYSGTRNGAAfqAQLAQLTLKLGFPVADNLDLYGRVGGGYGWTDSDQL........ENDSRFTFVGALGAEYALNLDWAARLEYQYSTPYGSRDDTGLRMDNGLLSLAAVYRFGQVAPapVAPAPAPEPV
615550405.pir  LVSGLLAANAQAAGQDNTWYGGAKLGWSNFYGVD...ESNAITTTDKDDDEVGAGAFAGYQINQNLGIELGYDYLGKLKYNGTRNAAAfeAQLAQLTLKLGFPVADNLDLYGRLGGGYGWTSSDEL........DSDSRAVFVGALGAEYAFNLDWAARLEYQYSTPYGSREDTGLRMDNGLLSLAAVYRFGQVPPapVAAPAPAPEPV
485943435.pir  ....MLVANAQAAGQDNTWYAGAKLGWSNFYGVDHNDAIKDAAdtDESNDALGAGAFAGYQINQNVGVELGYDWLGEYKYDVtkGDDKVTAQMVQLTMKIGFPVADSLDLYGRLGGAYAWADS........RELDNDDGAAFVGALGAEYAIDRNWATRLEYQYTTPLGdlDHTGVELDNGLLSIGMLYRFGQEAPEPVVVPPMPEPT
668967619.pir  VINACAVTNANAAADANTWYSGAKLGWSHYSDISGNNNFNGNQNndTDSDNVGGGVFWGYQILPWLAVESGYDYLGNMQVQGNNgnGKMKAQGLQFSMKASYSFNENWDIYGRAGMGYRAETDQA.....EHSNFETGVRPLAAIGTEYEFNDNWAGRLEYQWVSNVGNSNQIGMSADINTVTAGVVYRFGQNSAAPVAETAPVVEGP
320577124.pir  ..............................GFYGNGFDNRIGDGPTRDNQLGAGAFVGHQANQYLGFELGYDWLGRIAYKGSMNNGSFKAQGISLAAKLNYPLTDDLDIYTRLGGMVWRADSKGAYDNGNarISDHDTSISPMAAVGLEYSWTKNWATRLDYQWVNNIGDVGTVGTRPDNSVLSVGMSYRFGQNDEVTAPAPVVQAKHF
518678632.pir  VSGLLVSGAVQAAAQDNTWYVGGKAGWSNFYGVDYNEQVKAVVDldKKHSDLGVGAFVGYQLNPNLGFELGYDWLGKYKLdsAGLNGEAKAQMVQATMKMSLPATPVLDVYGRLGGAYAWTESAYSGWYRDSANPANDlgAAFVGALGIEYAIDRDWAARLEYQYTTPLGKTseSGIEMDNGLLAFAMLYRFGqeVVAPAPAPAATPAPA
320577116.pir  ..................................................GAGAFLGYQANPYLGFGLGYDWLGRMAYKGSVNNGAFKAQGISLAAKLSYPLTDDLDIYTRLGGMVWRADSKGAYDNGNarVSDHDTGVSPLAAVGLEYAWTKNWATRLDYQWVNNIGDAGTVGTRPDNSMLSVGVSYRFGQDEVAP.PAPTPAPAPV
667705883.pir  AVASLTANANAAYEENTFYVGGKLGWSDFydANKVRAYESASIKTSDHSNIAGGGFIGFQANPYLAFELGYDWLGSAKYKRSagNSKVSAQGVSLTGKLSYPLSdDLDVYGRLGAMVTIAKWkgGYVKEDFGRGGTKTDVSPVFALGMDYRLNDDLSTRLEYQYVNHIHT..KTDASPDAGILSLGITYRLGSPVAAPAPVVIQTQERV
530712460.pir  LVSGLLAANAHAAGQDNTWYGGAKLGWSNFYGVDEHNTITSLND...DNDEVGAGAFAGYQVNQNLGIELGYDYLGNYKYNGTRnnDKVEAQLAQLTLKLGFPVAENLDLYGRVGGGYGWTSSDEL........DNDGRFNFVGALGAEYAFNRDWAARLEYQYSSPYGDREETGLRMDNGLLAVAVVYRFGQ..VAPVVapAPTPAPE
435333715.pir  ..................................................AGAFGGYQVNPYVGFEMGYDWLGRMPYKGDNINGAYKAQGVQLTAKLGYPITDDLDVYTRLGGMVWRADTKSNVP.GAPSTKDHDTGVSPVFAGGIEYAITPEIATRLEYQWTNNIGDANTIGTRPDNGLLSGVSYRFGQQEAAPVVAPAPAPAPE
637111975.pir  AVASLTATAANAAYEDNTFYTGAKLGWSELYRLDagFDKDEVGIKTSNRSNVAGGAFLGFQANPYLAFELGYDWLGSAKYktKAGNSKLSAQGVSLTGKLSYPIIGDLDIYGRAGGMVTIAKWKNSGDTRVegHGGTKTDVSPVYALGLDYRLDEDFSTRLEYQYVQHIHTKSK..ATPDAGTVSLGITYRLGSPapAPVELKTEVNRYV
664675997.pir  AVASLTATAANAAYEDNTFYTGAKLGWSELydAKKVRAFDDVSIKTSNRSNVAGGAFLGFQANPYLAFELGYDWLGSAKYkyDLGKSKVSAQGVSLTGKLSYPLSdDLDIYGRAGGMVTIAKWKNGGLTKDLYGkgTKTDLSPVYALGLDYRLNEDFSTRLEYQYVQHIHT..KTDASPDAGTVSLGITYRLGSPAVAAPVEIKTEVNRY
480572252.pir  MIAGSFAATVQAAGQDNTWYAGGKLGWSNFYGVDYDPAFTGAAITDESKDDVGLGALVGYQLNRNVGFELGYDWLGKYKYtgLSGTDEVSAQLAQLSMKLGIPVSDAWDIYARLGGAYVWADSDQF........ENDNGGAFLGALGAEYAINRDWAARFEYQYTTPIgaIRDTGVSMDNGLLSLAMIYRFGQVAAvpVPAPEPQMV
597809980.pir  AVASLTATAANAAYEDNTFYTGAKLGWSELydANKIRENSNVGVSTSDRSNIGGGAFLGFQANPYLAFELGYDWLGSAKYSKTDnhSKVSAQGVSLTGKLSYPLSdDLDIYGRAGGMVTIAKWKNSGLveRHGHGGTKTDLSPVYALGLDYRFDEDFSTRLEYQYVQHIHTKSK..ASPDAGLLTLGITYRLGSPVVTPPEVEVKTQENR
480570607.pir  LLGLLLTGASQAAPDDHSWYLGAKAGWSNYFDADTQRLVEEIQGRLQSrdGLGLGLFAGYQLNRNVGFELGYDWLGRYKTRGELDggEAKSQMVQATMKIGGEVNEGLDFYTRMGGAYAWSDATAEAHYGARSVSdkEHSAAFVAALGAEYALSPNWALRLEYQYTTPIGDAsrTAIEFDNGLLALGLIYRIGQWQDAAPVVSSPAEIPP
664678209.pir  AVASLSATAANAAYEDNTFYIGGKLGWSELYRLDANkdKDEAGIKTSNRSNVAGGAFLGFQANPYLAFELGYDWLGSAKYkgIGNSKVSAQGVSLTGKLSYPLSdDLDIYGRAGGMITISKWKNSGYVKTNHRLDhkTDVSPVFALGLDYRLNEDFSTRLEYQYVQHIHT..QTNASPNAGAVTLGVTYRLNSPSVAVPVEIKSEVNRY
550214112.pir  MVNAFTATSAFAAADAGTWYGGAKFGWSHYFDTNMGSKAFDNNNSdfDHDNVGGGVYTGYQITPWLAVEGGYDYLGNMQIKGQHGAgamKTQGLQMSLKASYALTNDWDIYGRAGMGYRAESDVS.....GHNRFETGVRPLAAVGTEYAFNKNWAGRLEYQWVSNVGNANQIGVSSDLSSVTAGLSYRFGQYD............
226344125.pir  ..............................DTGFINNNGPTHENQLGAGAFGGYQVNPYVGFEMGYDWLGRMPYKGSVENGAYKAQGVQLTAKLGYPITDDLDIYTRLGGMVWRADTKSN.....VYGKNHDTGVSPVFAGGVEYAITPEIATRLEYQWTNNIGDAHTIGTRPDNGMLSLGVSYRFGQ................
392326470.pir  LANAFTATTAFAAADAGTWYSGAKFGWSHYFDASTGSKASDNNSGsiDHDNVGGGVYAGYQMTPWLALEGGYDYLGNMQMKGHHSAgamKSQGLQMSLKASYAVTDRWDIYGRAGMAYRAESDVS.....GHNRFDTGVRPLAAVGTEYAFNKNLAGRLEYQWVSNVGNANQIGVSSDVSSVTAGLTWRFGQHD............
480569860.pir  VPALLVHGMAQAAV.ENTWYAGGKMGWSNYydPGIEQLFERSGKSNTNSDDLGVGGFIGYQLNANLGFELGYDWLGTYqgVGYSLHGEGQAQMLQATMRISGAASDRLDIYGRLGGAYAWTDssAVSSLGSANAQASEHSPAFVGALGVEYTIDANWAARLEYQYTTPLgdLNKSGIEMDNGLLAFGMLYRFGPqeVAPPVAPAPATVVI
257451405.pir  ........ATAAPQENTFYAGAKAGWASFHDGIKQLDATHYNnyGINKNSVTYGAFAGYQIlfGLATEVGYEQFGRVRGHQNTQAgkHSAHGTTIALKPSYEVLPDLDVYGKVGIALVNNRYKTVDVQTKEPTKTSRFQSSLILGVGVEYAITPELAARVEYQWLNNAgdAQVTDYRPDISSVSAGLSYRFGQ..............
485941458.pir  IGATCTSGLAQSQALDNSWYLGAKAGWSHYFGADFEPDIDEAfgAVEQGDDLGLGLFVGYQLNPNLGFELGYDHLGKYRLSgaRLAGEARSQMIQASMRIGFPATQSLDLYGRLGGAYAWTESDLSAALTTSSRQFNESgAAFVGALGAEYAFNSSWALRLEYQYSTPLGStgESGIELDNGLLALGLVYRFGQEEEVVAASPAPAAVV
537117075.pir  ..................YGGAKLGWSNFYGVDHNQDIKDnaISEEDKNDVGAGAFVGYQVNQNLGFELGYDWLGKYKYTadIGRDEIKAQLAQLTMKIGMPVSDSLDLYTRLGGAYAWTDSKQF........DNDNGAAFVGALGAEYAFNRDWAARLEYQYTTPLGdlDKTGAELDNGLLAVAVVYRFGQVaaPMPAPAPEPVMV
639154778.pir  VAIASLTSAAHAAQPENTFYAGGKVGWSSFHHAG......LGDDFKKPDDQVGGGLFVGYQANPYMAIELGYDYLGNAKFKeDKDDYKFAVHGASVTGKLSYPLTDDLDFYARAGAFIHHTKFKGN.....EYDKSNVSVSPVFGGGFDYKLTDNFTARVDYQWVRNIGNNGPKDFHPDNGLLSLGLIYNLGGPTTKVVAKPAELEVKE
459649431.pir  VAGLAASVAQAAPQENTFYAGVKAGQGSFHDGINNNGAisSTNYGYRRNTFTVYGFGGYQinFGLAAELGYDNFGRKLREAGkkVKHTNHGAHLSLKGSYEVLDGLDVYGKAGVALVRSDYKFYNDANGTRdsRHSVRASGLFAVGAEYAVLPELAVRLEYQWLTR......................................
320577090.pir  ..................................................NPYLGFELGYDWLGRMAYKGSVNNGAFKAQGISLAAKLSYPLTDDLDIYTRLGGMVWRADSKGAYDNGNarVSDHDTGVSPLAAVGLEYAWTKNWATRLDYQWVNNIGDAGTVGTRPDNSMLSVGVSYRFGQDEVAP.PAPAPGCSDQ
410370151.pir  IAHAFTATAAFAAAETGTWYAGSKFGWSHYGDSS......TDRQVDVDRDNVGGGVFTGYQINPWLAVEGGYDYLGNMGINggASGSQMKSQGLQLSLKASYSLTSDWDLYGRAGLMGYRAESDVN.....GKNRFDTGVRPVLAFGTEYAFNDRWSGRLEYQWVNNVGNENQIGVSSDVSSMMAGISYRFGQ..............
144983137.pir  ...........................MAQLKNNLTLTSYGYRRNTFTYGVFGGYQinFGLAAELGYDNFGRVKLRLAGkqAKHTNHGAHLSLKGSYEVLDGLDVYGKAGVALVRSDYKFYEDANGTRdgRHTARASGLFAVGAEYAVLPELAVRLEYQWLTRVGknTAINYNPWIGSINAGISYRFGQGAAPVVAAPEVVSKTF
541135496.pir  VAGLAASVAQAAPQENTFYAGVKAGQASFHDGLRALA..REKNVGYHRNSFTYGVFGGYQinLGLAVELGYDDFGRAKGREKGKTVahTNHGAHLSLKGSYEVLEGLDVYGKAGVALVRSDYKLYDKNSSNNLkkhRARASGLFAVGAEYAVLPELAVRVEYQWLTR.......................................
541135500.pir  VAGLAASVAQAAPQENTFYAGVKAGQGSFHDGV..RAMGKLFGSGHYRNTFTYGVFGGYQinFGLAAELGYDDFGRAKLKNTGktGKHTNHGTHLSLKGSYEVLDGLDVYGKAGVALIRSDYKKYDQGVRQRAkeHSLRVSPVFAAGLEYAVLPELAVRVEYQWLTR.......................................
328492295.pir  LPLALLISGAVNAATENPFYAGVRAGGVHYADFDSKNYAAGTYGNpiDRDDFAAGLFMGYNVTPWFAIEGGYTWLGEVTLKGDkdQGKVEQQAIDLVGKFTYQATDKVDLYTKVGGAWQFTRSKGLP...GQTTTDRDDSLIATAGLGAEYHFTDNLSARAEYQYYHNMEAKPTGTDTKYndANYYGVSLVYGWGAPAPVVAPVVEEVVE
151567739.pir  ..........MPKDNTWYTGAKLGWSQYS.............RENQLGAGAFGGYQVNPYVGFEMGYDWLGRMPR........KAQGVQLTAKLGYPktDDLDIYTRLGGMVWRADTSKDD.GNGYISAAEASVSPVFAGGVEYVitPEIATRLEYQWTNNA..........SDNGMLSLGVSYRFGQGEAA..........
90441030.pir   LPLALLISGAVNAATVNPFYAGVRAGGVHYSDFDsaNGTYGSGAEGVDRDDFAAGVFMGYNVTPWFAIEGGYTWLGEVTSKYDedSGKIEQQAIDLVGKFTYQATDKVDLYTKVGGAWQFTRAKGLP...GQTATDRDDSLIATAGLGAEYHFTDNLSARAEYQYYHNMETKPTGTDTKIndANYYGVSLVYGWGAPAPVVAPVVEEVVE
239950061.pir  ......................................NGYGINRNSVTYGVFGGYQIIDNLAVELGYDYFGRVRGkeeFRAFKHSAHGTHLSLKPSYEVLNGLDVYGKVGAALVRNDYKFY...DTEDKAHNLKTSLLLGAGVEYAILPELAFRVEYQWLSRVGNANksTYSPDAHSVSAGISYRFGQGAaaPAAAPEVVTKNF
89048248.pir   LPLALLISGAVNAATVNPFYAGVRAGGVHYSDFDSKNYANGtnRNAIDRDDFAAGLFMGYNVTPWFAIEGGYTWLGEATLKGDenLGKVEQQAIDLVGKFTYQATDKVDLYTKVGGAWQFTRAKVE.....GVGSDRDDSLIATAGLGAEYHFTDNLSARAEYQYYHNMETKPTGTDTKFndANYYGVSLVYGWGAPAPVVAPVVEEVVE
326346124.pir  ............................................................MGYDWLGRMPYKGSVENGAYKAQGVQLTAKLGYPITDDLDIYTRLGGMVWRADTKSN.....VYGKNHDTGVSPVFAGGVEYAITPEIATRLEYQWTNNIGDAHTIGTRPDNGMLSLGVSYRFGQGEAAPVVAPAPAPAPE
363642983.pir  VALAGFATVAQAAPKDNTWYTGAKLGWSQFHDIG...NNRIDNNGPTHESQLGAGAFGGYQVNPYVGFEMGYDWLGRMPYKGSEQNGAFKAQGVQLTAKLGYPITDDLDIYTRLGGMVWRADAKNN.....TGFKDHDTGVSPVFAG..........................................
90324929.pir   LPLALLISGVVNAATENPWYAGARVGGTSYSDFS.....GLGPAQIDKEDWGGGVFLGYNIVPWFAVEGGYTYLGEADIKGT..NTSIEQQGIDLVGKFTWNVTNSIDLFAKAGGFYYFADSSG......PIDVSDDGIIGTAGVGAEYFFNKNLSARLEYQYYHDVekDSGIDASWDTHFYGISLVYGWGAPAPIAVVEPAPEPAPV
441428083.pir  LPVVLLVSGVSHAATDNPWYAGARVGGTNFDSFDGALDGQE...GAFERDDWGGGAFIGYNYNSWFAVEGGYTYLGQADAKQ..GSGGFEVHGLDIVGKFTWSVAPSIDIFGKAGGYLYDVDNSVN........SKDDSGLSGTIGVGAEYFFNDDLSARLEYQYYNQVGDDE..PGESDVHFYGLSLVYHWGAEPAvpVEPEPVPEPE
358410520.pir  IPLAVCLSDAVHAATDNPWYAGARVGVSHFSDFDKAVTD....VTGVDRDNFAGGVFAGYQVTPWFAVEFGYTHLGNAKLDD..VNGSYEAQGADIVGKFTWSISPAITDSFDIFAKAGTFYYFNNNLSTRVEYQYYNDVGGV......DIHFGGVGLVYHWGA..PAPVVVEPVYVDQV
46915744.pir   LPLALLISGVVNAATENPWYAGARVGGTSYSGLS.....IDSRSSDIDKEDWGGGAFLGYNIVPWFAVEGGYTYLGEMDFKAD..DASIEQQGLDLVGKFTWNVTNSIDLFAKAGGFYFFTDGSGA......ISGSDDGIIGTAGVGAEYFFNKNLSARLEYQYYN...DLELKDASWDTHFYGISLVYGWGAPVPVAVVEPEPAPV
387936435.pir  LMALAAAASSVVNADDAGWYLGGNIGQSNADIDNDRITENLLDGgdSDDRDTGYKLFSGYQFNRNFALEAGYFDLGEFSYRATtlDGEMKLRGFNLDLLGFIPSEKFSAFGRVGVNYAEAKdsGTGAVNVLSPRAEERDTNLKLGAGVQYAFTDALAMRVETERY.RINDA..VGNKGDIDMVSVGLLYRFGATSaaVVQPPEPAPAV
90441027.pir   LPLALVVSTVVSAATVNPFYVGARLGGAHYSN..FQQNYQDITGINLNKDDVAAGVFAGYNFTPWFAVETGYTWLGEAKF....ENNKIKQNAIDLAAKMTWNLNDQWGVFGKLGGSYMLADFHGY.....GIKDLDDHLLGTAGVGVEYKLTNHLSTRLEYQLYHDIRsdTNLAYNFDTQFYGLSLVYSWGAPAPVVDTAPPVQAA
268161022.pir  PLALLISGVANAAA.DNPWYAGFRVGATHYNDIS...VDGVDSNSAFDRDDMGGGLFAGYNVTPWFAVEVGYTWLGRAEFNNDYK.MRVDQQAIDLVGKFTWHATDYMGLYAKLGGAYYFSEAKGFGA.....EKYKDDGVVGTAGAGLEFFLDDHLSARLEYQYYHDVekDKDVRANWDTHFYGLSLVYSWGAPeaEPVYVDQVSVATL
14331084.pir   LVLAGQASMALASNQVGEFYVGGKAGLTQFSDEC......GANSLDCEEQSEGFGVYGGYQALDWLAVEVGYSDLGSKSKAdgEVAHYDATVQGIELGLKADYALTERAGLFAKAGTLLWQVEKSGSEPGLGSVNQEED.GSSLMLGAGVEYRLTEHWNTRLEYQYFNEVGGEKTGG..SDIHFIGVGLDYRFGGAAQTAMSEPMDVRPVD
90440061.pir   IPLALFVSNSVHASTDNPWYVGARAGASHITNMD....DAVTSVTKLEDTNFAGGVFGYQVTPWFALETGYTHLGNAEL..DNKQGSYEAQGADLVTKFSYAISDSVDIFAKAGAFYYDWNAIGS......GVSNSDNGWSPTAGAGIEYFINKNVSTRLEYQYYNDVAG.......PDIHFAGLGLAFHWGA..PAPVVVEEPIYVNQ
328490735.pir  PLVVTSLSSIAHASTDNPWYVGARAGASFLTNMD....DAVTSVTEIQNTNFAGGVFAGYQVTPWFALETGYTHLGNA..KLDNGQGSYEAQGADLVTKFSFSATDSIDIFAKAGAFYYDWNAIGS......GVTNSDNGWSPTAGAGLEYFINKNLSTRLEYQYYNDVGG.......PDIHFAGLGLAYHWGA..AAPIVEPVYVDQVT
541135494.pir  VAGLAASVAQAAPQENTFYAGVKAGQASFHDGLRALA..REKNVGYHRNSFTYGVFGGYQinLGLAVELGYDDFGRAKGREKGKTVahTNHGAHLSLKGSYEVLEGLDVYGKAGVALVRSDYKLYnsTLKNLGEHHRARASGLFAVGAEYAVLPELAVNTNG...............................
404623132.pir  LALAAAGTTAQAA...DDWYTGVGAGWAYAHNLD......DVQGLDTDKDATALNLFGGYNFNDYYAAELGYLYAGKAGVDGT...DFKTQGATLSGIARLPLSDIFSVFAEGGAYFNHVNGNGN..........SDNGTAPLAGLGLTAKLSDLIDVQARYRYLWNLGDEQKTW.ESDMSVATLELVMHPNrsYVAPVAPAPEPVPE
119863066.pir  PLALLVSGAATAaaTENPWYTGARLGGAHYSD....FAENIWTPDNQDQNDLAGGIFLGYNYKPWLAVETGYTYLGEAQF...GNNRSVEQQGIDLVGKFTWQATDSVDLFAKAGGFYFADGK......GEFNTSHDRGLIPTTGAGVEYFFNKNVSARLEYQYYHDIdkDSGVDTSWNTHFYGVSLVYSWGAPAPITMTAMEPAVAEP
89345405.pir   LIALAILAGPFALADDLGWYAGANVGRSRSATIDDARITSGLLGSGLtdNRSSGYKIFGGYQFNRNFAVEGGYFDLGNFGFNANTVsGNIKLGKLNLDLVGTLPISEKFSVLGRIGVNYADADsgTGAVNVLPSPSKRETNLKVGVGLQYAFTDALAMRAEIERY.RINDA..VGNKGDIDLVSLGLIYRFGAKTPTPVPVAYAPV
485941727.pir  IAIAMATLGATAANAADDVYFGAGVGAAHFNGLNN.....INGVDDGTEDAAAANAFVGYNFTENFGTELGYQYAGR....GNTEGSRYENQGATLSGIARLPVAKDLSIFAEGGAYWAHTD..........GLETSDTRVSPLAGLGLTYKLNDLDLQARYRYMWNVSDLStdNYKSNQSVATMEVVYHPFRtvAAPVVEEVVAPVIV
328492298.pir  LPLALIISTAASAATMNPFYAGVRVGGAHYSN..FQQNYQDVTGINLNKDDVAAGLFAGYNITPWFAVETGYTWLGEAKFQHN....KIKQHAFDLTGKATWHLNDKWGVFGKLGGSYMLTDFHGY.....GIKDIDEHLVGTVGAGVEYQLTDHLSTRLEYQLYHDLKSNPRYtyNFDTQFYGLSLVYSWGATKPA.VVEPVVNTAPP
320577092.pir  ...............................................................LGRMAYKGSVNNGAFKAQGISLAAKLSYPLTDDLDIYTRLGGMVWRADSKGAYDNGNarVSDHDTGVSPLTAVGLEYAWTKNWATRLDYQWVNNIGDAGTVGTRPDNSMLSVGVSYRFGQDEVAP.PAPAPVVQTK
480569187.pir  IAIALAAMGTTAA.QANDWYTGVGTGWADMRDLG.......KFGWDADEDATAINLFGGYNFNDNFGAELGYLYAG....KGGVDGVDFKTQGATLTGIGRLPLGNVFSLFAEGGAYFYHANGNDN..........TDNGAAPLAGLGVTAKLSDLVDLQARYRYLWNVGDEPKTW.ETDVGVASLELVLHPNrsYVAPVAApePAPEPV
369844096.pir  TDASVMASSAFPAYQNGHFYLGARMGWAAYQDAC.....GSSANDCNDDTFGYGAYGGYQFNHWLALEGGYTDLGVTAYGEPDASYPEGDLAAEVTGGEVAVKLSYPLTERVDVFTRLGAYFGDSYEKSASM....TTDSMNSHEWNTLASLGMSYRLSQRWSGEYQFIDGIGEDNDIG.QADLHFTSIGLTYHFGQSEPEvvvEPVVEPE
569548869.pir  IAIAMAMGATAAHAADDIYFGAGVGATHFNGLN.....KIEGVNAGEEDAAAANAFVGYNFNEYFGSELGYQYTGRGNTDGN....RYESQGATLSGIARLPLGNDFSLFGEAGAYWGHTDGMGT..........SDTKVSPLAGAGVTYKVNddLQARYRYMWDVADLHADNVRYKSNQSVATLEAVYHPFRtaPAPVVEEAPAPAPQ
208009243.pir  SLGIACNASANTNTEDETIYIGGKIGWTHFSNGC......ESQNLDCDKEAMGGGVFLGYKLNEWLAIEGGYDYLGKAKAIYPSLDdsAKFQGIELGLKADYYLNDNWNVFGKVGGLGWRADKKGEEL..NYDVEGRDQDISLMLGAGLEYRLTKQLKTRFEYQWFDSVGGENTGG..SNINFITIGLTYSFGSKEEVVVIAEPDPPVRI
260839290.pir  AAIISTTLLFASSAAVAEVYVGGKIGKSWLDDAC......QSGQPCDKDDTTLGAFVGYEANKWLSLEAGYDYLGKFTAAGL...ADEKVKAITLAPKLSLPLTEGIALYGKVGGAFVDYGSK...........DDYSYLGAAGLEFNTNHNVTMRLEYQ...NITDINNDIVRASGESATLGVYKFGGSePAPVVEQRPapAPV
239950057.pir  ...........................................ARXATSANGYXXXRNSVXYGVFGXYQIIXXLAVELGYDYFGRVRgkXXFRAFKHSAHGTHLSLKPSYEVLNXLNVYGKVGAALVRNDYKRYFQ.TXEPVKSHNLKTSLIVGAGVEYAILPELAFRVEYQWLSNVGnrATYNYSPDSHSVTAGISYRFGQGAXPVAAAPEVVTKNF
341632425.pir  AAIISATLLFASSAAVAEVYVGGKVGKSWLEDAC......QAGQSCEDDDQVVGAFLGYQANKWLSLEAGYDYLGKFTAAGLN...DEKVQAVTLAPKLSIPLTEGIALYGKVGGAYVDYGSK............DDYSYLGAAGLEFNTNHNVTMRLEYQ...NLTDINNDIVRARAETATLGIAYKFGGSepAPVVEQRPapAPV
569548871.pir  IALAIAAMGTSTAQAADDWYTGMGAGWAYGHDLD......SFGLDADKDATAISLFGGYNFNDYYAAELGYLYAG....KGGVDGVDFKTQGATLSALGRLPLGDIFSVFAEGGAYFNHVNGNG..........ESDNGTSPLAGLGLTAKVSDLLDLQARYRYIWDLGDEQKTW.KTDMSVATLELVMHPNRKavAPVAAPAPEPVPE
404632559.pir  IALALAATGAQAAP.DNDWYAGVGGGWAIAHDLG.......DFGKDASKDATALSLFGGYNFTENLGGELGYLSTGNWDVQGN....DFSSQGATLSVIGRLPLGDIFSVFAEGGGYLYHVKS..........VNGGDNNLAPLAGLGLTAKLHDWVDIQARYRYLVRVGDdgTQRWVSDISTATLELVIHPNRsaPAPMVVPPPPAPE
404614684.pir  IAIAMALGATAAHAADDIYFGAGVGAAHFNGL....NKIDGGGYAGTEDAAAANAFVGYNFTENFGTEFGYQYAGR....GNTEGLRYENQGATLSGIARMPVGNDFSLFAEGGAYWAHTDGLGI..........SDTKVSPLAGAGVTYKVSDALDLQARYRYMWDVADLNVDgyKSNQSVATLEAVYHprTSYVAPAPVVVEEAPA
U3BDN2.pir     AAVISASLLMASTSAMAQVYVGAKVGKTWLDDAC......ISGQPCDDEDSTLGAFAGYEWNKYFALEAGYDYLGEFNTGTAGLNsdDVSAITLAPKLSLPLTDDISLYGKFGGAYVDYGSK............NDWSYLGAAGLEIDSHQNVTVRLEYQT...ITDMNNDRVRAAGNSATLGVVYKFGGQEAvpvEEEPVVAEP
404632560.pir  IALAIAAMGTTTAQASNDWYTGMGAGWAYGHDLD......DFGKDADKDATALSLFGGYNFNENFGAELGYLYAG....KGGVDGVDFKTQGATLSGLARLPLGDIFSVFAEGGAYFNHVNGNGS..........SDNGTAPLAGLGVTAKLSDLLDLQARYRYMWNLGDEQKTW.ETDMSVATLELVMHPNRTtvAPVAPAPEPVPE
651332619.pir  IAIAMAMGATAAHAAEDIYFGAGVGAAHFNGLN....KIEGVNNGEEDAAAANAFVGYNFNEYFGTELGYQYAGR....GNTEGLRYENQGATLSGIARLPLGNDFSLFAEGGAYWAHTDGLGI..........SDTKVSPLAGAGVTYKVNDALDLQARYRYMWDVADLNVEgyKSNQSVATLEAVYHprTSYVAPAPAPVVEEAPA
530714709.pir  IAIAMAMGATAAHAADDIYFGAGAGAAHFNGLN.....KIEGVKNGEEAAAANAFVGYNFNEYFGSELGYQYAGRGNTDGN....RYENQGATLSGIARLPLGGDFSAFAEGGAYWAHTDGMGT..........SDTKVSPLAGLGVTYQVNddLQARYRYMWDVADLHADNVRYKSNQSVATLEAVYHprSSYVAPAPAPAPVVEE
480569188.pir  IAIALAAVTATAAHAADDIYFGAGVGAAHFNGL....NKIGGGGYEGTEDAAAANAFMGYNVTENFGAELGYQYAGRGNTFGE....RYENQGATLSGIARLPLGGDVSAFAEGGAYWAHTD..........GLTVSDTKVSPLAGLGLTYKVSDALDLQARYRYMWDVADLHVDGrsNQSVATLEAVYHPFRtaPAPVVEEAPAPAPA
485941726.pir  IALALAAAGSTAaaADNDWYTGIAAGWAYTHDLEDFGK.....NADVDKDATALSLFGGYNFTENFGAELGYLYAGKAGIDG....VDFSSKGATLSGIGRLPLGDIFSVFAEGGAYVFHANGNNQ..........SDNGIAPLAGLGLTAKLSDLIDVQARYRYMWDVGDENKTGNNmdMSVATLELVLHPNrsYVAPVAPVVAQPEP
569548872.pir  IALALATSGAQAAP.AHDWYIGAGGGWAIAHDIN.......DFSQDVDKDATAIDAFVGYNFTENLGAELGYLSTGDWDIAGN....DFNSQGATLSVIGRLPLGDIFSVFAEGGGYLYHVDS..........VNGGDDNLAPLAGLGLTARLTDWVDIQARYRYLVRVGDdgTQRWVSDISTATLELVLHPNRTVaapVAAPLVVTPPP
444791046.pir  QAAQAEEPFVNPDWANSAWYLGAGVGRSRANIdsLQANGATVTAFNTDQRDTGYKLFVGKQLNRYLALELGYFDLGKFGFDATTsnGEAGFRGANLDLLAQLPLTERLSLLGRVGANYAKTSThgNRLAGVTSPDHSERKLNAKAGLGLEYKLSEALALRGEVERY.RVNDA..VGNRGDVDLLSVGLYKLGRPasAPVYVPPAPAEAA
262349585.pir  AAIISATLLFASSAAVAEVYVGGKVGKSWLDDAC......QAGQTCEDDKAIGAFLGYQANPWLSIEAGYDYLGKFTAAGL...ADEKVQAYTLAPRVSLPLTEGIALYGKFGGAFVDYGSQ............EDYSYLGAAGLEFNTNNNVTMRLEYQ...NLTDINNDIVRASAESATLGVYKFGGSePAPVVEQRPapAPV
315179386.pir  AAMVSASLLVASSAAMADVYVGAKVGKTWLDDAC......NNTESCDLDGTTAGALLGYNFNDWLALEGGYDYLGKFNGAGL...DRDTIQAYTLAPKLSYALTEQWDLYGKVGAARVRYG.............DASDYSYLGAAGVEYAATRNLGVRVEYQYLS...DMNNDVVRAKAHTTTLGVLYRFGGNdpAPVMAEEQPVAEP
342795449.pir  LAVVISASLMMASNVaaEPYLGLKAGYSWLNDEC......KTNFVCDDDDSASAGIFGGYEFNDYLALEAGYDYLGKFGTGAGLP...DRSVGAFTLAPKLSVGLTDALDLYGKVGGAYVNYGSK............DDASFLGAGLGAEYDVMENGSVRLEYQA...ITDINNDIVRAFANSATIGFAYKFGGSEEapVAEEVMVVEEV
530714708.pir  IALAIAAMGTSTAQAADDWYTGIGAGWAYGHDLN.......DFGKDADKDATALSLFGGYNFNDYYAAELGYLYTGKAGVDG....VDFKTQGATLSGLARLPLGDIFSVFAEGGAYFNHVNGNGN..........SDNGTAPLAGLGLTAKLSDLIDVQARYRYMWNLGDEQKTW.ETDMSVATLELVMHPNrsYVAPVAPAPEPVPE
323310327.pir  AAVISASLLLASAPSMAELYVGGKVGKSWLDDAC......TAATANCDDDASTAGAFVGYEMWDFLSIEGGYDYLGKFTTDGLN...DDKVEAFTLAPRLNLPITNDISLYGKFGGAYVKYGNK............EDASYMGAAGVEFDSHENVTVRVEYQT...ITDANNEQVRAMGNSATLGVYKFGseEAAPVVVVEEEMVEE
114335356.pir  LLTSMLPFAASAAQELTPWYVGGGLGVNNYEPSCDQKTMKVCG....EDDPYAWDVFGGYLFNEYVGIELGYRDLGRAEWtySNKRNDVGARGMSLGFVGFYPLADKWSLSAEAGAFNYLISNKKEYN....TEYYSDSGIAPYLGAGIGYNFTDNLKLQAKYRRYENLDEEKWNTLSMESNYWGLELSYRFGTSAPAPVAAPVVAPVV
449080571.pir  AAIISTTLLFASSAAMAEVYVGGKVGKSWLDDAC......LANQTCNDDDEVVGAFLGYQANKWLSVEAGYDYLGKFTAAGL...ADEKVQAVTLAPRLSLPLTEAIALYGKVGGAYVDYGSK............SDYSYLGAAGLEFNTNHNVTMRLEYQ...NITDINNDIVRASAETATLGIAYKFGGSEAPAAVEQRPapAPV
U2ZK22.pir     LAVLISTSLMFaaPSFSEVYVGAKVGKSWLDDSC.......TSSVCDDEDSTVGAFLGYQMWDFLSLEAGYDYLGEFQ.....NGSMKDDISAITLAPKANLALTDDISLYGKLGGAYVDYGSK............NDWSYLGAVGLEFDSHQNVLVRLEYQTLT...DMNNDVVRAAGNSATLGVVYTFggAEPAPVVEPVMVVEQ
507220598.pir  IAIAMAMGATAAHAADDIYFGAGVGAAHFNGLN.....KIESTNVGEEDAAAANAFVGYNFTENFGTEFGYQYAGRGNTDGN....RYENQGATLSGIARLPLGGDFSAFAEGGAYWAHTDGLGV..........SDTKVSPLAGLGVTYKVNDALDLQARYRYMWDVADLHADnyKSNQSVATLEAVYHPFRtaPAPVVEEAPAPAPQ
404632561.pir  IAIAMAMGATAAHAADDIYFGAGAGAAHFNGL....NKIDGGGYAGTEDAAAANAFVGYNFTENFGTEFGYQYAGR....GNTDGLRYENQGATLSGIARLPLGGDFSAFAEGGAYWAHTDGLGT..........SDTKVSPLAGLGVTYKVNDALDLQARYRYMWDVADLHAGDAPDdSVATLEAVYHPFRTSYAPAPAPVVEEAPA
89050991.pir   IPLAFSVSNSVHASTDNPWYIGARAGASFLTNMD....DAVTSVSEIQDTNFTGGVFAGYQVTPWFALETGYTHLGNA..KLDNKQGSYEAQGADLVTKFSYAISDNVDIFAKAGAFYYDWNAIGS......GVIKSDNGWSSTAGAGLEYFINKSFSTRLEYQYYNDVAG.......PDIHFAGLGLAFHWG....APATVEIKETINV
```

```
24345232.pir    SFSTLASEPPSYLPFQSYWYAGAGLGQGHYSNGSN......PQSYDSVRDRFAGSVYLGYQVNPYLAPELSYQFLGSAYANYEQGQISGDFQQVVLAARFGYPLTTSLYPYVKVGGAGWFGDSEGL.......RSGSERGFSPIVAAGVEYAFTPRLSGRLEHQYTDSLG..ADSIGYTDHHLTTLGLSWRFGHS..APVTAPTPEVVTQ
530714707.pir   IALALASTGALAAP.VNDWYVGAGGGWAIAHDLG.......DFGKDASKDATALTLFGGYNFTENLGAELGYLSTGDWDVQGT....DFSSQGATLSVIGRLPLGDVFSVFAEGGGYLYHVKS..........INGGDDNLAPLAGLGLTAKLHDWVDIQARYRYLVRVGDdgTQRWVSDISTATLELVIHPNRSkvAPAPAPVLVAPPP
169810989.pir   LLTSMLPFAANAAQELTPWYVGGGVGVNDYEPSCDLKTMKVCG....KDDPYAWDVFGGYLFNDYFGVELGYRDLGRGEWtyANKRNDVGAKGMTLGLVGFMPLSQRWSLSAEAGAMNYLLSNNKQYG....TEYYSDSGVAPYFGVGVGYNITENLKLQAKYRRYENLEDDKWNTLDMESNYWGLELSYRFGRTAPAAVVAPVVAAPVD
615550345.pir   IALALAGTVAHAS...DDIYFGAGVGAAHFNGLN.....KVEGVESGTEDAFTGNLFMGYNFNETFGAELGYQYAGRGNTNGGINNNAYENQGATLSGIARLPLGEDFSLFGEAGAYWSHTHGFGV.........TDSRVSPMAGVGLTYKLNDALDLQARYRYMWDVADLTTYtyKSNQSVATLEAVYHprTSYVEPVAAPVVTEAAP
126637318.pir   LLTSMLPFAANAAQELTPWYVGGGLGVNNYEPNCDQKTMKQCG....EDDPYAWDVFGGYLFNDYFGVELGYRDLGRAeVDYANKLNDVGARGMTLGLVGFWPFADRWSLSAEAGAMNYLLSNNKQ....WGSEYYSDSGVAPYFGAGIGYNFTDNLKLQAKYRRYENLEDEKWNTLDMESNYWGLELSYRFGRAAPAAVAAPVVVAPKD
507219684.pir   .MLGLCTGSANaagDSADLYFGAGAGFTHFQGLN.....KINGVENGTEDATAANAFAGYNLNDYLGAELGYIYAGRGNTDGN....RFENQGGSLSLIGRVPLATDLSLFGEAGGYWAHTDGLGT.........KDTKVSSLFGAGLTYQLTDALDLQARWRYITDVADLHSSdtKPNQNMTTLELVYHprSSYVEPVAVERPIPAPE
167352937.pir   VYAAETDNSEAANEFDPYFYLGAKAGQMHYQNAC......ESWSISCDGNDVGFGGFVGYQAWQYLGFETAYLDLGEAVasgVNNTYTGSMKGWEISAVTRFSLSESFELFAKAGSVYWDGDNR......GPQSRNSDSDWAPMLGAGVEYQLSPSWVARLEYQYIDSLGSDLIGG..SNGHLTTLGISYRFGQSaaQPPVKETPVTLPE
10039003.pir    IAFLLASLIPSaaEEENGWYLGAKFGWSHFNPLKYDMNNSQVNNNDsnLSAPIVGLFLGYEFNPYFSLEIENDTNGFFPHLIFQKNNEhqSNSVQLATKLSYPITDEFHLYTKLGGIVSWNDLSSKNTLKNLFSKES..ALLPSLSLGAEYIFNETFITRLDYTWKNSVKNIANASIKPALGDAVLSIGWKFGKSDISDMFSSDDSEPLN
157845903.pir   LLTSMLPFAASAADGMSPWYVGGGVGINNYQPNCDQKTMKSCG....EDDPYAWDVFAGYLFNDYFGAELGYRDLGRAqVDYSNKLNDVGAKGMNLGLVAFWPFAGDWSLSAEAGAFNYIISNNKNYG....SEYYSDNGVAPYVGAGIGYNITDNLKIQAKYRRYENLEDEKWKTLEMESNYWGLELSYRFGssKPAPVVAPVIVADSD
268161981.pir   LPLALLVSAAANAATDNPFYAGARIGNAIYD........TDHKGPNVDDANLAGGAFLGYQIAPWFAVEAGYTHLGSTTIKGT..DAKFRTQGADIVGKYSYAINDKIDVFGKLGAYYYDVHTKNL.......ADAKEHGWAGTAGVGAEYYLTNNLSTAVEYQYYNDVAGA.......DIHFAGVSLAYHWGA..PAPVVAEPVYVDQV
408884556.pir   NEQAGASNIESNASHSEHFYLGARAGWAAYQDAC......SSSAQECNDDIFAYGLYAGYQFNQWIALEGGFTDYGKPDAKYSNGKVSTDTYGAEVAAKLSYAISPNWEVFTRIGAAYQDIDKQSDWA......ESEQSQDWNAMASLGIDYRLSHNWSLRGEYQFIDGIGDDIVQG..ADLHFTSIGLTYHFGEEAvaPVAIPEPRPETV
237500166.pir   GFVASAMSVSASANDAEGAFLGAGFGGNNYSELG.......DVGTADKSSTPAAHVFGGYTFNEYIGAEAGYNWLGNGRVDGE....RFKAHGANISLLPRYPLTEDLSLLGELGAMRWDATNEGT........DVSDFGVSPIFGIGLAYRVSDPVDLQVRYRHIDGVGDAKTG..ESDSESLGLDVVYYprSSTPAPVVTPAPEPAPA
485941725.pir   LSALIAVAFASAaaQANDWYVGGGGGVSIGHDLD.......DLGSDVDKNDTAWSLFGGYNFTENFGAELGYLNTGDWSVDGE....QFSSQGATLSAIGRLPLGDIFSLFAEGGAYIYQVDT..........INGSDNDYAPLLGLGMGAQLTDWLDLQARYRYMFRVGDdgTDRWVTDVSVATLELVFHPNRHavAPVAAPMVAAAVA
151364264.pir   LLASLLPFTVSAAQELTPWYVGAGLGVNNYEHI.........ATQNGQDDPYAWDVFAGYMFNDYFGAEIGFRDLGNADWTSAGISNDAGVKGSTLGLVGIWPLANRWSLSAEAGAMYYSLENSQNTA..GASTKYGSSDFAPYLGAGVGYNFTDNLKLQAKYRRYENLNDDYNI.VEADSNYWGLELSYRFGtVAAAPVAAAVIAAAPL
651306659.pir   IALAIAAMGTTTAQAADDWYTGMGAGWAYGHDLD.......TFGQDADKDATALSLFGGYNFTENFAAELGYLYAGKAGVDG....VDFKTQGATLSGIARLPMNDIFSVFAEGGAYFNHVNGNGQ..........SDNGTSPLAGLGLTAKLSDLIDLQARYRYMWDLGDDQKTW.KTDMSVATLELVMHPNRTtvAPAPAPAPAPEPVPE
342815798.pir   LAVVISSTLALASMNAndFYLGGKVGKSWLDDAC......TSITANCDDDSMAVGVLAGYQFMDYLSLEAGYDYLGEFTADGL...ADDKVKAFTIAPKLNLSLTEDISLYGKFGGARVDYGNK..............NDNSFLGAVGFEVDSHKNVSIRLEYQRLTDVNN...DYVRAAVNSATLGFVYKFgqLASEPYVAEEKVVEEV
291067496.pir   TTFYSIPFAVMASTSSDGLYAGANMGWSQYYNNN.......DFTRGHENNDIGVGGYIGYQLNPYFSLENGILILGDAEGD...KNSSMTVQGYQLSGKISYPV.SNTDIYTRLGGMWYRSDYTRD....DTNDSKVGTGIAPLISLGIEYAWNYNITSRVEYQWVGNIKNDAATSANVDNGLLSVGFSYHFGAQSSPSTTAVAMHQEQP
469769914.pir   LAALLVSSTAFAAHTDNDWYTGLRLGATDYTD....YKISGGESSSAIDANIGGGLFLGYNFVDWFALEGGYTYLGELETSE.....KITNHALELVGKFTLQATDNLDLFVKAGAFGYKTDAD..........SANDDGFNATAGFGAEYFFNDNVSTRLEYQYYNNLT...LDEAKWDAHLLAVSLVYNWGSPAaaAVAAPIVASQLV
517830321.pir   SSVVAFGSVAYAA.EAETGYVGAKVGWSHTESHS...GRNAVGVRHNRNNNWDYGIYGGYNFTSFLGLEAGYTYFGNFSNKggKRLGKHTLHGVELASLLAIPFNDTDDVFFKVGGLIINNHDKAF.........GHTETKTAPLLGLGTRIAF.GDFITRLEYNYAHKITD.STYGYQPDLSTINFGLEWKFGGAKPAPVVAPVEQPKTV
323316507.pir   AAVISASLLLASAPSMAEFYVGGKAGKSWLDDAC......TSTTVDCDTESSTLGAFAGYEMWDFLSIEAGYDYLGRFDGEGL...DNDKVEAYTLAPKLNLPITDAISLYGKFGGAFVNYGS..............EEDYSYLGAAGVEFDSNENIAVRLEYQTLTDINN...DLVRAKGDSATLAFVYKFGSHEEapVVEEMVEEVVE
519121657.pir   LAAVISASLLMASAAQAEVYVGGKMGKSWLEDAC......VAGQSCDKDDSTLGAFVGYEFNDYIAVEAGFDNVGDFDNTS....FGGHVEAITLAPKFSLPITEDIALYGKVGGAYVMFDGK..............DDYSYLGAAGLEFNLSQNVTARAEYQT...ITDINNDIVRATGNTATLGVSFKFGGSE.EPVVEEAPIVEEV
293328175.pir   QANQVKTHEASKVQDDSFFYLGAKGGWINYQNAC......EAQAVDCDANDSAWGGFAGYQFHRNWAVETGYNDFGQAGasGLLNEYIGTMKGWEFSLKGNLPLTENLDLFGKAGTLRWDGENS......GPFSYDSDDGWSPMVGIGLEYKLTPSWVARVEYQYFDSVGSDKLGG..SNMHFTSLGLSYRFGQTKPVAKSVTQPAPVAV
404623133.pir   IAIAMAALGATAAHAADDIYFGAGVGATHFNGLN.....KLESTNVGQEDAAAANAFMGYNFTENFGTELGYQYTGRGNTDGN....RYESQGATLSGIARLPVGNDFSLFAEGGAYWGHTDGLGT.........SDTKVSPLAGAGVTYKVNDALDLQARYRYMWDVADLHANGERYKAnaTLEAVYHPFRTSYVAPVAAPVVEEAPA
651332620.pir   LIALAIAATGTTAQASGDWYTGIGAGWAYAHDLDN......VQGLDVDKDATALSLFGGYNFTENFGAELGYLYAG....KGGIDGADFKTQGATLSGIARLPMGDVFSLFAEGGAYFNHVNGNG..........DSDNGTAPLAGIGLTAKLSDLIDVQARYRYLWNLGDEPKTW.ETDMSVATLELVLHPNrsYVAPVPAPEPEPVAP
114551368.pir   .....NTSLAMAA.DDSGFYLGGNVGQSVSHLDESGMTNRVSGPGLadNRDIGYKLYGGYQANKYLSLESGWFDLGRFNFTSTTtlGNIKVRGVNLDALIRLPLTDQFSVFGRGGLTYAQTRDSFStlALPANASPKKWAWNPKYGVGLDYNLTRAFGIRAEAERY.RINDA..TGSKGNIDLFSLGMTYRFGTEKPAPAPVAVVEPEPV
342803917.pir   AVVVSASLVMAANAAVAAPYVGVKAGYSWLNDEC......TATSLCGDDDSASAGIFGGYEFNDFLALEAGWDYLGKFTGAGLP...DDSVDAFTLAPKLSFGVTDAIDVYGKFGGAYVNYGDK..............SDESFLGALGLEFDVMENGSVRVEYQALTDINN...DVVRAFANTASIGFAYSFGGSEaePVVAEEVMVEEV
323310326.pir   LAVVMSGALLLaaSANAEFYLGGKVGKSWLDDAC......TALTSDCDDDSMTAGVIAGYEFFDMLSLEAGYDYLGEFTADGL...ADEKVKAFTIAPKLNLPLTEDISVYGKLGGARVEYGNK..............DDNSYLGAIGFEIESDDNFAVRIEYQRLTDVNN...DLVKAAVNSATLGLVYKFgqQEEAVVIEEKVVEETV
651306658.pir   IALALATSAAQAAP.VHDWYVGAGGGWAFAHDID.......DFSQDVGKDATAINAFVGYNYNENLGAELGYLSTGDWDIAG....QDFNSKGATLSVIGRLPLGDIFSVFAEGGGYLYHVDS..........VNGGDDNLAPLAGLGLTARLSDWVDIQTRYRYMVRVGDdgTKRWVSDISTATIELVLHPNRTVAapVAAPLVVASPP
640349854.pir   GLVASAVSVSALANDAEGAFLGTGMGWNNYSDLG.......GLGTANKSSTPAVHVFGGYTFNEYFGVEEGYNWLGNGRVNG....QRFKANGLNISALPRYPLTEDLSLLGEVGVMLWNTDNQD........LQAKDHGYSPVFGAGLAYRVSDPVDLQLRYRYMNGLGDAQT.GASDSSNVMFDVVYYPTRTSTPAPVVAPAPEPVAA
260937018.pir   LAVVISATLMLSAAANAEFYLGGKAGKSWLDDAC......TSATTNCDDESITAGLFAGYEFLDYLSIEGGYDYLGEFTADG...ISGDKAHAWTLAPKLTIPFNDTVSLYGKFGGAYVEYGDK..............DDNSYLGAVGLELNSDSNVRVRLEYQRLTDVNN...DLVKAAVNSATLGFVYHFgqTEQEPVVVEEKTVEVV
119766551.pir   LASALPFGVMASDEGMSPWYVGGGVGINNYEPACDQKTMRSCG....EDSPYAWDVFAGYLFNDYFGVELGYRDLGRAEWTDYANnnDVGAKGTTLGLVAFWPFASKWSLSAEAGAMNYLVYNNKR....WGSNYYSDSGIAPFVGAGIGYNFTDNLKLSAKYRRYENLDEEKWNTLEMESNYWGLELSYRFGS.KAAPVVAAPVVVAPV
224604377.pir   GITLLSVPAAHAaaAATGWYGGISVGQSKAQDFSASDFDSTLAQGgvDDTDTGWKLFAGYQFNRYFAVEGGYLDLGRFDAHTTVTAVDGskDGLFIDAVGILPITEQFSAFGKLGVYSLKTEISAS..AGGRSASDSARNSDLTYGLGVNYAFTKELALRAEWERFHKVGDSDKTGQS.DVDLLSLGLTYRFY................
486370256.pir   LIVALSPVASVFAQDDSNFYLGGRFGVSTLTDAC.......EAGDCGSNDDVAGGLILGYDFGNNFSIESTYDYLGRFDTAFTGVngAGDLTALTLAPKFNVGVTDLTDIYGKLGLAWWNWNSKV........GTGDVDDYTILAALGVDHRANDLVNVRLEYQY...IPDMDKDYFDADNHLITAGVTFHFGrsEPEPVVVEEVVVVEE
91716086.pir    LLTSMLPFAASAAEELTPWYVGGGVGVNNYEPSCDLKTMKTCG....KDDPYAWDVFGGYMFNEYFGLELGYRDLGRAEWtySNKSHDVGARGASLGLVALIPLANNWSITAEAGAFNYLLSNKKQ....TSTEYYSDSGVAPYIGAGIGYNFTENLMLQAKYRRYETLNEKQWDTLSMESNYWGLALSYRFGT.AAKPVAAKPAPVAPM
157845752.pir   VYAAEADSSEPANEFAPYFYLGAKAGQMHYQNAC......ESWSVSCDGNYVGFGGFAGYQAWQYLGFETAYLDLGEAVSGYsnNTYVGSMKGWELSAVTRFGLSEDFELFAKAGSFYWDGDNQ......GPYSRNSDSGWAPMLGAGLAYQISPSWVARLEYQYIDKLGSDLIGG..SNGHLTTLGISYRFGQKKPASVSQVKEKPITL
161329641.pir   LLTSMLPFAANAAQELTPWYVGGGLGVNNYEPNCDMKTMKSCG....KDDPYAWDVFAGYLFNDYFAVELGYRDLGRAevDYANKMNDVGAKGLSLGVVGFWPIANRWSLSAEAGAMNYTLSNNKEYG....SEYYSDSGIAPYIGTGIGYNITENLKLQAKYRRYENLDEDKWNTLAMESNYWGLELSYRFGT..PAAVVAPIVAAVVV
335341263.pir   AAVSMALVFASSAAMA.EVYVGAKVGKSWLDDACR.......AGDVCEDESSTVGAYLGYQAFNWLSLEAGYDYLGKFSGAGLN...DEKVTAITLAPKVSLPLTDAIALYAKVGGAYVDYGNK..............DDYSYLGAAGIEFNTDNNVTLRLEYQSLTDINN...DLVRAAGNSATLGVSYKFGGSKAEPAPVIVQEVFVE
148835798.pir   FNVLADETSADSDAFSSHWYLGGKLGAMKYQNAC......EEWAVDCDGTDVALGGFIGYQVWQHWGLELGYLDFGEAKAlgRVNQYSGSMSGIEMSVVGRISVTDNLDAFAKAGTLYWQGKNS......GPTRSLTDDGWAPTAGVGLEYHFSNHWAGRVEYQYVNALGSDKLGG..SNGHLATLGIVYRFSSAesKPETQTEPKPVTP
369843134.pir   VAAVVAGGSALLNTAQAEMYIGGKVGMSTLDDACY.......LNSPCDDEAFAAGMHVGYGFNEYFDLEYGVDFLGNYEANFKTAAGADtlWAMTLAPKLNLPLSDDFNLFAKIGGAYMMSG..............DEKDFVPTGSLGAEYALNRNLSLRAEYQRYQDMSD..DVWDDMDANFFSVGVNYTF...AAAPVVAAVVEPEPE
345538703.pir   IAFLLVSLVTsqAEEKNGWYLGTKMGWSHFNPLKYDFNNYQINKNDSITNNfiIGLFLGYEFNPYFGFEIENDTNGFFPHFMFQNKKEhqSNSVQLATKLSYPITNDFHFYTRLGGIISWDDLASKNTLRNLLSKESS..LFPSLSLGAEYIFNEQIITRLDYTWKNSVKNLVDLSFKPTIGDIALSIGWKFGKSYISDIFSSYDPELSN
149805744.pir   ENKSTMISAKEVAESDAFFYLGAKLGVNHYQNGC......QPWNVSCDKTDEAGGVYGGYQFNQYFALEAAYLHLGDapVNGLNQTYTGTMQGLELSALGLLPITDRFSLFAKGGVYNWHATND......GPHQRIKESGLSPTAGLGLSYQLSISWQARLEYQYFYELGNEEVGG..SHGQLTTLGIAYQFGRIKPTPVVQNIYMPVQL
320577106.pir   ................................................................................................NGAFKAQGISLAAKLSYPLTDDLDIYTRLGGMVWRADSKGAYDNGNarVSDHDTGVSPLAAVGLEYAWTKNWATRLDYQWVNNIGDAGTVGTRPDNSMLSVGVSYRFGQDEVAP.PAPAPAPVVQ
U3CEB4.pir      VATILAGGVMSNVALA.DTYIGGKLGYNSLDDSC.......HLNAPCDDDAFAAGLHLGYNFNEYIAAEYGVDYLGDFTgkAGKNVVDGDLWAITLAPKFNLPLNDAWNLFAKVGGAYMMAG..............DEKDFVPTGSLGAEYQINYNWSLRAEYQRYQDMSD..DIIKDMDANFFGLGVNYKFG...AEPVVQEVVETRPA
480569186.pir   TAIASIVLALSSPLYAGDWYLGGGAGLSVGHDMKDY.......GSDADKEDTAYDLFAGYQFNDYFAGELGYLNTGSWQGS....NGSLKSDGATLSAIGRYPLNDRFSLFAEGGGYLYSASTP..........NGSDTNLAPLAGLGMTVRLHRLVDLQARYRYMVRVGDdgTRRWVSDVSTATLELVIYPNRDEPVPPVApaPAPEPV
404626345.pir   IALALASTGAQAEP.VHDWYAGIGGGWAIAHNLN.......DFSQDVGKDATALSLFGGYNFTENLGAEIGYLSTGDWDIGG....HDFNSKGATLSVIGRLPLSEMFSLFAEGGGYIYHVES..........VNGGDDNLAPLAGLGVTARLHDWVDLQARYRYIVRVGDDPDNgwVSDISTATLELVIHPNRseAAPTPAPAVVPPPP
212557967.pir   LLTSMLPFVANAADGMSPWYVGGGVGINNYEPNCDQKTMKTCG....DDDPYAWDVFAGYLFNDYFGVELGYRDLGRAevDYSDKLNDVGARGMNLGLVAFWPFANRWSLSAEAGALNYLISNNKNYG....SEYYSDSGVAPYIGAGIGYNITDNLKLQAKYRRYENLDEDKWKTLEMESNYWGLELSYRFGSKSKPIvPVAVVVGDAD
328473467.pir   LAAVISASLLMASAAQAEVYVGGKMGKSWLEDAC.......VAGQSCDKDGSTLGAFVGYEFNDYIALEAGFDNVGDFDQTS....FGGHVEAITLAPKFSLPITQDIALYGKVGGAYVMFDGK..............DDYSYLGAAGVEFNLSQNVTARAEYQT...ITDISNNVSKAAGNSATLGVSFKFGGNDEPVvvEEEPVVEEV
536747537.pir   AAVVSGTLLLASASANAQFYLGAKAGASWVDDLC........TTGECDDNSWALGSFVGYEFNDYLALEAGLDTLGETTGSGYKDAGLVS...YSLAPRFSLPLTENIDAFAKAGGAYVEYGNK..............SDSSFVGGVGLSYSVLPNIDIQVEYQRLTDI.DIDTHGI..SGNAVTLGFTTKFGGSdeAPAQEEMLVEETV
387936359.pir   AMVSSPVLFAQADNKYdnWYGGLNIGQTHESINDGAIAQSEIGGGLtdDRDNGYKVFAGYQLNDNFAIEGGYFDLGQFGSRAVKGntRTKTRGFNLDLVGSLPMTEKLSAFARAGAHYSESKDRFQGSDNVIVTDRhdRDVNFKWGGGLQYDFTQKFGMRLEAERYA.INDVM..GNNGNINMYSLGALYRFGEPNAAPAAAYVEPVTRR
307631547.pir   CLPFTVSAEAGDDTYADHLYLGAKAGWSHYHNGC......ESGASDCDKDDLGWGAQLGYQFSPNWAAELEYLDFGQAEATYPfgTYTGSQRGWALSARASLPVTEQVELFGKLGALYWQGKVE......GPTPTRKDEGWAPVAEVGLGYRFSQRWQLNLATQYGDGVGSDELGG..SNLWLTTIGVQYRFGADRtaVQPTPTPPPGPV
371492849.pir   LIISNGTVASESKEEQSHVYLGGQVGVSHFLGAC......SSNAIECKNYVTGGGLYGGYQFNSWFALEGSWHDYGSPKTFYGVGDGYYsaTGVDLSVKLSLPVTDNLDLYAKGGAAYNYLSVSGNDNV.HLGMFESDSSLDGIWEIGAEYALAPNWSLRFGTSVIDGIGNAKTG..KSDLYFTSLGLTYKF...KAKPEPKPETIVKLV
88816686.pir    SSFAAHSEEAQanFNFNNWYVGGEFGRKRQDDLC......KKSLSSCDSSDNAWSVFFGYEFSKHWALEGGYTDLGEYSTTGLVNGLTNHatGLEFAGVFRMPLTEQAGLFTKLGAMYYDGEEKNSIA......KFDDKGWSALFGLGLSYDFTQNLQGRVEYNFLHDLGDSEFAGDH..GHLTTLGLAYRFGSNAKKAPRAPAPVTPVA
309367068.pir   QAVVISASLLMASVAHANVYVGGKVGKSWMDDAC.......VAGQPCDDEDSTYGAYAGYEFNQKIAIEGGIERLGNYDKSAFNGN....VQAFTLAPKFSLPVTPDIALYGKVGGAYTKFDGK..............DDYSYLGAAGVEFNISSNVTARAEYQALTDINNDVT...KAMGNSATIGISYKFGASQAPVATQPEPVIEDV
262219507.pir   LMVALSPTANVFAQNDANFYLGGRLGVSTLNDAC.......TTGNCGDNDDVAGGLILGYDLGNKFSIESTYDYLGRFNTaqGANAGAGDLTALTLAPKFNVSVTELTDIYGKLGVAWWDWSSKA........GTGDVDDYSVMAALGVDHRANDLVNVRLEYQY...IPDMNKEYFNADNHLITAGVTFHLGRQ.AKPKPRPRVVEKVV
159173032.pir   VATVLAGGVMSNVALA.DTYVGGKLGYNALNDAC.......HLNAPCDDDSFAAGMHIGYNFNEYVAAEYGVDYLGNFTakAGLNTVDGDLWALTLAPKFNLPVTDSWNLFAKVGAAYMMAG..............DEKDFVPTGSLGAEYQINYNWSLRAEYQRYQDMSD..DIIDDMDADFFGIGFNYKFGS...EPVVQEVVETRPA
260611401.pir   VAMISASLMAASSSAMADVYLGGKLGQTWLNDSCR.......AGEVCEDRDTSMGAFLGYNLNEWLAVEAGYDMLGKFTGIGL...ADDRVKAITLAPKFTLSLSPSVGAYAKLGGAYVDYG..............DESDYSFLAAAGLEFNVSPNVSLRTEYQVLTDINNDQT...RAEANTVSLGVSYRFGAAapTQVVEIRPVTVIE
190685438.pir   CAISTITSYYAAaeVETSIYVGIRAGYSYNEGSC......SSNHLKCDKSDTGYGLFIGYDINPRWAIETSFNDIGDTTATYSNAHLDGDLSQIDISVKYAYPIYQNLSLYGKVGAAYWDAKAK......GQNVSFSDTGVSPLLGAGLELIISEHWKGRIEYQYIDRIGNNLMGKASPNKLGLALIWNFPLRKHKPapVIVYQPVEAPP
K2I0S7.pir      ....MATGAAQAAP.VHDWYIGAGAGWAWAHDLD.......DFSKDVSNDDTAYNAFAGYNFTENLGAELGYLSGGDWDVNG....HEFSNQGATLSILGRLPLNDIFSVFAEGGGYLYSVKS..........INGSEDNLAPLAGLGMTARLHDWIDIQARYRYMVRVGDdgTQRWVSDISTATIELVLHPNRTVAapVAAPMVVAPPP
575427746.pir   VFLLASLVTSVQAEEKNGWYLGTKMGWSHFNPLKYNIDDLKKTDNTSSesAPIIGLFLGYEFNPYFAFEIENDTNGFFPHRIFQKEQKYTqnSVQLATKLSYPITDDFHFYTRLGGTVFWENLVSKQDLKSMFSKES..KLFPSLSLGAEYVFNKKFITRLDYTWKNTIRNIIDSSIKPSLGDAILSFGWKFGKSDVDDIFLSDDSELLN
639601994.pir   VALAGFATVAQAAPKDNTWYTGAKLGWSQYHDTG.....FINNNGPTHENQLGAGAFGGYQVNPYVGFEMGYDWLGRMPYKGSVENGAYKAQGVQLTAKLGYPITDDLDVYTRLGGMVWRADTKSN...............................................................................
151937341.pir   LAAVISASLLMASAAQAEVYVGGKMGKSWMEDAC.......VAGQACDKDDSTLGAFVGYEMNKYFAVEAGFDNIGDFNQTS....FGGHVEAITLAPKFSLPITEDIALYGKVGGAYVMFDGK..............DDYSYLGAAGLEFNLSQNVTARAEYQTLTDISNDVT...RATGNTATLGVSFKFGGNDEPVIvEEPVVVEEV
U2ZIH7.pir      AALISASMLLASTSAMAEFYLGAKAGKSWLDDAC.......TSTSACDDEDVTLGVFGGYQFWDFLSLEAGYDHLGKFDAAGL...DDDSVQAFTLAPKLNLYLTDSFSVYGKFGGAYVDYG..............DENDASYLGAVGLQLDPHKKVAVRLEYQVLTDINN...DIVRAEANSATLGVAFKFGAEEEPVMMVEEEMVVEE
159173699.pir   LAAVISASLLMASAAHAEVYMGGKMGKSWVDDAC.......VAGQACDKDGSTLGAFVGYEMNDYFAVEAGFDNVGDFDQTSLG....GHVEAITLAPKFSLPVTEDIALYGKVGGAYLMMN.............DGKDDFSYLGAAGVEFNISENVTARAEYQA...ITDISNDAVKAMGNSATLGVAFKFGGNDEVVvEEPIVEEVVV
469769863.pir   AIQKKDNENLSALQQHAFAYVGAKLSVNHYQNAC......QSWAIDCDATDIAGGFYAGYQFNKYFALEAAYLNLGKVDanEAEKNFTGSMQGIEISALALYPLSNDFNAFAKAGVFNWQAKSE......GGPLTQKDYGLSPTIGLGLSYQISKQWQARLEYQYFANIGSAEIGG..ANAHLSSLGISYQFKHEksKPAPLEIVYLETI
372985970.pir   IAMALATAGMSAGVQANDWYLGAGAGYADYEDIDR.......NNVDADDNAAAVSLFGGYNFNQYFGTELGFTNFG..........GDADTRALNLSAIGRLPLGERFSVFGEVGALGWDTDVA..........NGDEGVSPLAGLGLAYRASDLVDVQLRYRRVEDMGDEVNNGFETDVNNVMLELVLHPNRRVAAPAPVAPVAPQPQ
322416809.pir   IAALGFAVAAQAAVPE.TAYVGVRGGWAHMDNVD.......WASADDKDAVGYGIYAGYNLTSWAAIEAGYNYFDGFSAKDVEGSHDFDVHGPEAALRLAFPLgdGSDIYVRGGAFWATTKVE.........HGISNHEIAPFVGAGVQYAVTKNFGVRVGYDYYFDVID.DKDAPETDIGLLYAGLQFTFGGSEPAPAPAHAPQTVTE
432510784.pir   VATVLAGGVISNVALA.DTYIGGKVGYNSLNDACY.......LNEPCDDDSFAAGMHIGYNFNEYVAAEYGVDYLGDFTakPGLNTVDGDLWALTLAPKFNLPLTDSWNLFAKVGAAYMMAG..............DEKDFVPTGSLGAEYKINYNWSLRAEYQRYQDMSD..DIIDDMDSDFFGIGFNYKFGSEPVVQEEAVEEVVETR
408881058.pir   LAAVISASLLMASAAHAEVYVGGKMGKSWMEDAC.......VAGQACDKDGSTLGAFVGYEMNDYFSVEAGFDNLGDFDQTS....FGGHVEAITLAPKFSLPITEDIALYGKVGGAYLMMN.............DGKDDYSYLGAAGLEFNVSQNVTARAEYQT...ITDINNDSVKAMGNTATLGVSFKFGGQDEVVvEEEPVVEEVV
536747538.pir   LAAVISTTLLLASTSAnqFYLGAKAGASWLNDSC........STGPCDDNSWALGSFAGYEFNDMISLELGLDTLGETTGYGYTDAG...LSSYSLAPRFTMPLSQKFDAFAKVGGAYVEYGNK..............DDSSLLGALGVTYAFMPTIDFQLEYQ...RLTDINIDTHRFKGNALTLGFIAKFGGSdaQPVQEELMVEEVV
469772046.pir   ICPLLPFFLQAAENNTNNFYIGAKAGWSKYQDACLY......GYTKCEEDSLGYGLFVGYDFTDWFAIEGGVTNHNEVNSSYQNYDVEALAISSDLSTKFGYRLTDNVQLYTKVGISYQHINKTMS.....DLGDFSGSSFGGLVAMGIDYKLDNNWSLRGEYQFIDGIGD..DTSLKADLHFTSVGIKYRFSqKDIIPITQPKETVVSE
348002024.pir   .........MQAAPQANTFYAGAKAGWASVRHGLNQYQYEQDGtgKAKKNSEAYGIFAGYQITDNFAVEAGYEFFGRGKVKEATGEKRITAHGSSLSLKASYPVLHNLDLYARAGAALIRVDYKDNTF..GETHKFHDLKVSPVFAGGLNMPF....FLNWHYVWSING.................................
342822254.pir   QLVVLTLLSSLPFTASSQIYIGGKAGASSLSNSC.......AVDSNCDDASSAFGAYVGYQLLDNVALELGYDDYGKFVSngTLDVIDGSMTAITFAPKFNIPLTDDLDFFSKLGAGYSQYE.............EAKDDLNLLAGLGFEGKLTEALRARIEYQLMTEINAGPADDTVA..SMFTLGLNYHFGVKSPEPVAKVEPRPAPE
369842914.pir   VATVLAGGVISNVALA.DTYIGGKLGYNALNDACY.......LDTSCDDDSFAASMHLGYSFNDYIDAEYGVDYLGDFKakAGKNTVDGELWALTLAPKFNLPLTDSWNLFAKVGAAYMMAG..............DEKDFVPTGSLGAEYQINYNWSLRAEYQRYQDMSD..DIIDDMDADYYGIGFNYKFGSEPVVQEEVVETRPATN
148868362.pir   VAFTLGTSMANAA.SVSDFYIGSKLGYSQVDSDC.......ASEFQCDKDSASVGAYLGYQYNDNVAVEYGVDYLGHHTTshLTDVLTTDVLAISLAPKFSYSLTDNLALFTKIGAAYMIAE.............EENDVVATASAGAEYALTQNLKLRAEYQRFEKMSDIHIEDMSFNN..FSFGISYQFGEQNQAVVESAESHQDIV
519728038.pir   LLLLATAASLFSLSAQAEWYVGGALGQARFDDASVSGKVMGYStsLEDDKSTTASIFGGYQFNRYIALEGTITGIDALDGSVVTVG...DMSYIALQPKFSLPVTDQVSLFAKAGLAYFNAEIKVSNSVAGYSgtFSDSDVTGILGLGVEVAATDNWHIRASWDYLRPELDVVNVGYAsdINVFSLGVSYHFH................
148835419.pir   AASIIGTMMALPVVADDGFYLGGRLGGSQLSDACN......VSAAPCTDSGFSAGVYTGYDWNDYIALEFAYDWLGSYETsgAEYRRDANLSALTLAPKFSYPFNDEWAIFGKVGAAYTMYD.............DRKSTALMGGVGLEYDFATAWSGRLEYQ...RINDAKDDWFNTDVDTFWLGISYSFGSASEAAATAAASSTQE
149286.pir      ....................................................................................................LTAKLGYPITDDLDIYTRLGGMVWRADSKGNYASTGVSRSEHDTGVSPVFAGGVEWAVTRDIATRLEYQWVNNIGDAGTVGTRPDNGMLSLGVSYRFGQEDAAPVVAPAPAPAPE
145964761.pir   CAVIAASGFAAAPSISATTYVGAKVGMGWLDSAC.......VDSVNCEDDSVAGGLYGGYNFTDNIALELNADYLGDYdyNGTTQRYSDSIIAISLSPMYRLAIKQEFDVFFKAGPAYIMHD.............DEDDVVLALGIGAEKQFADDWAVRLEYQYFDDFDD..KIIQNLNSNLVTVGLSYNFGTGNTTASAAASAAAVT
342815799.pir   VVVASSLILLVSASANAQIYLGAKAGASWVDDLC.......TSGSQCDENSWALGSFLGYEFNDHFALEAGLDTLGKTTGAGYTDAGLIS...YSLAPRFNIPLTNSLDVFAKAGGAYVEYGNK..............SDSTLMGSLGLSYNVLPNIDIQLEYQ...RLADIDIESHGISGNAVTLGFTTKFGSSanEPVVQEEMQLQNV
469770726.pir   STSHEFSDYSDTRQNNAHAYVGLRGGGTYFEGAC......SSNGSECNDNTFGYGLYGGYQFISWFALEGAVTHYGTPDASYGLKNISAEVLGSELTGVFSYDLSESFDAYARVGVAYQNIEKNST.....GGSELTSNEWGMLSALGVDYRVSQNWSLRGEYQFIDGIGNSE..LMKSDMHFLSLGLTYHFGQEKALIVATTALAVATP
U4JIA9.pir      QLVVLTLLSSLPFTASSQLYFGGKAGVSSLKNSC.......AVGSPCEDDSNGFGGFVGYQLIDNVALEFGYDKYGDFKSngTIDTLEGDLTAYSIAPKFNVGLSESIDFFSKIGAAYSYYDT.............ANDDLNLLAALGLEGKLTDAIRARLEYQVMAELNSGPAKDT..SASMFSVGLSYHFGTKKPEPVAKVEPRPAPE
229485743.pir   ....................................................................................................LTAKLGYPITDDLDIYTRLGGMVWRADTKAHNNVTGESEKNHDTGVSPVFAGGVEWAITPEIATRLEYQWTNNIGDANTIGTRPDNGLLSLGVSYRFGQGEAAPVVAPAPAPAPE
532610063.pir   AALLATCSMANAAP.....YIGASLGKATFDDISGSIKSNTYNASEkdNESLSGKVFGGYTFNEYigAIGGYDAlgSVVTVG.......DMKFLAIQPKLTLPIGDRFNLFAKAGLSYFNAEFKVSNSVLtgHTTFSETTVTGLYGLGAEFAITKNLALQVEWEYMKPELDVAKLgvEAEISAFSVGMSYRF................
548008408.pir   SALTAAALILTALPaaGNWYGLISAGLVHSDNNYKEAVGAVNtvNDADNNNVTAKLAVGCRVNDHFAVEAAAWWFGKSEHDHAGihAEFKGYGLAVDAVGILPVTDALSVFGKAGVALIRIDGKNNASEPQYSFSKSKTRLAPKLGAGAEWLMTERLSLRLEYEHFFNAGKESRDGQTfdYDTVTVGLKMNF................
K2IWD0.pir      ......................MGAGWAYGHDLD.......SFGLDADKDATAISLFGGYNFTENFAAELGYLYAG....KGGVDGVDFKTQGGTLSGIARLPMNDIFSVFAEGGAYFNHVNGNG..........DSDNGTSPLAGLGLSAKLTDLLDLQARYRYIWDLGDDQKTW.KTDMSVTTLELVMHPNRKavAPVAAPAPEPAPE
```

```
323316506.pir   AAVISTTLLVASASANAQFYLGAKAGVSWLNNLC......TSSSSSCDEDSWALGSFLGYEADDFVAIEFGLDTLGKTTGAGYEDAG...FSAYTLAPRLSFPMNEDLDLFVKAGASYVQYGDK..............DDETLFGALGLNYNLFANIDLQAEYQHFNNIDIDTT...RFHANSFTLGFRTKFGGSD.EPTPEPVQEELVE
U3B4F4.pir      PLILAFTSTAFAAESdsHFYGGIKLGTANYGDLG....SAVTDVAGVDDSPFSWGAYFGIQALPWLAFELGYHDLGEADLKD..VSGSYEAKTLDLTAKASYEFAKQFSVFGKVGFQAYEWNAN......GENVYEDDQGWTPHLGAGVEYQFHKNWSGALEYTWYNDIGG......PDINYFGISATYHWF................
218324441.pir   CAVVAASGLAAAPSIAATTYVGAKVGMGWLDSAC.......VSGEKCDDDAIGAGIYSGYNFTDRLGVELSSDFLGDYKTsgVTSNFSDPLIAISLTPMYRLPVQQEFDVFFKAGPAYISHGG..............ETDVVLSAGIGLEKQLSSDWAVRVEYQYFDDFDD..NYVQDLNSNFVTVGLSYNFGTGNTTASAAAASAAVVT
345539257.pir   LLASLVTSVYAEENNQNRWYMGTKMGWSNVNPFTGNINIFKIKKENILDdaPILGLFLGYQFNPYFSLEVENNTNGFFPHLIFQKNKSIMQhtMKIATKLSYPIIDELNLYTKLGGEIVWNDLISKNVFTNIFSK..DSALSSSLSLGAEYVFNDSLITRLDYNWKNSFKNIINHSIKPDLGDAVFSIGWKFGKSNTNDIFSSYDPEFLN
386422620.pir   CLLLGLTSMAQafSTGPKMYVGSSIGGSQQSDSCNDPFFE....GSCDEGDLAWKAFGGIRFNPMLGAELGYYDLGSVNMDGTAGglDSSATGTAIAGVGYVPLTSQIEAFGKAGVIAW..DQKNNKTSGASSTQSAESGTSTLLGGGAQYQLNSNLFLRGEWEHMFNIGS..DSAYETDTDLYSLGVSYSTL................
398217221.pir   LAASTAASTALAAPLEAGWYLGAGIGRAS.....HAVSPGFPPDTRMDNQGNAYRLYGGYQFNRYLMLETAYIDLGSVKVDSPTEYARIKARAMTFGVIGMLPVWSGFSLTGKLGLASLNADAEAGTKSGSSRSESSATRATMLAGVGLRYTITPNITVRADYDHMQKTGIFARGG.KLGNHMMSLGVDYRF................
208009146.pir   NINTEFSNSTLLPDNGDYWYVGTKLGLSVYQYGC......EEWSIDCDRTDLGGGFFVGYQINESWGIELGKTFLGAAKAEYFSRKVTGDMETIDLFGKYTYGLTDRFGFFGKAGLANWNGKTKSDY......VETTTFGHDLSVGFGAQYALNKRWLTQLEYQYINSIGD..DTVGESDYHLMSIGIIYRFGFHELkpFSCPIPNERQI
270317227.pir   .....................................................NSFTYGVFGGYQinLGLAVELGYDDFGRAKgkEGRTVAKHTNHGTHLSLKGSYEVLNGLDVYGKAGVALVRSDYKFYP..IDNHKDGHRVRASGLFAVGAEYAVLPELAVRLEYQWLTRVGKYRPQdnSAIAIRSRLKLFYRPAKLSLDPFDAMKKVVFKA
319934177.pir   VLASSGFFSATANAAWSDLYFGGKIGYVNLDDACY.......VSSPCDDDSAGFGLYYGYNFSKHISAELGIDLLGEHETNFSNGYFsaKLAAYTLAPKFSLPLNEKLDAFAKIGAAYMIYG..............DDKDLVPTGSLGLEYALTESFKARAEYQRYQDMSD..DIVKDMDANFFGIGITYLFGGASSAATAAAANETAV
381362915.pir   LPIATKAPPPAIAASWAGFYLGAHGGYGWKKDDFSQSDNFTFTanGVKSQGAVYGGHAGYNWQFGRAVTGLELDFSAGDIKGSnsLAENVKYLGSARARLGWLPTDNLLLYGTAGLAWERLDVTASSLLTdiTTKTAIDKFGWVAGVGAEMMlsPNWIGRVEYLHYdrVGgeLSTAGSQTIDLVRAGVSYKFGEPakAPVAPPQASWAGF
219548647.pir   AAVISTTLLVASASANAQFYLGAKAGVSWLNNLC......TSSSSSCDEDSWALGSFLGYEVDEYVAIEGGLETLGETTGAGYKDAG...LTAYTIAPRLSFPMNEDIDLFVKAGAAFVKYGNK..............DDENLFGAVGLNYSLFENIDIQAEYQH...INDIDIDVSSFHANSFTLGFRTKFGGSspEPVQEVMVVEETv
386422621.pir   AGLALLANVANavPSNVSLYGGASAGMANQEGACSAANASSNCDDSAGGYKVFVGSRVTPQGnpTLGVEAGYLDFGASTADGKAgnFSSDLSASYLAGVGYVPVAPRTELLGKAGAAFWKQNGKREVLqtDLNIETSNSGVGLLGGGAQYKVSDNLSVRGEYEHVF..GTASDTSYESDAGMYSIGAVFSTF................
129138.pir      .....................................................................................................LTAKLGYPITDELDIYTRLGGMVWRADSKYNIP.GGASFKDHDTGVSPVFAGGLEWAVTRDIATRLEYQWTNNIGDANTVGTRPDNGLLSVGVSYRFGQQEaaPVVAPAPAPAPE
545238096.pir  VALAGFATVAQAAPKDNTWYTGAKLGWSQYHDTG.....FIPNNGPTHENQLGAGAFGGYQVNPYVGFEMGYDWLGRMPYKGDNINGAYKAQGVQLTAKLGYPITDDLDIYTRLGGMACRHQG.....................................................................................
21623226.pir    IFLLVSLVTGIQAKENDHWYLGTKMGWSDFNILEYRSKDITPFDTKIDTkaPVFGLFLGYEFNPYFSFEIENDTTGFSPHLIFQKnqNIQINSLQLATKLSYPITDDFHIYTQLGGMMFWDNLSFKKD..LQNIFTEKSRLIPNVSLGAEYIFNKKFITRLDYTWKSNIAKIMNLSMKPVLGDVALSFGWKFGKSNINEIFSSYIPQSSD
58422899.pir    .........................................................................................................SYPVMDDLDIYTRLGGMVWRADSKANFG.NGTRLSDHDTGVSPLAAVGVEYALTKNWATRLDYQWVNNIGDAGTVGARPDNGMLSVGVSYRFGQDEAAPVVAPAPAPAPV
403399504.pir   .....................................................................................................LTAKLSYPIYDDLDIYTRLGGMVWRADAKNNV..GGGDRSNHDTGVSPVFAGGVEYAWTPSIATRLEYQWINNIGDAGTVGTRPDNGMLSVGVSYRFGQDEAAPVVAPAPAPAPQ
129142.pir      .....................................................................................................LAAKLSYPIADDLDIYTRLGGMIWRADSKANYGRTGERLSNHDTGVSPLAAVGVEYALTKNWATRLDYQFVSNIGDAGTVGARPDNTMLSLGVSYRFGQDDVaPAPAPAPAPVVE
469771069.pir   AVSREFSDYRDTRQNNSHVYVGLRGGGANFEKAC......SSGSSKCNDDTLGYGFYGGYQFTSWFALEAALTDYGSISATYYGTnVSVETWGSELSGVFSYDLFESLDAYLRVGASYQHAHKESIWP.....TEELVGDWGVVSAVGLDYRLSTSWSLRGEYQFIDDTGK..NDLTHVDVHFVSLGLTYHFGQEKLFSAVAISAPLISE
469743159.pir   SAVVLTVGSPFAIAQQADIYLGTGVGVGHIHGLNH.....IEGMQSSVDEAAAANLFLGYDFNEYLSVEGGYLYVG....KGKSDEQPFQNHGATLSLNGRVPIVGDLSLIAEAGAYWATTE..........GLGEKDRKVSPMIGAGLSYQINEQFDVQARWRYIKDVADIQstRFEPNENVGMIELVYHPFRSTKNELVQPVTVQEAI
147001.pir      .....................................................................................................LTAKLGYPITDDLDIYTRLGGMVWRADAKAQVPGTGASFKDHDTGVSPVFAGGVEWAMTRDIATRLEYQWVNNIGDAKTLGTRQDNGMLSVGVSYRFGQQeVAPVVAPAPAPAPQ
159120663.pir   MGVFGVSALSVIAAHASGIYVTGQAGYANNHLGKhsRANGESPGFTMNNIGLAGRLAIGYQVNPYFATELGYLRlgRAFDnlAPATETLKQNAIDVAAKGILPVTDKLNVYGKLGVAYLTSTITSHfdNQNAWFGVKKHIFAPEAAVGASYNLTPNVFVDTSLTHIQTIGYKHKPG...NIDFATVGIGYTFG................
406980370.pir   GLPEEMPMAPAAMTSDAGIYVGINGGWG.LTNWKNFGDRTGVITNISNDNGVVGRAFVGYDFNRYFATEFGYSYFFNQPKLNAGSTTDYktQTLDLVGKGKLPVVDNFDLYAKLGVGYMMSKVKLETTTEgaNFSGNRNVFNVVYGAGADYYITPNVIANVEWLRVNGSAKFNSTdyQPYTDAFMVGLRYKFDV...............
260614446.pir   ............MSAHAEWYVGGALGQARFDDASVSGTVMGYSTtlEDDKSTIASIFGGYQFNRYFALEGSVTGIDALDGSVVTVG...DMSYIAIQPKVSLPVTDHVSLFAKAGVAYFNAEIKVSNAVAGYSgtFSDSDVTGILGLGVEVAATDNWHIRASWDYLRPELDVVNVgvETDINVFSLGVSYHFN...............
406980371.pir   VLALGLNGTALasDSNSGIYLGAQLGMSNLHYGP..SSNYTTSSTTVDNRNFAGRGYAGYAFSQFISAELGYDYYGFPKFKNTNGNtnFLQQGVDLVAKATLPLDYGFGFYIKGGLAWIHRGSLNSNS.GTFASKDANSTITPVGGLGANYWFAPNIALDLSWTKTMTVSDL......PTTDFIALGIVYKLNI...............
58422905.pir    ..........................................................................................................GYPITDDLDVYTRLGGMVWRADTKSNTP.GGESTKNHDTGVSPVFAGGIEYAITPEIATRLEYQWTNNIGDANTIGTRPDNGLLSVGVSYRFGQQEAAPVVAPAPAPAPE
478768361.pir   VLVLAGFLVAGPVVAGDGFYLGAGLGAGLVGAsgRLLESETRNYDFENEQTFAYRLFAGYAWNEYLALELAWFDYGETDVTngREDFELNTQGASLTSVLSYPLAEDLSIFGRLGAAWTKqdSVRNLGSEAWTEEVDDTHIDIAYGLGATYQFSGSWAARLEAN.....GVELENGRVFD...ATFSVVYRL...............
74056684.pir    VSAALASAMAMSAPATaaPYVGASAGESTTDSCI.........TGTCDDADTAWKIYGGLEVNEFISMEVGYVELGEVEYSAP..TGTRGTHGMILQLVGSYALGPKFTLLGRGGMNILNTEVEGAVA..GARGNEGDTDVSWSLGLGAQYNLTKSVGLRVEWERYFEVGDPDSTG.EADIDLLTAGVVFKF...............
149805963.pir   LSLLTLFRINYAQAKESSFYIDLGIGSALSANSNSYLQQKVEAISSSdKNTFAYRAQLGYQANKWVALEVGYTNLGEQTLTItaNVQLNSASGATFGGKLSYPISNWISIYTRIGGFIWHGDYDSNSSVNesSIKVSDHGTDLYYGLGVQLPLYENFSFALDWQRYE........LTKYKTDLITGAIIYKF...............
315266048.pir   QLIDEDKPQTLITLPEAYLYMGSEIGINHYQHGC......ESWSIDCDKNSTMASFFAGYQFNHHLAFEVAYIDLGKAEATYLEASkqGSMRGLNLSSVASIDFGADLALFAKAGVFNWHGENK......GPFSTIKADDWSPSFGVGMTYQLSDSWQARLQYEYFHHLGNDDIGGTNA..HATSLGISYQFGRSRPEVITSTVINSVTN
146985.pir      .....................................................................................................LTAKLGYPITDDLDIYTRLGGMVWRADSSNSIA.....GDNHDTGVSPVFAGGVEWAMTRDIATRLEYQWVNNIGDAGTVGVRPDNGMLSVGVSYRFGQEENAPVVAPAPAPAPE
U3ABG2.pir      QSSCIIALAALSHSAMADMYIAPTIGVSYFSGGC......ESHELSCDTNDISFGATLGYQYSPNIAIELGYQHYGEVEATylKADYSAKVQSAELGLAYSLPVADSLALNLIGGAALWFVDMDGKEL..SYRVNKSSNGLSPYLTTSGSYKLSEQLSVDSGLKWVSSVGSSATG..KSDIWQAFIGLTYRFPSNMSTASVPRTYDTEPL
408875342.pir   SPVVLLCGIFTPIVSHADFYIGGKIGSSNVVNSC.......EVNLNCNSESFSVGFYGGYDFDNYISAEIGYDFLGIFDSNYKQLNDNFKNnaLTFAPKFNYTLMRDLDVFGKIGAAQLLSHS..............ERDMVTVATVGLEYVVDFNLKLRGEYQYFREMRD..QDIYNLDTNVFSLGVTYSFGDSENISVEEEIVPQDAI
129134.pir      .....................................................................................................LTAKLGYPITDDLDIYTRLGGMVWRADAKNN.....EGFKDHDTGVSPVFAGGVEYAITPEIATRLEYQWTNNIGDANTVGGRPDNGLLSVGVSYRFGQQEEAAPVVAPAPAPE
145963788.pir   ARVSPLLLLSASAVGAENLYVGAKVGNVTADN....FAPLVESRTAIDDSDTSYGLFVGYHIVDTLAIEGGYNYLGEYDLK.DIAGGSYEASSFDVVLKASGNVTDSLALYAKGGFAVYDWKAN......GPGVESDDSGVTPTIAFGSEYSITSAMKVGIEYQLYQDIGGV......DIDSFNVALSHSF...............
37624554.pir    ................................................................................................................DDLDVYTRLGGMVWRADTKANVP.GGASFKDHDTGVSPVFAGGVEYAITPEIATRLEYQWTNNIGDAHTIGTRPDNGMLSLGVSYRFGQGEAAPVVAPAPAPAPE
515419509.pir   LAVSLSSVLLVTQSNAGDLYLGTSLGKSNFSLSGSEKAEFASDGVstDKSATSYKLYTGYRFNPNFALEFSYANLGELKMNAGSIHGKTEANSVGVSALGIIPIYENLEFFGKLGVHRW..DTKTKVLFDDFTETTSESGTNVHYGIGASYILNQ.IALRVEIERF........KVDKANSTLVTTGISYRF................
309371108.pir   QRASLAAFLFFSHGSIAQIYVTPSLGLSHFSGGC......SSQRIECEKSSSAVGAVLGYQAFPHWAVELGYQHYGTVNAKYPAIGssAEIQGIEFGLSRKFSFTSTLDGSLSAGMSAWFVDMDGREL..AYSVERSADGFSPYLRAELGYALSQHLRLKTGIKWISSVGSDQTG..KSEIWQPFIGLSYAFGSSsSAPSQHQEVNVSSS
550661146.pir   VAGVALSGSASMA.LAQQGYAGISLGQVEVKDFC.....DGWSGVDCDDTALSGRLYAGGFFDKYLAFEAGYRYIDETSAEGasAGIDASYHMFDSSLLVFTPEFGPVRLFAKAGLQFWrvDAKLTVLSESFNGSEHESGLAFRTGAGATIDFTESFGLRLEWDYLQNVGDDVLDNLESDIHIFSVGPEFRF................
37624574.pir    ................................................................................................................DDLDIYTRLGGMVWRADSKGNYASTGVSRSEHDTGVSPVFAGGLEWAVTRDIATRLEYQWVNNIGDAGTVGTRPDNGMLSLGVSYRFGQEDVAPVVAPAPAPAPE
159120883.pir   FFLFLLFFSVFADASIPGFYLLGQLGTADTHQEASHAAAQSINN....KMAFTGRIAGGYQFNQNVAFEIGYFRFSDVDFSGigQNVSLNQNAIDFMAKPMLPISCNVNFYAKLGLAYRRANGSAFV..NGQSYLNYSDSWNPSFGLGFSYDITPFVPIDLAWTRIQRVGGENNL...PSADFYSIGLAYYFG................
406903197.pir   NSSGSEFSSDSSSNSNSGIYFGGQIGATNLHYRGSSYTTSNSSYDDTYK..LAARGYLGYAFTQFISTELGYDYYGRPKFKNTNGNtdILQQGMDLVVKANLPLDYGFGLYIKGGMAWV.YRSALHNNANTFAEKGAGSKFTPVAGIGVNYWFAPNMALDLSWAKTMTVSDL......PTIDLITLGFIYKINI...............
U3B2E1.pir      LAIPSASLATENTAFHSHLYGGVNIGLTNWGGLGTG....VTNVTSVDDPDFAWGAFVGLHTLPWLAFELGYQNLGEADLTD..VSGSYDAQTINFTAKFSHTVVENISIYGKVGTQWYDWNANGSN......VAESDTGWTPHLGMGLEYKFNQHWSGALDYTWYNDVGG......PDINYFGIAAIYHWH...............
58422903.pir    .........................................................................................................SYPIMNDLDIYTRLGGMVWRADSDGV....GSNGGNHDTGVSPLAAVGVEYALNRDWATRLDYQWVNNIGDAGTVGARPDNGMLSVGVSYRFGQEVAAPVVAPAPAPAPV
406939085.pir   IGLSWGLFNQSLEASPVGLYMGGQLGWDNVHQGHFIakKIDNFDEIYHDTGMAERFFVGYQFNPYFALETGYTHFNSLEVNTNRNaaFVQTFAIDFVAKGILPISERFSVYGKGGLAYLNANGKVRISANaiSGNPSMNVVYPTVSIGLNYDLSQHVSADLSWNRIQQLSHRSF....PSTDFVALGFIYHF...............
V5FE49.pir      ASLILAIPTASLATESSTFHSHLYGGVNMGMTNWGGLGAGVTDVTGVDDPNFAWGAFVGLHTLPWLAFELGYQNLGEADLTD..VSGSYDAQTINFTAKFSHTVAENTSIYGKVGTQWYDWNANGSN......VAESDTGWTPHLGMGVEYKFNKHWSGALDYTWYNDIGG......PDINYFGIAAIYHWH...............
333816838.pir   GAVLLAGTVSQANA.ADNWYVEGFVGQAQVDSSRRDLQPQTAAGVVTSVddTAFGLSVGYQWTPMIAIELGYADFGNGSARIEGaeTPVLADGVMLGLRFTLLQHDAWRFEVPIGLFRWQADISSTM..GNSRLTTELDGTDWYAGVRFSYQVSDAWSVGLGYQYVDIEPN........DFLSYQLNLRYQF................
58422901.pir    ..........................................................................................................GYPITDDLDVYTRLGGMVWRADSSNNI.....SGDNHDTGVSPVFAGGVEWAMTRDIATRLEYQWVNNIGDGNTVGVRPDNGMLSVGVSYRFGqeEAAPVVAPAPAPAPE
315181920.pir   QHVLLSLLCVSVSSTAAPYYLGVKIGKSWLSEQC......QSSEENCVTDNPAYGLFAGYHVNDTLALESSYEDLGTFTVKSLH...SDTIRLLTLAPKLTFPIVDDYRWYAKAGMAYANTSGH..............SDGSWFAATGVEYDLRPQMTLRVEYSVATDVNDGTN...RYTMNGAAVGIRYAFGASNEPRSLHDAVETTSF
406940345.pir   VVVADDFEPVPLPMNEQALYLDAHLGYAQSNWTDNDTNGIIGNTNLALYNafSGGLDFGYVMMHYFALEAGWLHlgGLTVNGSQgnDSVNSWLTYGALKFLLPAGHDITLFGKVGAAYR.SLHFGLPQEIVSPASGSGNLPSNVSSNGHYWA..PVFATGVEYNNDNWlgIQYLYLPGNdiNNGAAGALQGFGAPNAAPEVNLYTGFVGY
553948569.pir   LGCLLFLSVTQQA.QATDWYVEGFIGQAKADKTLPELNAQVGEGqnVDDSDTAFGVSLGYQWTPTVALELGYADFGEGSARIKGAttPVLADGVMLGLRLTLLEHEGWRFEVPFGLFHWQADIGSTM..GNTTIKTDLDGTDWYAGVRFSYQFSDAWSVGLGYQYIDIEPN........DLLSYQLNLRYQF................
260610973.pir   SFNFAHSNDDVYNESESYFYAGQKLGWARYSNNC.......DDKVNCKIDTFGYSVFSGFQFNRWYALEGSISNYGSPDYKINSDFIESNTSgEILSKFRVLDITSHLHAYAKLGLGYQYLNQPSSY.......TNKSHYFGLLGAAGLEYDITDQWSLRAEVQHIDFKEKEDNFNQGKDVTFSSIGLSYKFGQSRTKTVFQEVEKIVEK
37624594.pir    ................................................................................................................DDLDIYTRLGGMVWRADSKANYG.NGTRLSDHDTGVSPLAAVGVEYALTKNWATRLDYQFVSNIGDAGTVGARPDNTMLSLGVSYRFGQDDVVAPVAPAPAPAPV
12382061.pir    ..................................................................................................................LDVYTRLGGMVWRADATQTNPTYG.RISNHDTGVSPLAAVGVEYAVTKNWATRLDYQWVNNIGDAGTVGARPDNSMLSVGVSYRFGQDEAAPVVAPAPAPAPV
14578714.pir    ..................................................................................................................LDVYTRLGGMVWRADSTQYN..NGTRISDHDTGVSPLAAVGVEYALTKNWATRLDYQWVNNIGDANTVGTRPDNGMVSLGVSYRFGQDDVaPAPAPAPAPAPV
```

The top layer shows the consensus sequence. Residues are coloured after their physico-chemical properties. (GPX black; positive blue; negative red; CM yellow; H dark purple; NQ light purple; all others green).

Class 3B

```
CONSENSUS      VTIDYRHEYKDTHADRLKISHRFDNGFGFSVEAKWKSVSNGTEFEASYQYKLNDNFTLQPGFSLESGSDGTTYKPYLRGQYKFDDGLYVALRYRYEYKRYSSDDGDDDDTNNDGDELGYYVGYKWLEYLYDYEHNVKLAYKIDKNWTPYVEVGNVSRQTRYRVGVQYSF
668984690.pir  VTVDLRHEFIdtNADRVSVSHRFANGFGFSVEAKWKsvGNGHEESISYQYKFNKNFSIQPGFNIESNDSRSIYKPNLRVQYSFDSGFYVAARYRYDYTRYPSNANKDDDKVNRGDAWAGYVFGDWRTElySQEYNVKVAYKIDKNWAPYGEVGNVgrQTRFRVGVAYSF
H3LCK6.pir     VTVDLRHEFIDgnADRVSVSHRFANGFGFSVEAKWKsvGNGHEESISWQWKADKNFSVTPGFNIESNDSRSIYKPNLRVQYSFDNGFYVAARYRYDYTRYPANAGKDDDKVNRGDAWAGYVFGDWRTElySQEYNLKVAYKLDKNWAPYGELGNVgrQTRFRVGVAYSF
674723206.pir  VTIDYRHEFMDthKDRLLMSHRFANGFGLSLEAKWRqvSNGTEVVASYQQNLTDEFSLQPGFSLESNSNNNGYRPFLRAQYNLTKELYVAARYRYEYKRTTSKGSDADEKTNRGDFWLGYTFLKDWQAEydYEHNVKLAYKWDKNWKPYIEVGNVkrQTRYRMGVSYSF
668961021.pir  VTVDLRHEYIdtNADRVSVSHRFANGFGFSVEAKWKSggNGHEEAISWQWKANKNFSLTPGFNIESNDSRTIYKPNLRAQYSFDNGFYVAVRYRYDYTRYPASANKEDDKVNRGDAWAGFVMGDWRTElySQEYNVKLAYKLDKNWAPYGEVGNVgrQTRLRVGVAYSF
671731105.pir  VTIDYRHEMQdtHKDRLLMSHRFGSGFGLSVEAKWTgvSNGTEVVASYVYNFNKTFSLEPGFSLDSSSDSNNYRPYLRGKVNITDDVSTSLRYRPYYKRYSKNIGTTKETSENGYNLTAVVSYKFLKDfyDISHDVKLSYKLDKNWKPYMAIGNVsrQTRYRVGVQYSF
V3HGH3.pir     VTIDLRHEYIDsnADRVAVSHRFDNGFGFSVEAKWKsvGNGHEDQISWRWKATDNIALTPAFTIESTDSRTIYKPNLHVQYSFDNGFYVAARYRYEYTRYPSSSNKDDDKVNRGDAWVGWVLGDWRTElySNEYNAKLAYKWDKNWAPYVEVGNVgrQTRFRLGVAYSF
679636521.pir  VTIDYRHEMKDthRDRLSMSHRFANGFGLSVEAKWRqvSNGTEVVASYVYNFNKTFSLEPGFSLDSSSTSNNYRPYLRGKVNITDDLSTSLRYRPYYKRNSDKIGGAGPTQENGYNVTAVVSYKFLKDfyNFDHDVKLSYKIDKNWKPYMAVGNVarQTRYRVGVQYSF
595639148.pir  VTIDYRHETQDqhKDRLLMSHRFDNGFGLSLEGKWKsvSNGTEVVASYVYKIDKTFSIEPGFSLDSSSTANSYRPYLRGKANITDDLSISLRYRPYYKRTSANINTKKDTSENGYNLTSVLSYKINKEfyDWTHDFKLSYKWDKNWSPYMAIGNVsrQTRYRVGIKYSF
640470833.pir  MSVDLRHEFMDthKNRMLVSHRFANGFGFSLEAKWKqvSNGTEAVASYQYKVTDEFSLQPGFSIESNSNNNGYRPFLRAQYNLTKDLYVATRYRYEYKRTTSKGSDADEKTNRGDFWLGYTFFDDWQTEydYEHNVKLAYKWDKNWKPYIEVGNVkrQTRYRVGVSYSF
640696592.pir  MSLDLRHEWQDthKDRMLVSHRFDNGLGFSLEAKWRqvSNGTEAVVSYQHKF.DAITLQPGFSIESSSNNNGYRPYLRAQYNFTKDLYVAGRYRYEYKRTTSKGADFDEKTNRGDFWLGYNFYKDWSAEydYEHNVKLAYKWDKNWKPYVEVGNVkrQTRYRVGINYSW
A4WE32.pir     VTVDLRHEYIDsnADRVAVSHRFANGFGFSVEAKWKsvGNGHEDQISWRWKATDNISLTPGFTLESNDSRTIYKPFLHAQYSFDNGFYVAARYRYDYTRYPSSAEKDDDKVNRGDAWLGWVMGDFRTElySEEYNAKLAYKIDKNWSPYVEVGNVnrQTRFRLGVAYSF
C6C6T4.pir     VSIDYRHEMQDthKDRLSISHRFANGFGLSSEVKWAqvSNGTEVVASYVYKFNNVFSIEPGFSLESSSTSNNYRPYIRGRANVTSDLSVALRYRPYFKRNSGSIGSSSSTTDKGYTLTGNIDYTFLKDyyDFTHNVKLSYKWDKNWKPYVEVGNVsrQTRYRVGVQYSF
KdgM.pir       VSIDYRHEMQDthKDRLLISHRFANGFGLSSEVKWAqvSNGTEVVASYVYKFNSVFSIEPGFSLESGSSNNNYRPYLRGRANVTDDLSVALRYRPYFKRNSGNIGKDNTMDKGYTLTGNIDYTFLKDYtyDITHNVKLSYKWDKNWKPYVEVGNVsrQTRYRVGVQYSF
J8T2Z2.pir     VTIDYRHEMKDthRDRLSVSHRFANGFGLSVEAKWRqvSNGTEVVASYVYNFNKTFSLEPGFSLDSASDSNNYRPYLRGKVNLTDDLSTSLRYRPYYKRISGNLNNNKNNQENGYNLTAVVSYKFLKDfyDFSHDVKMSYKLDKNWKPYMAVGNVarQTRYRVGVQYSF
H3M395.pir     VTFDYRHETNDkhKDRLLMSHRFANGFGLSLEGKWKgvSNGTEVVASYVHKINKTFQIEPGFSLDTSSSSNNYRPYLRGKANLTDDLSASLRYRPYYKRNSGNIGTSKDTSENGYNLTSVISWKFLKDyyDWTHDFKLTYKLDKNWSPYMAIGNVsrQTRYRVGVKYSF
588286361.pir  VTIDYRHEMQdtHKDRLSISHRFANGFGLSLEGKWKgvSNGTEVVASYVYKINKTFSIEPGFSLDSNSTSNNYRPYLRGKAVIVDDLSVSLRYRPYYKRTSANINTPKDTSENGYNLTSVISYKVAKDyyDWSHDLKLTYKWDKNWSPYMALGNVsrQTRYRVGVKYSF
I6SIA3.pir     VTVDLRHEYIdaNADRVSVSHRFANGVGFSVEAKWKsvGNGHEDQISWRWKATENIALTPAFTLESNDSRTIYKPNLHLQYSFDNGFYVAARYRYEYTRYPSSSNKDDDKVNRGDAWVGWVMGDWRSElySTEYNAKLAYKWDKNWAPYVEVGNVgrQTRFRVGVAYSF
583709540.pir  VTIDLRHEFIDgnADRVSVSHRFANGLGFTVEAKWRsvGNGHEDTISWRWKATSNFFLTPGFTIESNDSRSIYKPHLHVQYSFDNGFYVAARYRYEYTRYPNNAGKDDDKVNRGDAWAGFVLGDWRTElySQEYNVKVAYKLDNNWSPYGEIGNVgrQTRFRVGVAYSF
668987447.pir  VTIDLRHEYTDTskDRVLISHRFANGFGFSVEAKTKsvDNGDEYVISYQFNAMPNITFQPGFALETSSSKAIYKPYLRGQYNFEGGFYVAARYRYEYTRDTSSAKDDEHVNRGDLWLGYKTGAWTFEGNydYELDLKVAYSIDKNWKPYAQIGNVsrQTRYRVGVQYTF
668981850.pir  VTVDLRHEYIDtnADRASVSHRFENGFGFSVEAKWKsvGNGHEDQISWRWKATPNIALTPGFTVESKDSFTIYKPYLHAQYSFDNGFYVAARYRYDYTRYPSSADKDDDKVNRGDAWVGWVMGDWRTElySQEYNVKLAYKIDKNWAPYGEVGNIgrQTRLRVGVAYSF
C4S0E2.pir     VTIDYRHEMQdsHKDRLLMSHRFANGFGLSLEGKWKgvSNGTEVVASYVYKIDKTFSIEPGFSLDSSSTANSYRPYLRGKANIIDDLSVSLRYRPYYKRTSANINTKKDTSENGYNLTSVISYKLAKDyyDWTHDLKLTYKWDKSWSPYMAVGNVsrQTRYRVGIKYSF
C4T244.pir     VTIDYRHEMQdtHKDRLLMSHRFANGFGLSLEGKWKgvSNGTEVVASYVYKIDNTFSIEPGFSLDSSSTANSYRPYLRGKANIVDDLSVSFRYRPYYKRTSANINTPKDTSENGYNLTSVVSYKFFKDyyDWSHDFKLTYKWDKNWSPYMAVGNVarQTRYRVGVKYSF
G7T7S5.pir     ATLDYRHEYADrnKDRIAIIEKLPNGIGFYVDASVKsvANAIELGVSYNYKVTDNFALQPGFIFESGPDTSIYKPYLRGQYNFDSGVYMAGRYRYDYARKTANYSDDEKTNRFDTYIGYVFDELKLEYntNYEHNVALAWKLNKSFTPYVEVGNVarQTRYRVGLQYHF
C4US06.pir     VTIDYRHETQDqhKDRLLMSHRFANGFGLSLEGKWKsvSNGTEVVASYVYKIDKTFSLEPGFSLDSSSTANSYRPYLRGKANIIDDLSVSLRYRPYYKRTSANINTPKDTSEHGYNLTSVLSYKISKNyyDWSHDFKLTYKWDKSWSPYMAIGNVsrQTRYRVGVKYSF
653405761.pir  VTVDLRHEYRDdnADRVAVSHRFANGFGFTVEAKWKSgaNGHEEQISWRWRPNKNFALTPGMNLDSNDSKSTYKPFLHAQYSFDSGVYVAARYRFEYTRYPDNADKDDDKVNRGDVWVGYVLGDWRAElySEEYNLKVAYKLDKNWAPYAELGNIsrQTRYRVGVAYSF
666390879.pir  MSIDLRHEWMDthKDRMLVSHRFANGFGFSLEGKWKqvSNGTEAVVSYQFNVNDSLSLQPGFSMDSTSSSNGYRPYLRAQYNLTKELYVAARYRYEYKRTPSKGSDYDAKTNQGDFWIGYTFLNDWQVEydYEHNVKLAYKWDKNWKPYIEVGNVkrQTRYRVGVSYSW
639213623.pir  VTVDLRHEYIDsnADRVAVSHRFENGFGFGVEAKWRsvGSGHEDNISWRWKATDNIAITPGFTLESGDSKTIYKPNLHVQYSFDNGIYLAARYRYEYTRVPDGAEDDDRVNRGDAWVGFALGEWRTELNysNEYNAKVAYKIDKNWSPYFEVGNVgrQTRLRVGVAYSF
639211280.pir  VTVDLRHEYIDsnADRVAVSHRFDNGLGFSVEAKWKsvGNGHEDQISWRWKATDNIAITPAFTIESSDSRTIYKPNIHVQYSFDNGFYVAGRYRYEYTRNPSTPDREDDKVNRGDAWVGFALGDWRTElySTEYNAKLAYKIDKNWSPYVEVGNVgrQTRFRLGVAYSF
586611429.pir  VTFDYRHELNDshKDRLLMSHRFANGFGLSLEGKWKgvSNGTEVVASYVYKINPTFQIEPGFSLDSSSTSNNYRPYLRGKVNFSNDVGATLRYRPYFKRNSNNIGTAKDTSENGYNLTAAVSWKIFGDyyDWTHDLKLTYKLDKNWSPYIAVGNVsrQTRYRVGVKYNF
560881858.pir  VSIDYRHEMQDthKDRLTIAHRFANGFGLSSEVKWAqvSNGTEVIASYVYKFNNTFSIEPGLSLESGSSGNNYRPYIRGRANVTDDLSVALRYRPYFKRNSGNIGKDNTTDKGYTLTGNIDYTFLQAYtyEITHNVKLSYKWDKNWKPYVEVGNVarQTRYRVGVQYSF
654081671.pir  VSIDYRHEMTdgHKDRLLISHRFANGFGLSSEVKWAqvSNGTEVVASYVYKFNDVFSIEPGFSLESGSSNNNYRPYLRGRANVTDDLSVALRYRPYFKRNSGSINNTKGDSTMDKGYTLTGNIDYNFLdyDITHSVKLSYKWDKNWKPYVELGNVsrQTRYRVGVQYSF
668974313.pir  VTVDLRHEYIDsnADRVAVSHRFDNGLGFGVEAKWKtvGNGHEESINWRWKATDNFALNPGFNIESKDSFSIYKPYLHAQYSFDNGFYVAARYRYEYTRVPASGGVDDKVNKVDTWVGWAMGDWRVELNysTEYNGKLAYKWDKNWSPYFEVGNVgrQTRLRVGVAYSF
H2J256.pir     VTIDLRHEWLDdhKDRALVSHRFANGFGFSLEAKWKsvDNGTESTISYQVKVTPEVFVQPGFTLESSSDSSIYKPFLTAGYTFDSGIYFNARYRYEYTRYTKENTEDRKTNRGEIWLGYKMADWRFEYNydYEQNLKVAYNINKQWTPYAEIGDVsrQTRLRLGIQYNF
515508199.pir  VSIDYRHEMQDthKDRLTIAHRFANGFGLSSEVKWVqvSNGTEVVASYVYKFNNTFSIEPGFSLDSASSGNNYRPYIRGRANVTDDLSVALRYRPYFKRNSENINNSDPKKSTMDKGYTLTGNIDYTFlyEITHNVKLSYKWDKNWKPYVEVGNVsrQTRYRVGVQYSF
M1JBI6.pir     VTVDLRHEYIDsnADRVAVSHRFDNGFGFSVEAKWKtvGNGHEDQISWRWKATDAIAITPAFTIESVDSKTIYKPNIHVQYSFENGFYVAARYRYEYTRVPDGKDDDDKVNRGDAWVGFALGDWRTELNysNEYNAKVAYKIDKNWAPYFEVGNIgrQTRLRVGVAYSF
K7ZXR2.pir     VTFDYRHETNDshKDRLAMSHRFASGFGLSLEGKWKgvSNGTEVVASYVHKLNDSFQIEPGFSLDTGSSYNNYRPYLRGKANLTDNISASLRYRPYFKRYSTNIGTQKNTSENGYNLTSVLSWKFLKDyyDWTHDFKLTYKFDKNWSPYMAIANVarQTRYRVGVKYSF
679616777.pir  VTIDYRHEMQDthKDRLSMSHRFANGFGLSAEAKWKqvSNGTEVVASYVYNFNKTFSLEPGFSLDSSSDSNNYRPYLRGKVNITDDLSTSLRYRPYYLRKSGNTNKTGASNYTQETGYNLTAVVSYKFLkkIEHDVKLAYKLDKNWKPYMAVGNVvrQTRYRVGVQYSF
C6CIH7.pir     VTFDYRHEMKDthRDRLLISHRFENGFGLSSEVKWRqvSNGTEVTASYLYKFDKMFSVEAGFNLVSNSTSNEYRPYLKGGVNFTDDIYYTLRYRPAYKRFSDNINRTDPTTMKGYTITSVLGYKFLNNFtnFTHDVKLAYKIDKNWTPYVQVANVerQTRYRVGVQYNF
C4SD12.pir     TTIDYRHEMKDthKDRLLISNRFANGFGLSLEGKWGqvSNGTEVVASYVYQFNKTFQLEPGFSLESSSDSNNYRPYLRGKVAFTDDFSTSLRYRPYFKRNQPSQTKSDEKGHEFTMLFAYNFLKSYSAEyeWSHDLKVAYKWDKNWKPYVAIGNVarQTRYRVGVQYSF
J7KRZ8.pir     VTIDYRHEMQDthKDRISMSHRFANGFGLSVEAKWKnvSNGTEVVASYVYNFNKTFSLEPGFSLDSNSTSNNYRPYLRGKVNLTDDLSTSLRYRPYYKRNSGSIGGRNTSENGYNLTAVVSYKFLKDFQvkIDHDVKLSYKIDKNWKPYMAIGNIarQTRYRVGVQYSF
D4E449.pir     VTLDVRHEWFDdhKDRALVSHRFANGVGFSLEAKWKsvSNGTESTISYQYKLNPQWFIQPGFTLESTDDKSIYKPYFTSGYTFDNGIYLNGRYRYEYTRENEADKNDVKTNRGEFWLGYRYQNWRFEYNydYEYNFKTQWSYDAHWAPYAEVGNVsrQTRLRVGVQYNF
C4UNB2.pir     ATLDYRHEYADrnKDRIAIIETLPNGIGFYVDASVKsvTNAIELGASYNYKITDRITLQPGFIFESGQDTSIYKPYLKVQYNLDSGVYIAGRYRYDYARKTAYNNDDEKTNRFDTYIGYTFDKLNLGYntNYEHNVALSWKLNKSFTPYAEVGNVarQTRYRVGLQYNF
R9VQ75.pir     VTVDLRHEYIDsnADRVAVSHRFDNGLGFGVEAKWKsvGNGHEESINWRWAATQNFALTPGFNIESKDTNSIYKPYLHVQYSFDNGVYIAGRYRYEYTRNPDSNTVDNKVNKFDTWVGWAMGDYRIELNysNEYNAKVAYKLDKNWSPYVEVGNVgrQTRFRLGVAYSF
647647191.pir  VTIDYRHEMKDthKDRLLMSHRFDNGFGLSMEGKWGqvSNGTEVTASYLYKFNKTFGLETGLNMVSTSDSNNYRPYLKGTANITDSLYYGLRYRASYLRINSKVGSPEPTDMTGYTITSTLGYKLPANfyGLTHNVKLAYKYDKNWTPYAEVGNVirQTRYRVGVQYNF
D4F2U0.pir     TTLDFRHEYADthKDRIAIIQSFANGIGFYVDASVKsvTNAVEMGLSYNYQLNQNISLQPGIIFESGSDTAIYKPYLRAQYRFDNGLYLAGRYRYDYARKTQDGSDDEKTNRLDGYIGYNFNSARVEYNynYEHNVAFSYRINEMFTPYIEVGNVarQTRYRVGLQYHF
671729801.pir  VTIDYRHEMKDthKDRLLMSHRFDNGFGLSMEAKWGqvSTGTEVTASYQYKFDKTFGLEAGLNMVSSSKDNNYRPYIKGTANITDSLYYGLRYRASYLRKSDSIGTTAPSDMKGYTITSTLGYKLPANfyDMTHDVKLAYKFDKNWTPYVQVGNVsrQTRYRVGVQYNF
639183715.pir  TTLDFRHEYADshKDRIAIIQSLDNGVSFYVDASVKsvTNAIEMGLSYNYKIDQNISLQPGIIFESGNDNAIYKPYLRAQYSFDNGLYLAGRYRYDYARKTQDGSNDETTNRLDGYIGYRFADARLEYNynYEHNVAFAYKINQTFTPYIEVGNVarQTRYRVGLQYNF
V5A981.pir     VTVDLRHEYIDsnADRVAVSHRFDNGLGFGVEAKWKsvGNGHEESINWRWAATKNIALTPGFNIESKDTFSIYKPYIHAQYSFDNGVYIAGRYRYEYTRNPDSNVVDNKVNKFDAWVGWAMGDFRTELNysNEYNAKLAYKLDKNWSPYVEIGNVgrQTRFRLGVAYSF
679636660.pir  VTIDYRHEMQDthKDRLLMSHRFDNGFGLSLEGKWGqvSNGTEVTASYQYKFDKTFGLEAGLNMVTTSDDNNYRPYLKGTANITDSLYYGLRYRASYLRKSNNIGNTSTAANPTDIKGYTITSTLGYKlyGLTHNVKLAYKYDKNWTPYAEVGNVvrQTRYRVGVQYNF
C4U2C7.pir     TTIDYRHEMKDthKDRLLISNRFANGFGLSLEGKWGqvSNGTEVVASYVYKINKTFQVEPGFSLDSTSDSNNYRPYLRGKVSFTDDFSTSLRYRPYYKRNQPAQTKKTEKGHEFTMLFAYNFLKDYSAEyeWAHDVKIAYKWDKSWKPYVAIGNVprQTRYRVGVQYTF
656055859.pir  VTIDMRHEWLDVskDRVAISHRFDNGFGFSLETKWKsvSNGTESSVSYQYKFTPQWFIQPGFTLESSESNSTYKPYLTVGHTFDNDIYLNGRYRYEYTRETKKGKDDVKTNRGEFWLGYRYQDWRLEYNydYEHNLKVLWNYDASWAPYTEVGNVsrQTRLRIGLQYKF
J8TJ96.pir     VTIDYRHEMKDthQDRLLMSNRFDNGFGLSMEAKWKqvSNGTEVTASYLYKFDKTFGLEGGLNMVTSSDDNNYRPYIKGTANITDSLYYGLRYRASYLRRSNKITTTEPTDMKGYTITSTLGYKLPANfyGLTHNVKLAYKYDKNWTPYAEVGNVirQTRYRVGVQYAF
D0KCR6.pir     VTIDYRHEMKDthRDRLSISHRFDNGFGLSAEAKWRqvSNGTEVVASYVYKINKTFQVEPGFSLDSNSTSNNYRPYLRGRAAVTDDFSVSFRYRPYYLRNSGLRVGQSNNDPSVEKGYNLTTVLSYKFlyDFTHDVKLAYKWDKNWKPYMAVGNVarQTRYRVGVQYSF
J7KZ74.pir     VTIDYRHEMNddHKDRLLMSHRFDNGFGLSLEGKWGqvSNGTEVTASYLYKFDKTFGLEGGLNLVSTSTSNNYRPYIKGTANITDSLYYGLRYRASYLRVSDDIGKSSVTNPTDIKGYTITSTLGYKLpyGLTHNVKLAYKYDKNWTPYAEVGNVvrQTRYRVGVQYNF
C6DGZ3.pir     VTIDYRHEMKDthKDRLLMSHRFDNGFGLSMEAKWGqvSNGTEVTASYLYKFDKTFGLEAGLNMVSNSSSNNYRPYIKGTANITDSLYYGLRYRASYLRVSGDIGKSSVTNPTDITGYTITSTLGYKLpyEMSHDIKLAYKVDKNWTPYAQIGNVkrQTRYRVGVQYSF
518683377.pir  VTFDYRHEMFDthKDRLLISHRFDNGFGLSSEVKWKqvSNGTEVTASYLYKFNKMFSTEFGFNLVTESSSNNYRPYIRGGVNFTDNLYYTLRYRPFYKRYSGSINDPKGPAEATNMKGYTITSVLGYKfdNFTHDVKLAYKIDKNWTPYIQLANVerQTRYRVGVQYNF
648420117.pir  TTIDYRHEMKDthKDRLLLSNRFANGFGLSLEGKWGqvSNGTEVVASYLYKFNNTFSLEPGFSLESSSDSNNYRPYLRGKVNITDDLSASLRYRPYFKRNQPAETKTDENGHTLTMVLGYKFLESYTVEyeWSHDIKLAYKWDKNWTPYVQVANVarQSRFRVGVAYNF
497961120.pir  VTIDYRHEMQDthKDRLLMSHRFDNGFGLSMEAKWAqvSNGTEVTASYLYKFDKTFGLEGGLNMVSTSNNNNYRPYIKGTANITDSLYYGLRYRASYLRNSNNIGNPNSATNPTAITGYTITSTLGYKlyEMSHDIKLAYKFDKNWTPYAQIGNVkrQTRYRVGVQYNF
515508059.pir  VTFDYRHEMNDthKDRLLISHRFDNGFGLSSEVKWKqvSNETEVTASYLYKFDKTFSLESGFNLVSSNTYSNYRPYIKGGVNITNDLYYFLRYRPSYKRYSGNIGGTDTTMKGYTITSTLGYKFLNNFtdNFTHDVKLAYKIDKNWTPYIQLANIdrQTRYRVGVQYNF
D0ZE45.pir     TTIDFRHEYADSskDRIAVIHNFASGVGFYVDASVKsvSNAIEMGLSYSHKLSDNITLQPGIIFESGTDIAIYKPYLRAQYSFDSGIYIAGRYRFDYARKTQDGAHDEQTNRLDGYLGYRFNDWRVEYDynYEHNVALSYRINPSFTPYVEVGNVarQTRYRVGLQYNF
C4U633.pir     TTIDYRHEMKDthKDRLLFSNRFANGFGLSLEGKWGqvSNGTEVVASYVYPFNKTFQIEPGFSLESSSDTNNYRPYLRGKVAFTDDFSTSLRYRPYYKRNQPAQTKNTEKGHEFTMLFAYSFLKNYSAEyeWTHDLKIAYKWDKNWKPYVAIGNVarQTRYRVGMQYSW
D2C0Q8.pir     VTFDYRHEMLDthKDRLLISHRFDNGFGLSSEVKWKqvSNGTEVTASYLYKFNKMFNTEFGFNLVTDSTGNTYRPYIRGGVNFTDSLYYTLRYRPFYQRMDDKITTSGADTTMKGYTITSVLGYKFLDndNFTHDVKLAYKIDKNWTPYIQLANVerQTRYRVGVQYNF
C6C5T7.pir     VTIDYRHEMKDthRDRLLISHRFDNGFGLSSEASWKnvSNETEVIASYLYNVNKTFSLESGLSYVTSSTSANYRPYVRAKANVISDLTMSLRYRPYFKRYSSNIGVSGKDTSESGYAITGNIDYTFLKDynFDHNVKLSYKWDKNWKPYMEVGNVarQTRYRVGVQYNF
G9Y108.pir     VMVDLRHEYLDDsyDRVYISHRFDNGFGFAVEAKTRsgDNGDEYTLSYQFS.TSDFVIQPGFVLETASSTAIYKPYLRVQYNINDQWWVAGRYRYEYSRNTDSGSEVDKDDGHMNRGDVWLGYNVGNLgdDYEYNFRTSYKID.NITPYVEVGNVsrQTRFRVGMAYTF
654082093.pir  VTFDYRHEMKDthQDRLLISHRFENGFGLSSEVKWKqvSNGTEVTASYLYKFNKMFNAEAGFNLVSDNTGNKYRPYIKGGVNFTDDIYYTLRYRPFYQRYSSTINAYDRNGNPAEATNMKGYTITSVLGynLTHDVKLAYKIDKNWTPYVQVANVerQTRYRVGVQYNF
E0SBW0.pir     VTFDYRHEMKDthQDRLLISHRFENGFGLSSEVKWKqvSNGTEVTASYLYKFDKMFNVEAGFNLVTDSSTNSYRPYIRGGVNFTDSLYYTLRYRPFYKRYSGSINTTPPAEATNMKGYTITSVLGYKFLdnLTHDVKLAYKIDKNWTPYIQVANVerQTRYRVGVQYNF
U6ZI98.pir     VTFDYRHEMKDthQDRLLISHRFENGFGLSSEVKWKqvSNGTEVTASYLYKFNKMFNAEAGFNLVSDNTGNKYRPYIRGGINFTDNLYYTLRYRPFYQRMDDKILTSGADTTMKGYTITSVLGYKFLDndNLTHDVKLAYKIDKNWTPYIQVANVerQTRYRVGVQYNF
G7LMQ7.pir     VTIDYRHEIKDDskDRLLMSHRFASGFGLSAEVKWKgvADGTEVVASYQYKINSTFAIEPGFSLESSSNSQNYRPYIKGSAKITDGFSASLRYRPWYKRLNGNIGKGDDVDTVQRGHQGNLVLDYKFLEkdYLYEFKLAYAWDKNWKPYTAIANVsrQTRFRVGIQYNF
V3U140.pir     VSIDYRHEWRDTarDRLLISNRFANGFGLSSEVSWKqvSNETEVIASYLYKFNQTFSLESGFSLVSGSTYRTYRPYLRGGVKFADDWTATLRYRPYYKRISGNIGSSSNTAENGYNITANVGYTFLKNyyNADHQVKLSYKWDKNWKPYISLSNLarQTRYRVGVVYTF
R8WJ46.pir     GYVDYRHEYYDdnYDRVYMSHRFATGFGVAVEAISRseSNGNEYTASYQFTW.KDLVWQPGIAIETGDNIAIYKPYIRVQYNINDNWWTAFRYRQEYMRRNTDGKDDRMVYRPEAWLGYNINDWMFELndDYEYNFRVAYKIG.SWVPFVEVGNVsrQTRYRVGLGYNF
```

C0AU33.pir	TTLDFRHEYADsnKDRVAFIHSFSNGVGFYIDASVKsvNNAIEMGLSYNYKLNNHITLQPGLIFETVTDTSIYKPYLKAQYNFDNGLYIAGRYRFDYARKTKQGVDDEKTNRLDGFIGYKYNKFKVEYDynYEHNVALSYQINSTFTPYIEVGNMarQTRYRVGLQFHF
M7CYE3.pir	TTLDFRHEYSDSskDRIAFIHSFSNGIGFYIDASIKsvNNAIEMGLSYNHKINNNITLQPGLIFETVTDTSIYKPYLKAQYNFDNGLYVAGRYRFDYARKTKQGVDDEKTNRFDTFIGYKYSKYKVEYDynYEHNVSFSYQLNDIFTPYIEVGNVarQTRYRLGLQFHF
V1HXE1.pir	VYVDVRHEYLDDsyDRAYISHRFANGFGFAIEAISKseTQGNEYTISYQFK.TGDVAWQPGFVLETGDGYSTYKPYFRATWTLNESWWVGARYRFEYVRRSSDIRDDDTINRMDVWAGYKWNNFDWTIedNYEYNFRTAYIID.QWSPFVEVGNVsrQTRFRVGIGYTF
640533557.pir	VYVDVRHEYLDDsyDRAYISHRFDNGFGFAIEAISKseVQGNEYTASYQFS.TGAVAWQPGMVLETGNGYSVYKPYFRATWKLDDSWWLGARYRYEYIRRSSDIRDDDTINRMDVWAGYKWNKFDWTLenNYEYNFRTAY.LTGNWSPFIEVGNVsrQTRFRIGVGYTF
V2IH50.pir	GYVDYRHEYYDdnYDRVYMSHRFATGFGVAVEAISRseSNGNEYTASYQFTWQ.DLIWQPGIAIETGDNIAIYKPYIRVQYNINDSWWTAFRYRLEYMRRNSDGKDDRMVYRPETWLGYNINNWMFELneDYEYNFRVAYNIG.SWLPFIELGNVsrQTRYRVGLGYNF
514987866.pir	.....RHEMKDthKDRLLISNRFANGFGLSLEGKWGqvSNGTEMVASYVYKFNNTFQIEPGFSLDSTSDSNNYRPYIRGKVAFTDDFSTSLRYRPYYKRNQPAQTKSTEKGHEFTMLFAYNFLKDYSAEyeWAHDVKIAYKWDKNWKPYMAIGNVprQTRYRVGIQYSF
585356513.pir	VYVDVRHEYLDDsyDRAYISHRFANGFGFAIEAISKseVQGNEYTVSYQFK.TGEFAWQPGFVLETGNGYSNYKPYLRATWTLNESWWVGARYRFEYVRRSSDAREDDTVNRMDAWVDYKWNNFDWTIedNYEYNFRTAYAIG.QWTPFVEVGNVsrQTRFRVGLGYTF
640697039.pir	VYVDVRHEYLDDsyDRAYISHRFANGFGFAIEAISKseAQGNEYTASYQFN.NGNLATQPGLVLETGNGYSVYKPYLRTTYTINDSWWIAGRYRFEYIRRSSDSREDDTINRVDAWVGHKINKFEFVTesNYEYNFRTAYSID.AWTPFVEVGNVsrQTRFRVGLGYTF
640473027.pir	VTLDYRQEYTdtHKYRFLITHRFDSGLGFGLEAMWKnvSNGTEATLSYQFAATDKLNLQPVFVMQSGTSHTGYKPGLYGSYKFTNDFSAALRYRWEYIRYTTPGVDDDYVNRADVWLFYKMASWAFEYNydYEHSVKVQYNIDKNWGPYATILNKsrQTGFRLGVQYRF
498991530.pir	.MIDYRHEIMdtHKDRLLVSHRFSSGFGLSLEGKWKgvSNGTEVVASYVHKVNNRLQMEPGFSLDSGSSSNNYRPYLRGKLLIQDGLTASLRFRPYYKRYSGNIGSEHKTSEKGFNLTGVISLAGLGNshEWAHDIKLTYKLNSSWSPHIAVGNVsrQTRYRVGVSYLF
668715321.pir	GYVDYRHEYYDdnYDRIYMSHRFATGFGVAVEAISRseSNGNEYTASYQFTWQ.DLIWQPGIAVETGDNITIYKPYIRVQYNINDSWWTAFRYRQEYARRNADGKDDRMVYRPEAWIGYNIADWMFELneDYEYNFRVAYKFG.SWVPFAEIGNVsrQTRYRVGIGYNF
668978780.pir	GYVDYRHEYYDdnYDRVYMSHRFGTGFGVAVEAVSRseSNSSEYTASYQFTWQ.GFVWQPGIAVEMGDNMSIYKPYLRVQYNINDNWWAAFRYRTEYTRRNADGKDDRMVYRPEMWLGYNIDNWMFELneDYEYNFRVAYKID.SWVPFVEVGNVsrQTRLRVGLGYNF
639209310.pir	GYMDYRHEYYDdnYDRIYMSHRFATGFGVAVEAISRseSNGNEYTASYQFIW.RGFIWQPGIAVETGDDIAIYKPFIRVQYNIDDNWWAAFRYRNEYTRRNADGRDDRTVYRPEVWLGYNIGSWMYELneDYEYNARVAYKIG.SWVPFIELGNVsrQTRYRVGLGYNF
635759348.pir	GYVDYRHEYYDdnYDRVYMSHRFGTGFGVAVEAVSRseSNSNEYTASYQFTWQ.GFIWQPGVAVEMGDDNAIYKPYLRVQYNINDSWWTAFRYRTEYTRRNADGKDDRMVYRPEAWLGYNIENWMFELneDYEYNFRVAYNID.SWIPFVEVGNVsrQTRLRVGLGYNF
635729153.pir	GYVDYRHEYYDdnYDRVYMSHRFASGFGVAVEAISRseTNGNEYTASYQFTWQ.DLVWQPGLAIETDEGQAIYKPYLRVQYNINDSWWTAFRYRFEYTRRNSEGRDDRMVYRPEAWLGYNLHDWMFELneDYELNFRVAYKIG.SWIPFAEVGNVsrQTRYRIGIGYTF
639143704.pir	VYVDVRHEYLDDsyDRAYISHRFGNGFGFAIEAISKseAQGNEYTASYQFN.SGPLAIQPGLVLETGNGYSVYKPYLRTTYAINEAWWVAGRYRFEYIRRSSDSREDDTINRLDAWVGHKTNNFEFVIesNYEYNFRTAYNID.AWTPFVEVGNVsrQTRFRVGLGYTF
J3GNI2.pir	ASINYRHQYTEdhADRIKFNYRLDSGLGFEAEMKYRtvNNGHEFTVSYNYKLSAQSTLTPAFQMDSSKDSTTYKFGLKYNYKINDAFYVATRYRLDAKKLDRDQVNEDMPDHIKDDQTTNRFEGWLGYtsDYEQNLIIKYKLTKKWAPFMEIGDVkrQARWRLGVQYNF
648211627.pir	AYINYRHQYAEshADRVKFGTRLDSGWGFEGELKYKtvGNGHELTVSYQHKLNDKWLLTPSLALESIERSTAYKMGLKVGYKVTDQLTLSGRYRYDSIKLDRDKVNKDMPDNQMNDQSINRYDVWVNYAtdYEQNAAFKYKWDKNWVPFFEVGDIkrQLRLRVGVQYNF
I0QUM4.pir	LTIDLRHEWLDDskDRILLSHRFDNGIGISFEDKWRsvGNGTETTLTYQYKVTPALFLQPGFAVESNTGNNIYKPSLSAGYTFSNGVYFNTRYRYEETRYSNDTSSKKTNRGEIWLGYRLNNWRLEYNYidYEEDLKLAYNLTKHWTPFAEIGNVsrQTRFRVGIQYAF
S6IMR0.pir	ANINYRHQFTEdhADRIKLGYRLDSGLGFEAEMKYRtvNNGHEFTVSYNYKLSPQSTLTPAFQMDSSKDSTTYKFGLKYNYKINDAFYVATRYRLDAKKLDRDQINEDLPDRAKDNQTNTNREFGWLGYtsDYEQNMIVKYKLTKEWVPFIEIGDVkrQARWRFGIQYNF
F3K151.pir	AYINYRHQYAEshADRVKFGSRLDSGWGFEGELKYKtvGNGHELTVSYQYRLDDKWLLTPSFALESIEKSTAYKMGLKLGYKVNDQLSVSGRYRYDSIKLDRDKVNHEMPDNQMDDQSINRYDVWVNYAsdYEQNAAFKYKWDKHWVPFFEVGDIkrQLRLRVGVQYNF
656056203.pir	VYVDVRHEYLDDsyDRAYISHRFASGFGFAIEAISKseTQGNEYTANYQFNLG.SFAVQPGVVLETGSGYSVYKPYLRTTYTINDSWWLSGRYRFEYIRRSSDERSDDTINRVDLWAGYKINNFEFITesNYEYNFRTSYTVD.SWTPFVEIGNVarQNRFRIGIGYTF
668995843.pir	GYVDYRHEYYDdnYDRVYMSNRFATGFGVAVEAISRseSNSAEYTASYQFEW.KGFIWQPGIAVEMGDDMAIYKPYIRVQYNINDSWWIAFRYRTEYARRNGDGRDDRTVYRPEAWLGYNIENWMFELndDYEYNFRVAYNID.SWIPFVEVGNVsrQTRYRIGLGYNF
D4E9M0.pir	TYVDIRHEYLDDsyDRVYISHRFDSGFGFALEAISKseSQGNEYTANYQFKYH.GVDIQPGLVLETGNGYSVYKPYIRATKVLNESWWIAGRYRFEYFRRSSDQREDDKINRIDLWAGYKVNSWDFSVeyNYEYNFRTAYHID.AWSPFIEIGNVsrQTRFRVGLGYTF
635730670.pir	SVIDLRHDYFDdnKDRIYFAHRFDNQFGFSVETRYKSsnNGSEATISYRFILNPKMYLIPAFNIDSNSGNTIYKPNLLGTVLGNGFYLNLRYRYDYTLQDNEDLNNKATGRYDAWLGYKTGDWKFEYNfdYEHDMKVAYSVTKNWAPYIEIGNVsrQTRFRIGFQYTY
516062260.pir	VTLDIRHEWLDdhKDRILISHRFANGIGLSFEDKWRsvGNGTETTLTYQYKATKSFFLQPGFAAEVNSGNKVYKPSLAAGYTFSSGIYFNTRYRYEETHYDNGNNDKKTHRGEIWLGYRLADWRFEYNyidYEEDFKVAYNINHHWTPYAEIGNVsrQTRLRVGIQYSF
515554885.pir	ASLNYRHQYTEehADRIKLNYRLDSGLGFEAEMKYRtvNNGHEFTVSYNYKLSAQSTLTPAFQMDSSKDATAYKFGLKYNYKIDDAFYVATRYRLDLRKLDRDQVNKDMPDHYKDDQTTHRFEGWLGYtsDYEQNLIVKYKLTKQWAPFMEIGDIkrQARWRLGVQYNF
653590596.pir	ASINYRHQFTEehADRIKLNYRLDSGLGFEAEMKYRtvNNGHEFTVSYNYKLSAQSTLTPAFQMDSSKDSTTYKFGLKYNYKINDAFYAATRYRLDAKKLDRDQVNKDLPDHIKDDQTTHRFEGWLGYtsDYEQNLIVKYKLNKEWAPFMEIGDVkrQARWRLGIQYNF
S6HU02.pir	ASINYRHQMTEehADRIKLGYRLDSGLGFEAEMKYRtvNNGHEFTVSYNHKLSPQSTLTPAFQMDSSKDSTSYKFGLKYTYKINDTFYVATRYRMDMKKLDRDQVNSDLPDHIKDDQDTHRIEGWVGYtsDYEQNLIVKYKINKQWAPFMEIGDVkrQARWRLGVQYNF
653580248.pir	ASINYRHGYTEdhSDRIKLNYRMDTGLGFAAEIKYKtvNNGHEFTVDYNYKLSPKSTLTPAFQMDSSKDATTYKFGLKYNYKISDTWYVATRVRHDARNLDRDQIDHSKADRAKDNQNTTRLEGWLGYtsDYEQNLIVKYKLNKQWAPFMEVGDIkrQARWRLGIQYNF
676254746.pir	ASINYRHSFTEdhSDRIKLSYRMDSGLGFAAEVKYKtvNNGHEFTVDYNYKLSAKSTLQPLFIMDSVKDATTYKFGLKYTYKIDDAWYVATRIRHDARRLDRDVIDRSKDDRAKDNQNTTRFEGWLGWtsDYEQNMVVKYKINKQWQPFMEVGDIkrQARWRVGIQYNF
639231041.pir	ASLDARQEYKHGsaSRIKMSGSVDnfFGAEMKQKGKpeAADNEFEYGYRFKLNDQWMIQPSMPVTFGSDSVTYKPQVRVQYSFESGLKAKLRYRHEFRDYTSDSSNDNRNRSKITGNLDYNWNAFQFGfyEWDYNLKVGYKedWNWRPYVEFGNVqrQLRSRVGITYSF
T0QW98.pir	TTLDYRHEYLDdhADRARVSHRFDNQLGFAVEAKWGSkaKGHEFELNYQYKVGQKLTLSPAFVLDSSSSSTTYKAQLKGTYQLTDKLYGAVRYRYGLQNYTSVSTGDRHFNQGNFQLGYQFGWGKVEYdnTYLHNLVVQVPVNKEWVPYVELGYVpfQVRYRMGIKYNF
J3D3P9.pir	ASIDYRHGYTEdhSDRVKLNYRMDSGLGFAAEIKYKtvNNGHELTVDYNFKLSPKSTLTPSFQVDSSKDATTYKFGLKYNYKISDTWYVATRLRLDAKSLDRDQIDHSYSDRAKDNQNTTRFEGWLGYtsDYEQNLIIKYKINKQWAPFMEVGDIkrQGRWRLGIKYTF
F2KCJ9.pir	GYINVRHQYAEqhADRAKFGMRLDNGIGLEGELKYKtvGSGHEFTVNYQHKIDDRWTLTPSFAMESDEESTTYKLGMRLGYKITKELSIAGRYRYDSSKLDRDKVDHDVPDNGQDDQKINRYDVYVNYAddYEQNAVVKYKWDKRWAPFFEVGDIkrQLRLRVGIQYNF
655483195.pir	TTIEYRHEYRDghSDRVKAFIDTGKNIGFELDARYNntMNGSELSIFYYDKLNDNTVGLVGASLDYNPDGLVYVPYVRLNYAFDNGFRVQGRYKWKLWDYSQTGTDGQSYHSKIQQGDAWLGYKTGNWdtNYLNNVRFMYSINttWRPFIEIGDVsrQLRYRVGIKYTW
516228497.pir	ASLDARQEYKHGsaSRIKMSGSVDnfFGAEMKQKGKpeAADNEFEYGYKFKISDHWLIQPSMPVTFGSDSVTYKPQVRVQYSFDSGVKAKLRYRHEFRDYTSDSSNDNKNRSKVTGNIDYNWNAWQFGfnEWDYNLKVGYKedWSWRPYVEFGNVqrQLRSRVGITYSF
654439426.pir	ASINYRHQFLEdhADRVKLSYRMDNGWGFEGELKYRtvSSGHELTTSYQYKPNAVSTWQPAIQLDSNENGTTYKFGVRYTHRLSDQFYVAARYRFDALKLNRDRIDEEVPDRGSDNRNTQRYEGWLGYtsDYEQNLVVKYKWDKSWAPFVEIGDVrrQMRWRIGVQYNF
655681749.pir	TTVEYRHQYLDghADRFKAFLDTGKNIGFELDARYSNkmNGSEFSAFYYKKLNKNIVGLAGTSLDFTSAGLVYVPYVRLNYAFDNGFRVQGRYKWKLWDYGQQGTDGNSYNSKIQQFDTWLGYKTGDWdtDYLHNVRVMYSigENWRPFVEVGQVkrQLRYRIGIKYTW
551320067.pir	TTIEYRHEYRDghSDRVKAFIDTGNNIGFELDARYNnsMNGSELSIFYYDKLGENAVGLLGASLDFNPNGLVYVPYARLNYSFDNGFRVQGRYKWKLWDYSQDGTDGESYHSKIQQLDGWFGYKTGNWdtNYLHNVRFMYSINtlWRPFLELGDVsrQLRARVGIKYTW
Q6LKZ4.pir	TTVEYRHEYKDghSDRVKAFIDTGKNIGFELDARYNntMNGSELSIFYYTKLKPNLTGVFGSSLDFNPDGLVYVPYARLNYSFDNGFRIQGRYKWKLWDYSQEGTDGNSYHSKIQQFDTWLGYKTGDWdtDYLHNVRLMYAFneKWRPFIELGQVkrQLRYRVGIKYTW
U7RJF7.pir	ASINYRHGYTEdhSDRIKLSYRKDSGLGFAAEIKYKtvNNGHEFTVDYNYKMSPRSTWTPAFQMDSSKDATTYKFGLKYNYKLSDTWYVATRVRHDARSLDRDQVDHSKADRAKDNQNTARLEGWLGYTpdYEQNLIIKYKLNKQWAPFMEVGDIkrQARWRLGIQYNF
654512417.pir	ASIDYRHQYTDghADRIKLNYRLSSGLGFEAEMKYRtvNNGEEFTVSYNYVLSPQSTLTPAFQMDSSSDATTYKFGLKYKYKVNDRYYVATRYRLDTKKLNRDAIDEDMPDNAKNDQHVNRFEGWLGYtnDYEQNLIVKYALTKQWVPFMEFGDIkrQVRWRLGVVYNF
659046544.pir	VTIDYRHEMQdtHKDRLLMSHRFANGFGLSLEGKWKgvSNGTEVVASYVYKINNTFSIEPGFSLDSNSTSNNYRPYLRGKAVIVDDLSVSLRYRPYYKRTSANINTPKDTSENGYNLTSVISYKIAKDyyDWSHDLKLTYKWDKNWSPY....................
573000875.pir	GYINYRHQYAEshSDRVKFGTRLDSGWGFEGELKYKtvGNGHELTISYQHKLNDRWTVTPSLALESIEKSTSYKTGIKVGYKVNDALSISGRYRYDSIKLDRDKINESMPDNAMDNQSINRYDIWINYStdYEQNAAFKYRWSKQWVPFFEIGDIkrQLRLRVGIQYNF
604183594.pir	ATLDYRHEYADrnKDRIAIIEKLPNGIGFYVDASVKsvANAIELGVSYNYKVTDNFVLQPGFIFESGPDTSIYKPYLRGQYNFDSGVYMAGRYRYDYARKTANYSDDEKTNRFDTYIGYLFDELKLEYntNYEHNVALAWKLNKSFTPYV..................
G9Z416.pir	VTVDVRQEYFDdnFSRLLLSHRFDSGIGASLESTIKsvTHSNEYSLSYQGDY.KGLLLEPGVSLEAGDSYSIYKTYVKATYEINDSWWVAGRYRFEYINNSERDNRDDTVNRADLWVGYNLENWSFTLetVSEGNFRTAYKIG.AWVPYVEVGNVprQTRYRVGVSYTF
V5F7L1.pir	TTIEYRHEYRDghGDRIKAFLDTGKNIGFELDARYNntMNGSEFSAFYYTKLNPNLVGIGGLSLDFTSDGLVYVPYARLNYRFDNGIRVQGRYKWKLWDYSMNNTAGEAYVSKIQQADFWLGYNTNDWdtNYLHNFRLMYTygTMWRPFIEVGNVsrQTRYRTGIKYTW
Q5DYR8.pir	TTVEYRHEYKDghADRVKMFLDTGKNIGFELDARYNNkmNGSELSIFYYDKIAENTTGLLGSSLDFNPDGLVYVPYVRVNHSFDNGFRAQARYKWKIWDYSQTNAAGEKYTSKIQELDTWLGYNTGKWdyDYLHNFRIMYSfgTKWRPFVEVGNVrrQTRYRVGIKYTW
518445055.pir	TTIEYRHEYRDghSDRVKAFIDTGNNLGFELDARYNntMNGSELSIFYYNNLSENIVGVFGGSLDYNPDGLVYVPYARLNYSFDNGFRVQGRYKWKLWDYSQEGTDGESYHSKIQQVDGWFGYKTGNWdtNYLNNIRFMYSINttWRPFIEIGDVsrQIRYRVGIKYTW
V2ITM4.pir	TTIEYRHDYRDghGDRFKVYLDTGENIGLEFDARYNNklNGSELSAFYYTNLNKNTVGLAGLSLDYNSDGLVYIPYVRLNYTFDNGFRLQGRYKWKLWDYGMVGADGNSYHSKIQEFDSFIGYKYRDWRDfdYQHNIRLMYSLDKNWRPFIEIGNIkrQTRYRVGIKYTW
656054969.pir	TTIEYRHDYRDghGDRVKVFLDTGQNIGFELDARYNNemNGSEFTAFYYDKINANTNWLAGTSLDFTKEGLVYIPFVRLNYQFDNGIRLQGRYKWKVWDYDVNGVDGTPYRSKIQQFDTFVGYRYHDWNfdYQHNVVVTYSLNKNWRPFVEVGNIkrQTRYRVGIKYTW
603151654.pir	........MPEXXGFYVDASVKSGGVDGEQDKHLSDLVANAIELGVSYNYKVTDNFVLQPGFIFESGPDTSIYKPYLRGQYNFDSGVYMAGRYRYDYARKTANYSDDEKTNRFDTYIGYLFDELKLEYntNYEHNVALAWKLNKSFTPYVEVGNVarQTRYRVGLQYHF
D0Z3T4.pir	TTIEYRHQYVDghSDRIKAFLDTGKGVGFELDARYNnsMNGSEFSAFYYKRLNPNTVGLVGMSLDFVPDGLVYVPYVRLNYKFDNNIRVQGRYKWKLWDYSQMTSGPSGDSYHSKIQEFDAWLGYNTQNWDfnYLHNFRLMYTydTSWRPFIEIGNVsrQTRYRMGIKYTW
595581682.pir	TTIEYRHEYRDghGDRIKAFLDTGKNIGFELDARYNntMNGSEFSAYYKKKLTDNIMGIGGLSLDFTSQGLVYVPYARLNYRFDNGIRVQGRYKWKFWDYNQTNLNNVNYISKIQEVDFWLGYNTNNWdtNYLHNFRLMYTygTMWRPFVEVGNVsrQTRYRMGIKYTW
V5FN80.pir	ASVDFRQEYKeSYASRIKIGSSVGnyFGLEAKQTGKpgSADNEFEYGYNFKLDDHWRITTSMPITFGDDRITYKPQVRVQYKFDSGLTTKLRYRHEFRDYADSTDKDSVNRSKITGNIDYNIGALQLGfnEWDYNFKVGYKedWSWRPYFELGNVqrQLRTRVGVTYSF
515589328.pir	ASLDVRQEYKHDsaSRIKIGGSTGnfYGLEAKQKGQpeSADNEFEYGYKFQVTDHWLIQPSMPITFSSDKVTYKPQVRVQYAFDSGVKAKLRYRHEFRDFTDESGESSEQKSKVTGNLEYNYENLQLGFenWDMNLKIGYKedWAWRPYVEFGNVsrQLRSRVGVTYSF
518194350.pir	GYINYRHQYLEdhSDRVLIGIRLDNGLGFEGELKYKtvGDGHEFTADYRYSLTPQWTIAPFISLVAVENATTYRTGARLGYKVNNQWTISGRYRYEARKLDRNQVDNSVPDRAKRDQHVHRFDGWLGYanDYEQALAFKYKLDKRWQPFFEVGNVkrQMRLRVGVHYSF
H7E932.pir	TSIEYRHDYRDghGDRFKVYLDTGKNIGFEFDARYNNklNGSELTAFYYTNLNKNTVGLAGLSLDYTSEGLVYIPYVRLNYTFDNGFRLQGRYKWKLWDYGMVGVDGNSYHSKIQEFDSFIGYKYDRWDfdYQHNVRIMYSLNTNWRPFVEIGNIkrQTRYRVGIKYTW
R8ATT2.pir	TTLEYRHQYVDghSDRIKAFLDTGKNIGFELDARYNNemNGSEFSAFYYNSLNKNTTGIAGLSLDFVPEGLIYVPYVRLNYTFDNNIRLQGRYKWKFWDYGMTGVDGQNYHSKIQQFDTWLGYSLQNWdtNYSHNIRLMYTygTAWKPFVEVGNVsrQTRYRVGIKYTW
636345584.pir	VTIDLRHEFIDgnADRVSVSHRFANGLGFTVEAKWRsvGNGHEDTISWRWKATSNLFLTPGFTIESNDSRSIYKPHLHAQYSFDNGFYVAARYRYEYTRYPNNAGKDDDKVNRGDAWAGFVLGDWRTELNYVYARSSEGVSRN.DNKPYSQEYNVK........VAYSF
U3BW23.pir	ATLDYRAEYKHEsaQRIKISGSSktKLNFGVEQKFHsvAGDSEFDWGVRYQINKQWFIQPGMPITFGDdkTTYKPQVRVGYKSSFGLSTALRYRHEFQVSYDGAGNKTLTDGTSVSVAGKTVEQGKWTLteYLDNVKLSYEIngDFRPYFELWNVkrQLRTRLGLKYSF
D1P6L2.pir	TTIEYRHQYLDAsgDRIKAFLDTGKNIGFELDARYGnkIDGSEFSAFYYTKLNSNLVGLVGSSLDFDKQGLVYVPYVRLNYTFDNGIRLQGRYKWKVWDYGQVGENGSNYHSKIQEFDSWIGYKWGKLDldYQHNIRLMYSLDigALWRPFVEVGNVkrQTRFRVGIQYTW
515587028.pir	ASLDFRQEYKHEsaSRIKIGGSTGnfFGLEAKQKGQpeSADNEFEYGYKFQITEHWLIQPSMPVTFSGDKITYKPQVRVQYAFDSGLKAKLRYRHEIRDYTDESGVSTEQKSKVTGNLDYNYENLQFGFenWDMNLKIGYKedWAWRPYVEFGNVsrQLRSRVGVTYSF
515595246.pir	GTLDYRTEYKHDdaHRAKIGSSTkaKLYFSIEQKWQSerGDSEFDWGVQYKINNNWYVQPGMPITFSSNKTTYKPQLRVGYKANFGLTTALRYRHEFQDYNSDGGTFTDTDGNKVDRADKTIQQGKWTLteWDLGVIVGYQID.NFRPYMEFWTVdrQLRTRIGLKYSF
515593644.pir	ASLDYRHEykeSNGDRIKIGGSTKVELGTHYYSVEMkeRGDSEFEYSFKYFVNDNFYLQPGMPITMGDQRVTYKPQLRVGYEFDSGITAKLRYRHEFRDFTDEKENGETWQKSKVTANLTYTWNSTVQFdnYDYNLKVGYKNDSNWRPYFELGNVsrQLRSRVGLTYSF
M4U7M7.pir	TTLEYRHEYKDghSDRIKIFLDSGENLGLELDARYNNemNGSELSIFYYKALNRNLLGLVGLSMDFNPDGLVYVPYVRLNYSFDSGFRTQARYKWKLWDYADTTTDIRSKIQELDTWVAYASGNWDFQmsDYLYNFRVMYAfgKKWRPFVEVGNVrrQTRYRVGIKYTW
658927258.pir	ASLDFREEYKHDsaGRIKISGSTGnyYGVEMKHTGNLsrGDSEFEYGYHFKLDDHWRITTSMPITFGDEAVTYKPQVRVQYKFDSGITTKLRYRHEFRNYASGKTSTGLDGQQHDSLNAGKITGNIDYnyEWDYNLKVGYKegWDWRPYVEFGNVqrQLRSRVGVTYSF
515595253.pir	ASLNVRHEYkeQHSTRIKMGDSIGnfKGADGDFMKDLKNNGWELDLGYRYKVDgnWTIQPGMPIEGRESGMTYKPQLRATYEFEsgLNVSARYRYDIKTYSTGESTDLRHRLTANVNYAYADWRFGFEgyELYNNTEEDYELngSWAPYVEFGDVsrELRSRVGVTYSF
Q9RGQ2.pir	ASVDYRQEYKhsYASRVKVghFFSLEAKQTGKPISDWQAADNEFVYGYNFKVNKKWRITPSMPITFGNDRVTYKPQVRVQYKFDSGLTSKLRYRHEFRNYTGDKSDKDSIDRSKITGNLDYKVGALQLgnEWDYNVKIGYKedWDWRPYIELGNVqrQLRTRVGLTYSF
U5A9U9.pir	ASLDFREEYKHDsaGRVKIGGSTGnyYGVEMKHKGNLsrGDNEFEYGYHFKFDNNWRVTTSMPITFGDEAVTYKPQVRVQYKFDSGVTTKLRYRHEFRNYASGKTSTGLDGKQHDSLNAGKITGNIDYnyEWDYNMKIGYKedWSWRPYVEFGNVqrQLRSRVGITYSF
668971670.pir	LTFDVRGGYRAGseSRFKVSESWKNGWWASMETDNKntSDYNEIETNYTYKLTDKWSLQPGGILHWSSKGTQLRPYFRINYKITPDLSTSLRYRYDYNDYETTNDNGEYHRDSVNRLDLYLRYKINDTWssTENAFSLRYKWNDWFSPYIEYDYLDreSRYRIGMTFTL
K8WCQ6.pir	TYIDYRHEWLEdhEDRVKIGHSMQNGTYFSLEGKWRssSKGNEVEVGYKYKATKDFALTPSFALDSGDKDTAYKFSVKAAYNLTDDFNIGTRLRYGVRHYDNSNKPKKDNQHYWQSNFYLGYKLQDFSLsdYAHNLNVSWIGNKTWVPYIELGYVswQWRYRVGIAYNF
D5CGY2.pir	ETREAYNTASELHEVILRAGYNFDMGAGLMFTNAYNVghSYNEIEGWYPLFktDKLTFQPGGLINDSSAGSGGAVYLDTNYKFTDGFNLTFRYRYNHNNYDTPDYNGQMDKNDTHEFANYWNFKVTDAFfhWEITNKFSYKIDKNWLPYLELQWLDrqYRIRLGLRYSF

```
R9PUI6.pir        TSVDFRHEYKSdhASRVKMAHTFENNFSMALELKFKGesNGSEIDLGYRYKINDEWALIPGMPIEFGQSGATYKPQLRLTYTPEkgLSLSGRYRLDVKPGEDIKKYRHRYTANIGYKYQQWTFGLEGNYynYENNFTIHYSMG.SWTPWLEFGDVsrELRSRIGLRYNF
652169853.pir     ATLDFREEYKhdWAGRIKIGGSAGnfFGVELKHKGNleSGDNEFEYGYKYTFTDHLLAKPGMVITFGDGNTTYKPQIRFEYAFDNGITTKIRYRHEFRNYAAGKTSTGRDGEQHDALNRTKITGNLTYqyEWDYNLKIGYKMSTKWRPYVEFGNIqrQLRSRVGVTYSF
515591232.pir     TSINIRHEWKPehADRIAVSHRFKSGIGFEVEAKYKtsGNGNQANISYRWKINDTFSLTPQFKLEANSDKVGYQGNLTLGYKATDTISTSIRYRYHYENYKSSLDKANSHYNRITLAAGYSGIEDWKFGliSEINSKISYtfGGGWAPFVEIGVQpwRPRYRVGIVYSY
A5L6R1.pir        ASFDVRHEYkdQHATRVKLGDSIGnfKGEDGKFMEDLKNNGWELGLNYRHVLNDNWTMTYGMPIEGRESGVTYKPQVRATYKVDsgLSLSARYRYDMRQNTSSTEYQVDDNGEIVAVDQNIENQRRHRlyDIYDNDDTNYELngQWAPYVEFGDVsrELRSRVGLTYSF
E8M6F3.pir        ASFDVRHEYkeQHATRVKMGDSIDnfKGEKGKFLEDLKNNGWELDLGYRYKfnNNWTLQPGMPIEGRESGMTYKPQLRLTYDLDggLSLSGRYRYDIRQnrRHRLTGNVNYRMDDWRFGFEANYYKADGYDIFDNSDTNYELngQWAPYVEFGDVnrELRSRVGLTYSF
D0ZBP0.pir        TSVEYRHEYREghSDRFKVFLDTGNHIGFEFDARYGNhlNGSELNVFYYTDLNKNTLLLAGNSFDFTSEGLVFIPFVRLNYTFDNGFYINGRYKWKLWDYSMKGVDQQPYHSKIQEFDTFIGYKWGQWNfdYANNFRVVYAIDANWRPFIEIGDIkrQGRYRIGLKYTW
652565157.pir     ASLDFRQEYKHEsaSRVKMGAGVGnfFGVEAMLKGKpeSNGNEFEYGYKFKLNDHWTLLPSMPLTFFSGHTSYKPQLRVQYKFDSGLVAKLRYRHQFRYFTDDRDMERLSYVTANLNYNVNYFQLGLEayNWDAGVWIGYKggWKWRPYVQFTNVsrQLRSRVGITYSF
G9YC49.pir        LTFDVRGGYKAGseSRFKVSQSWSNGWWASMETDNKntSDYNEIETNYTYPLTDKWSIQPGGILHWSSKGTQLRPYFRVNYKITPALSTSLRYRYDYNDYETENNNGESHRDSVNRLDLYIGYKINKQWsaTENAFTLRYKWNDWFSPYIEYDYLGkeSRYRIGMTFTM
A6CY66.pir        ASLDIRTEYkeQHATRIKMGDSIGnfKGEDGAFLKDLQNNGWELDLGYRFKINDNWTIQPGMPIEARSAGMTYKPQLRVTYALDsgLSLSGRYRYDIRQNNGEDAQNERRHRLTANINYSVNEWRFGLEGNYYKAATDGYIlfGQWAPYVEFGDVnrELRSRLGVTYSF
S6UJ37.pir        MPTALTSTIATNTRKAVKFGSRLDSGWGFEGELKYKtvGNGHELTASYQHRLNDKWMLTPSLALESIEKSTAYKMGLKLGYKVNDQLSVSGRYRYDSIKLDRDKVNHDMPDNQMDDQSINRYDVWVNYAsdYEQNAAFKYRWDKHWVPFFEVGDIkrQLRLRVGVQYNF
S7JNR7.pir        TSLNLRHEFQDghKDRLLLDHRFENGIGLSAEVKWAnhDAGHEVVVSYNYKVTDTFTLQPAYGADSNTNGITHKFNLKGTQKLSDDWDVALRYRYGDANKTEDSGKADSHYHQLNLTSGYKAGDFKFGlyKFEQSSSSGYKGDegWRPFAEFAMVsyAPRYRVGLKYSF
515595248.pir     GSLDYRAEYkeTYAQRIKIGESVntKLYFSVEQKFQstRGDSEFDWGVRYDINKQWYIQPGMPITFGNERTTYKPQLRVGYKASFGLTTALRYRHEFREYSDSDSNGNRTLTDGTSVASAGKTFQEGKWtnWDIGGIIGYQIG.DIRPYMEFWNVkrQLRTRIGVAYSF
515595251.pir     GSLDYRGEYKHEsaHRVKIGSSVkaKLYFSVEQKFNseRGDSEFDWGVTYTLNKNWYVQPGMPITFANGKTTYKPQLRIGYKADFGLTTALRYRHEFQSFVDGTSDATSSDGNSKITRDGKTLQQGKITleWDLGLKVGYKID.NFRPYVELWNIkrQLRTRVGITYSF
C4SNF7.pir        ETREAYNTASEQHEVILRGGYNFDNGSGLMLTNAYNAghSYNELEGWYPLFksDKFTISPGGLINSSSAGSGGSGYIDFNYKFLPIFNLTARYRYNHNNYESKDLNGDYAHNDTHEFATYWNFKLTDALshWEVTNKFSYKIDTHWMPYFELQWLDrqYRFRLGLRYTF
516228313.pir     ATLDYRAEHkdKYAHRIKIGGSTktKLYFSVEQKFApeRGDSEFDWGVRYALNDKWYLQPGMPITFGDEKTTFKPQFRVGYKADFGLTTALRYRHEFQNYTDSSSSSLTKVNGDKIALAGETVQQGKFTleWDLGVKIGYKFD.NLRPYVEFWTSdrQLKTRVGITYSF
656054568.pir     ETRTAYNTASDLNEVILRGGYNFDMGAGLMFTNAYNVghSYNEIEGWYPLFktDKLTIQPGGLINDNRDGSGGAAYLDINYKFTPTFNLTFRYRYNHSNYDTMDLNGDMNKNDTHELANYWNFKVTDALshWEITNKFSYKVDQNWMPYLELQWLDrqYRIRLGVRYSF
515639873.pir     ASFDMRHEYkdQHATRVKLGDSIGnfKGEDGKFMEDLKNNGWELGLNYRHVLNDNWTMTYGMPIEARESGVTYKPQVRATYKVDsgLSLSARYRYDMRQNTSSSEYQFDDNGDVIVDSDGVPITVDKNvyDIYDNDDTNYELngQWAPYVEFGDVsrELRSRVGITYSF
K8WNW7.pir        TYIDYRHEWLGdhEDRVKIGHSMQNGIYFSLEGKWRSksKGNEVEVGYKYKVTKDFVLTPSFALDSGEKDTAYKFSVKAAYNLTDDFNIGTRLRYGERHFDNSYKPKRDNQHYWQSNFYLGYKIKDVSIsdYAHNVNFSWVGNKTWVPYFELGYVswQMRYRVGISYNF
653845010.pir     ASINLRHEFIPehRDRMTLAHTFENGIGISGEMKWGyeSVGHEAKVSYTYKATDSFTLQPAYALDSNDSAVTHKLDLKGTQKLTDDWNVALRYRYGYKNVSAPSTDNSHYNQLNLTSGYNIGDFGLGIdyLNLVNLTAEYKGFegWRPFIEFGMVsyVPRYRAGLKYNF
V5HM68.pir        GQIDYRAEYkdEYQHRVKIGSSVkaKVYFSVEQKFNseRGDSEFDWGVRYDLTKNWYIQPGMPITFSNEKTTYKPQFRVGYKADFGLTTAIRYRHEFQTYTSDAEGSSTTVDGESLLRAGKTFQQGKITlgWDAGVIIGYQVN.NFQPFIELWNVkrQLRTRVGVKYKF
B7VN43.pir        ASFDMRHEYkdQHATRVKLGDSIGnfKGEDGEFMEDLKNNGWELGLNYRHVLNDNWTMTYGMPIEGRESGVTYKPQVRATYSVDsgLSLSARYRYDMRQNTKSIATDSDGKPILDNGYLTYQNDENQRryNIYDNDDTNYELngQWAPYVEFGDVsrELRSRVGLTYSF
G9Y8F9.pir        ETREAYNTASEESEIILRGGYNFDNGSGLMYTNAYNVghSYNELEGWYPLFhtENWTISPGGLINSSSAGSGGAAYLDVNYKFLSWFNLTTRYRYNHNNYDTIDTNGEYDKNDTHEMAMYWNFKVSDQFlhWEITNKFSYKIDDNWMPYLELQWLDrqYRIRLGLRYSF
A7MMS8.pir        ETREAYNTASELHEVILRAGYNFDMGAGLMATNAYNVghSYNEIEGWYPLFktENLTFQPGGLINDSIDGSGGALYLDTNYKFTPWFNLTVRYRYNHNNYDTPDFNGKMDKNDTHEFANYWNFKVTDKlhHWEITNGFRYKLDQHWLPYVELQWLDrqYRLRLGLRYSF
Q6LGJ4.pir        TSINLRHEYmdQHRDRITVSHRFDNGIGFSVEAKWRhkSGGHEVGVSYNYKVTDRFTLQPSYAADASSTGTTHKYNIRGITKITDNWSANLRLRYGDSRRTDGGTNRSYHQLNLVTDYKLSWGKVGVDlsDHLVNFFGEYnfESGWIPFMEVGILthALRYRFGVKYNF
U3B1F8.pir        GSLNYRGEYkeDFAHRIKIGESVntKVYFSVEQKFQseRGDSEFDWGIRYSLTKQLWLQPGMPITFGSGRTTYKPQFRVGYKADFGLTTAIRYRHEFREYTNDGQTISLAGGGSTDVSGKTTQKSKITLteWDLGLIVGYKMG.NFTPYAEFWTVdrQARTLGLKYDF
C4U763.pir        ETREAYNTASDQHEIILRGGYNFDNGSGLMYTNAYNAghSYNELEGWYPLFrtDKLTITPGGLINSNSTGSGGAAYIDLNYKFLPVFNLTSRYRYNHHNYESKDINGYYAHNDTHEFAMYWNFKLTDVLshWEITNKFSYKMDTHWMPYIELQWLDrqYRFRLGLRYTF
639223498.pir     ETREAYNTASDLHEVILRAGYNFDMGAGIMATNAYNVghSYNEIEAWYPLFknDRLTFQPGALINDNRSGSGGAVYLDTKYKFTDWFNLTLRYRYNRTNYDTPDFNGNMDKNDTHEFANYWSFKLTDQFthWEITNGFRYKVDQHWLPYLELQWLDrqYRIRLGLRYSF
515603440.pir     ASFDVRHEYkeQHATRVKLADNIGnfKGKDGEFLKDLQNNGWELGLNYRQKLNDNWTLTYGMPIEGRSSGMTYKPQLRATYSLEsgLSLSARYRYDIRQNNTSDNERRHRLTGNINYSVDNWRFGLEANynTEYNSTVRYIMG.QWAPYAEFGYAsfELRSRIGVTYSF
U3CNU2.pir        GQIDYRGEYKHDsqHRVKIGSSVkaKVYFSVEQKFNseRGDSEFDWGVQYSLNKHWYVQPGMPITFSDEKTTYKPQFRVGYKTSFGLTTAIRYRHEFQTYTSDASGSSTTVDGEKLVRAGKTFQQGKITlgWDAGVIIGYQVD.NFQPYIELWDVkrQLRTRLGLKYKF
515599502.pir     GTLDYRTEYKHDdaHRAKIGSSVksNDDGSVDFWNNTSRGDSEFDWGVRYALDGGWWLQPGMPITFASTKTTYKPQFRVGYKAGFGLTTAIRYRHEFQDYTSDGGTFTDTDGNKVDRADKTLQQGKWTLteWDLGVIVGYQID.NFRPYMEFWTVdrQLRTRIGLKYAF
639191517.pir     ETREAYNTASELNEIILRGGYNFDGGAGVMATNAYNIghSYNELEGWYPLFrtDKWTVSPGGLVNSSSAGSGGAVYLDNNYKFLPFFNLTVRYRYNHNNYDSIDFNGNPARNDTHEIANYWNFKLSDELnhWEITNKFSYKIDTHWMPYLELQWLDrqYRIRVGLRYTF
635729054.pir     ENREAYNLASDQQEIMLRVGYNFDMGAGIMLTNTYTlkHGYNEIEGWYPLFktDKLTIQPGGLITDKSIGSSGAVYLDVNYKFTPWFNLTVRNRYNHNNYSSVDLNGDRDNNDTYEIGNYWNFIITDKFshWEITNTFRYRINQNWLPYFELRWLDrqNQIRVGVKYFF
665969372.pir     ....MKDTSKSDHKDRLLISNRFANGFGLSLEGKWGqvSNGTEMVASYVYKFNNTFQIEPGFSLDSTSDSNNYRPYIRGKVAFTDDFSTSLRYRPYYKRNQPAQTKSTEKGHEFTMLFAYNFLKDYSAEyeWAHDVKIAYKWDKNWKPYMAIGN.............
654468489.pir     FTIDYRHELRSenFDRICLIGAFDNGVGFYVDSSFKsvANATEMSLWWGYKVAggFTITPGIITETVSNQTAYKPYVRLQYNTDFGLWFAVRPRYDYARNDNTKGDFHTYRIDAWVGYNHGNFGINYNYtnYEQNLAMNYRMG.QWNPYIEFGDMarQLRVRAGIQYTF
V3FCR4.pir        GFIDYRHEYLdtHADRVEFGTFFSNGIGLMGELRYNTqnNGSGLSIVYRFKpdKNFWLEPMFWLDSTEYWTTYEYGLSAGYNFSKEWRVSGRFRYDMDKATSKSKGYGNDDRNNRRYDLWVDYRPGNSNfnYTASFKVGYKFG.SWVPYVSIADYrrQGRYRMGLTYTF
V0PEX9.pir        ATLDYRHEYADrnKDRIAIIEKLPNGIGFYVDASVKsvANAIELGVSYNYKVTDNFVLQPGFIFESGPDTSIYKPYLRGQYNFDSGVYMAGRYRYDYARKTANYSDDEKTNRFDTYIGYVFDELKLEYNFTWMDSDQIKFDNKKQTMNIMWL...............
R9PUK1.pir        GQLDFRSEYkeAHAYRIKIGDSTknEDGEGYGGFLENLQRGDSEFDLSYKHKLNKKWYVQPGMPITFGDERVTYKPQFRVGYTSDFGLKTALRYRYEIRQYADGKENGETWYKSKITLTGGYKVKSMPNldNWDAGIFIGYQI.EKWRPYVEFWNSsrQLKTRVGVKYKF
E3GBY1.pir        VSLDYRHEYKSDsaDRLKISHTSESGYFASVEGKLAEggSGSEWEFGRNYKITDDLTLAPAVNLDIGDTYVGYRAQIKAIYKISDTWMTTFRWRPGIEVSEQSGTDNKNYNQFNWEFGYTGEQISVIGdyYWLYNIVASYKIDKNWTPYTEIGYVPreMRYRLGVKYNF
L4DRL0.pir        ENREAYNLASDQGEVMLRVGYNFDMGAGIMLTNTYNykHGYNEIEGWYPLFktDKLTIQPGGLINDKSIGSGGAVYLDVNYKFTPWFNLTVRNRYNHNNYSSTDLNGELDNNDTYEVGTYWNFKITEKFshWEITNTFRYRINEHWLPYFELRWLDrqNQIRIGTKYFF
653847214.pir     ASVDIRHEYkeSNNNRIKIGGSTSTDLGKHYYSVEMkeRGDSEVVYEFKHYITEQFYVQPGMPITMGDERVTYKPQLRIGYEFDSGITTKLRYRHEFRDYTSNKENGETWQKSKVTANLDYTWNNMIQFgnYDYNLKIGYKNETNWRPYVEFGNVsrQLRSRVGITYSF
D4BJ32.pir        MTFDMRGGYKEAseSRFKMSQSFQSGYWLSMETDNKQgtDYNEIEGNYKYKLNPALAIQPGMVYHWSSAGTQIRPYVKFIWNMTDSVYSGIRFRYDWNQYDSVDLSGNMDKGSVERVDLYFGWQNEKWnsSYENDLVIKYKLNKTWQPYVEWDYMetENRFRVGLTINF
612160075.pir     VTFDYRHETNDkhKDRLLMSHRFANGFGLSLEGKWKgvSNGTEVVASYVHKINKTFQIEPGFSLDTSSSSNNYRPYLRGKANLTDDVSASLRYRPYYKRNSGNIGTSKDTSENGYNLTSVLSWKFLK...DYQLDYELDYKKPTPRGLF..................
H3LFD2.pir        ENREAYNLASDQMEFMLRVGYNFDMGAGLMLTNTYTlkHGYNEIEGWYPLFrtDKLTIQPGGLITDKSVGSSGAVYLDVNYKFTHWFNLTVRNRYNHHNYSSTDLNGEQDNNDTYEVGTYWNFIITDKFshWEITNTFRYRINQNWLPYFELRWLDrqNQIRVGVKYLF
F9AWE9.pir        ATINIRHEFSPEhrDRIAVSHRFDNGIGFEVEAKWRsaGNGQQANVSYRYRLSDSFTLTPQYKWESNDSKMGNQFNLTLGYKINSDWSASFRHRYHYETKPNVDESSHYNRWTFGAGYTGFDLWSLGASliSEVNFTAEYrlESGWRPFGEFGMTpyRPRFRVGMKYNF
567432933.pir     TSINIRHEFVPehSDRIEISHRFSNGIGYGVEAKWKstGNGQQTNISYSYKLSDKLTLKPQYKWESGSDKVGHQFNLSLGYKVSDDWSVGFRHRYHYENKVGSDNSHYNRWTFSAGYKGIEDWAFGADLdvSDINFKGTYklNNGWSPFAELGFTpwRPRYRIGVKYSY
E3G3T3.pir        LTFDVRGGYKTGseSRYKISESWVNGWWASAEMDNKntTDYNEIETNYTWRFADKWNLQPGGIYHWSSKGTQLRPYIRVNYLPTADWSLGLRYRYDYNTYETLNDDGESHRDSVNRLDIFVGYKFNSEWsaTENAFTLRYKWNKWFSPYVEYDYLdsESRYRIGMTFTL
C9P7F8.pir        ATVNVRHEYKpsNVDRVAVSHRFKNGIGFEVEAKWKssGNGQQANISYAYKLSDSFTLTPQYKWESNDSKFGQQFNLTLGYRVNSDWNVSFRQRYHYETRPNNDKSSHYNRWTFGAGYRGLESWNLGTsyFSEVNFTFEYtlESGFRPFGEIGFNpwRPMFRVGVKYSF
N0U5P6.pir        AYVENREAYNLAseFMLRVGYNSDMGAGIMLTNTYTlkHGYNEIEGWYPLFktDKLTIQPGGLINDKSIGSGGAVYLDVNYKFTPWFNLTVRNRYNHNNYSSTDLNGELDNNDSYEIGNYWNFIITDKFshWEITNTFRYRINEHWLPYFELRWLDrqNQIRIGAKYFF
666389932.pir     ETRTAYNTASDLSEVILRGGYHFDGGQGLMATNAYNVghSYNELEGWYPIFrtDKWTITTGGLVNSNSAGSGGSVYLDNNYKFLPSFNLTVRYRYNRSNYDTIDFNGNPSRNDTHEIANYWNFKISEQFnhWEITNTFRYKIDNNWMPYLDLQWLDrqYRIRVGVRYTF
V2JXS3.pir        MTFDMRGGYKEAseSRFKMSQSFKSGYWLSMETDNKQgvSYNEIEGNYKYILNPKITIQPGMIYHWSADGTQIRPYVKFVWNMTDSIYSGVRFRYDWNQYDSTDLSGNQDKGSVERLDLYFGWQNQYWgsAYENDLVVKYKLNKTWQPYVEWDYMetENRFRVGLTINF
639210140.pir     ETREAWNTASDRHEVILRAGYNFDMGAGLMVTNAYDVghSYNEIEGWYPLFktRRLTFQPGGLVNDSRDGSGGALYLDTNYQFTPVFNLTFRYRYNHNNYDTPDVNGIMDKNDTHEFANYWNLKVTDSLthWEITNGFRYKLDRHWLPYLELQWLDrqYRVRLGLRYAF
660552996.pir     LTFDVRGGYRAGseSRYKVSQGWETGWWASMEMDNKntVDYNEIEANYSWPFADKWALQPGGIYHWSSKGTQLRPYLRVNYKATPDLTFGLRYRYDYNTYETVNSAGESHRDSVNRLDLYVAYKFNAKWsaTENALTLRYKWNDWFSPYIEYDYLgsESRYRIGMTFTL
V2D737.pir        .MTVRIFLIGFANGVGFAIEAISKSGGDDTNKAFNDLETQGNEYTISYQFK.TGDVAWQPGFVLETGNGYSTYKPYFRATWTLNESWWVGARYRFEYVRRSSDIRDDDTINRMDVWAGYKWNNFDWTIedNYEYNFRTAYIID.QWSPFVEVGNVsrQTRFRVGIGYTF
674432810.pir     ASINIRHEFQPDhaDRVAVSHRFANGVGFEVEAKWRsaGNGQQANISYLYRINESFTLTPQYKWESNDTKMGNQFNLTLGYKVNDDWSTSFRHRYHYETRPNVDQSAHYNRWTFGASYRGIENWGLGATvvSEVNFTFEYqfDSGWRPFGEIGATpyRPRFRVGMKYNF
A6VRQ2.pir        TTINIRHEYVPenKDRIEVSHRFANGIGFGVEAKWGssGNGQQTNISYRYKISDTLTLTPQYKWESGSDKVGHQFNLSLGYKVNDDWSVGFRHRYHYDKLADKAKQNSHYNRWTFSTGYKGIEDWGFGAsfSEVNFTGEYKGFkgWSPFAEVGMKP....YKSGDTYSY
T5JMD0.pir        ASVDIRHEYvdEHRDRIYVSHRFDNQIGFSVEAKWNygNAGHETGVSYRWKATDNFSLTPGINIDASPSgsVFKYNLTGAYKINDEWDVAARIRHGYKNTDDSRYNQLNLYANRKFEWGKLGVEAEYKdhDQLIDFKGEYtlESGVIPFFAIAAItyVPRFRVGLKYNF
674722155.pir     ETREAYNTASELNEVILRAGYNFDGGAGVMATNAYNIghSYNELEGWYPLFrtEQWTVSSGGLVNANSAGSGGALYLDNNYKFLPMFNLTVRYRYNRSNYDSIDFNGQPARNDTHEFASYWNFKVTDELnhWEITNKFSYKIDTHWMPYLELQWLDrqYRIRVGLRYTF
D4BEA1.pir        ENREAYNLASDQIEVMMRVGYNSDMGAGIMLTNTYTmkHGYNEIEGWYPLFktDKLTIQPGGLINDKSTGSGGAAYLDVNYKFTPWFNLTVRNRYNHNNYSSTDLNGNLDNNDTYEIGNYWNFTITDKFshWEITNTFKYRINEHWLPYLELRWLDrqNQIRVGAKYFF
M4JQ81.pir        GFIDYRHEYLDdhADRVEFGTFFSNGIGLMGELRYNtgNNGTGLSVVYRFKpdKKFWLEPMFWLDTTQYWSTYEYGLSAGYDFSKEWKVSGRFRYDMDKATDKSKGYGNDDRNNRRYDVWIDYRPQKTNfnYTASFKVGYKIG.SWIPYMSIADYkrQIRWRWGLTYTF
647266605.pir     LTFDVRGGYRAGseSRYKVSQGWQNGWWASMEMDNKntVDYNEIEVNYSWPFAEKWALQPGGIYHWSSKGTQLRPYFRINYKATPDLTFGLRYRYDYNTYETVNSVGESHQDSVNRLDLYVNYKFNAQWsaTENAVTLRYKWNEWFSPYIEYDYLdsESRYRIGMTFTL
640517675.pir     VTLDIRHEYkdQHGTRVKVSDSIDnyYGLEAKFAADkeRGDTEIDWGFKFNLGENWYIQPGMPIAFGDKKNTIKPQLRIGYRASspLTTALRYRREYSSYSEDSSDDYEQNKITFTMSYKDGKNKYWLekDIYNNTRENYDYigSWFPYMEFADVsrQLKSRVGIKYYY
514987290.pir     ................................PFNETVSNGTEVVASYVYKINNTFSIEPGFSLDSNSTSNNYRPYLRGKAVIVDDLSVSLRYRPYYKRTSANINTPKDTSENGYNLTSVISYKIAKDyyDWSHDLKLTYKWDKNWSPYMALGNVsrQTRYRVGVKYSF
E3G651.pir        WSLNYRHEFLEKssDRLVVGHRFDNGWGFSVESKFTNetTGTEYDLSYLAKPNSTWWWEAGFNTEITDDVQDLRVHFKPTYWFTPELYTAARFRVGQRNYTDASGQDNGHYFQLNYYLGYNAENWSIAyySYLNNVTIRVPINKNWRPYTEIGYVswQWRWRVGVEYVF
668720618.pir     VTLDIRGGYREGseSRFKMSQAWSNGFWLSMETDNKQgtDYNEIEGNYKYKLTDTVAMQPGMVYHWGGDGAQIRPYVKFVWNMTPATWSGIRFRYDWNQYDSTDLSGNQDKASVGRVDLYFGWQNDKWysAWENDLVIKYKLSKVWQPYIEWDYMgtENRYRVGISINI
650007777.pir     LTLNLRGGYRSAseTLIQVSQGWNNGWWASFESNSWNtlNYNKIDVNRTYKLDDKWALIPGIQTQWMSAGSQYNPYLKLSYQVSPDLNLALRYRYDWKAFRQTDLDGNRARNNQHQIDGYLTYRINDDWlaTENAFVLQYKLSPTFTPYVEYDYLDkeNSYRVGLLVNF
F9T3P7.pir        TKLDVRFGYNTTsdSRVKFMHTFDNGFYFSAEAAQLhqAAAQEFEASYKFNLNDDWYWSPGLVTVTTSDWTEYRPYLKLGTTFDNGVSMTGRYRYNWSNEANGKQYLDGSGTTRGASNQFDLWISKNIGdyWEHTIMVNYKVDETWTPYMELVSVDETYVDSNGNREND
607000613.pir     ATLDIRGGYRSGseTRLKVSEGWQNGWWASLESNSWNtlNDVQAEINYAIKLDDKWSVRPGMLTHFSSNGTRYGPYIKLSWDVTKDLNLGIRYRYDWKTYRQEDLSGKMDRDNAHRWDGYLTYKINKDFtaTENAFVLQYHMTPDITPYIEYDYLDRQGVYNGKDNLSE
A8AGB9.pir        VQFDVRGGYNIEneSRFKVSNSWSSGWWASMETDSNNgsSYNEMETNYTWHINDRFALVPGGVIHWSNSGTQLRPYIRLNYKISERFSSGIRYRLDHHNYDVVDNTGDTIHDNEHRIDLFLGYKINQDWnaTENAFTIKYRWNEYFSPYFEYDYLdpDSRIRVGVTFNL
654672276.pir     LTLNLRGGYRSAseTLIQVSQGWNNGWWASFESNTWNtlNYNKIDVNKTYKIDDKWALIPGIQTQWMSAGSQYNPYLKISYQVTPDLNLALRYRYDWKAFRQTDLDGNRARNDQHQFDGYVTYKINDSwtATENAFVLQYKMSPVFTPYIEYDYLdhENSYRVGVLINF
585354392.pir     AYVENREAYNL..ASDLGAGIMLTNTYTFQREDELK..HGYNEIEGWYPLFktDKLTIQPGGLINDKSIGSGGAVYLDVNYKFTPWFNLTVRNRYNHNNYSSTDLNGELDNNDTYEVGTYWNFKITEKFshWEITNTFRYRINEHWLPYFELRWLDrqNQIRIGTKYFF
635567373.pir     DNRYAHTTASEKNQYKIGLGHIFDNGAGVLASAMYDLgsSFQEFEGWYPIPLNDKWTLTPGGLTDIDSKGTKLAPYISLDYKISKSLSLSSRYRYNHMTHKDRDYNGYMDYNDSHQFDLYLNYQATEKltHWEPSIVARYRVDKHWLPYAEVAWLddQVRFRLGIRYYF
603141504.pir     ATLDYRHEYADrnKDRIAIIEKLPNGIGFYVDASVKsvANAIELGVSYNYKVTDNFVLQPGFIFESGPDTSIYKPYLRGQYNFDSGVYMAGRYRYDYARKTANYSXXGLLAEVGQRRIVIHSVFAQHAAVAVRGVFAEAGVGHDDHLRHGLLTDAghQSVFFPGIAAGV
655006846.pir     ASIDFRGQYreRWESRFLLGHELDNGLGGTIEYTINnsWKDTEFQLYYKYKLNDTITLLPSVLWDSVKSseVYKVGLQANWAFQPGWRLDGRVRYEYNNEPSANLNRQIDNDDWTRTDITLRNTINKEFdnYEHNIGGGYKITKNVRLHGEFGYVgdDWRFRLGATYTF
K1IVK4.pir        ESREAFNFATDQHELLLRLGYNWDSGAGIMASNAYNTehSYNEIEGWYPLLntTKLSVNAGGLLNSSSSGSGGSAYLDGKYNVTDNFSVIARYRYNHQDYLTTDLDGYGDNNDTNEYALYIVNKITPALthWELTSQTRYRINPQWMPYLELQWYDraYRIRIGVKYFF
```

```
677870033.pir   ATLDYRHEYADrnKDRIAIIEKLPNGIGFYVDASVKsvANAIELGVSYNYKVTDNFVLQPGFIFESGPDTSIYKPYLRGQYNFDSGVYMAGRYRYDYARKTANYSDDEKTNRFDTYIGYVFDELKLEYNFTWMDS.................................
F6CWF0.pir      TSINMRHEIVPehSDRIEVSHRFSNGIGFGVEAKWKssGNGQQSNISYRIKLSDDLTFTPQYKWESGSSKLSHQFNFSFGYKVSSDWSVGFRHRYNYQAKVDDDNSHYNRWTFSAGYKGVEDWSLSSSIdfSDVNFKGEYtlNNSWKPFLEFGLKpwRPRYRLGLKYSY
551321590.pir   TSIDFRHEWKAEdaSRVKMGHNYggNIGIEMKFRSYDalTETELDWGYTYKVNRNWELKPGMPIAITDRKTTFKPQLRIVYRADMGLTTALRYRYEIANYADSADGDTSMETGEKINQPHRTKLTLTGAydYDIGLKAGYRMG.NWQPFTELWDVkrQMKLRAGVKYYF
L2W9Y8.pir      ATLDVRGGYRSGseTRLKVSEGWQNGWWASMESNTWNtlNDVQVEVNYAIKLDDQWTVRPGMLTHFSSNGTRYGPYVKLSWDATKDLNFGIRYRYDWKAYRQQDLSGDMSRDNVHRWDGYVTYHINSDFtaTENAFVLQYHITPDITPYIEYDYLDRQGVYNGRDNLSE
655000093.pir   ASVDVRGQYrdKFESRVLLNHEWSNGIGAGFEYVVDNtwKENELELYYKYKVSDTLTLLPSVLFQDTksGDIAKIGLRANWAFAPTWRLDARVRYEHKTRDTRDLAKQWDNDDTTRSELWLRKTFNKDlnLYEHNVGAAYKLNANFKPYAEIGYLpdDWRFRLGLVYSF
655481417.pir   AYLDLRHEYRdrHRTRLILGNHFDNGYGIEMLTNVFHgiSNTEITNYYTYAVNKNFTLNPGLTMNFLSSNTFMMPYLKLNYNFDNGLFVHGRYRYDFSNAPMQNDFGEDETVRRNRYDVWTGYNTDDYqtGREHTFKLVYKWKPDVRPYLQLVDAdtEWRYQIGLNLAF
NanC.pir        ATLDVRGGYRSGseTRLKVSEGWQNGWWASMESNTWNtlNDVQVEVNYAIKLDDQWTVRPGMLTHFSSNGTRYGPYVKLSWDATKDLNFGIRYRYDWKAYRQQDLSGDMSRDNVHRWDGYVTYHINSDFtaTENAFVLQYHMTPDITPYIEYDYLDRQGVYNGRDNLSE
L0M8V3.pir      DNRLAHTTASEKNQYKIGLGHIFDNGAGVLASAMYDLgsSFQEFEGWYPLPLNDRWTLTPGGLTDIDSSGTKLAPYISLDYKISKTLSFSSRYRYNHMTHKDFDYNGRMDYNDSHQIDLYLNYQATDKlkHWEPSIVARYRMNKNWLPYAELAWLdnQVRVRLGVRYYF
515678629.pir   GYVDLRAEYrdQYRSRFIVGHHFDNGYGLETLTNVRhkVINTEFTHYYTYAINDNFMLNPGVVMNFQGSNTFLMPYLKLNYNFDNGLFVHGRYRYDFSTEALVNDYGNEETAKRNRYDIWTGYNTDKYmnAREHTVKAVYKWKPTVRPYAEVVDAdtDWRFRVGVNFSF
Q1Z3H2.pir      TKLDVRFGNndIKDSRVKFMHTFDSGFYFSAEAAQIhkAAAQEFEATYKFQINDDWFWSPGLVMVTAPNWTEYRPYLKLGTVFDNGLSVTSRYRYNWSNDANGKNKLDGSGTTRGASNQFDLWLSKSFGdyWEHTVMVNYKLNETWTPYMELVSVDETYVDENGKREND
515585635.pir   TKLDVRFGNndIKDSRVKFMHTFDTGFYFSAEAAQIhkAAAQEFEATYKFNINEDWYWSPGLVMVTAPNWTEYRPYLKLGTNFDNGISVTGRYRYNWSNDENGKNYLDGSGTTRGASNQFDLWVSKSFGdyWEHTVMINYKIDDTWTPYMELVSVDETYVDSNGNREND
A6AML5.pir      TKLDVRFGHndIRDSRVKFMHTFDTGFYFSAEAAQIhkAAAQEFEATYKFDLGEDWYWAPGLVMVTAPNWTEYRPYLKLGTTFDNGVSLTGRYRYNWSNDANGKNYLDGSGTTRGASNQFDLWISKNFGdyWEHTIMVNYKLNETWTPYMELVSVDETYVDEQGNREND
603142470.pir   RYIRELKAYPYGCLEQTTSGLFPALYTNAAQLQSLGITGDSDEKRRAAVDIgsRILQMQRDNGGXXGPDTSIYKPYLRGQYNFDSGVYMAGRYRYDYARKTANYSDDEKTNRFDTYIGYLFDELKLEYntNYEHNVALAWKLNKSFTPYVEVGNVarQTRYRVGLQYHF
655487076.pir   AQIDFRHEWRSHdaSRVKLGTGFnaNIGIEMKFASFdeLTETELDLGLTYKMG.KWQIKPGMPIALTDRKTTFKPQIRVVYKSDFGLTTALRYRHEFANYSDPTDGDTNVETGLKVNDPTKSKVTLTGSyeYDAGIVVGYQFG.NWRPFAEIWTIdrQAKYRAGIKYKF
571190120.pir   ................MRVGYNSDMGAGIMLTNTYTmkHGYNEIEGWYPLFktDKLTIQPGGLINDKSIGSGGAVYLDVNYKFTPWFNLTVRNRYNHNNYSSTDLNGDLDNNDTYEIGNYWNFAITDKFshWEITNTFKYRINEHWLPYLELRWLDrqNQIRVGAKYFF
R9NJI7.pir      VSMDYRHEYRttHYDKLTLSTQLPANYFFAAETKFKtvLNAVEMTLGKKIIIG.NWTVSPIIQPEFNSTRTEWKFGVSPWYKINNRWSVGGLYRLELTDYAHDLSGCANCSTNKHRTVNRVDGYLRFRvyNYEQELQFNYALgkEWSPYISFGDInrQLRLRAGLAYTF
G5P738.pir      ATLDYRHEYADrnKDRIAIIEKLPNGIGFYVDASVKsvANAIELGVSYNYKVTDHFVLQPGFIFESGPDTSIYKPYLRVQYNFDSGIYMAGRYRYDYARKTANYNDDEKTNRF.....................DTYIGYVFDELKLEYNFTWMDSDQIKFTTGWIAIK
H9YCG4.pir      ...............MLRVGYNFDMGAGIMLTNTYTfkHGYNEIEGWYPLFktDKLTIQPGGLINDKSIGSGGAVYLDVNYKFVPWFNLTVRNRYNHNNYSSTDLSGELDNNDTYEIGTYWNFKITDKFshWEITNTFRYRINEHWLPYFELRWLDrqNQIRIGTKY..
578244172.pir   TSVDLRFGHndVNDSRIKVMHQADNGFYFSVEAAQNhtEAAQEIETRWRFDLGNGFAIAPGMVTVFTASNTHYRPFIQGWKTFDNGLNLSARYRYNTVNDAHSDKRLDGSGYTRRESHQFDLWFAYNIgtYWEHTVAFNYKLDDGWTPYVELVSLDKTYLTQDGNHEND
673534431.pir   TSVDLRFGHneANDSRIKVMHQADNGFYFSVEAAQNhtEAAQEIETRWRFDLGDGYSVAPGMVTVFTSSNTHYRPFIQGWKSFDNGLNLSARYRYNTVNDAHSDRELDGSGYTRRESHQFDIWFAYNIgtYWEHTLAFNYKLDDGWTPYVELVSLDKTYINDDGHHEND
498113550.pir   LELNYEHKYEdeHTDEIEISHDFESGIGIGSKLKSHPvlSEKEFKINYNKDITDRFSIEPGMSFTIKDDEEKYKPSIKFKYRLFDKTKLSLRYRKEISDRDEKPTKRVDRIEGKISQKIGKFDLGYTLtyNTEHEVEVGYQLTKHFSPYIGVKNEarQTEYIVGMNYKF
515599487.pir   ..................FYFSGELKFKGADGDFMKDLKNNGWELDLGYRYKVDgnWTIQPGMPIESRESGMTYKPQLRATYALEsgLSLSARYRYDIKTYSNGDSTENRHRITGNVNYSVADWKFGFEayNIYDNKEENYELngDWYPYVEFGDVsrELRSRVGLTYSF
671631157.pir   ATVDYRHEYRltHYDKLGLSTTLPDNMFLGVETKFKtvLNVVEVTFLKSYYWG.NWTLSPFIQPEFNSSRTEWKFGVAPWYKFNDRWSLGGLYRLELTDYAHDDQCRTGGIDYCDTDRHRTANRFDLYldDYEQELQFDYAldRVWVPYVTFGDIgrQLRLRTGILYIY
654547603.pir   TSVDLRFGHndVNDSRIKVMHQADNGFYFSVEAAQNhtEAAQEIETRWRFNLGNGFAVAPGMVTVFTPSNTHYRPFIQGWKAFDNGLNLSARYRYNTINDAHSDKELDGSGYTRRQSHQFDIWFAYNIgtYWEHTVAFNYQLDDGWTPYVELVSLDKTYINNDGNHEND
668994791.pir   LSLDYRHEYRvtHYDKLNMSAALPDNYNFSIETKYKtvINAVELTLSKSFTFG.NFKIAPLIQPEFNSERTEWKFGFVPWYTINENWAIGGMYRFELTDYANDPDCGYNGDKSCSTNKHRTVNRGDLYlyDYEQEWQFDYSLgkEWKPYVTFGDIgrQFRLRMGVVYTF
594396893.pir   VTVDYRHGYRltHYDKLALGASLPDNWAFNVETKFKtvLNVVEMTLLKTYNWG.NWSWSPFIQPEFSSSRTEWKFGVAPWYKINDSWSIGGLYRLELSDYAHDSQCGDAGHDFCSLDKHRTVNRFDAYfdNYEQELQFNYAMGaeWGPYITFADIgrQLRLRLGVTYTF
515594352.pir   ............SIGNFYFSGELKFKGADGDFMKDLQNNGWELDLGYRYKIDgnWTIQPGMPIEGRSSGMTYKPQLRATYALEsgLSLSARYRYDIRTHSNGDSTQFRHRITGNVNYSVADWKFGFEAnnYELNLTAGRKFG.DWYPYVEFGDVnrELRSRVGLTYSF
A6CXP1.pir      NYVDVRGAYKttYETRVRAGFDFaqAQGDKLFSNSGKSVAAFETELNYNWAINDNFTLTPGFVYWNGSDHMEYRPYLKATYAEGN.FYTAARYRYQAASSSTTPGVKTNDTNQVDLWVGYNLNDFALEytKWEHTFQVKYTGFESWAPYVDYQILdtENRIRAGVTFNF
```

The top layer shows the consensus sequence. Residues are coloured after their physico-chemical properties. (GPX black; positive blue; negative red; CM yellow; H dark purple; NQ light purple; all others green).

Class 4A

CONSENSUS	[illegible]
E2QLS8.pir	[illegible]
EcOmpG.pir	[illegible]
D4B911.pir	[illegible]
666376500.pir	[illegible]
V1H3R5.pir	[illegible]
F8VJH4.pir	[illegible]
D2TKA6.pir	[illegible]
L9HT82.pir	[illegible]
A9MQ52.pir	[illegible]
647319304.pir	[illegible]
578276270.pir	[illegible]
A4WAV1.pir	[illegible]
668724511.pir	[illegible]
N2Z2R6.pir	[illegible]
668964394.pir	[illegible]
446481394.pir	[illegible]
G9Y5U3.pir	[illegible]
639209387.pir	[illegible]
H5V239.pir	[illegible]
671535537.pir	[illegible]
658548019.pir	[illegible]
491210144.pir	[illegible]
607463328.pir	[illegible]
H2ITF3.pir	[illegible]
449467465.pir	[illegible]
485713379.pir	[illegible]
657150665.pir	[illegible]
D1AL54.pir	[illegible]
D4E462.pir	[illegible]
485713378.pir	[illegible]
446657896.pir	[illegible]
635780718.pir	[illegible]
518442615.pir	[illegible]
490385782.pir	[illegible]
684202871.pir	[illegible]
559166129.pir	[illegible]
491045365.pir	[illegible]
573500784.pir	[illegible]
559165615.pir	[illegible]
658301715.pir	[illegible]
577070577.pir	[illegible]
493869722.pir	[illegible]
674722870.pir	[illegible]
640470393.pir	[illegible]
522210633.pir	[illegible]
485787536.pir	[illegible]
502623387.pir	[illegible]
559166130.pir	[illegible]
658549097.pir	[illegible]
485707625.pir	[illegible]

The top layer shows the consensus sequence. Residues are coloured after their physico-chemical properties. (GPX black; positive blue; negative red; CM yellow; H dark purple; NQ light purple; all others green).

Class 5A

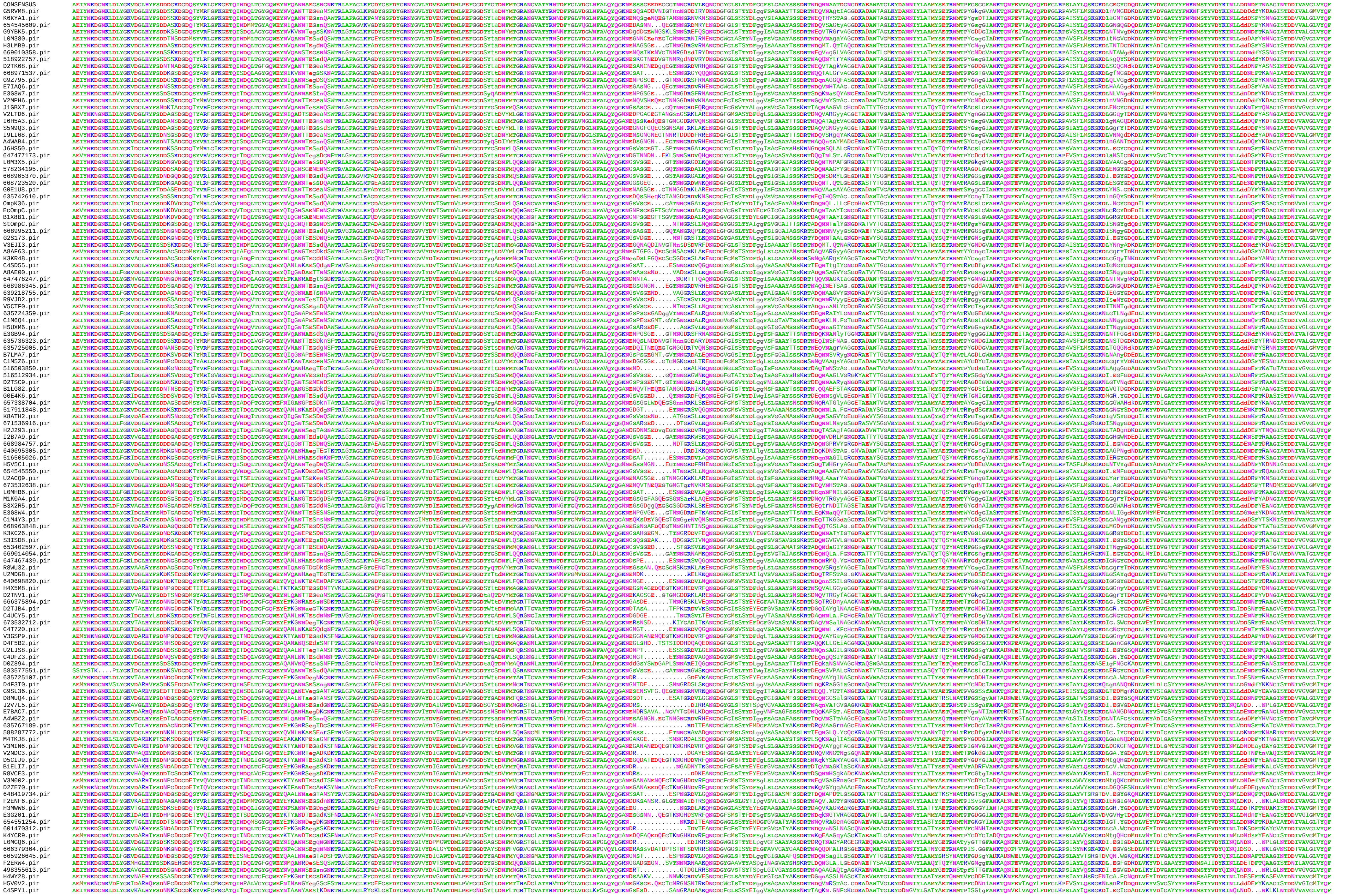

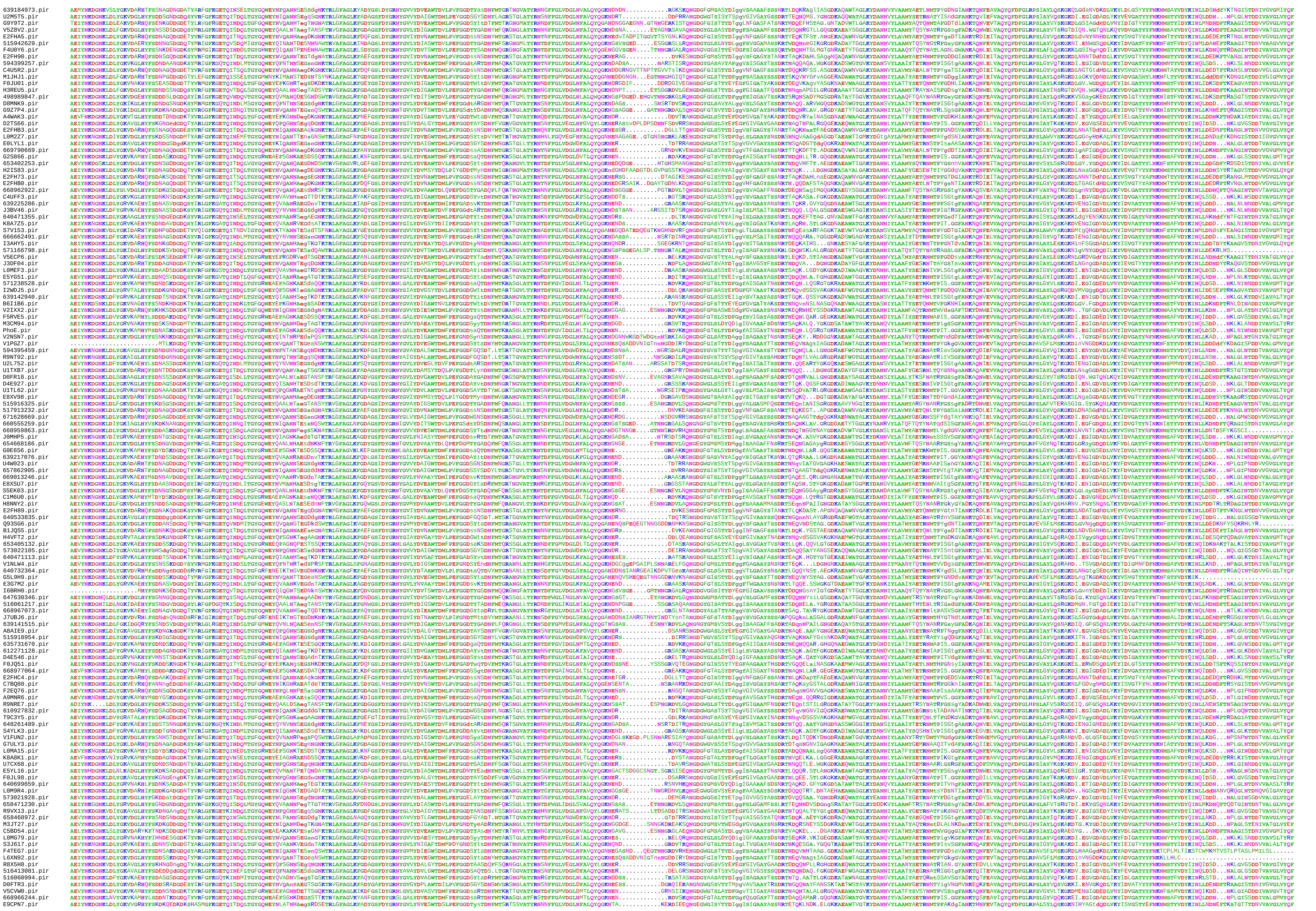

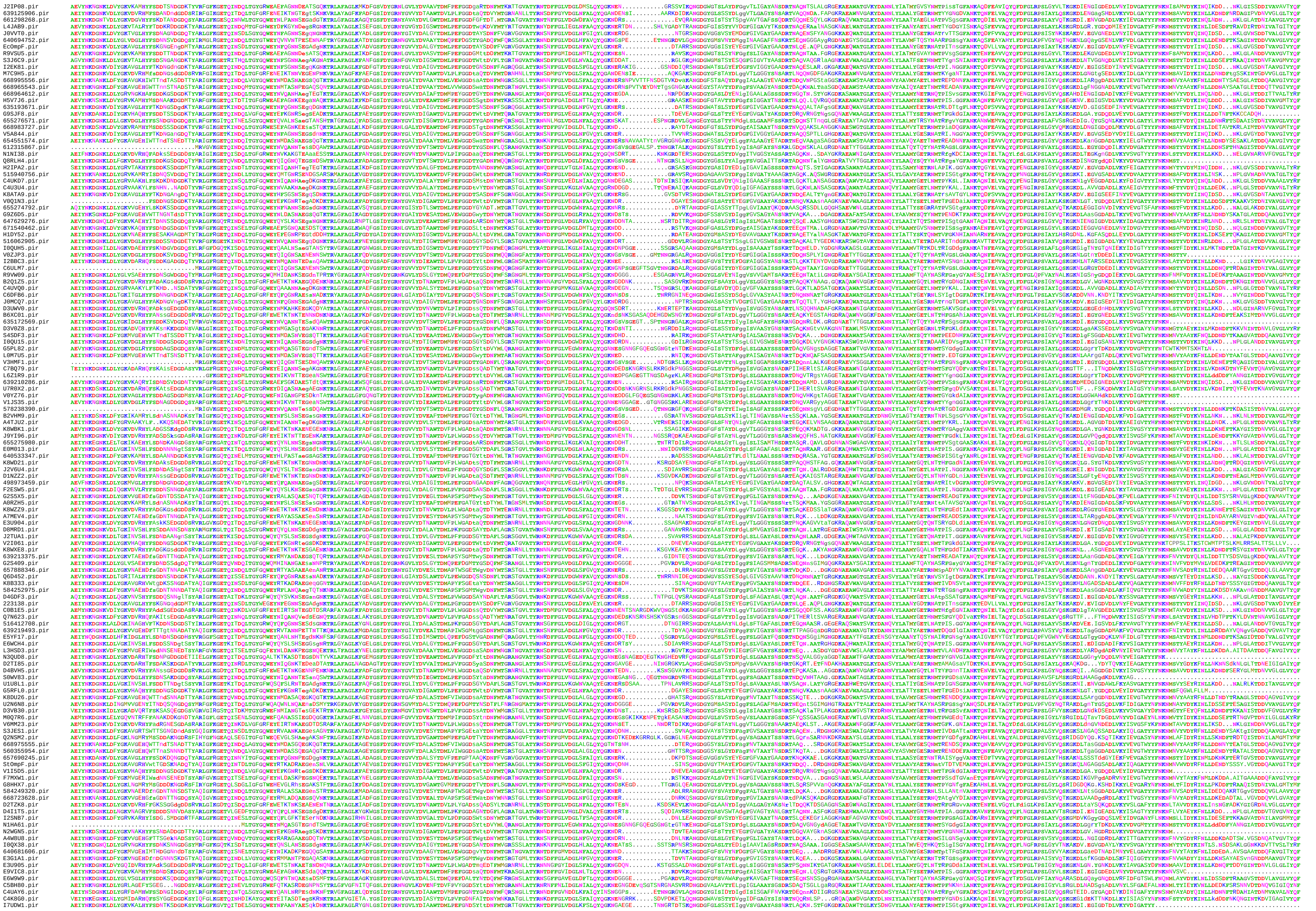

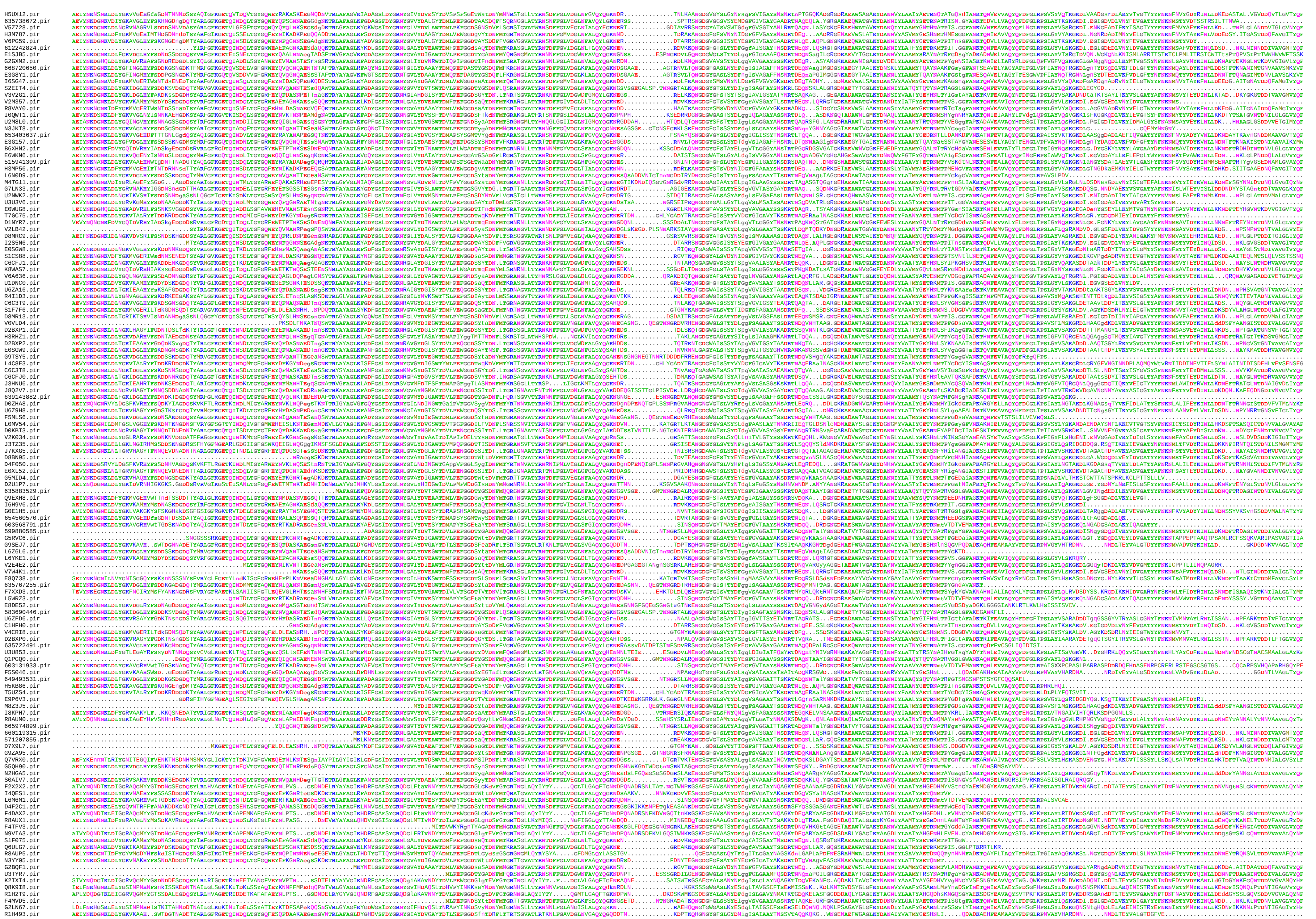

[illegible]

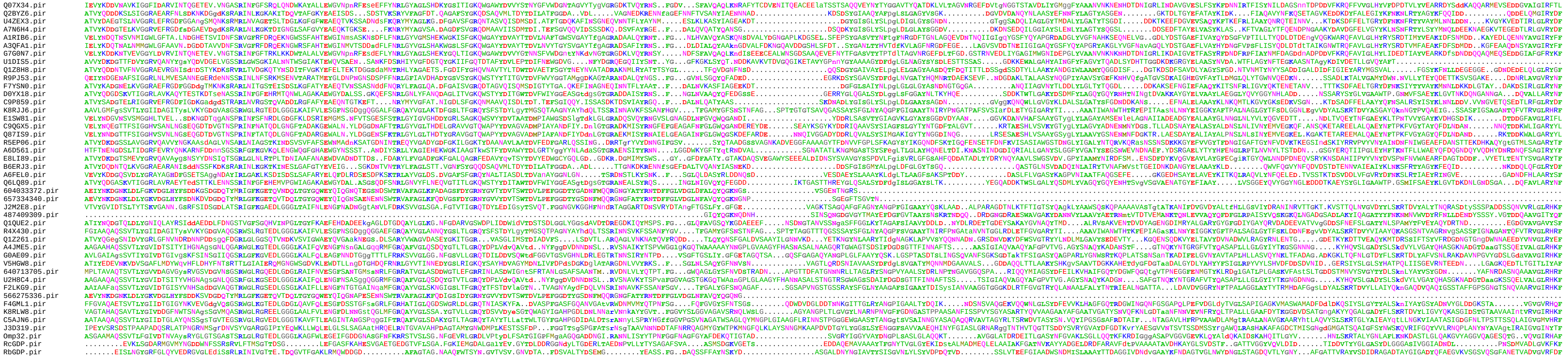

The top layer shows the consensus sequence. Residues are coloured after their physico-chemical properties. (GPX black; positive blue; negative red; CM yellow; H dark purple; NQ light purple; all others green).

Class 5B

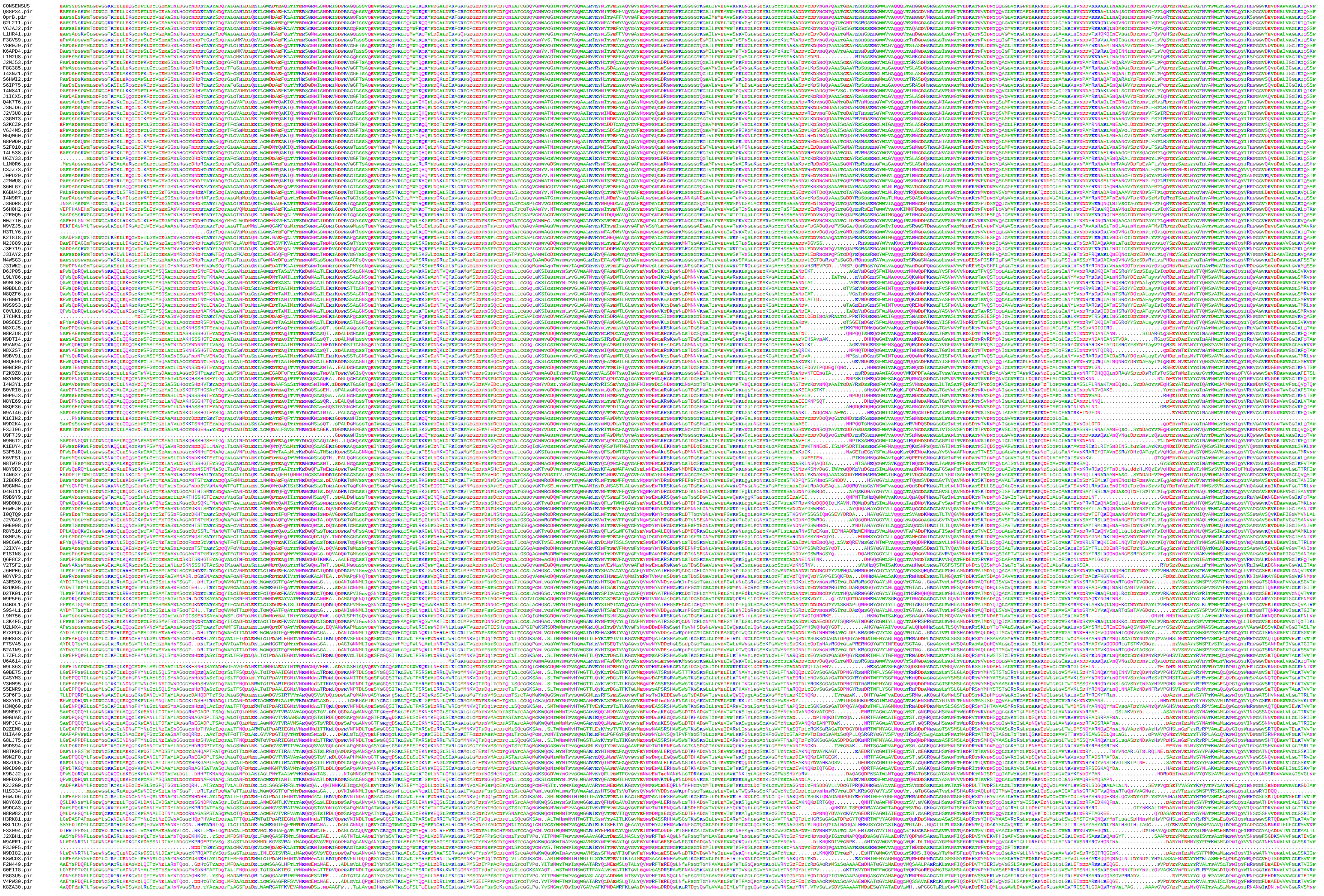

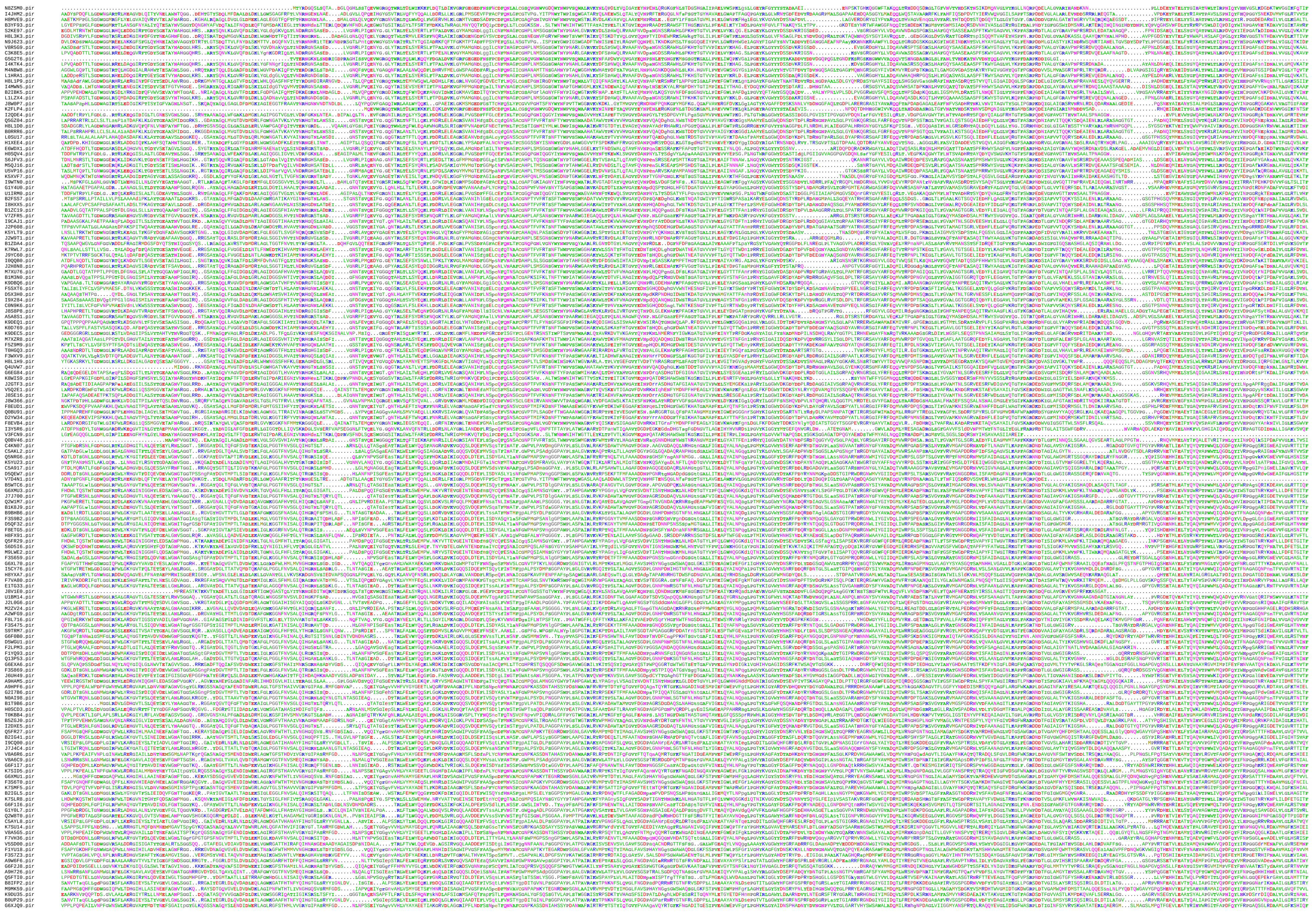

B1ZEI5.pir EGSIQEALGPYGDPFGLRAALAARGITYVLTYIGEGFASTSGGLRRGTT..FGGRLDAALDIDLDKLAGWSGARFHTDVFQIHGHGLSRRFVN.......NLTTVSGIepSTRLFELWIEQRFFDDRFSVKIGQVSADTEfaQSAVVFLNAGFGWPNIGAVvpSGGPIYPLATPALRLKYEPTAELSFQGGLFNGDPAGtqRRNRTGTNFRVNDPALLIGEVAYAYHSlrLPGTATLGGWHHFGRFESPRLDAVGGRLLADPEASGVarGNTGLYAMIDQTVYREPGRDEAGASAFLRISAVPSNRNLIDLYVDAGIAYRGLFAGRPNDTLGLGLIHSRISPAARAFDRDA.......LLFGTGPAPLRSSETVFEATYQAEVVPGLTVQPDLQYVMRPGGGLADdnATVLGLRATVNY

F3S7N0.pir YASSEAPGHILPQIGALRSRLQNRGVTFAFTYKGEAMADVGGGI..SRGMDYVHELTLQTQFDLGRLFGFTGWTVHTLLMERVGREVSHDRVGD....YNIsLMEVylSgsVAHLTDMYAQKSFLHNTVDLAFGRMALTHVFATSpcSFMMT..CSAPVAIKLDAGFSVYPKATWGGRVRLRPTRDTLVQIGAYSVSPLN.EDISGWAWAGEKSTGLMIPVEFAWQPFFgkLPGHYVLGFAHDTTRYADN....IGDVPAGSAIRPHGGEARDTFYLEADQMLYRKGGASqaGGYVLAGYIHNTPDVSVISDEFYVGSSLLGIIPHRPHDRFGVMYSYYRMSPRTELGQQLRMDAGMALGVHVNG...PQTHAAVLEAYYGVPVYPGILVQPEFQYMMRPGETAHIPNAEVVGLKVIGTL

The top layer shows the consensus sequence. Residues are coloured after their physico-chemical properties. (GPX black; positive blue; negative red; CM yellow; H dark purple; NQ light purple; all others green).

Class 5C

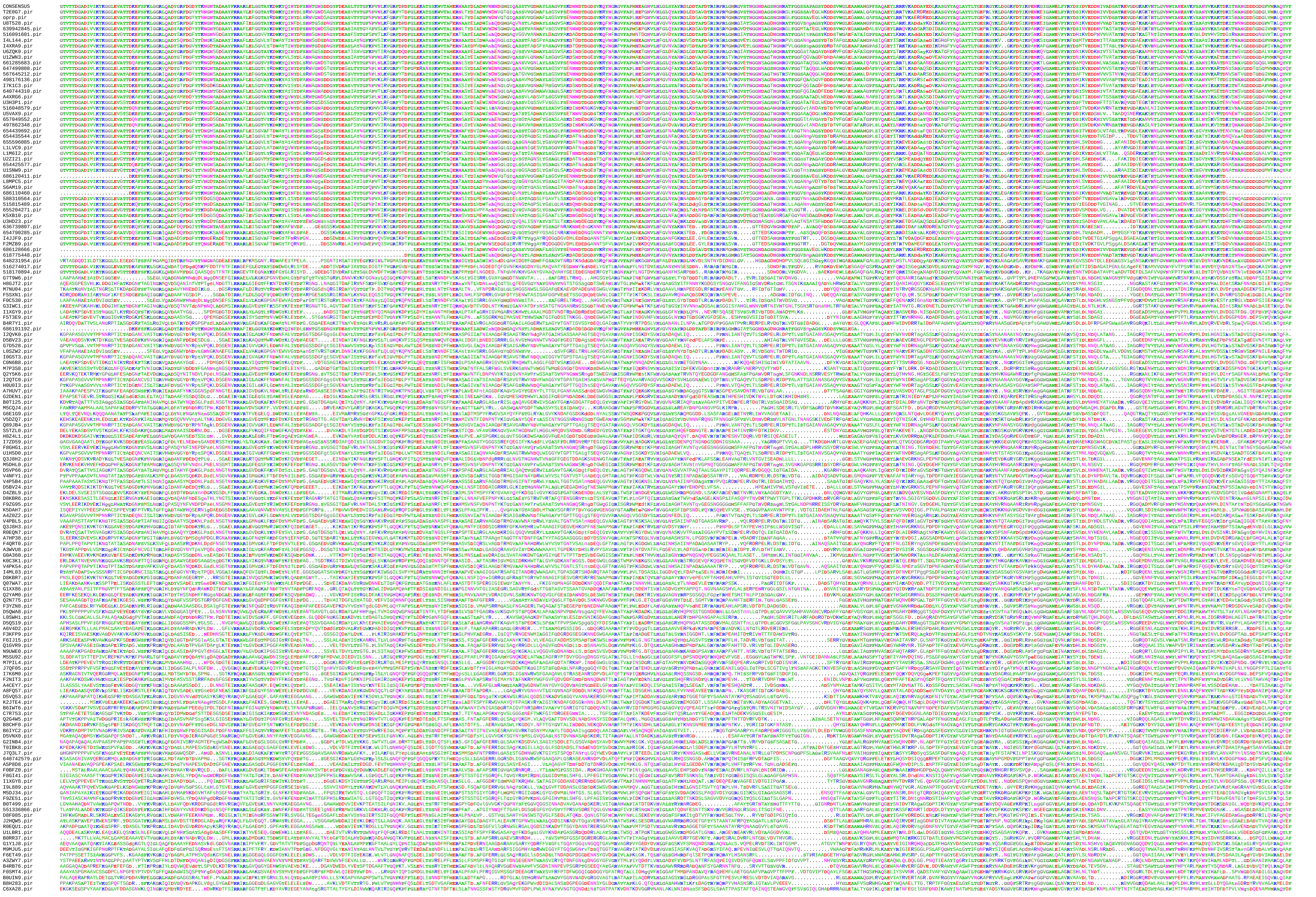

```
K7A270.pir    KDSPDDESPVSIKWKGAPEIKGESG.WSVKPKGRVLYDFANLSSVPsdIPGEGFSSEARRLRFGIQGTMPGGFGYKLIADYL......DGVVLTDAYMSYQDG.PLKIIIGQQNTFQGLEELSSSNDTSFVERSAFTDAF...GFERRVGLSATYNLGDFMLEGGIFSDNIDDLNDGNNSFSVDGRVTYATLV.DDALIHLGSSVHIRDLGDEIDSIRYRQRPLVHSVDTRFINTDQILGAEDELS..............YGLEAAVIAGRFHMAAETHWAKINRVE.LADPTFFGGSIEAGYFLTQDQREYK..GGVFKGVkpgNGGTGAWQINLRFDRLDLVDAGIV.........GGEQDAYMASLIWTPVNNVRFLLDYGHLAYSnvEGAPKDFSVNVLGARTQLSF
M4RZ36.pir    PPPAKAAPETGISWKGSPQFTSDDR..AFKVKGRIQADAGYVSHPPGSDdgFGFTSEMRRIRLGGEGKLGSGFGYKLELELSDNK.....VDLVDTFVTHET.KDWLVAIGNQNQFQSLDELTGDTTGSVMERAAFTD.AFGFERRLGISAQYHHKDwtDDIGALSNSSDGPSGGDENNSFGVDGRVVFAPKV.DDTQLHFGASGHWRTLKRLakPTVYRQRPFLHGTNSYVLETPGLNV...............DDEVNYGLEAAAIRGPWHAVAEAHWLRAMRPDA.PDVGFFGGYAEVGYFlkGDTRSYR..NGIFGGADPKrgGIGAVQLNLRYDYLSLNDRSAG.......VLGGRQNAYLAGLVWTPINYLRFNLNYGYLTYSPGVAVDgdYNLHVIGTRIELDF
U2YHJ4.pir    DDMKKVQAKNAPSWKGAPQFADADEGWSFKVRGRMMLDTAYQSTPDNLNsdFGFNSRIRRIRIGVEGSLPGNFGYKAEVDYA....NSSVGFGDVVLSWNPNKGPLTLTAGNQESLDGMEQMTSSRWSSFIERAQVND.AFGNTRRLGMTVGYKSKDNLFRIDGGLFVAHSIDSSLDNRGWIGAARATYTPFVG.SGFVHLGLNYQHREFQANNGSTALTAGTGSPSAGQLVRYRARPFLQTTSQRFVDTGNFAAKGDDIFGAEAYGVFGPLHFGGEAQYAKVRSYRAGdnPSFWGGHFEIGYFLTGETRGYKNgwDRTKVLKPMGKgsGAFQILGRVDYLNMNSSKLQrsATSGFGKGGSQTGYLLGVTWIPNDYVRFLLNYIHTEVEGGPFAgrSYSTDAVALRAQFDF
G2I4Y5.pir    EEALHMGGMVVGFPGGRPTISSEDGMYAMSIGLAFHEDIGGFMGMSPRRGEtgLTENARRLRIPISFRY.KNWIANITPDfsGSADGTSTSQLYEANLNYAGFNHTVITAGYFQPRVTEEDSESSNEFELMERPAITDIVRNIAAGDARMSLGALHYAKRWWIAGYVTGQTYGKrtYDSQSGATFRVAGRPYMSKDIDVHIGLSAITAFKVAQAKASDDRSYNF..........SEAPEVNLTTTKLLSATVGNVGSIWSAGPELGFRWKRLTLKGEYYNIGVTRGDNgaTVNFTGYYGAANYTIFGKGRSYNIKEGAFAApdPSHGYWGALEMTARYSVADLNANLGNASTALR...GGQQTVWSAGLNWYPNRHFRFMLDFNHFIVSRNSVAAngREGNSVAGRIQAAF
```

The top layer shows the consensus sequence. Residues are coloured after their physico-chemical properties. (GPX black; positive blue; negative red; CM yellow; H dark purple; NQ light purple; all others green).

Class 6B

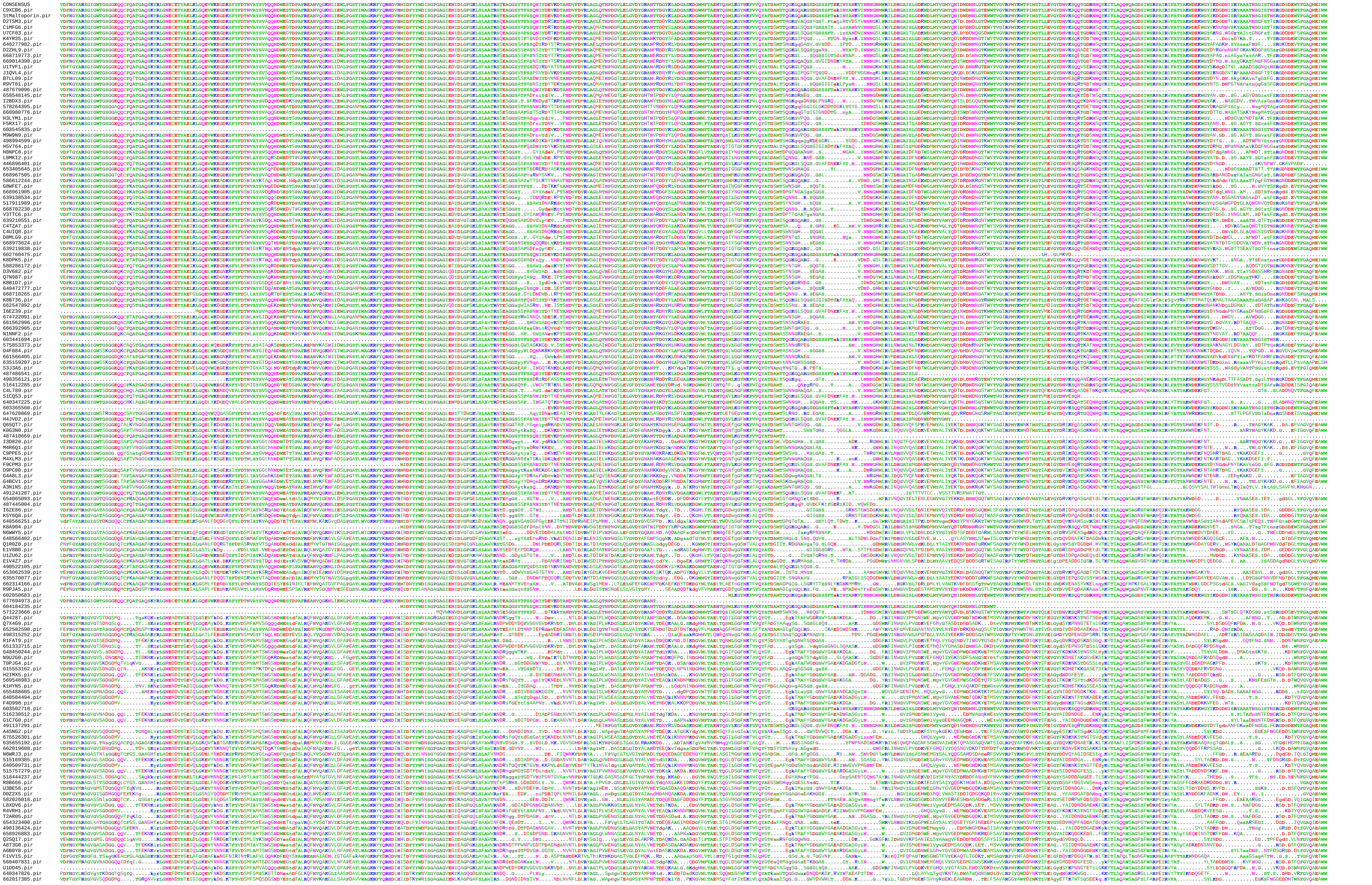

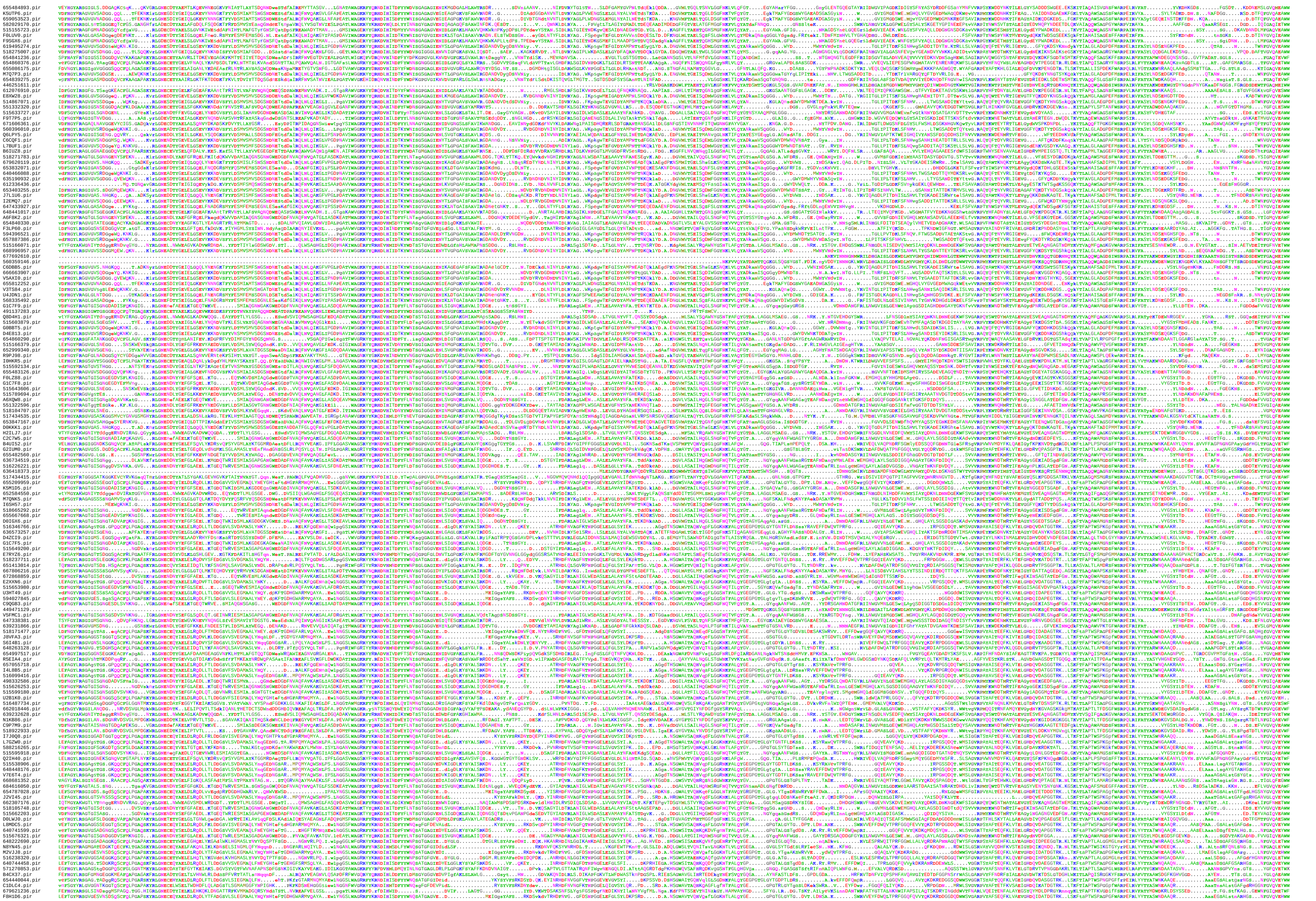

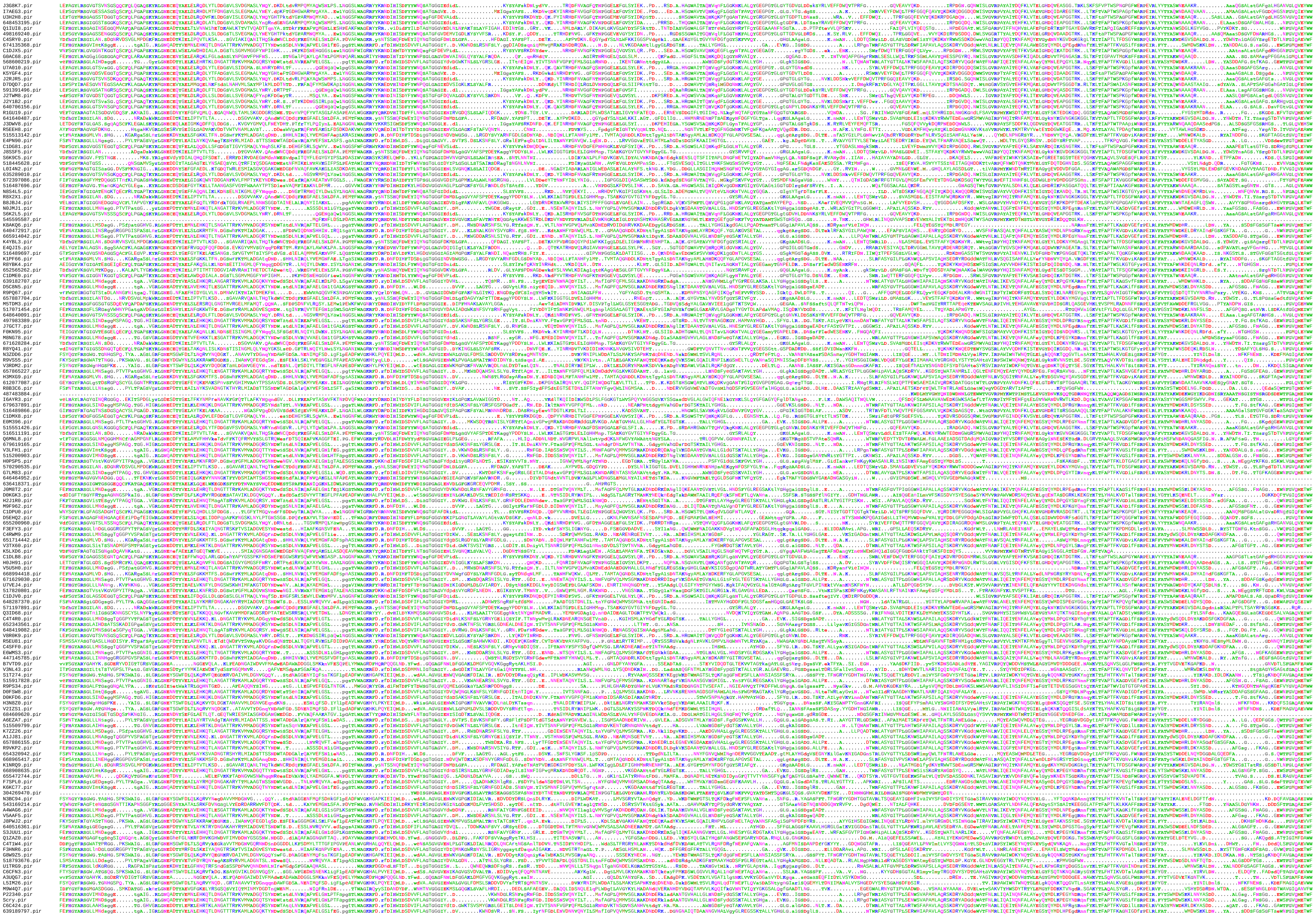

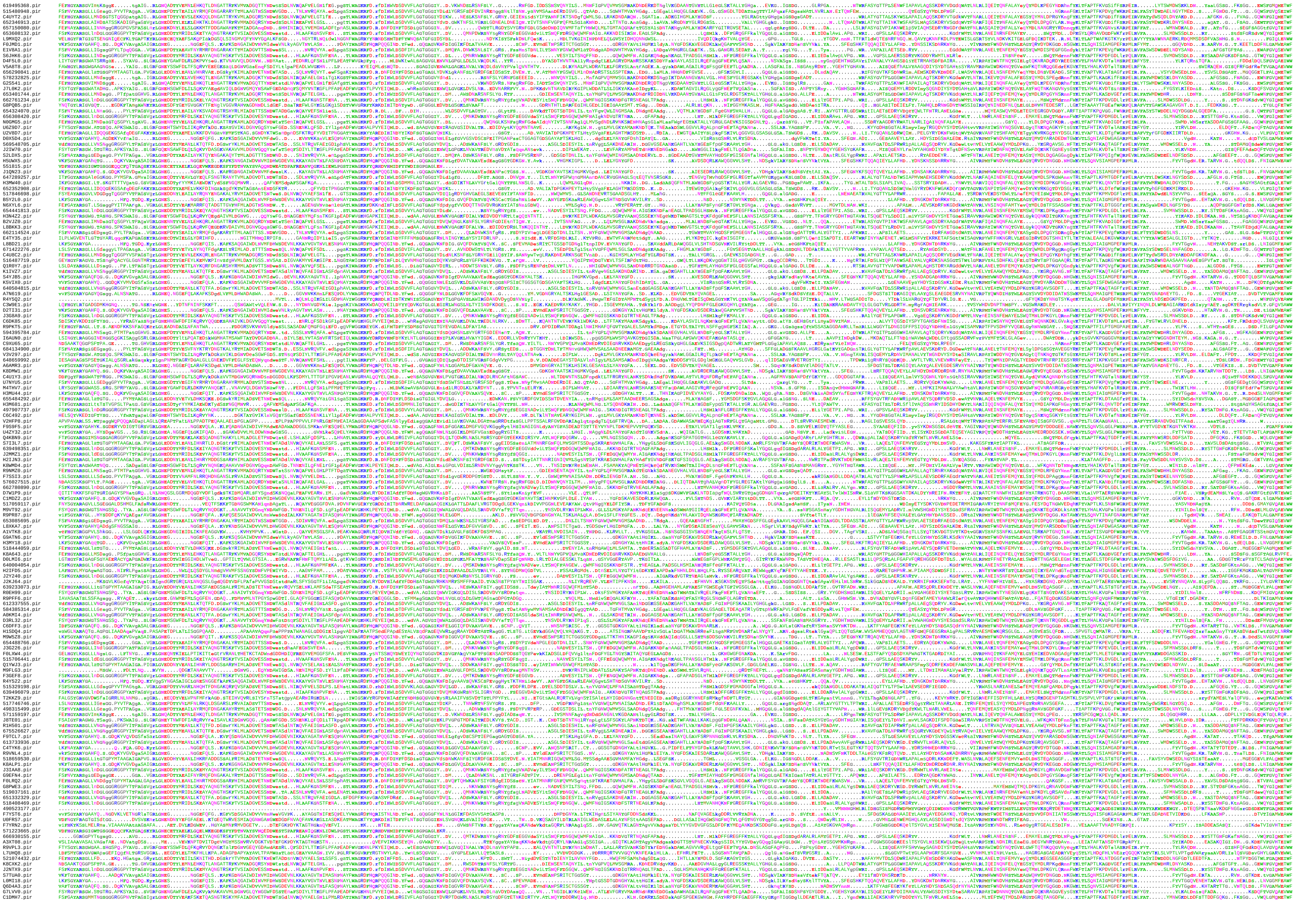

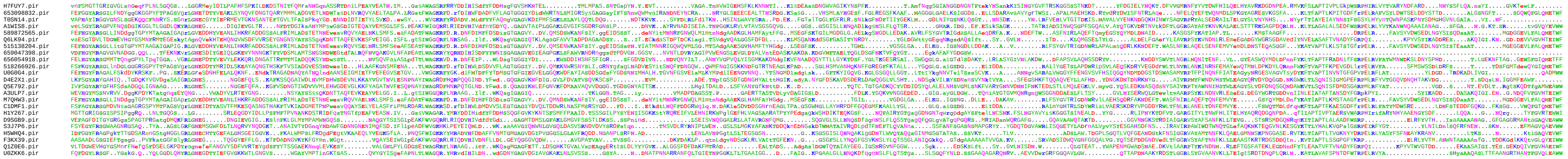

The top layer shows the consensus sequence. Residues are coloured after their physico-chemical properties. (GPX black; positive blue; negative red; CM yellow; H dark purple; NQ light purple; all others green).

Class 6C

```
CONSENSUS        [illegible]
S6AT13.pir       [illegible]
L1HN04.pir       [illegible]
640477144.pir    [illegible]
516564060.pir    [illegible]
S6AQ05.pir       [illegible]
631779814.pir    [illegible]
L8MGX1.pir       [illegible]
639934660.pir    [illegible]
655207681.pir    [illegible]
L1HQ40.pir       [illegible]
L8MRA0.pir       [illegible]
639920567.pir    [illegible]
U3H9W0.pir       [illegible]
654425599.pir    [illegible]
L8MTB7.pir       [illegible]
655211110.pir    [illegible]
J2Q5W5.pir       [illegible]
L1HRH3.pir       [illegible]
L1LUJ7.pir       [illegible]
516091200.pir    [illegible]
U2ZK25.pir       [illegible]
U7DJF8.pir       [illegible]
639932637.pir    [illegible]
E2ZP59.pir       [illegible]
S6JEI0.pir       [illegible]
M5QHJ6.pir       [illegible]
H7ERS9.pir       [illegible]
C1DK87.pir       [illegible]
636741289.pir    [illegible]
M4WZ96.pir       [illegible]
K5YGK5.pir       [illegible]
B0KV45.pir       [illegible]
U1SM90.pir       [illegible]
V6J792.pir       [illegible]
I4JL52.pir       [illegible]
U3HEX8.pir       [illegible]
498248264.pir    [illegible]
657733376.pir    [illegible]
515814434.pir    [illegible]
S6AKR6.pir       [illegible]
517758414.pir    [illegible]
639495635.pir    [illegible]
654434790.pir    [illegible]
653581127.pir    [illegible]
653604765.pir    [illegible]
J2ZGP8.pir       [illegible]
662761583.pir    [illegible]
588309556.pir    [illegible]
U8KCZ1.pir       [illegible]
I4JJ14.pir       [illegible]
665416570.pir    [illegible]
L8M963.pir       [illegible]
S6BA64.pir       [illegible]
U2ZHS2.pir       [illegible]
M4X4V2.pir       [illegible]
516090219.pir    [illegible]
L1LYN9.pir       [illegible]
A4Y0I7.pir       [illegible]
U8JNY9.pir       [illegible]
I4CUK5.pir       [illegible]
567644329.pir    [illegible]
L0GJY9.pir       [illegible]
M9S6V0.pir       [illegible]
OccK8.pir        [illegible]
591393183.pir    [illegible]

L8MJT9.pir       [illegible]
497906748.pir    [illegible]
L1HP58.pir       [illegible]
662776834.pir    [illegible]
573001765.pir    [illegible]
662765850.pir    [illegible]
I6ZEK9.pir       [illegible]
J2RJT3.pir       [illegible]
I4JL36.pir       [illegible]
A4VPE4.pir       [illegible]
J2S6U8.pir       [illegible]
640745300.pir    [illegible]
U3H4S8.pir       [illegible]
J3AV42.pir       [illegible]
Q4KGR5.pir       [illegible]
657910696.pir    [illegible]
F2KHF1.pir       [illegible]
655596325.pir    [illegible]
S2KLR4.pir       [illegible]
J2XB39.pir       [illegible]
F2N0L6.pir       [illegible]
L1M005.pir       [illegible]
A4VRR3.pir       [illegible]
U3H8K6.pir       [illegible]
U1SSP4.pir       [illegible]
J3DF27.pir       [illegible]
653595516.pir    [illegible]
655598317.pir    [illegible]
635288084.pir    [illegible]
640707533.pir    [illegible]
J2QUZ8.pir       [illegible]
U2AZZ3.pir       [illegible]
612111141.pir    [illegible]
S6AHL9.pir       [illegible]
653610744.pir    [illegible]
515154200.pir    [illegible]
U3BEL2.pir       [illegible]
655280800.pir    [illegible]
518240576.pir    [illegible]
516349017.pir    [illegible]
F2KDX7.pir       [illegible]
L8MBX6.pir       [illegible]
L1HW83.pir       [illegible]
518409999.pir    [illegible]
518172506.pir    [illegible]
655206393.pir    [illegible]
640741491.pir    [illegible]
631780672.pir    [illegible]
S6BBC9.pir       [illegible]
498116157.pir    [illegible]
J2MWZ1.pir       [illegible]
Q1I5N7.pir       [illegible]
E2Y0H8.pir       [illegible]
A4XSA7.pir       [illegible]
654507944.pir    [illegible]
U2ZJH6.pir       [illegible]
E2XN69.pir       [illegible]
664810247.pir    [illegible]
S6AEZ0.pir       [illegible]
654785283.pir    [illegible]
498171365.pir    [illegible]
636739993.pir    [illegible]
I7A5C6.pir       [illegible]
C3V9Z2.pir       [illegible]
631782432.pir    [illegible]
U1ZLF0.pir       [illegible]
M2USX0.pir       [illegible]
L1LYZ2.pir       [illegible]
K6BMV9.pir       [illegible]
```

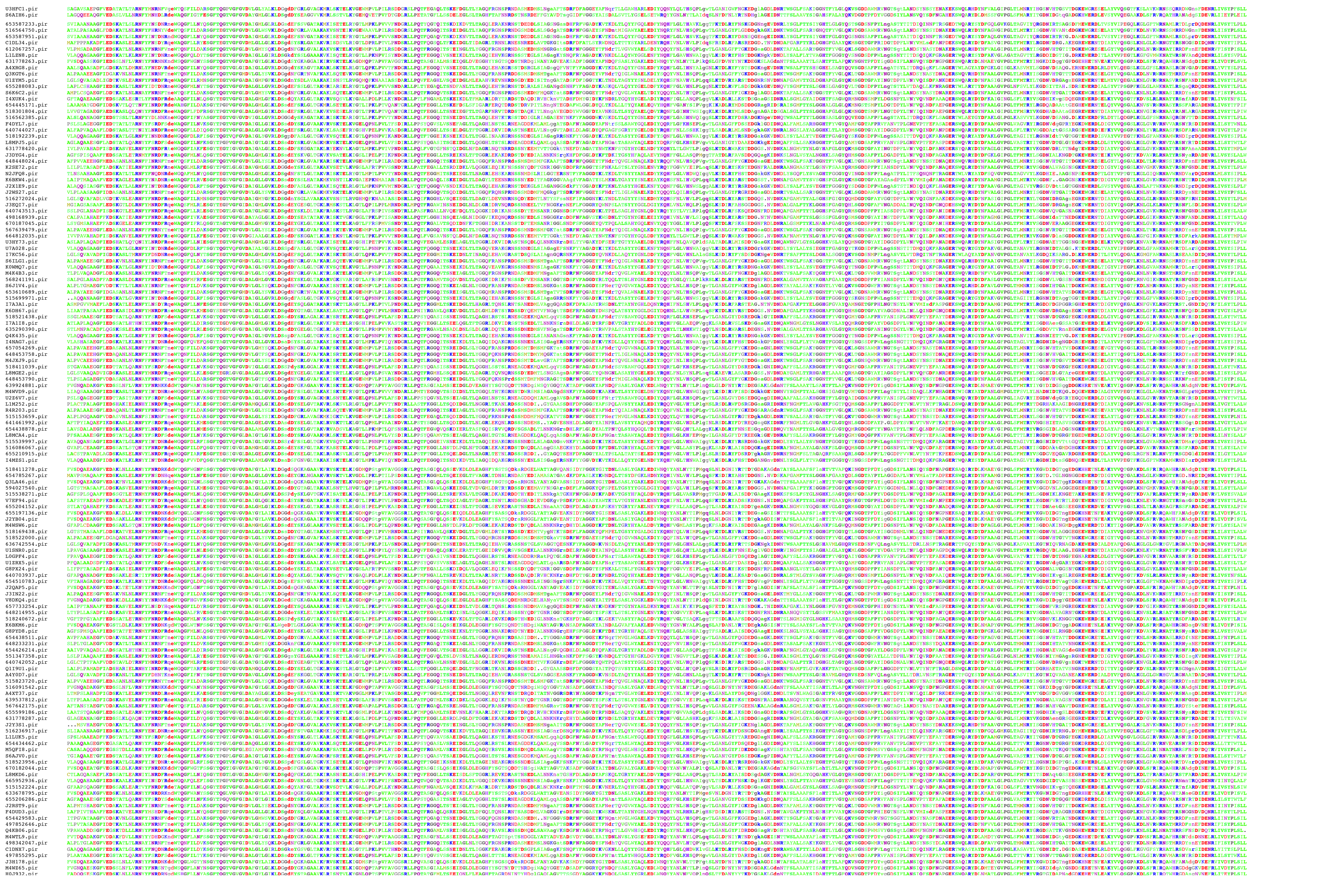

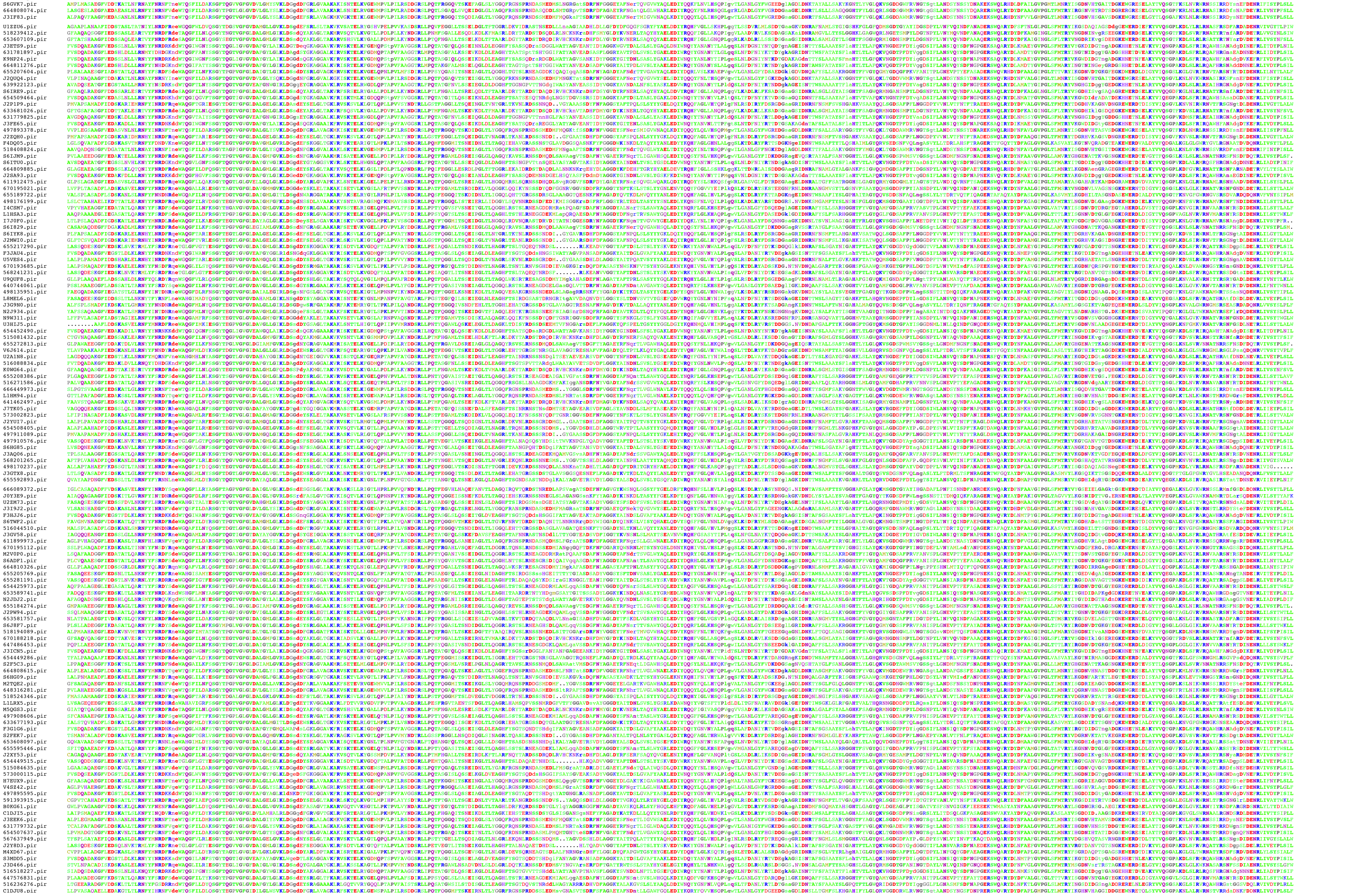

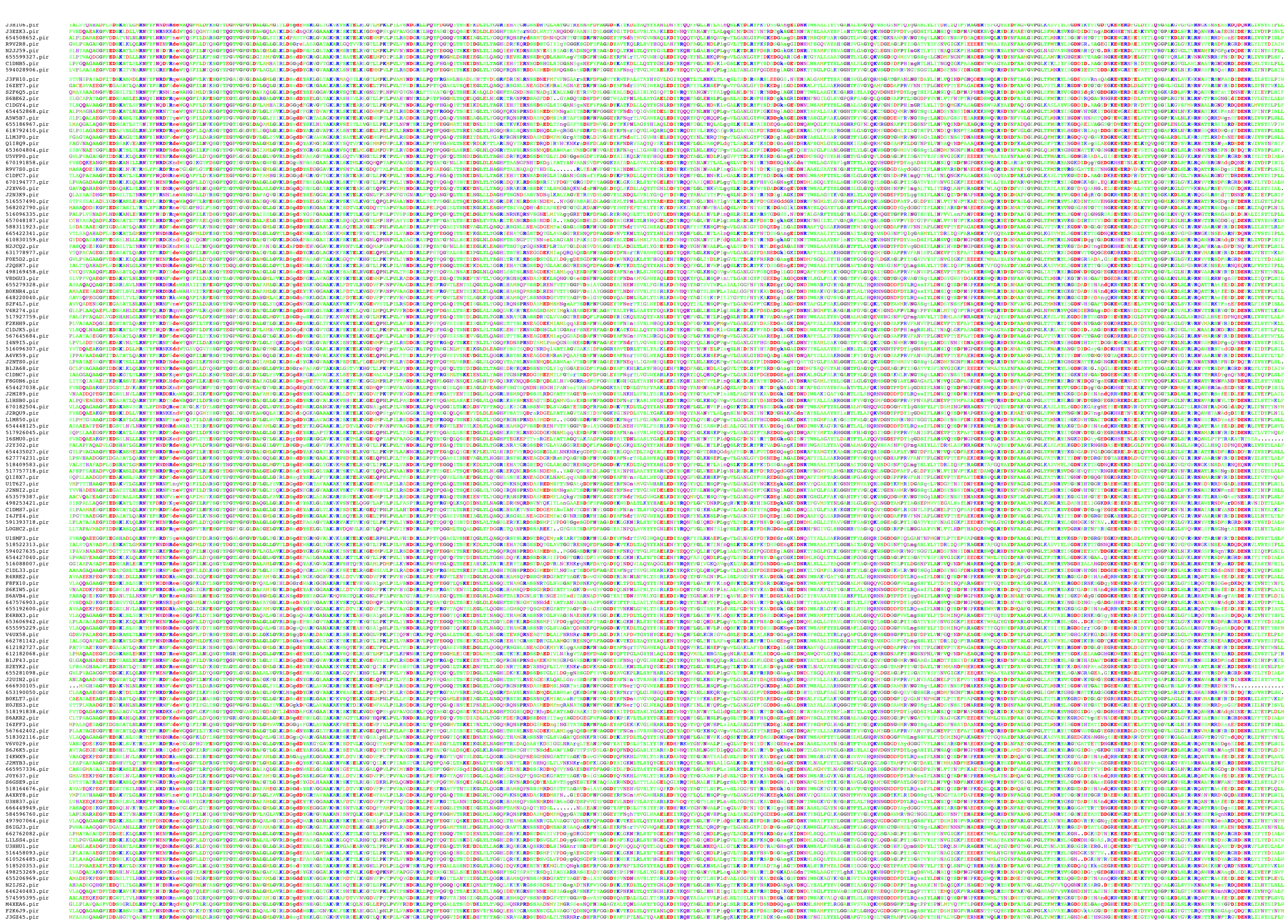

[illegible]

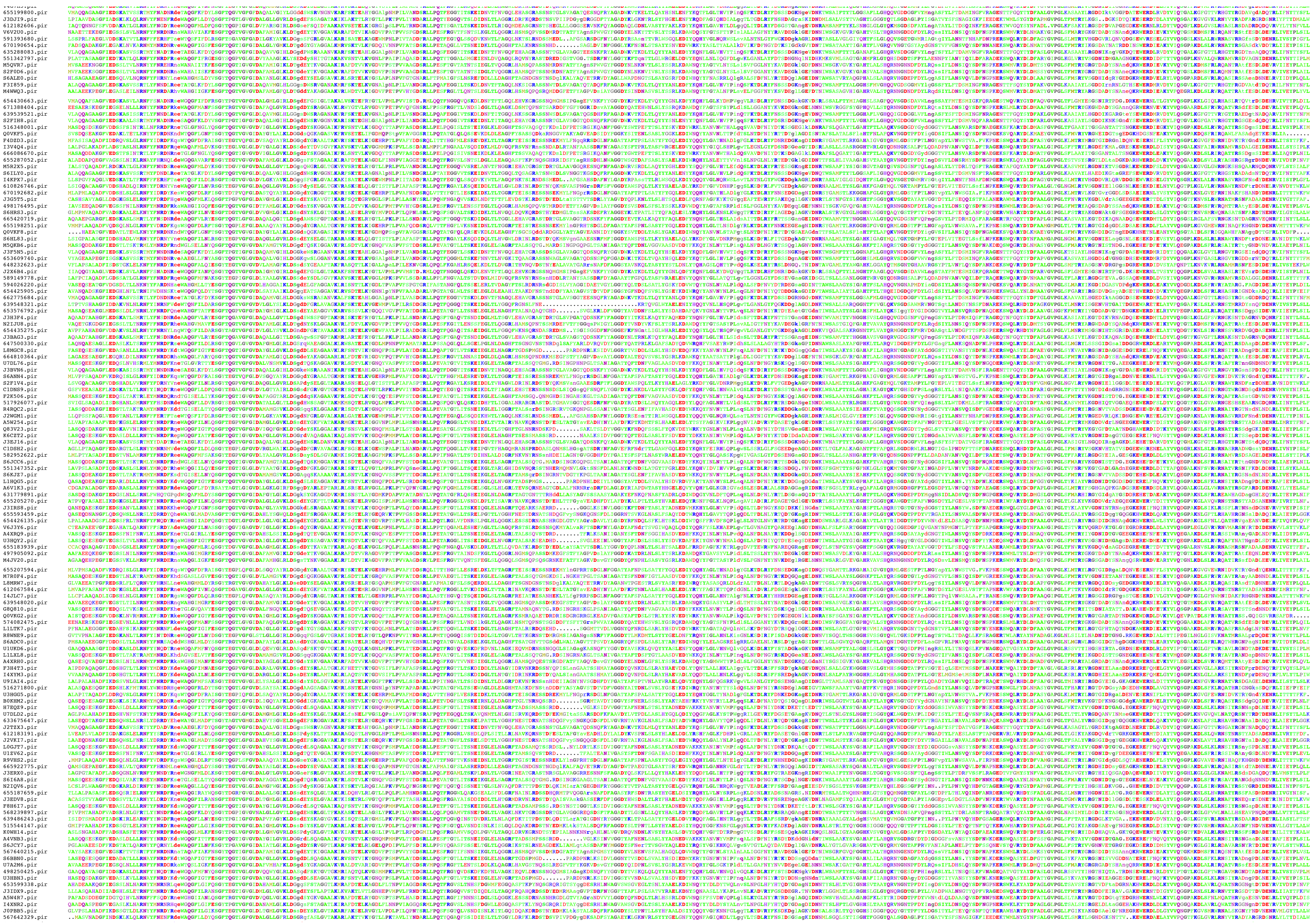

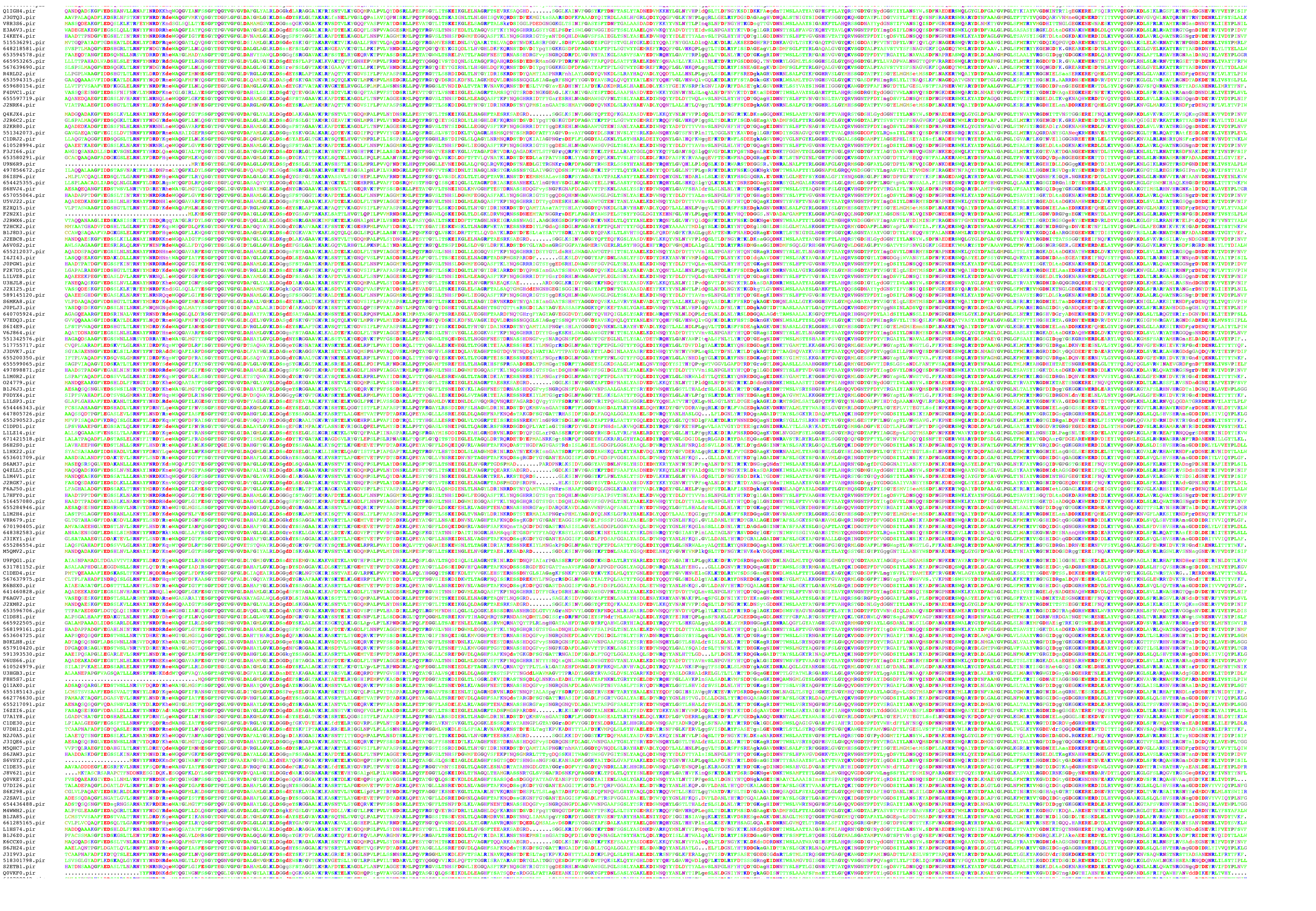

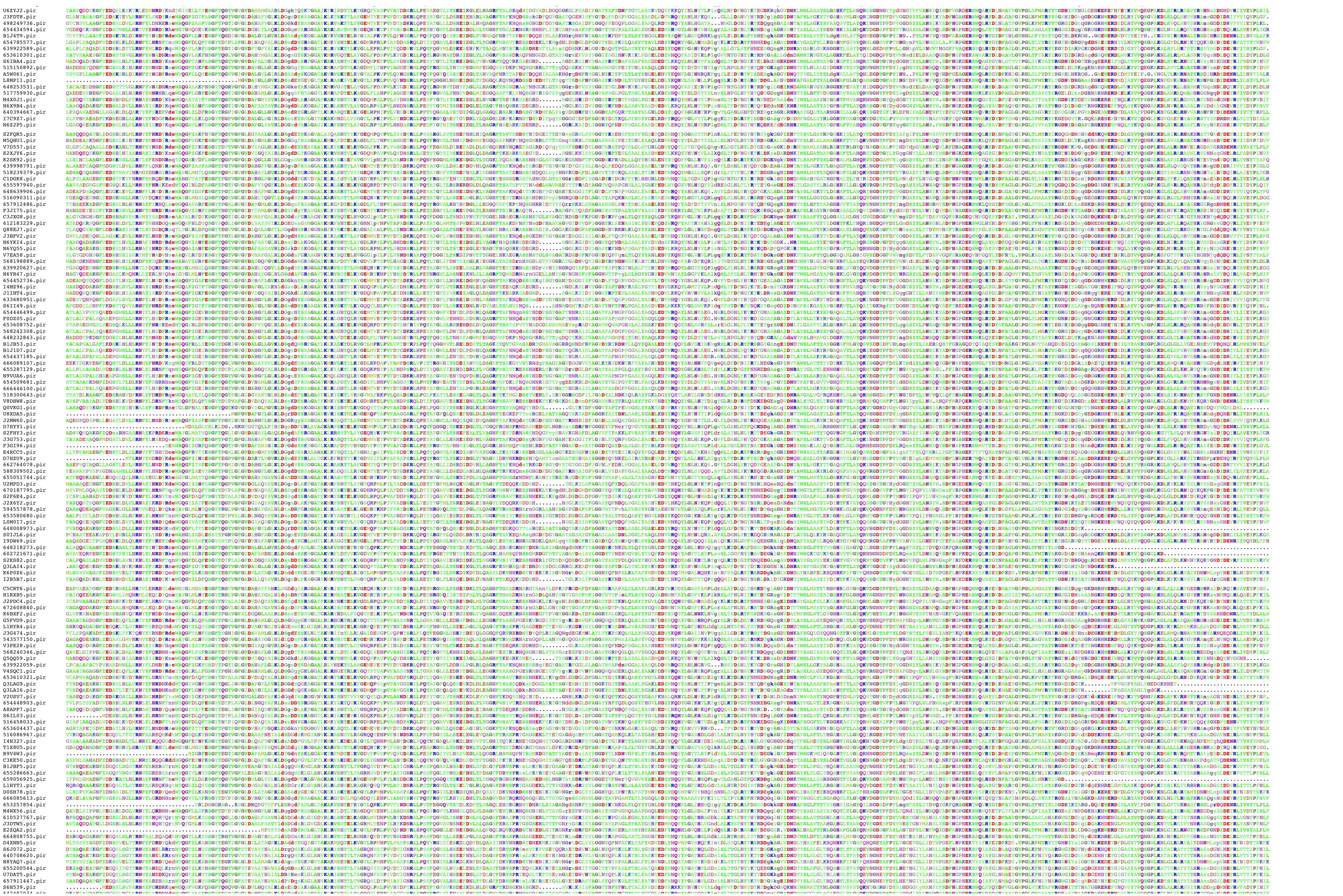

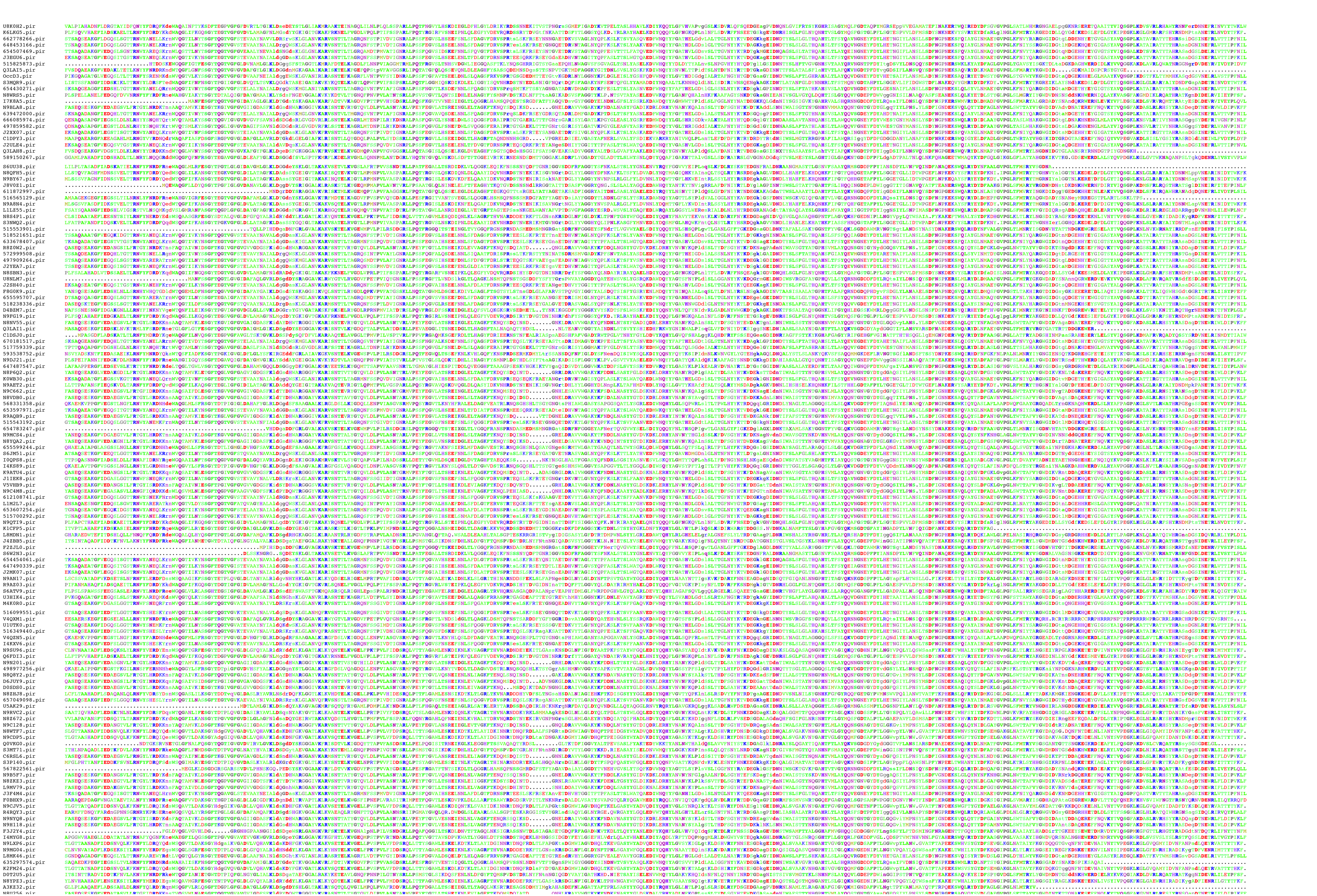

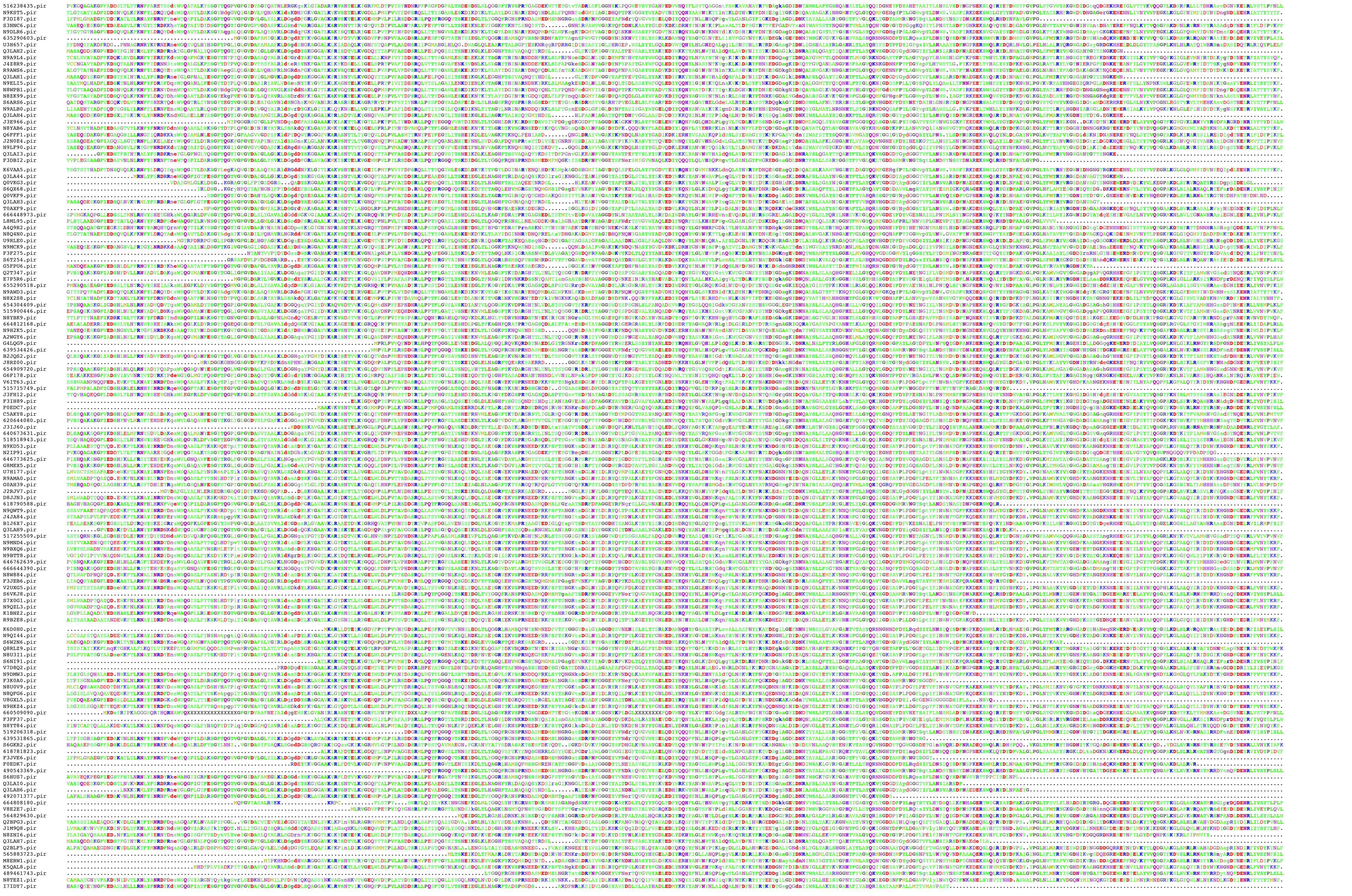

The top layer shows the consensus sequence. Residues are coloured after their physico-chemical properties. (GPX black; positive blue; negative red; CM yellow; H dark purple; NQ light purple; all others green)
