# Supplementary material for: In Silico Structure and Sequence Analysis of Bacterial Porins and Specific Diffusion Channels for Hydrophilic Molecules: Conservation, Multimericity and Multifunctionality
Source: Int J Mol Sci. 2016 Apr 21;17(4):599. doi: 10.3390/ijms17040599 (PMC4849052; doi:10.3390/ijms17040599)
Supplement: Supplementary file 1 [file ijms-17-00599-s001.zip › ijms-120323-Supplementary Materials/ijms-120323-Supplementary Materials.pdf]

Supplementary Materials: *In Silico* Structure and Sequence Analysis of Bacterial Porins and Specific Diffusion Channels for Hydrophilic Molecules: Conservation, Multimericity, and Multifunctionality

Hilde S. Vollan, Tone Tannæs, Gert Vriend and Geir Bukholm

Table S1. Porins divided into eight subclasses, based on structure (number of strands in the β-barrel), function (specific/non-specific) and multimeric state (monomeric/trimeric). The PDB ID lists the structure used to guide the MSA with structure templates listed first (template), followed by the available homologous structures used to update the initial structure alignment (other). The Superfamily column corresponds to the families found in the OMPdb with protein names in parenthesis [35].

Subclass Number	Porin Subclass Name	OMPdb Superfamily Name	Protein Name	PDB ID Template (Other Structures)	Number of β-Strands
1A	Non-specific, petite porin	The OmpA Family	OmpA	2K0L (1QJP)	8
3B	Oligogalacturonate-specific, small channel	Oligogalacturonate specific Porin Family	KdgM and NanC	4FQE (2WJR)	12
4A	Non-specific, intermediate porin	The OmpG Porin Family	OmpG	2IWV	14
5A	Non-specific, medium porin	General Bacterial Porin Family (GP-1 and GP-4)	OmpC, OmpK36, OmpF, PhoE, Omp32, RcGDP and RbGDP	2J1N (1PRN, 3UPG, 1OSM, 3NSG, 4GCS, 2POR, 2FGQ, and 1PHO)	16
5B	Sugar-specific, medium channel	Glucose-selective OprB Family	OprB	4GEY	16
5C	Phosphate-specific, medium channel	The Pseudomonas OprP Porin Family	OprP	2O4V	16
6B	Sugar-specific, large channel	Sugar Porin Family (Maltoporin and ScrY)	Maltoporin and ScrY	2MPR (1AF6 and 1OH2)	18
6C	Carboxyl-specific, large channel	The Outer Membrane Porin OprD Family	Occ Channels	3SZV (3SYS, 3SZD, 3T0S, 3T20, 3T24, 4FRT, 4FRX, 4FT6, 4FSO, 4FSP. 3JTY, 3SY7, 3SY9, and 3SYB)	18

Table S2. This table compares the whole protein structure analyses with core structure analyses (loops removed and only the barrel core left in the structure). The percent of aligned residues represents the average aligned residues for each pair-wise motif alignment divided by the smallest structure (which is the maximum possible aligned residues). The percent identities are calculated using the average sequence identity collected from every alignment. The raw data used to calculate these numbers are found in TableS2raw.

**Subclasses**		**Analysis Core Structure**				**Analysis Entire Structure**					
		**Mustang RMSD (Å)**	**% Residues Aligned (Mustang)**	**% Sequence Identity (Mustang)**	**% Sequence Identity (Clustal Ω)**	**Mustang RMSD (Å)**	**% Residues Aligned (Mustang)**	**% Sequence Identity (Mustang)**	**% Sequence Identity (Clustal Ω)**	**% Sequence Identity SwissProt (Clustal Ω)**	**Number of Sequences in MSA**
1A	Non-specific, petite porin	1.60	98.72	96.10	93.59	1.90	68.61	91.49	84.67	85.93	389
3B	Oligogalacturonate-specific, small channel	1.38	95.00	25.56	28.06	1.17	94.12	24.38	26.19	23.98	246
4A	Non-specific, intermediate porin	–	–	–	–	–	–	–	–	–	50
5A	Non-specific, medium porin	1.35	90.73	40.18	41.32	1.45	72.17	37.88	38.82	38.86	713
5B	Sugar-specific, medium channel	–	–	–	–	–	–	–	–	–	319
5C	Phosphate-specific, medium channel	–	–	–	–	–	–	–	–	–	160
6B	Sugar-specific, large channel	0.90	95.58	50.31	49.47	0.94	78.35	47.76	45.13	44.95	663
6C	Carboxyl-specific, large channel	0.94	96.16	46.44	45.68	1.09	90.75	44.83	43.69	41.67	1384

Table S3. Loop variability statistics derived from EVA. Extracellular loop residues form all eight subclasses (classification used as described in Table 3). Red is for those residues likely to be in the main active site; orange is the main active site; green is the regulatory site; yellow residues communicate and blue represents those residues with unknown function. Loop type: LL = large loop; SL = Small loop.

Number of Residues in Loop	Box 11	Box 12	Box 22	Box 23	Box 33	Subclass	Loop	Constriction Loop
25	–	–	1	7	17	1A	L1	No
18	1	–	1	5	11	1A	L2	No
20	–	–	1	6	13	1A	L3	No
21	–	–	3	10	8	1A	L4	No
2	–	–	1	–	1	3B	L1 (NanC)	No
1	–	–	1	–	–	3B	L2	No
2	–	–	–	–	2	3B	L3	No
14	–	–	–	1	13	3B	L4	No
10	–	–	–	–	10	3B	L5	No
13	2	–	6	2	3	3B	L6	No
9	–	3	4	2	–	4A	L1	No
12	–	1	4	5	2	4A	L2	No
6	–	–	1	1	4	4A	L3	No
10	–	2	3	2	3	4A	L4	No
10	–	1	3	5	1	4A	L5	No
13	–	1	4	7	1	4A	L6	No
5	–	–	3	2	–	4A	L7	No
7	–	–	1	6	–	5A	L1	No
8	–	–	3	5	–	5A	L2	No
34	3	4	13	13	1	5A	L3	Yes
24	–	1	10	8	5	5A	L4	No
14	–	1	4	2	7	5A	L5	No
10	–	–	2	4	4	5A	L6	No
8	–	–	1	6	1	5A	L7	No
17	–	2	6	8	1	5A	L8	No
8	–	1	–	6	1	5B	L1	No
27	1	–	11	15	–	5B	L2	Yes
37	2	1	12	21	1	5B	L3	Yes
18	–	1	2	13	2	5B	L4	No
27	–	–	4	20	3	5B	L5	No
7	–	–	2	4	1	5B	L6	No
30	–	1	7	17	5	5B	L7	No
9	–	2	1	5	1	5B	L8	No
9	–	–	–	3	6	5C	L1	No
10	–	–	3	5	2	5C	L2	No
34	3	4	3	15	9	5C	L3	Yes
2	–	–	–	–	2	5C	L4	No
45	–	–	9	11	25	5C	L5	No
7	–	–	3	2	2	5C	L6	No
15	–	1	9	2	3	5C	L7	No
8	–	–	1	4	3	5C	L8	No
25	5	–	6	11	3	6B	L1	Yes

11	–	–	2	8	1	6B	L2	No
18	1	–	12	5	–	6B	L3	Yes
18	–	–	2	9	7	6B	L4	No
13	–	–	2	4	7	6B	L5	No
35	–	–	13	14	8	6B	L6	No
5	–	–	–	–	5	6B	L7	No
3	–	–	–	1	2	6B	L8	No
27	–	–	12	10	5	6B	L9	No
7	–	–	–	2	5	6C	L1	No
20	–	–	1	1	18	6C	L2	No
17	2	–	7	5	3	6C	L3	Yes
22	–	–	1	10	11	6C	L4	No
2	–	–	1	1	–	6C	L5	No
8	–	2	1	3	2	6C	L6	No
28	1	–	5	20	2	6C	L7	Yes
9	–	–	1	4	4	6C	L8	No
5	–	–	–	3	2	6C	L9	No

Table S4. Extracellular loop statistics comparing the conservation of large *vs.* small loops. Constriction loops were excluded. Red is for those residues likely to be in the main active site; orange is the main active site; green is the regulatory site; yellow residues communicate and blue represents those residues with unknown function.

Extracellular Loops	Box 11	Box 12	Box 22	Box 23	Box 33	SUM
Large Loops	2<br>0.30%	7<br>1.06%	82<br>12.44%	136<br>20.64%	151<br>22.91%	57.36%
Small Loops	1<br>0.15%	13<br>1.97%	58<br>8.80%	120<br>18.21%	89<br>13.51%	42.64%

Table S5. Percentage of residues found in box in large or small loops. Red is for those residues likely to be in the main active site; orange is the main active site; green is the regulatory site; yellow residues communicate and blue represents those residues with unknown function.

Extracellular Loops	Box 11	Box 12	Box 22	Box 23	Box 33
Large Loops	2<br>66.67%	7<br>35.00%	82<br>58.57%	136<br>53.13%	151<br>62.92%
Small Loops	1<br>33.33%	13<br>65.00%	58<br>41.43%	120<br>46.88%	89<br>37.08%

Table S6. Constriction loop conservation statistics. Red is for those residues likely to be in the main active site; orange is the main active site; green is the regulatory site; yellow residues communicate and blue represents those residues with unknown function."

Subclass	Loop	Function	Box 11	Box 12	Box 22	Box 23	Box 33	Number of Residues
5A	L3	Constrict pore	3	4	13	13	1	34
5B	L2	Constrict pore	1	–	11	15	–	27
5B	L3	Constrict pore	2	1	12	21	1	37
5C	L3	Constrict pore	3	4	3	15	9	34
6B	L1	Constrict pore	5	–	6	11	3	25
6B	L3	Constrict pore	1	–	12	5	–	18
6C	L3	Constrict pore	2	–	7	5	3	17
6C	L7	Constrict pore	1	–	5	20	2	28
-	–	Sum residues	18	9	69	105	19	220

Text S1: Supplemental Information for Classes 1 to 6.

S1.1. Class 1: Petite Porins and Specific Diffusion Channels

Class 1A is composed of the smallest porins which are non-specific and include the OmpA porins. OmpA porins are abundant multifunctional porins involved in host evasion, binding to receptors and having nonspecific pore activity [22, 116]. Although OmpA homologue sequences are found across a wide range of species, only two structures currently exist in the PDB isolated from *E. coli* and *K. pneumonia* [117]. Class 1A OmpA porins have four long flexible extracellular loops involved in invasion (see Figure 9 and Table S3). The longest loop, L1, has the highest percentage of variability (25 residues long). Correlation between sequence variability and loop dynamics has also been identified in the literature [56]. *E. coli* is able to use OmpA loop variability to bind to various host receptors inducing different signaling patterns [118-121]. The most conserved loop residues are found in the shortest loop L2 (18 residues long). The residues found closest to the barrel are more conserved and are also found to be less motile in NMR studies [56]. The most surface-exposed residues situated at the tip of the loops are highly variable blue residues, which confirms that OmpA loops bind host receptors [8,22,119,122]. The distribution of residue conservation for the Class 1A analysis shows high variability of extracellular loops and some highly conserved lipid-facing residues (see Figures 6 and 7). Our findings support previous literature describing a conserved OmpA pore with respect to pore function [61,123]. The conserved residues Glu 69 and Tyr 20 cluster together, and facing the pore is probably important for pore function. Further laboratory work is needed to validate these functional residues and get a better understanding of these porins.

S1.2. Class 3: Small Porins and Specific Diffusion Channel

Hutter *et al.* [71] and Wirth *et al.* [24] conclude that acidic sugar-specific structures have two strings of basic residues facing each other across the pore that are likely to facilitate the diffusion of acidic oligosaccharides such as sialic acids of NanC and KdgM porins. Hutter *et al.* [71] term these two strings of charged residues lining the pore as track 1 and track 2. There are 13 basic residues facing the barrel core of the KdgM structure (PDB ID 4FQE, see Figure S1). Our analysis reveals track 2 to be the most conserved, indicating that this is the likely pathway found among KdgM homologous. This analysis indicates which basic residues are likely to have the most important functional role in the pore. Furthermore, the most conserved residues from this analysis are described as core residues by Hutter *et al.* [71]. The structure with the highest amount of loop residues was chosen for the loop variability analysis (PDB ID: 2WJR, see Figure 9).

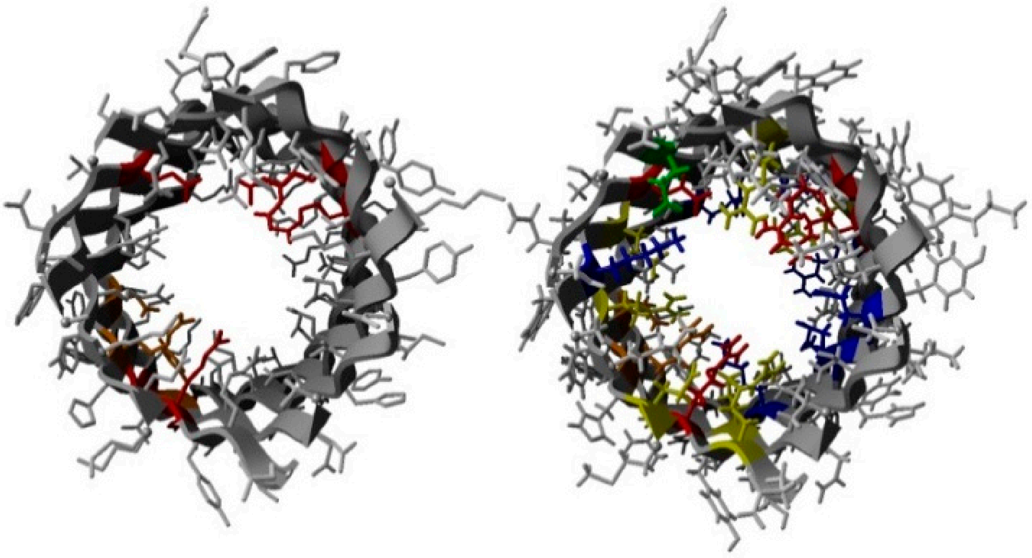

Figure S1. The Class 3B EVA and mapped onto the KdgM structure (PDB ID: 4FQE). The conserved basic residues highlighted to the left and all the basic residues facing the pore are colored to indicate residue conservation as described in the Materials & Methods section.

S1.3. Class 4: Intermediate Porins and Specific Diffusion Channels

Class 4A is a non-specific, intermediate specific porin subclass which includes the solved structure of the pH-dependent *E. coli* OmpG porin. OmpG is a rare rescue porin that can control membrane permeability in a pH-dependent manner using the extracellular loops under acidic conditions. The rest of the porin protein remains stable, demonstrating its tolerance to acidic environments. OmpG can also replace other protein channels (e.g., larger oligosaccharide-specific channels) if they are damaged or insufficient for nutrient uptake [43,44,75–77]. The Literature shows that loop L6 is involved in pH gating and, via H-bonding, extends from 10 to 17 residues [43]. Three loops are affected by pH-change (L1, L2 and L7) [77]. The pH-gated mechanism of OmpG has been explored in the literature, identifying L6 as being crucial to the mechanism [76]. Our analysis shows some degree of conservation in all loops, but no highly conserved loop residues were identified (Box 11 residues). There are six relatively conserved residues (Box 12) scattered throughout the extracellular loops and located between less conserved residues (from Box 22, 23 and 33). The longest loop has the most variable loops at the tip (see Figure 6). The highly variable residues are situated mainly at the top and bottom of the protein structure (including L3). Loop L1, L2, L4, L5 and L6 contain conserved residues (from Box 12) and are therefore of probable importance for the protein. The OmpG structure ranges in size from 5 to 13 residues in long extracellular loops. These results indicate that L4 and L5 have residues with important modulator roles that should be investigated by further experimental work. The pH gating of loop 6 does not show any significant conserved residues in our analysis, indicating a less important functional role for the Class 4A porins. In total, 50 sequences were used in this analysis. This class contains the fewest sequences. Precautions should therefore be taken when discussing this subclass, because 50 sequences is sub-optimal for running the analyses.

The importance of the aromatic and the charged residues found in the barrel pore of the OmpG structure has been discussed in the literature. Our data might shed light on which of these residues are of most importance and likely to be necessary for substrate translocation. Figure S4 shows a cluster of conserved residues (mainly charged) on one side of the barrel. The aromatic slide discussed by Yldiz *et al.* [75] seems to be of less importance than the charged residues found around the constriction site of the pore. Our analyses indicate a more communicative role rather than a substrate translocation role for all but three of the aromatic residues found in the pore (see Figure S2). The two histidine (His) residues discussed have a communicative role (Box 22), but they are probably not crucial to the function of Class 4A porins. The residues discussed by Yldiz as being involved

in substrate binding are conserved. None of these pore residues are highly mutable (Box 33). Instead, five of the eight substrate binding residues are found in Box 11 or 12. However, none of the "greasy slide" or "aromatic slide" residues discussed by Yldiz are highly conserved. Most of the residues found in this path are more likely to be involved in communicative roles rather than substrate translocation.

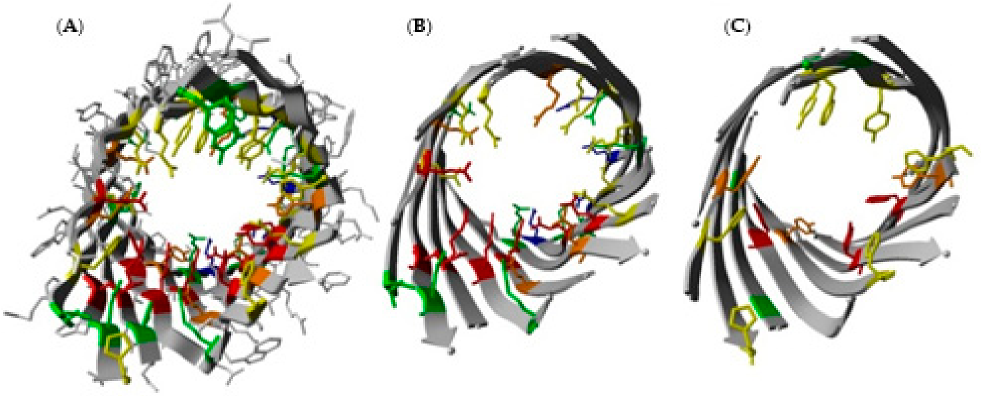

Figure S2. The Class 4A OmpG structure (PDB ID: 2IWV) showing the pore from the extracellular side. Turns and loops have been removed for clarity. (**A**) all residues are shown with aromatic and charged residues colored according to EVA result. The remaining residues are colored grey; (**B**) only charged residues facing the barrel pore are highlighted; (**C**) only aromatic residues facing the barrel pore are highlighted (including His residues).

S1.4 Class 5: Medium Porins and Specific Diffusion Channels

Class 5A NTP (non-specific trimeric porins) includes nine structures solved as trimeric units of classical porins (also known as general diffusion porins (GDP) or general bacterial porins (GBPs)). Trimeric porins are highly stable and detergent resistant. Only extreme environmental conditions are capable of dissociating them—for example, temperatures of 70 °C or higher [10]. Our analyses yield conserved residues forming the constriction site (with residues situated in loop L3 and the barrel wall facing this loop). Class 5A (NTP/GDP) porins have a cluster of conserved Arg residues found inside the pore, probably needed for substrate translocation of Class 5A porins [11]. Conserved residues are also found facing the lipid, which indicates GDP involvement in protein-protein interactions like BamA. Like Class 1A "non-specific, small porins", Class 5A porins show high loop-variability, which is likely to be a host evasion mechanism. Residues found in loops involved in host interactions are more variable and found in Box 33 [5]. Recent external loop deletion studies indicate that L1, L4 and L6 influence the antigenic structure in *Y. pseudotuberculosis* OmpF [124]. Stenkova *et al.* [125] identified niche adaptation in surface exposed OmpF loops (through sequence analyses of the gene).

Class 5B porins include the solved structure of the oligosaccharide specific OprB channel. The sugar-specific OprB channel can be compared to both Class 6B (maltoporins) and Class 5C (OprP). Class 6B channels also have a sugar preference, but Class 5B OprB channels are larger (18-stranded β-barrels). Class 5B and 6 are both 16-stranded β-barrels from *Pseudomonas*, but have different substrate specificities. Our analyses show that Class 5B have generally more conserved loops than the other subclasses. However, 119 extracellular loop residues are lacking from the OprB structure and were thus excluded when mapping the EVA result to the OprB structure. Most variation is observed in loops L4, L5 and L6 (see Table S5).

Both L2 and L3 loops are found inside the pore and form the constriction site [23,87]. These are the most conserved loops, indicating functionally important residues. The conserved residues found in the extracellular loops are probably involved in fetching the substrate (as observed in other specific protein channel subclasses). There are also conserved residues facing the membrane that will be discussed later. Our analyses show highly conserved residues clustered together facing the barrel interior and facing the membrane (see Figure S3). This is on the same side as the long extracellular loop and the opposite side of the β-bulge. Similar to Class 5A, Class 5B porins also have a longer loop

L3 inside the barrel (connected with a β-bulge). Conserved residues found inside the barrel are located in the constriction site. Figure S3 shows the conserved residues from the Class 5B analyses mapped onto the OprB structure.

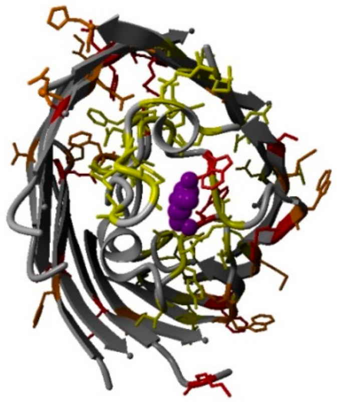

Figure S3. The Class 5B EV conservation results mapped on the Pseudomonas OprB specific monomeric structure (PDB ID: 4GEY) colored as described in the Materials & Methods section. Top-view zoomed into the pore. Extracellular loops and periplasmic turns were removed for clarity (loops L2 and L3 inside the pore are visible). Sugar molecule (BGC) is colored purple.

Class 5C includes the phosphate-specific channel. OprP is involved in high affinity uptake of phosphate mediated by an Arg ladder in the barrel pore [30,126]. The Class 5C MSA also included sequences from OprO, an OprP homologue. They are both phosphate specific, but exhibit a different preference in terms of substrate length. OprO has a higher preference for orthophosphate, while OprP prefers orthophosphate [91,127]. Our analysis yields high variability of Class 5C porins (see the EV-plot in Figure 5). Preliminary data indicate that *Pseudomondales* sp. cluster together and differ from other Class 5C sequences. Highly variable residues are found in the N-terminal region and extracellular loops. There are some relatively conserved (Box 22) residues found in the loops. The EV results show that the highly conserved residues are mainly bound to the constriction loop, while the loop itself contains yellow residues with communicative roles. Simulation studies have identified five residues in loop L3 that are involved in the constriction site. Four of these are found in an arginine ladder thought to be of importance for substrate translocation. This ladder starts at loop L5 and traverses through the pore, where a total of 13 residues have been identified [89]. These residues are highlighted in Figure S4. The L5 loop does not have any conserved Arg. The functional and communicative residues are found inside the pore, especially the highly conserved Arg 59. The strand residues that face the pore and interact with L3 or L5 probably have a greater functional role in Class 5B porins than previously discussed in the literature. There are eight acidic residues involved in the OprP phosphate translocation (loop L3 and L5; strand B1 and B2 [30]). EVA results show that the conserved residues are not found in the loops. They are typically strand residues interacting with the loops that form the constriction site (see Table S7 and Figure S4).

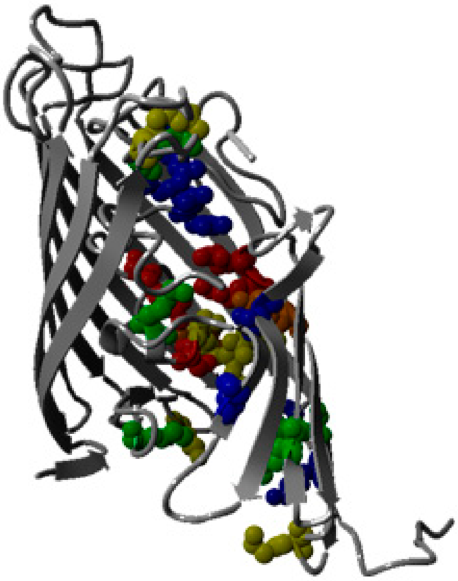

Figure S4. OprP residues colored according to Class 5C EV results. The residues highlighted correspond to the residues discussed by Pongprayoon *et al.* [89].

Table S7. This table combines the EV-result with the predicted function derived from the OprP structure [30].

Residue	Box	Function
R59, R60	11	Arginine ladder
R34, R218	22	Arginine ladder
R220	23	Arginine ladder
R222, R226, R242	33	Arginine ladder
R133, S124	11	Phosphate binding site
Y62	12	Phosphate binding site
R34, S125, K126	22	Phosphate binding site
K121	23	Phosphate binding site
D94	33	Phosphate binding site
K378	12	Lysine cluster
K25, K323	22	Lysine cluster
K30, K74, K313	23	Lysine cluster
K13, K15, K109	33	Lysine cluster

S1.7. Class 6: Large Porins and Specific Diffusion Channel

The trimeric sugar-specific channels are found in Class 6B. These porins are moderately specific for a certain group of substrates. Maltoporin and ScrY are involved in the translocation of maltose and sucrose respectively [128]. Like porins found in other subclasses, maltoporins are receptors for bacteriophages [129]. Currently, three structures of Class 6B porins are found in the PDB. The sugar-specific channels use the long, flexible loops to capture substrates [92]. However, the variable residues found among the conserved residues in these loops are likely needed for host and bacteriocins evasion [92]. Mutation analyses show that L4, L5 and L6 are needed for substrate binding in EcMaltoporin. The variable L9 loop indicates a protective role, because deletion would allow antibiotics to pass through and strongly impair maltodextrin binding [31,92,122]. The conserved residues cluster together on one side of the barrel. These 17 residues (bound to the wall and constriction loop, see Figure 7) are important for substrate translocation. There are also four conserved residues involved in the β-bulge in the transition between strand and constriction loop residues (see the right side of the pore in Figure 7).

Class 6C consists of the largest group with regard to both size and the number of sequences used. There are 15 solved porins in this subclass, all belonging to the OM Carboxylate Channels (Occ). Pseudomonas lack non-specific porins (like the trimeric NTP/GDPs found in other Gram negative bacteria) and have a tighter membrane with only specific channels in the OM (including OprP and OprB). This makes Pseudomonas sp. more resistant to antibiotics and other harmful toxins [28]. Like other specific channels, Class 6C proteins have many conserved loops. L3 and L7 fold into the barrel pore and form the constriction site [28]. Template structures used to represent the eight subclasses are listed in Table S2. Structures with the highest amount of solved loop residues were used in loop variability analysis (PDB ID 3JTY instead of the 3SZV template; see Figure 9). The extracellular loop L2 contains many conserved residues and no function for this loop can be found in the literature. However, like L5, L2 could be important for pore specificity. There might also be a structural role for this loop, e.g., stabilization of a possible trimer. Six conserved core residues are likely to be functionally important as part of the constriction site, creating substrate specificity. Like Class 6B, the β-bulge residues are conserved in the analyses. A cluster of conserved residues is found at the periplasmic end of the pore, indicating residues important for translocation. The conserved residues in this analysis do indicate a path for substrate translocation, because conserved residues are found throughout the pore.
